# The Morita–Baylis–Hillman reaction for non-electron-deficient olefins enabled by photoredox catalysis†

**DOI:** 10.1039/d1sc06784b

**Published:** 2022-01-05

**Authors:** Long-Hai Li, Hao-Zhao Wei, Yin Wei, Min Shi

**Affiliations:** State Key Laboratory of Organometallic Chemistry, Center for Excellence in Molecular Synthesis, Shanghai Institute of Organic Chemistry, University of Chinese Academy of Science, Chinese Academy of Sciences 345 Lingling Road Shanghai 200032 China weiyin@sioc.ac.cn mshi@mail.sioc.ac.cn; Key Laboratory for Advanced Materials, Institute of Fine Chemicals, School of Chemistry & Molecular Engineering, East China University of Science and Technology 130 Meilong Road Shanghai 200237 China

## Abstract

A strategy for overcoming the limitation of the Morita–Baylis–Hillman (MBH) reaction, which is only applicable to electron-deficient olefins, has been achieved *via* visible-light induced photoredox catalysis in this report. A series of non-electron-deficient olefins underwent the MBH reaction smoothly *via* a novel photoredox-quinuclidine dual catalysis. The *in situ* formed key β-quinuclidinium radical intermediates, derived from the addition of olefins with quinuclidinium radical cations, are used to enable the MBH reaction of non-electron-deficient olefins. On the basis of previous reports, a plausible mechanism is suggested. Mechanistic studies, such as radical probe experiments and density functional theory (DFT) calculations, were also conducted to support our proposed reaction pathways.

## Abstract 2

A strategy for overcoming the limitation of the Morita–Baylis–Hillman (MBH) reaction, which is only applicable to electron-deficient olefins, has been achieved *via* visible-light induced photoredox catalysis in this report.

## Graphical abstract

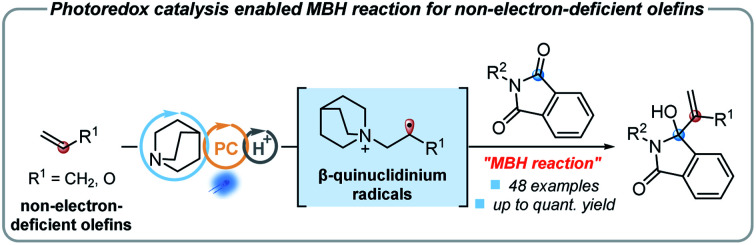

## Introduction

**Scheme 1:**
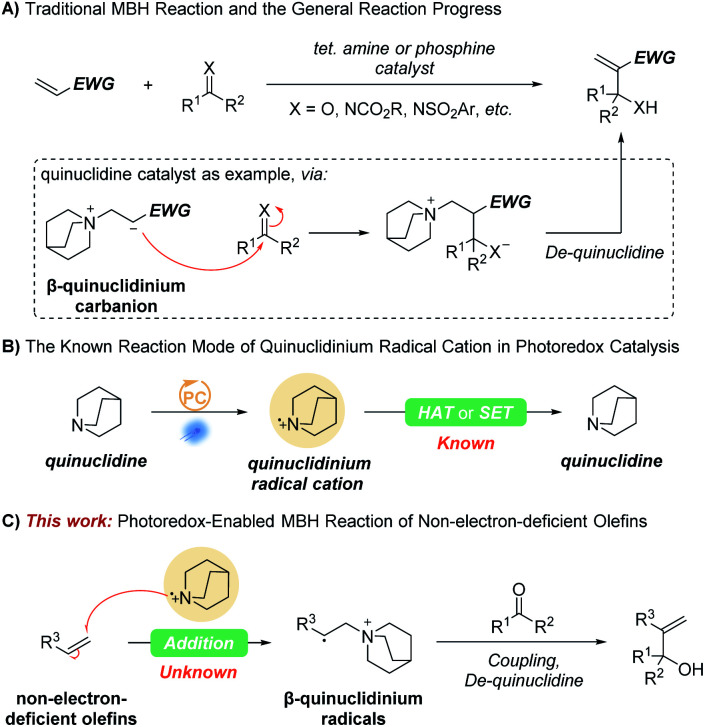
(A) Traditional MBH reaction and the general reaction progress. (B) The known reaction mode of quinuclidinium radical cation in photoredox catalysis. (C) This work: photoredox catalysis enabled MBH reaction of non-electron-deficient olefins.

The carbon–carbon bond-forming reaction is one of the most important transformations in organic chemistry, and therefore has been and remains an important and fascinating area in organic synthesis. Among these carbon–carbon bond-forming reactions, the Morita–Baylis–Hillman (MBH) reaction is one of the most useful and popular carbon–carbon bond-forming reactions with enormous synthetic utility, promise, and potential.^1–7^ Since the pioneering report presented by Morita in 1968 in the presence of tertiary phosphines and the similar tertiary amine catalyzed transformation described by Baylis and Hillman in 1972,^8,9^ the research on MBH reaction has grown exponentially over the past 50 years. Taking the quinuclidine catalyst as an example, the currently accepted mechanism of the MBH reaction involves a Michael addition of the catalyst at the β-position of the activated alkene to form an electron-withdrawing group (EWG) stabilized β-quinuclidinium carbanion zwitterion, which then reacts with the electrophilic carbonyl derivative to give another zwitterion that is deprotonated, and the catalyst is released to deliver the product (Scheme 1A). Though the scope of olefins has been expanded, the MBH reaction of non-activated olefins is still unknown. Thus, the discovery and development of complementary methods for non-electron-deficient olefins are meaningful and challenging.

Due to the possibility of groundbreaking synthetic transformation or more efficient alternative solutions, the synthetic chemistry community's interest in photocatalysis has enjoyed tremendous growth over the past decade. One of the most remarkable emerging features in this body of recent literature is the frequency with which dual catalysis approaches are utilized.^10–18^ Since first being developed in 2015 by MacMillan's group,^19^ quinuclidine and its derivatives as dual hydrogen atom transfer (HAT) catalysts in photoredox catalysis have enabled direct functionalization of substrates that are not readily oxidized by typical photocatalysts (Scheme 1B).^20–28^ In a few cases, a quinuclidinium radical cation also works as an oxidant that reacts with nucleophilic radicals or transient-metal intermediates through single electron transfer (SET).^29,30^ However, as an electrophilic species, the quinuclidinium radical cation addition to olefins has not yet been revealed. We suspect that the obtained β-quinuclidinium radical species, structurally similar to β-quinuclidinium carbanion zwitterions, may provide an opportunity to achieve the MBH reaction for non-electron-deficient olefins (Scheme 1C). Herein, we report our efforts to develop the first strategy that achieves the MBH reaction for non-electron-deficient olefins by introducing a novel photoredox-quinuclidine dual catalysis.

Based on the previous photoredox-quinuclidine dual catalysis, we selected Ir[dF(CF_3_)ppy]_2_(dtbbpy)PF_6_ (PC1) as the photocatalyst to oxidize quinuclidine and cyclopentene (1a) as the olefin partner to evaluate our working hypothesis. Fortunately, when using *N*-phenyl phthalimide (2a) with a higher reduction potential as the acceptor,^31–35^ we obtained the desired product 3aa. Based on the initial investigation, we further optimized the reaction conditions (Table 1) and found that the reaction efficiency was not affected by increasing the loading of quinuclidine (entries 1–4). Upon further evaluation, we observed that diluting the solution or extending the reaction time has a positive effect (entries 5 and 6). Under the current conditions, we determined that the reaction would not occur without the photocatalyst and quinuclidine or one of the two (entries 7–9). It has been disclosed in some reports that upon introducing the hydrogen-bonding effect in substrates containing phthalimide moieties, the reduction potential can be increased.^34,36^ Inspired by these findings, the introduction of a catalytic amount of Brønsted acids, such as AcOH, CF_3_CO_2_H, BzOH or TsOH, significantly increased the yield in a shorter reaction time (entries 10–13). By further diluting the solution, the yield of 3aa was slightly improved as well (entry 14). Further investigations focused on reducing the loading of 1a, the photocatalyst and quinuclidine. The results showed that the yield of 3aa was not affected when 1a was reduced to 5.0 equiv., but decreased when the loading was reduced to 2.0 equiv. (entries 15 and 16). When the loading of the photocatalyst was reduced to 1 mol% alone, the yield was not affected, but as the loading of the quinuclidine catalyst was simultaneously reduced, the yield decreased (entries 17 and 18). Then, combined with the above reaction conditions, we further examined the reaction time, and the results indicated that entry 21 had the best reaction conditions (entries 19–22). Lastly, changing the photocatalyst to 1,2,3,5-tetrakis(carbazol-9-yl)-4,6-dicyanobenzene (4CzIPN) did not afford a better reaction outcome (entry 23) (see the ESI† for a more detailed optimization of reaction conditions).

**Table 1:** Optimization of reaction conditions^a^

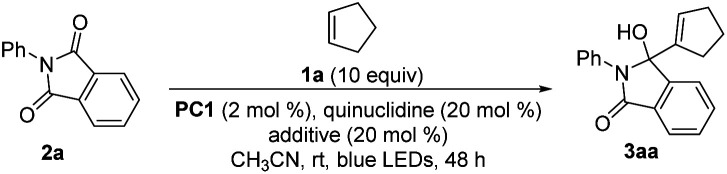					
Entry	Cond./(mol L^−1^)	PC	Quinuclidine/(mol%)	Additive/(mol%)	Yield^b^/(%)
1	0.2	PC1	20	—	35
2	0.2	PC1	50	—	36
3	0.2	PC1	100	—	36
4	0.2	PC1	150	—	36
5	0.2/3	PC1	20	—	50
6^c^	0.2/3	PC1	20	—	61
7^c^	0.2/3	W/o	W/o	—	0
8^c^	0.2/3	PC1	W/o	—	0
9^c^	0.2/3	W/o	20	—	0
10^d^	0.2/3	PC1	50	AcOH (20)	71
11^d^	0.2/3	PC1	50	CF_3_CO_2_H (20)	65
12^d^	0.2/3	PC1	50	BzOH (20)	71
13^d^	0.2/3	PC1	50	TsOH·H_2_O (20)	71
14^c^	0.05	PC1	20	—	67
15^c^^,^^e^	0.05	PC1	20	—	70
16^c^^,^^f^	0.05	PC1	20	—	54
17^c^	0.05	PC1 (1 mol%)	20	—	70
18^c^	0.05	PC1 (1 mol%)	10	—	54
19^e^^,^^g^	0.05	PC1 (1 mol%)	50	AcOH (20)	62
20^e^^,^^h^	0.05	PC1 (1 mol%)	50	AcOH (20)	64
*21* d ^,^ e	*0.05*	*PC1* *(1 mol%)*	*50*	*AcOH (20)*	*77 (76)*
22^e^^,^^i^	0.05	PC1 (1 mol%)	50	AcOH (20)	79
23^e^^,^^i^	0.05	4CzIPN	50	AcOH (20)	68

a Optimization reactions were performed on a 0.2 mmol scale.

b Yields were determined by ^1^H-NMR analysis of crude reaction mixtures relative to an internal standard.

c 72 h.

d 36 h.

e 5 equiv. of 1a.

f 2 equiv. of 1a.

g 12 h.

h 24 h.

i 60 h.

With the optimal conditions in hand, we investigated the applicability of this reaction. First, we investigated the scope of olefinic substrates, as shown in Table 2. In addition to cyclopentene, other cyclic olefins such as cyclohexene and cyclooctene could also perform well under the standard conditions, and the corresponding target product yields of 3ba and 3ca were 95% and 80%, respectively. Among them, the structure of 3ca was confirmed by X-ray single crystal diffraction. In addition to *N*-phenyl phthalimide 2a, *N*-methyl phthalimide 2b could also react with cycloheptene and cyclooctene efficiently by prolonging the reaction time. The desired products 3bb and 3cb were obtained with 98% and 87% yields, respectively. Next, we investigated the reactions of 2a and 2b with *n*-hexene 1d. Under the standard conditions, the target products 3da and 3db could be obtained in 63% and 39% yields, respectively. Comparing these reaction results of olefins with *N*-phenyl phthalimide 2a and *N*-methyl phthalimide 2b, we speculated that 2a has a higher reduction potential, which makes it easier to obtain an electron in the photoredox process, leading to higher reaction efficiency.^31^ For other olefins, such as 4-methyl-1-pentene, 4-phenylbutene and methyl 5-hexenoate, they could also react with 2a under the standard conditions, but with lower efficiency. The desired products 3ea–3ga were obtained in yields of 54%, 30% and 32%, respectively. Then we turned our attention to study the vinyl ether olefinic substrates. Although the substrates have C(sp^3^)–H bonds at the O-α position, the MBH reaction occurred selectively. Specifically, when the substituents on vinyl ethers are simple alkyl groups, the corresponding reaction products 3ha–3ka could be obtained in 87–95% yields. When the alkyl substituent contains an alkyl tertiary C–H bond, the reaction was almost unaffected, affording the desired product 3la in 90% yield. Using benzyl, 4-vinyloxy-butan-1-ol or cyclohexyl vinyl ether as the substrate, the reaction could also specifically furnish the target products 3ma–3pa in ≥68% yields. Among them, although the hydrogen atom at the α-position of the hydroxyl group has been proved to be captured by the quinuclidinium radical cation,^19^ the desired product 3oa could still be obtained in 81% yield. Finally, 1,2-dihydrofuran, a cycloalkenyl ether substrate, was also investigated, and we found that the corresponding product 3qa could be obtained in 66% yield.

**Table 2:** Scope of the olefins

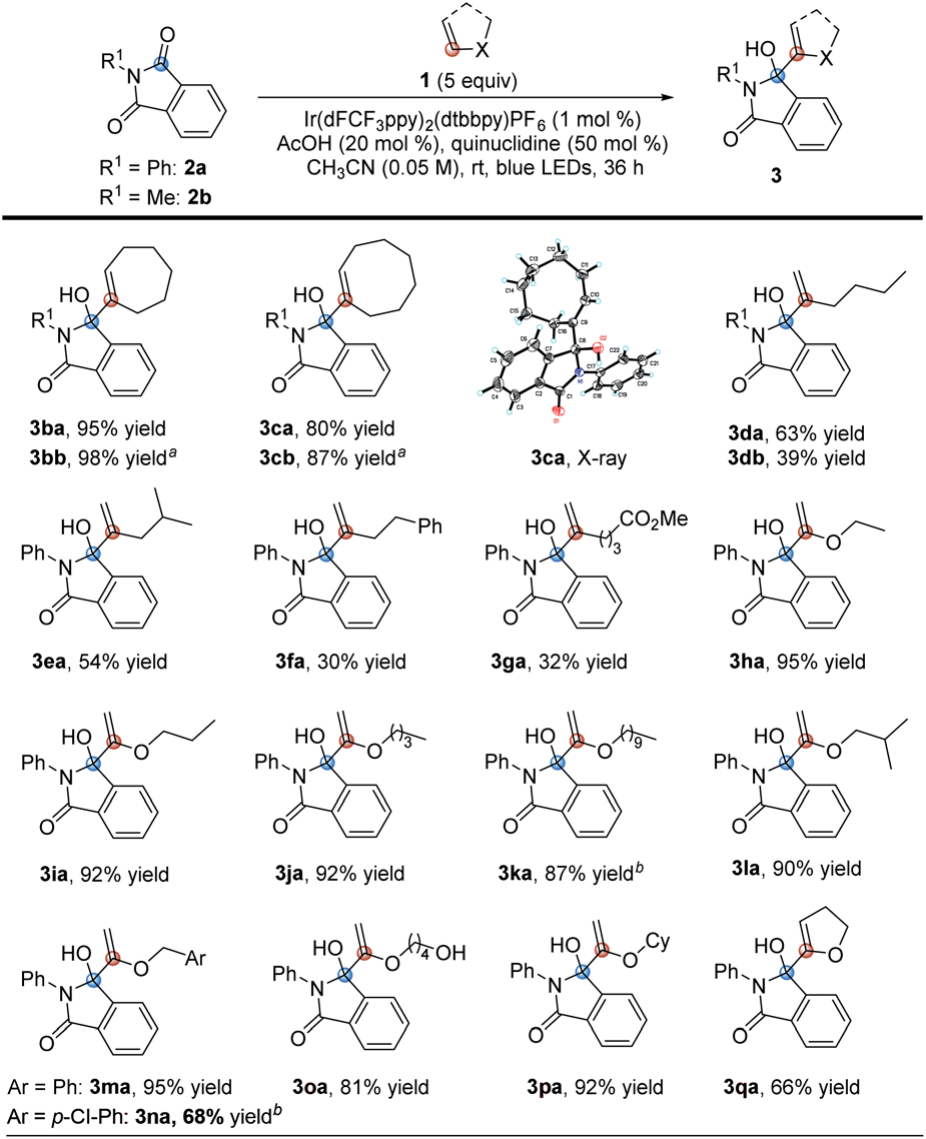

a 60 h.

b 1k or 1n (2.5 equiv.).

Next, we explored the suitability of phthalimide substrates in the reaction, as shown in Table 3. When the substituents on the nitrogen atoms of phthalimides were simple alkyl groups, the target products 3hb–3hd could be obtained in high yields ranging from 93% to 99%. The reaction could also tolerate some functional groups, such as fluoride, alkenyl, alkynyl, hydroxyl, methoxy and cyano, affording the desired products 3he–3hj in 89% to quantitative yields. The same results were obtained when the substituents are benzyl, allyl and propargyl groups (3hk–3hn). Even hydroxymethyl substituted phthalimide could also perform efficiently under these conditions to deliver the corresponding product 3ho with a yield of 63%. Its structure was determined by X-ray single crystal diffraction. Though the hydrogen atom at the acetal position can be easily abstracted by free radicals, the reaction of 1h with 2p also proceeded smoothly to afford the corresponding product 3hp with a quantitative yield. The α-amino carbonyl derivatives proved to be able to react efficiently under the standard conditions as well, furnishing the target products 3hq, 3hr and 3hs in 81% to quantitative yields. Furthermore, the ethoxyacyl and vinyl substituted phthalimides were also compatible, producing the desired products 3ht and 3hu in yields of 75% and 74% under the standard conditions, respectively. Moreover, halogen atoms were also tolerable in this reaction, and the desired products 3hv–3hx could be produced in 72% to 75% yields. As for 5-chloro or 5-bromo substituted substrates 2w or 2x, a regioisomeric mixture of 5- and 6-substituted products was obtained. Lastly, we investigated the unsubstituted phthalimide and electron-donating methoxyl group substituted phthalimide and found that the target products 3hy and 3hz (as a >10 : 1 regioisomeric mixture) could be obtained in 53% yield and 24% yield, respectively. These results may suggest that as the reduction potential increases, the yields of the corresponding product increased sequentially.

**Table 3:** Scope of the *N*-substituted phthalimides

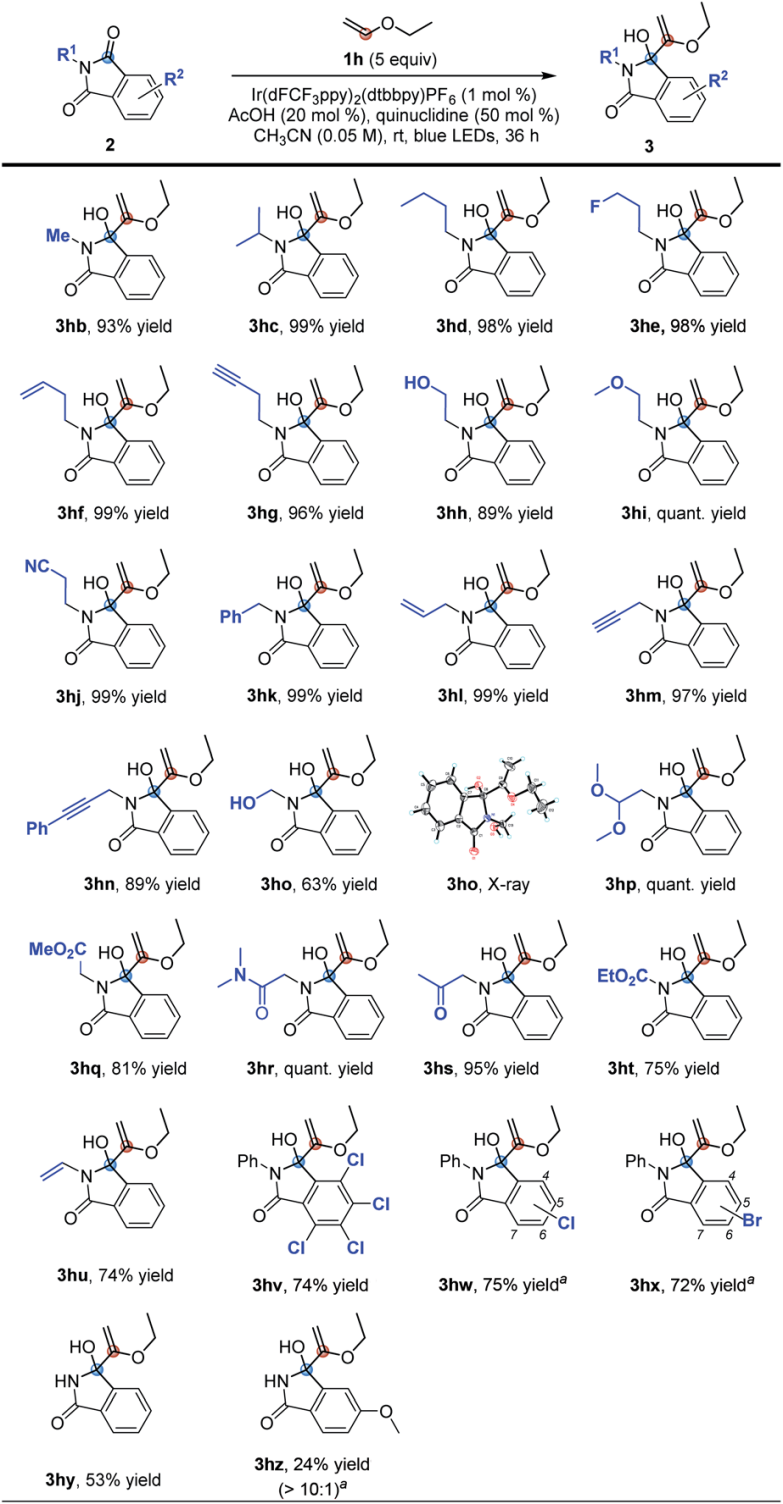

a A regioisomeric mixture of 5- and 6-substituted products.

**Scheme 2:**
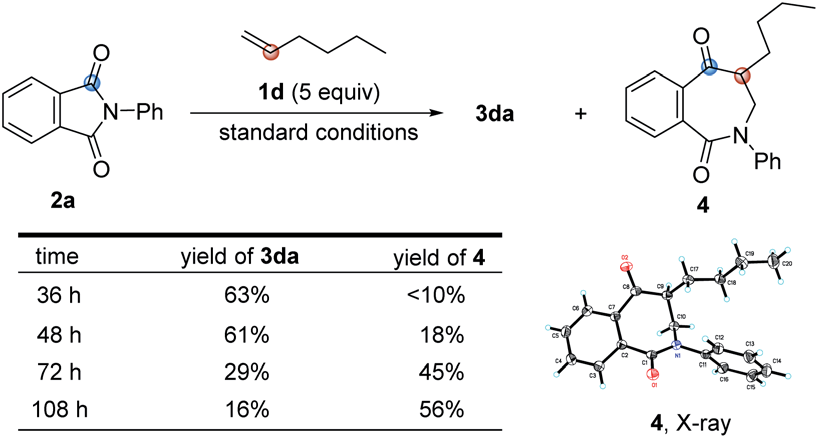
Ring-expanded product formed in the reaction.

**Scheme 3:**
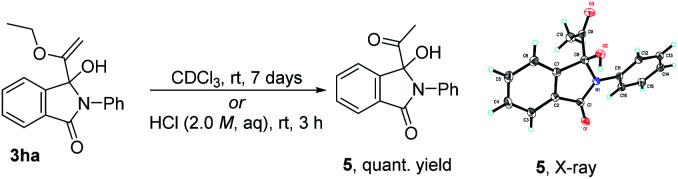
Transformation of product 3ha.

In the course of examining the substrate scope, an interesting result was obtained in the reaction of 2a with 1d, as shown in Scheme 2. Upon lengthening the reaction time, the yield of 3da decreased, along with increased yield of the ring-expanded product 4. We confirmed that the formation of 4 stemmed from the ring-opening and then re-closure of 3da under the standard conditions (see Scheme S4 in the ESI†). In addition, after placing product 3ha in deuterated chloroform for one week or in 2.0 M HCl aqueous solution for 3 h, the corresponding hydrolyzed product 5 was obtained in a quantitative yield, and its structure was determined by X-ray single crystal diffraction (Scheme 3).

In addition to phthalimides, we also investigated some other carbonyl compounds and found that phthalic anhydride could also undergo the same reaction. However, as shown in Table 4, the corresponding ring-opened adducts 7 rather than products 3 were produced in moderate yields due to the easy ring-opening of phthalic anhydride (for the detailed procedure, see Page S6 in the ESI†).

**Table 4:** Phthalic anhydrides as substrates

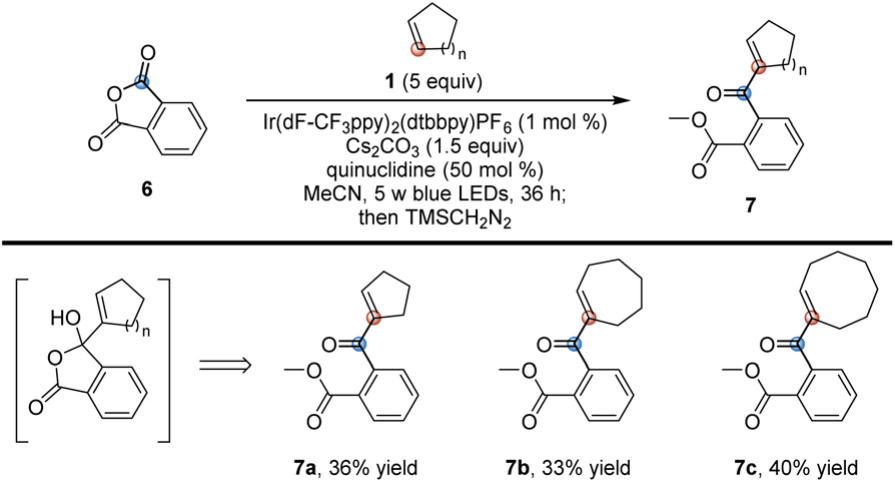

**Scheme 4:**
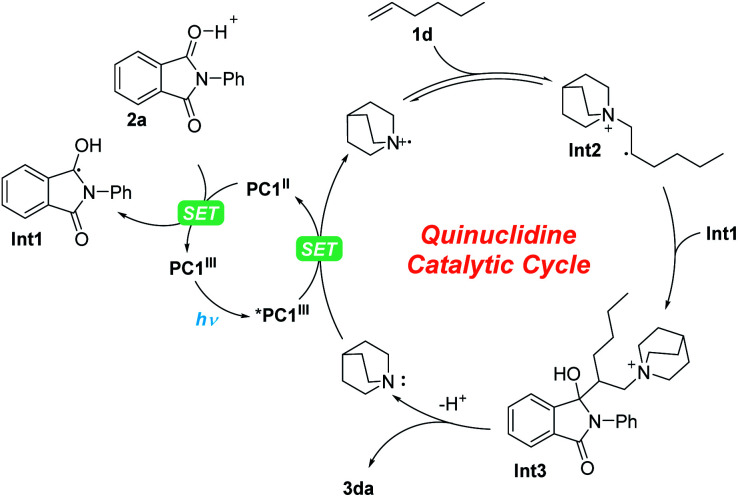
Proposed mechanism.

For this photoredox catalysis enabled MBH reaction, we proposed a reaction mechanism, as shown in Scheme 4. First, under visible-light irradiation, the photocatalyst PC1^III^ entered into the excited state *PC1^III^ (*E*^Ir(III)*/Ir(II)^_1/2_ = 1.21 V *vs.* SCE),^37^ which underwent a SET process with quinuclidine (*E*^ox^_p_ = 1.10 *vs.* SCE)^38,39^ to produce PC1^II^ and a quinuclidinium radical cation.^19^ Then, under the promotion of Brønsted acid, another SET process took place between PC1^II^ (*E*^Ir(III)/Ir(II)^_1/2_ = −1.37 V *vs.* SCE) and *N*-phenyl phthalimide 2a (*E*^red^_p/2_ = −1.31 V *vs.* SCE)^31^ to obtain the radical intermediate Int1. On the other hand, the β-quinuclidinium radical intermediate Int2 was obtained from the addition of the quinuclidinium radical cation with olefin 1d. Subsequently, owing to the persistent radical effect (PRE),^40,41^ the persistent radical intermediate Int1 underwent radical–radical coupling with Int2, giving intermediate Int3, which afforded branched olefin 3da and recovered quinuclidine after elimination.

**Scheme 5:**
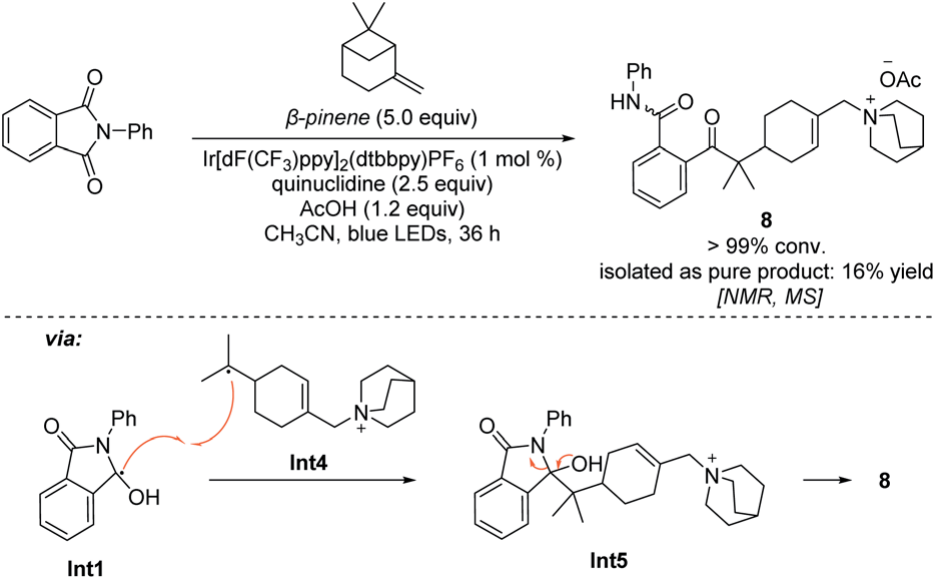
Radical probe experiment.

**Scheme 6:**
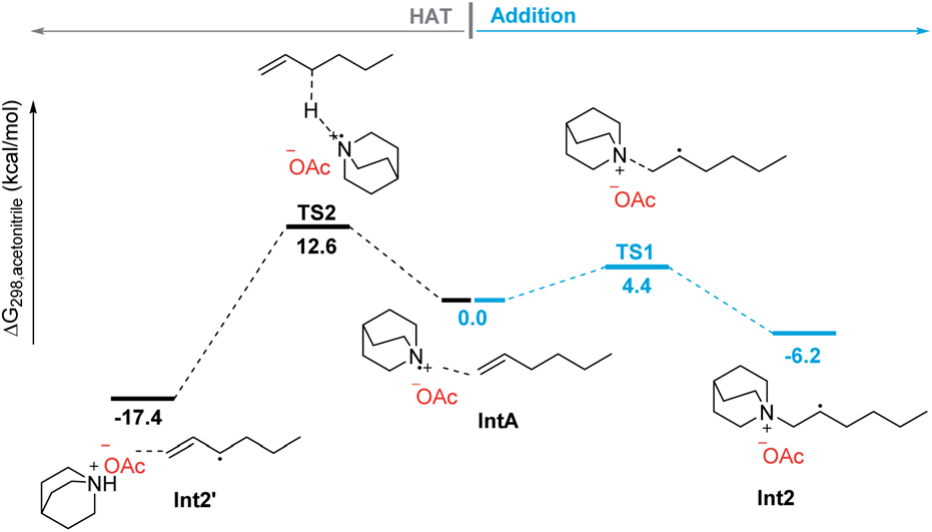
DFT calculations for radical addition and HAT processes.

The addition process of a quinuclidinium radical cation with olefins has not yet been reported. However, recently it has been reported that primary and secondary alkyl amines are involved in the hydroamination of non-activated olefins.^42–44^ In these processes, carbon–nitrogen bond formations proceed through key aminium radical intermediates that are generated *via* a SET process between the excited-state photocatalyst and amine substrates. Furthermore, several recent reports also suggested that a tertiary aminium radical derived from Selectfluor could also undergo the addition to olefins for the catalytic oxidative functionalization of alkenes.^45–47^ Thus, the quinuclidinium radical intermediate derived from quinuclidine as a tertiary amine should also perform the addition process with olefins. In order to detect this mechanistic paradigm, a radical probe experiment was designed. As shown in Scheme 5, we expected that β-pinene reacted with the quinuclidinium radical cation, giving intermediate Int4, which underwent coupling with Int1 followed by a ring-opening process *via* intermediate Int5 to afford a quinuclidinium salt 8 (conv. > 99%). Its structure was determined by NMR, 2D-NMR and MS spectroscopy (see Pages S90–S95 in the ESI†). In addition, our mechanistic hypothesis was also supported by density functional theory (DFT) calculations (see the ESI†). Compared with the 12.6 kcal mol^−1^ energy required for the HAT process through transition state TS2, the addition process only needs to overcome a 4.4 kcal mol^−1^ energy barrier *via* transition state TS1 to give the β-quinuclidinium radical adduct, suggesting that the addition process between the quinuclidinium radical intermediate with *n*-hexene is superior to the potential HAT process (Scheme 6). Based on the DFT calculation results, we disclose that the presence of the OAc^−^ anion in the catalytic system not only stabilizes the key intermediates but also promotes the deprotonation and catalyst elimination step (for details, see Scheme S5 in the ESI†). However, the subsequent KIE studies revealed that *k*_H_/*k*_D_ was 1.04 in the reaction of 2a with 1k, indicating that the breaking of the carbon–hydrogen bond is not involved in the rate-determining step (for details, see Page S96 in the ESI†). This observation reveals that the product yield is independent of the counter anions shown in Table 1, entries 10–13.

## Conclusions

On the basis of the *in situ* formed key temporary β-quinuclidinium radical intermediates, this newly developed photoredox catalytic reaction upon visible-light irradiation overcame a long-standing limitation of the MBH reaction, which is only applicable to electron-deficient olefins. More importantly, we are optimistic that this protocol will serve as the basis for future work in the area of selective C(sp^2^)–H bond functionalization of olefins. Further investigations are ongoing in our laboratory.

## Data availability

Experimental and computational data have been made available as ESI.†

## Author contributions

Long-Hai Li discovered the reactions. Min Shi and Long-Hai Li designed the experiments. Long-Hai Li and Hao-Zhao Wei performed and analyzed the experimental results. Min Shi and Long-Hai Li wrote the manuscript. Yin Wei performed and described the computational section.

## Conflicts of interest

There are no conflicts to declare.

## Supplementary Material

SC-013-D1SC06784B-s001

SC-013-D1SC06784B-s002
