# Supplementary material for: The Morita–Baylis–Hillman reaction for non-electron-deficient olefins enabled by photoredox catalysis
Source: Chem Sci. 2022 Jan 5;13(5):1478–83. doi: 10.1039/d1sc06784b (PMC8809420; doi:10.1039/d1sc06784b)
Supplement: SC-013-D1SC06784B-s001 [file SC-013-D1SC06784B-s001.pdf]

Electronic Supplementary Material (ESI) for Chemical Science.
This journal is © The Royal Society of Chemistry 2021

Supporting Information

Morita-Baylis-Hillman Reaction for Non-electron-deficient Olefins Enabled by Photoredox Catalysis

Long-Hai Li[a], Hao-Zhao Wei[b], Yin Wei*[a], and Min Shi*[a,b]

[a]State Key Laboratory of Organometallic Chemistry, Center for Excellence in Molecular Synthesis, Shanghai Institute of Organic Chemistry, University of Chinese Academy of Science, Chinese Academy of Sciences, 345 Lingling Road, Shanghai 200032 China. *weiyin@sioc.ac.cn; *mshi@mail.sioc.ac.cn.

[b]Key Laboratory for Advanced Materials and Institute of Fine Chemicals, School of Chemistry & Molecular Engineering, East China University of Science and Technology, 130 Meilong Road, Shanghai 200237, China.

Table of Contents

(A) General Information 2
(B) Reaction Setup 3
(C) Reaction Optimization and Controls 4
(D) General Procedure for the Photoredox Enabled MBH Reaction 6
(E) Ring Expansion Reaction 8
(F) Characterization Data 11
(G) X-ray Crystal Data of Compounds **3ho**, **3ca**, **4** and **5** 87
(H) Radical Probe Experiment 90
(I) Kinetic Isotope Effect (KIE) Study of the Reaction between **2a** and **1k** 96
(J) Computational Details 98
(K) References 109

(A) General Information

Melting points were determined on a digital melting point apparatus and temperatures were uncorrected. Proton nuclear magnetic resonance (^{1}H NMR) spectra and carbon nuclear magnetic resonance (^{13}C NMR) spectra were recorded at 400 and 100 MHz, respectively. ^{1}H NMR spectrum uses TMS (δ = 0.00 ppm) as internal standard, ^{13}C NMR spectrum uses $CDCl_3$ (δ = 77.00 ppm), DMSO-d_6 (δ = 40.00 ppm), Acetone-d_6 (δ =29.00 ppm) or CD_2Cl_2 (δ = 53.50 ppm) as internal standard. Data are presented as follows: chemical shift (ppm), multiplicity (s = singlet, d = doublet, t = triplet, q = quartet, m = multiplet), coupling constants in Hertz (Hz) and integration. IR spectra were recorded on a Perkin-Elmer PE-983 spectrometer with absorption in cm^{-1}. High Resolution Mass Spectra (HRMS) were recorded by ESI method. The employed solvents were dry up by standard methods when necessary. Commercially obtained reagents were used without further purification. For thin-layer chromatography (TLC), silica gel plates (Huanghai GF254) were used. Flash column chromatography was carried out using 300-400 mesh silica gel at increased pressure.

Substrates **1a**-**1j** and **1l**-**1p** are commercially available. Substrates **1k** and **1k**-**D** were synthesized by the procedures reported in the previous literature.[1-2]

(B) Reaction Setup

8W LED strip (1 meter, NVC® Lighting) was purchased from https://nvc.tmall.com/. The strip was wound in two circles and equipped with a fan. The 5 mL sealed tubes used in this report are made by Shanghai Institute of Organic Chemistry.

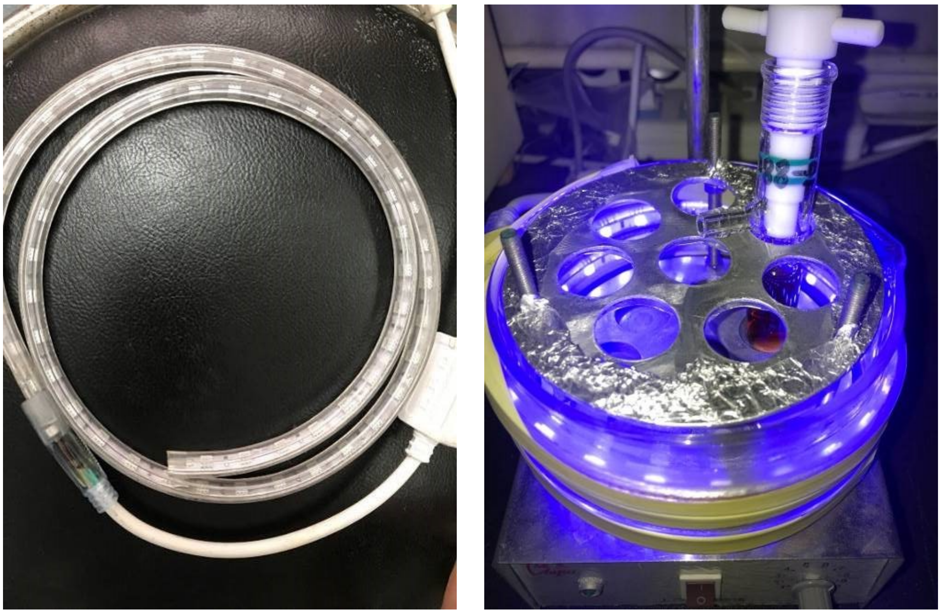

Figure S1. 8 W LEDs Strip and Reaction Setup

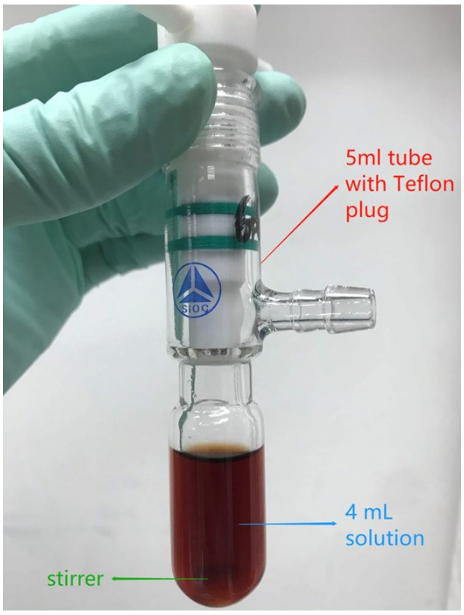

Figure S2. Reaction Tube (after reaction)

(C) Reaction Optimization and Controls

Table S1. Optimization of Photocatalysts and the Loading of Quinuclidine

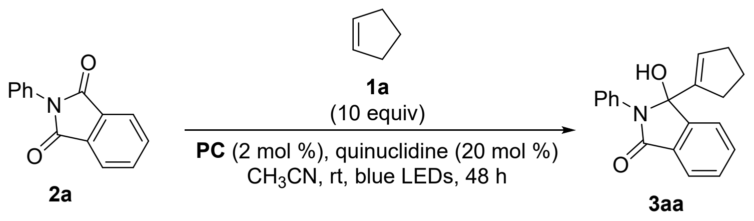

entry	solvent	cond. /(mol/L)	PC	quinuclidine /(mol %)	additive /(mol %)	Yield/(%)[a]
1	CH_3CN	0.2	**PC1**	20	-	35
2	CH_3CN	0.2	$Ir(ppy)_2(dtbbpy)PF_6$	20	-	15
3	CH_3CN	0.2	$Ru(bpy)_3(PF_6)_2$	20	-	0
4	CH_3CN	0.2	$Mes\text{-}Acr^+ClO_4^-$	20	-	0
5	CH_3CN	0.2	4CzIPN	20	-	33
6[b]	CH_3CN	0.2	**PC1**	20	-	41
7[c]	CH_3CN	0.2	**PC1**	20	-	46
8	CH_3CN	0.2	**PC1**	50	-	36
9	CH_3CN	0.2	**PC1**	100	-	36
10	CH_3CN	0.2	**PC1**	150	-	36

[a]Yields were determined by ^{1}H-NMR analysis of crude reaction mixtures relative to an internal standard. [b]100 W LEDs lamp. [c]96 h. **PC1** = $Ir[dF(CF_3)ppy]_2(dtbbpy)PF_6$.

Table S2. Optimization of Solvents

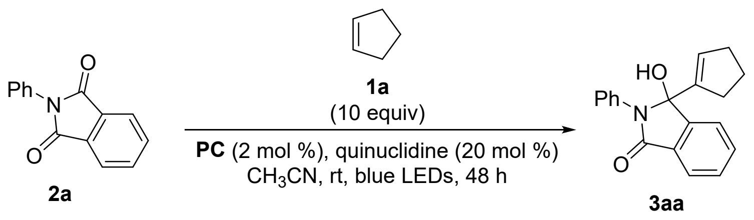

entry	solvent	cond. /(mol/L)	PC	quinuclidine /(mol %)	additive /(mol %)	Yield/(%)[a]
1	CH_3CN	0.2	**PC1**	20	-	35
2	DMSO	0.2	**PC1**	20	-	38
3	DMA	0.2	**PC1**	20	-	18
4	DMF	0.2	**PC1**	20	-	32
5	acetone-d_6	0.2	**PC1**	20	-	trace
6	THF	0.2	**PC1**	20	-	trace
7	DCE	0.2	**PC1**	20	-	0
8	TFE	0.2	**PC1**	20	-	trace

[a]Yields were determined by ^{1}H-NMR analysis of crude reaction mixtures relative to an internal standard. **PC1** = $Ir[dF(CF_3)ppy]_2(dtbbpy)PF_6$.

Table S3. Further Optimization of Reaction Conditions

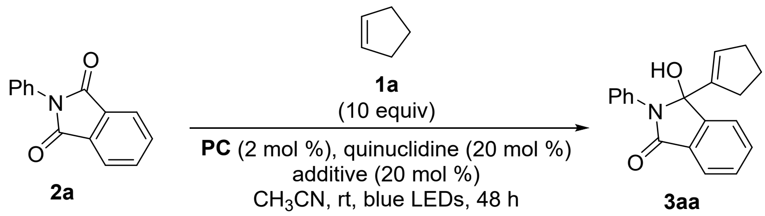

entry	solvent	cond. /(mol/L)	PC	quinuclidine /(mol %)	additive /(mol %)	Yield/(%)[a]
1	CH_3CN	0.2/3	**PC1**	20	-	50
2[c]	CH_3CN	0.2/3	**PC1**	20	-	61
3[c]	CH_3CN	0.2/3	**PC1**	50	-	62
4[c]	CH_3CN	0.2/3	**PC1**	100	-	62
5[c]	CH_3CN	0.2/3	**PC1**	4-CN-quinuclidine(20)	-	0
6[c]	CH_3CN	0.2/3	**PC1**	Thianthrene (100)	-	0
7[c]	CH_3CN	0.2/3	4CzIPN	20	-	55
8[c]	CH_3CN	0.2/3	w/o	w/o	-	0
9[c]	CH_3CN	0.2/3	**PC1**	w/o	-	0
10[c]	CH_3CN	0.2/3	w/o	20	-	0
11[c,d]	CH_3CN	0.2/3	**PC1**	20	-	58
12[e]	CH_3CN	0.2/3	**PC1**	50	AcOH (20)	71
13[e]	CH_3CN	0.2/3	**PC1**	50	CF_3CO_2H (20)	65
14[e]	CH_3CN	0.2/3	**PC1**	50	BzOH (20)	71
15[e]	CH_3CN	0.2/3	**PC1**	50	TsOH.H_2O (20)	71
16[c]	CH_3CN	0.05	**PC1**	20	-	67
17[b]	CH_3CN	0.05	**PC1**	20	-	64
18[c,d]	CH_3CN	0.05	**PC1**	20	-	70
19[c,f]	CH_3CN	0.05	**PC1**	20	-	54
20[c]	CH_3CN	0.05	**PC1** (1 mol %)	20	-	70
21[c]	CH_3CN	0.05	**PC1** (1 mol %)	10	-	54
22[d,g]	CH_3CN	0.05	**PC1** (1 mol %)	50	AcOH (20)	62
23[d,h]	CH_3CN	0.05	**PC1** (1 mol %)	50	AcOH (20)	64
24[d,e]	***CH_3CN***	***0.05***	***PC1 (1 mol %)***	***50***	***AcOH (20)***	***77 (76[j])***
25[d,i]	CH_3CN	0.05	**PC1** (1 mol %)	50	AcOH (20)	79
26[d,i]	CH_3CN	0.05	4CzIPN	50	AcOH (20)	68

[a]Yields were determined by ^{1}H-NMR analysis of crude reaction mixtures relative to an internal standard. [b]100 W LEDs lamp. [c]72 h. [d]5.0 equiv. [e]36 h. [f]2.0 equiv. [g]12 h. [h]24 h. [i]60 h. [j]isolated yield. **PC1** = Ir[dF(CF_3)ppy]$_2$(dtbbpy)PF_6.

(D) General Procedure for the Photoredox Enabled MBH Reaction

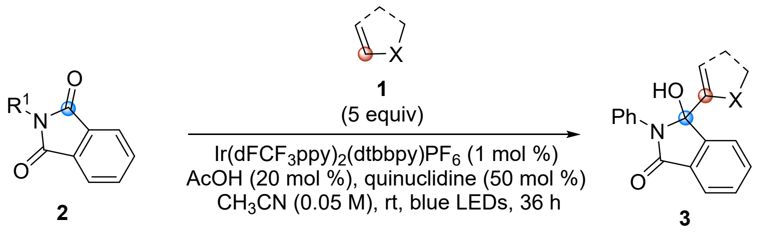

A 5 mL dry sealed tube equipped with a Teflon plug and a magnetic stirrer bar was charged with Ir[dF(CF$_3$)ppy]$_2$(dtbbpy)PF$_6$ (0.002 mmol, 0.01 equiv) and phthalimide **2** (0.2 mmol, 1.0 equiv). After replacing the air in it with argon, 4.0 mL of dry acetonitrile, which has been degassed with argon, was injected under argon. Then, also under argon, 100 μL of quinuclidine acetonitrile solution (1.0 M, without molecular oxygen), 2.3 μL of glacial acetic acid (0.04 mmol, 0.2 equiv) and olefin **1** (1.0 mmol, 5 equiv) were injected with micro-injectors, respectively. After all the materials were added, the reaction tube was sealed with a Teflon plug under argon. Then, the reaction tube was placed under the blue light of an 8 W blue LED strip at room temperature (using a fan to maintain the temperature). After stirring for 36 hours under these conditions, the mixture was concentrated directly on a rotary evaporator. The corresponding residue was diluted by dichloromethane and purified directly by a column chromatography (SiO$_2$) using ethyl acetate/petroleum ether as the eluent to obtain the desired product **3** of the MBH reaction.

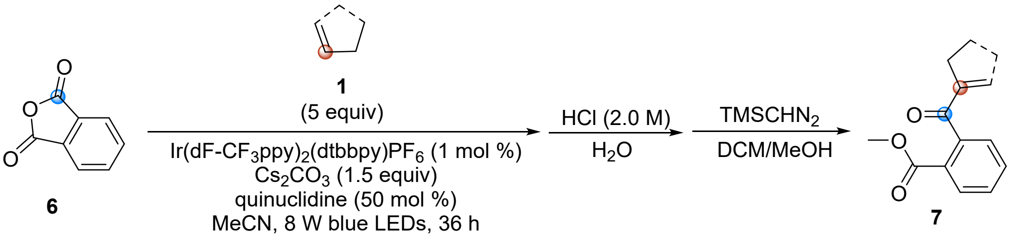

A 5 mL dry sealed tube equipped with a Teflon plug and a magnetic stirrer bar was charged with Ir[dF(CF$_3$)ppy]$_2$(dtbbpy)PF$_6$ (0.002 mmol, 0.01 equiv), Cs_2CO_3 (0.3 mmol, 1.5 equiv) and phthalic anhydride **6** (0.2 mmol, 1.0 equiv). After replacing the air in it with argon atmosphere, 4.0 mL of dry acetonitrile, which has been degassed with argon, was injected under argon. Then, also under argon, 100 μL of quinuclidine acetonitrile solution (1.0 M, without molecular oxygen) and olefin **1** (1.0 mmol, 5 equiv) were injected with micro-injectors, respectively. After all the materials were added, the reaction tube was sealed with a Teflon plug under argon. Then, the reaction tube was

placed under the blue light of an 8 W blue LED strip at room temperature (using a fan to maintain the temperature). After stirring for 36 hours under these conditions, the reaction solution was adjusted to pH ~ 1 with the addition of HCl (2.0 M) aqueous solution and extracted with DCM. The organic phase was concentrated to 5.0 mL and then 2.0 mL methanol was added. Then 0.6 mmol $TMSCHN_2$ (2.0 M in *n*-hexane) was added dropwise at room temperature. The resulting reaction solution was stirred for another 1.0 hour at room temperature and concentrated directly on a rotary evaporator. The corresponding residue was purified directly by a column chromatography (SiO_2) using ethyl acetate/petroleum ether as the eluent to give the corresponding product **7**.

(E) Ring Expansion Reaction

In the course of examining substrate scope, an interesting result was obtained in the reaction of **2a** with **1d**, as shown in Scheme S1. Lengthening the reaction time, the yield of **3da** decreased, along with increasing the yield of a ring-expanded product **4**. Similarly, by extending the reaction time, the reaction of **2a** and **1g** can produce the ring-expanded product **S4** (Scheme S2). For these ring expansion reactions, control experiments and a proposed reaction process have been given in Scheme S3 and Scheme S4, respectively. Product **4** was obtained through the ring-opening intermediate **S3da** and the subsequent intra-aza-Michael addition process.

Scheme S1. Ring-expanded Product 4 Formed in the Reaction

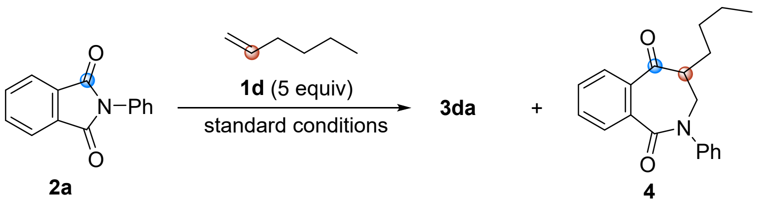

time	yield of **3da**	yield of **4**
36 h	63%	<10%
48 h	61%	18%
72 h	29%	45%
108 h	16%	56%

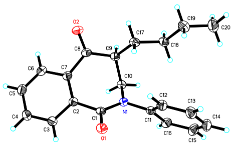

4, X-ray

Scheme S2. Ring-expanded Product S4 Formed in the Reaction

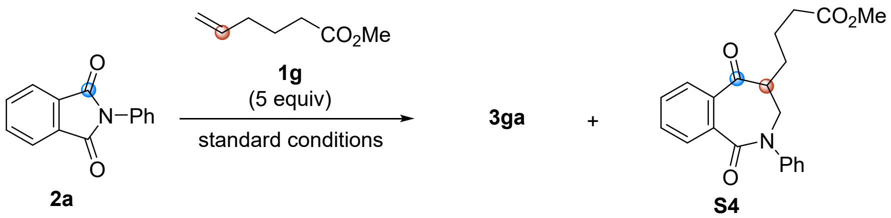

36 h	32% yield	trace
72 h	29% yield	20% yield

Scheme S3. Control Experiments of Ring-expanded Reaction

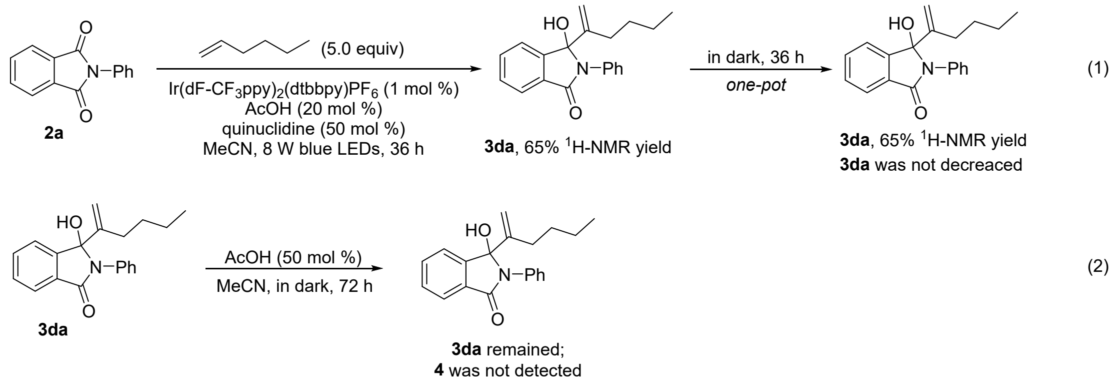

Procedure for Scheme S3, Eq. 1: A 5 mL dry sealed tube equipped with a Teflon plug and a magnetic stirrer bar was charged with Ir[dF(CF_3)ppy]$_2$(dtbbpy)PF_6 (0.002 mmol, 0.01 equiv) and phthalimide **2a** (0.2 mmol, 1.0 equiv). After replacing the air in it with argon atmosphere, 4.0 mL of dry acetonitrile, which has been degassed with argon, was injected under argon. Then, also under argon, 100 μL of quinuclidine acetonitrile solution (1.0 M, without molecular oxygen), 2.3 μL of glacial acetic acid (0.04 mmol, 0.2 equiv) and olefin **1d** (1.0 mmol, 5 equiv) were injected with micro-injectors, respectively. After all the materials were added, the reaction tube was sealed with a Teflon plug under argon. Then, the reaction tube was placed under the blue light of an 8 W blue LED strip at room temperature (using a fan to maintain the temperature). After stirring for 36 hours under these conditions, 1.0 mL of reaction solution was taken out under argon atmosphere to determine the yield of **3da** with 1,3,5-trimethoxybenzene as an internal standard by ^{1}H-NMR spectroscopy and the rest of reaction solution was stirred in dark for another 36 hours at room temperature and another 1 mL of reaction solution was taken out to determine the yield of **3da** with 1,3,5-trimethoxybenzene as an internal standard by ^{1}H-NMR spectroscopy.

Scheme S4. Proposed Ring Expansion Reaction Mechanism

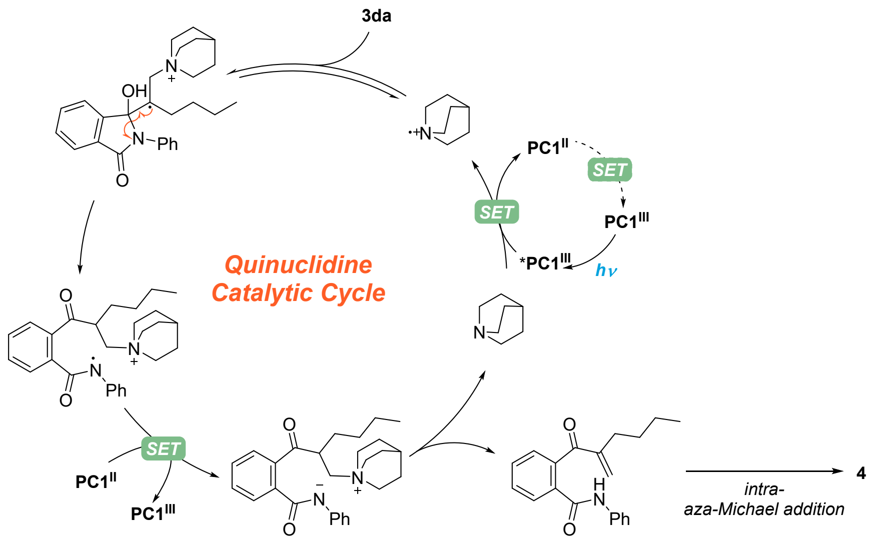

(F) Characterization Data

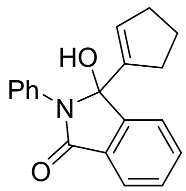

3-(cyclopent-1-en-1-yl)-3-hydroxy-2-phenylisoindolin-1-one (3aa). A white solid, 44 mg, 76% yield; M.p.: 162-163 °C; ^{1}H NMR ($CDCl_3$, 400 MHz, TMS) δ 7.62-7.55 (m, 2H), 7.55-7.50 (m, 2H), 7.44-7.39 (m, 1H), 7.35-7.30 (m, 1H), 7.30-7.23 (m, 2H), 7.21-7.14 (m, 1H), 6.20-6.12 (m, 1H), 4.25 (s, 1H), 2.38-2.18 (m, 2H), 1.93-1.83 (m, 1H), 1.73-1.63 (m, 2H), 1.63-1.54 (m, 1H); ^{13}C NMR ($CDCl_3$, 100 MHz, TMS) δ 167.1, 146.4, 141.6, 136.2, 132.8, 131.0, 130.9, 130.3, 129.5, 128.4, 125.8, 124.4, 123.6, 122.0, 92.1, 32.5, 31.1, 23.4; IR (neat) ν 3301, 2964, 2910,2852, 1676, 1601, 1497, 1463, 1359, 1200, 1042, 868, 756 cm^{-1}; HRMS (ESI) Calcd. for $C_{19}H_{17}NO_2Na^+$ Requires: 314.1152, Found: 314.1147.

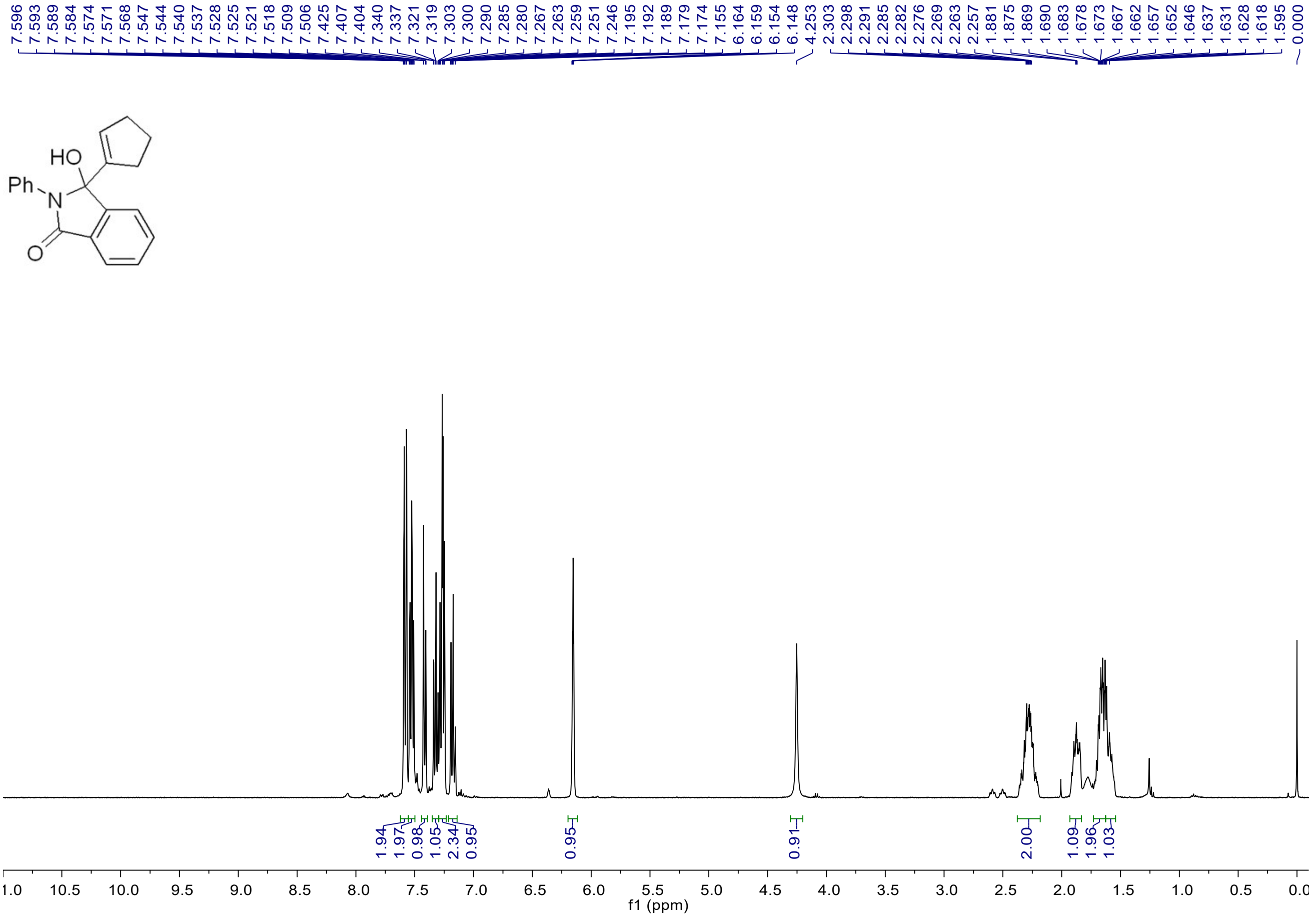

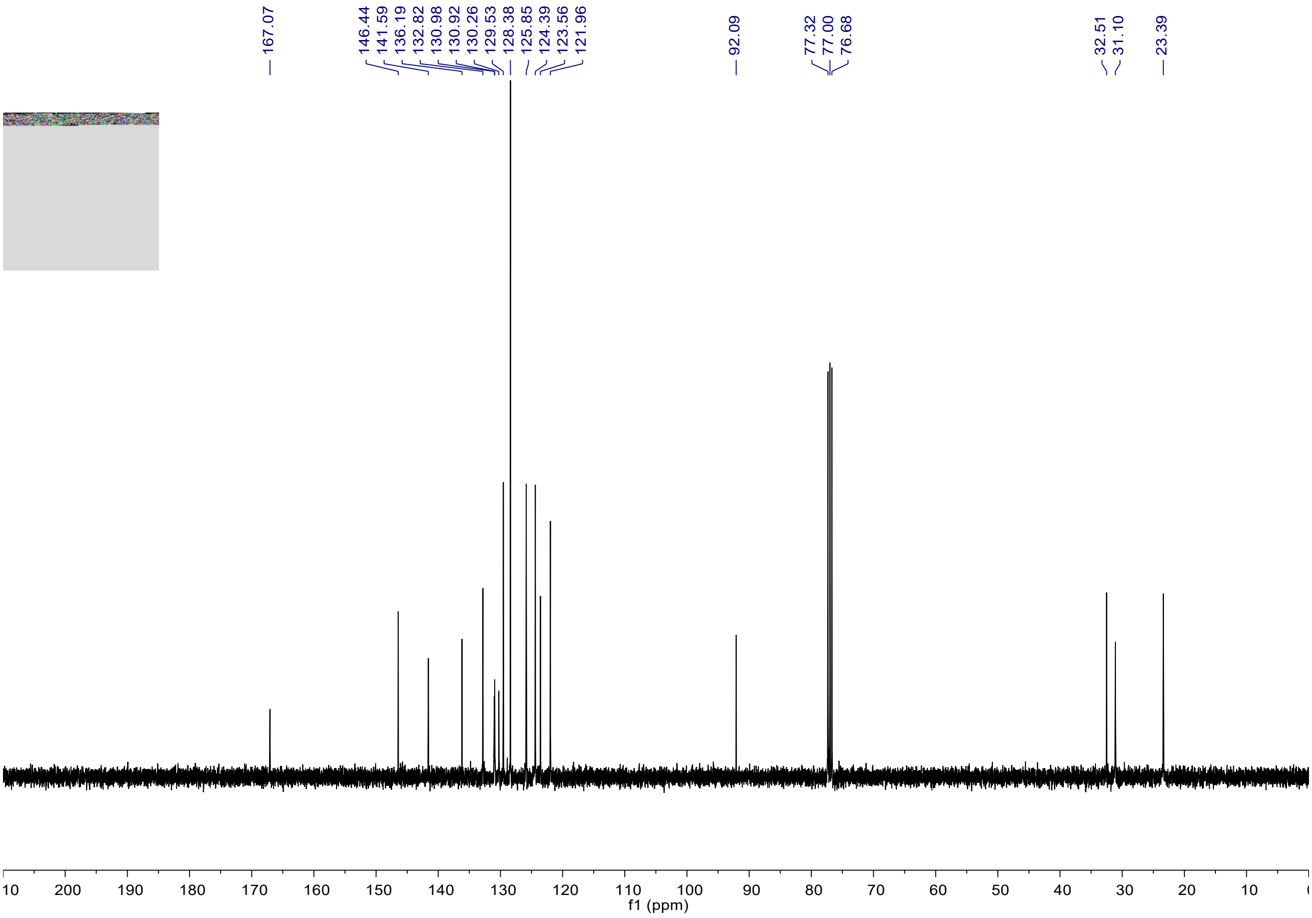

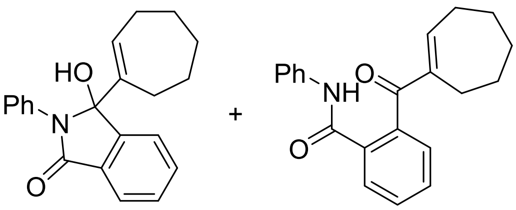

minor

3-(cyclohept-1-en-1-yl)-3-hydroxy-2-phenylisoindolin-1-one (3ba). A white solid, 61 mg, 95% yield; M.p.: 156-157 °C; ^{1}H NMR (CD_2Cl_2, 400 MHz, TMS) δ 7.93 (s, 1H), 7.59-7.52 (m, 1H), 7.51-7.36 (m, 16H), 7.34-7.23 (m, 8H), 7.23-7.14 (m, 7H), 7.14-6.98 (m, 4H), 6.56 (t, J = 6.8 Hz, 3H), 6.43 (t, J = 6.6 Hz, 1H), 4.11 (s, 2H), 2.47-2.40 (m, 2H), 2.17-1.93 (m, 8H), 1.66-1.54 (m, 4H), 1.54-1.37 (m, 10H), 1.37-1.31 (m, 6H), 1.31-1.12 (m, 7H), 0.90-0.71 (m, 6H); ^{13}C NMR (CD_2Cl_2, 100 MHz, TMS) δ 199.1, 167.5, 166.1, 150.4, 146.8, 146.6, 139.8, 139.4, 138.1, 136.5, 136.0, 132.9, 131.9, 131.1, 130.4, 129.8, 129.7, 129.0, 128.6, 128.3, 127.3, 126.0, 125.0, 124.5, 123.4, 122.3, 119.8, 94.2, 32.3, 32.0, 29.4, 28.4, 28.3, 26.5, 26.3, 26.2, 25.9, 25.8; IR (neat) ν 3326, 2956, 2922,

2845, 1679, 1592, 1494, 1401, 1359, 1205, 1139, 1099, 1042, 871, 840, 755, 693 cm^{-1}; HRMS (ESI) Calcd. for $C_{21}H_{21}NO_2Na^+$ Requires: 342.1465, Found: 342.1463.

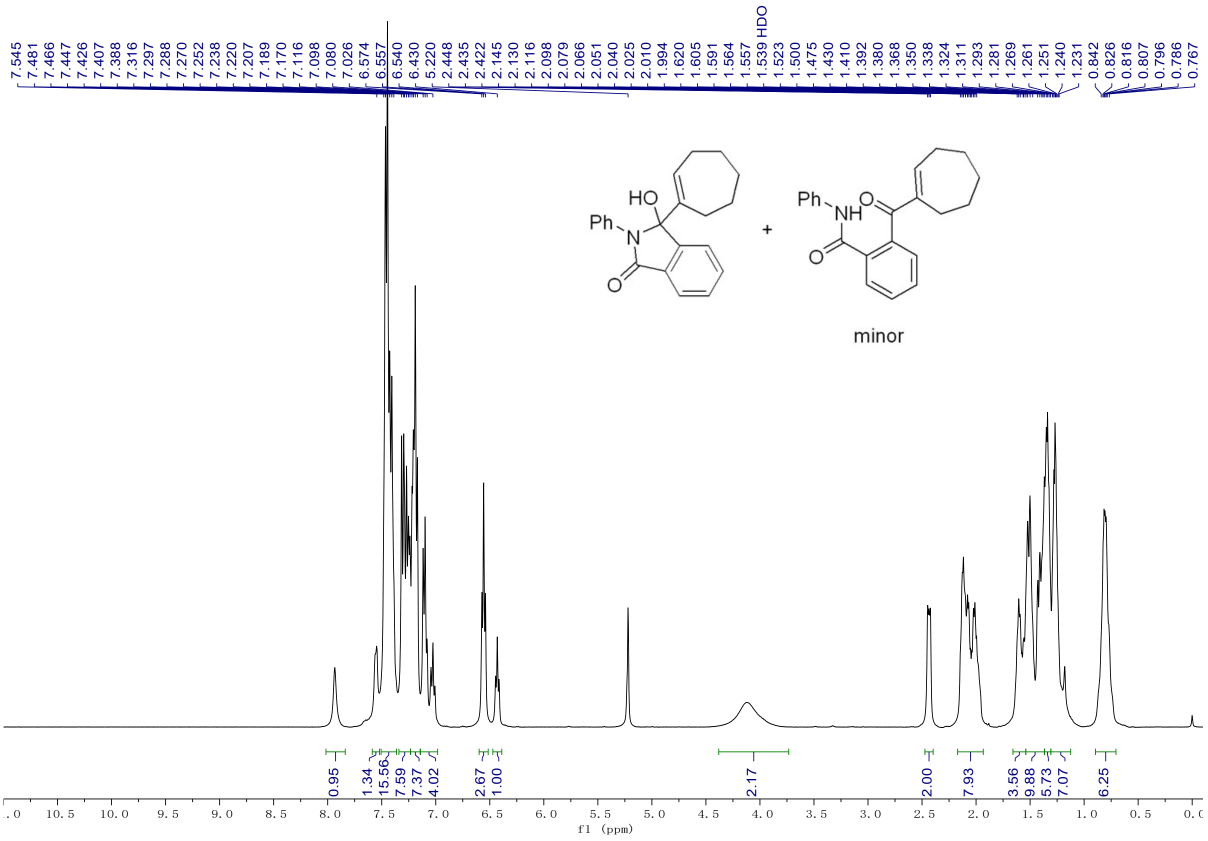

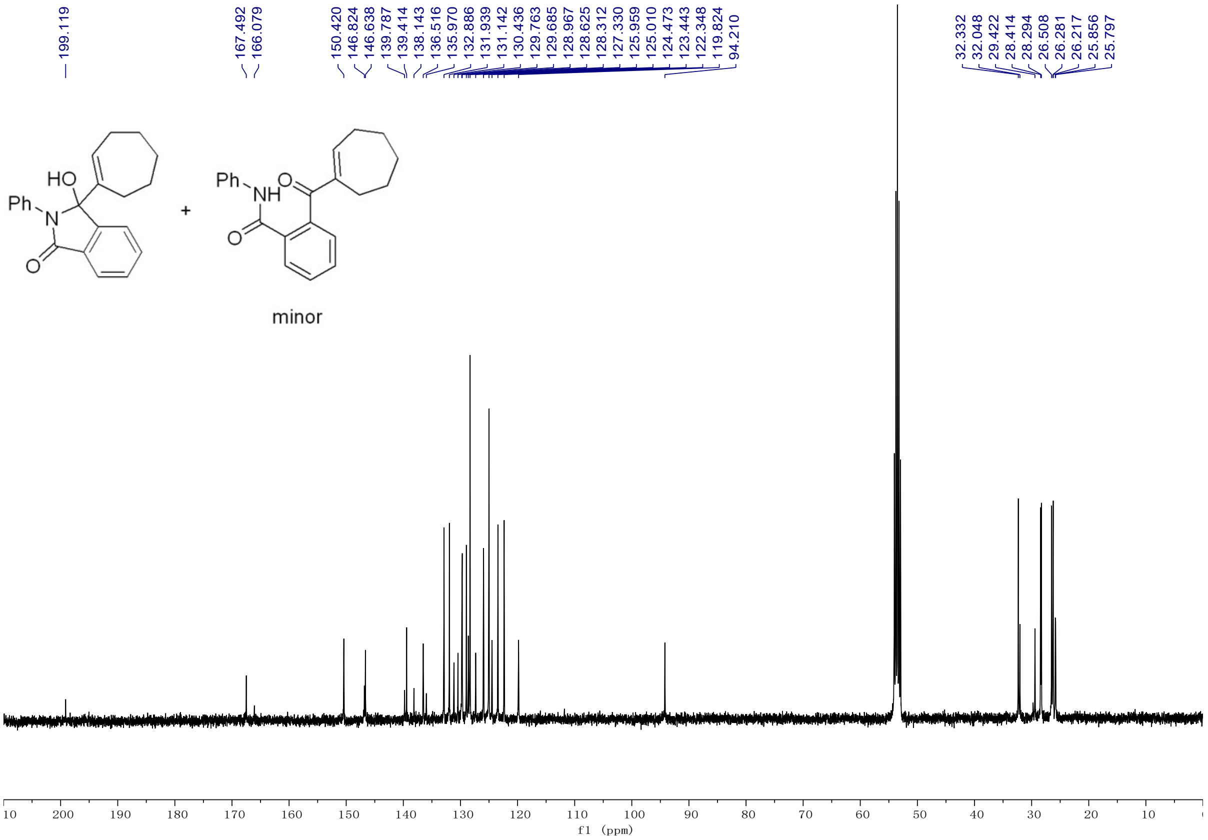

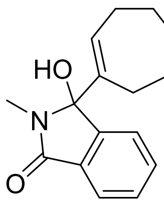

3-(cyclohept-1-en-1-yl)-3-hydroxy-2-methylisoindolin-1-one (3bb). A white solid, 50 mg, 98% yield; M.p.: 145-146 °C; ^{1}H NMR (CD_2Cl_2, 400 MHz, TMS) δ 7.54-7.47 (m, 1H), 7.42-7.35 (m, 1H), 7.35-7.27 (m, 2H), 6.73-6.64 (m, 1H), 4.52 (s, 1H), 2.56 (s, 3H), 2.31-2.20 (m, 2H), 1.74-1.51 (m, 4H), 1.51-1.40 (m, 1H), 1.40-1.29 (m, 1H), 1.25-1.12 (m, 1H), 1.07-0.93 (m, 1H); ^{13}C NMR (CD_2Cl_2, 100 MHz, TMS) δ 167.9, 147.2, 138.8, 132.1, 131.7, 131.5, 129.2, 122.6, 122.3, 91.8, 32.5, 28.4, 27.7, 26.8, 26.6, 23.7; IR (neat) ν 3161, 2921, 2845, 1674, 1614, 1477, 1388, 1338, 1286, 1240, 1169, 1048, 1024, 878 cm^{-1}; HRMS (ESI) Calcd. for $C_{16}H_{19}NO_2Na^+$ Requires: 280.1308, Found: 280.1304.

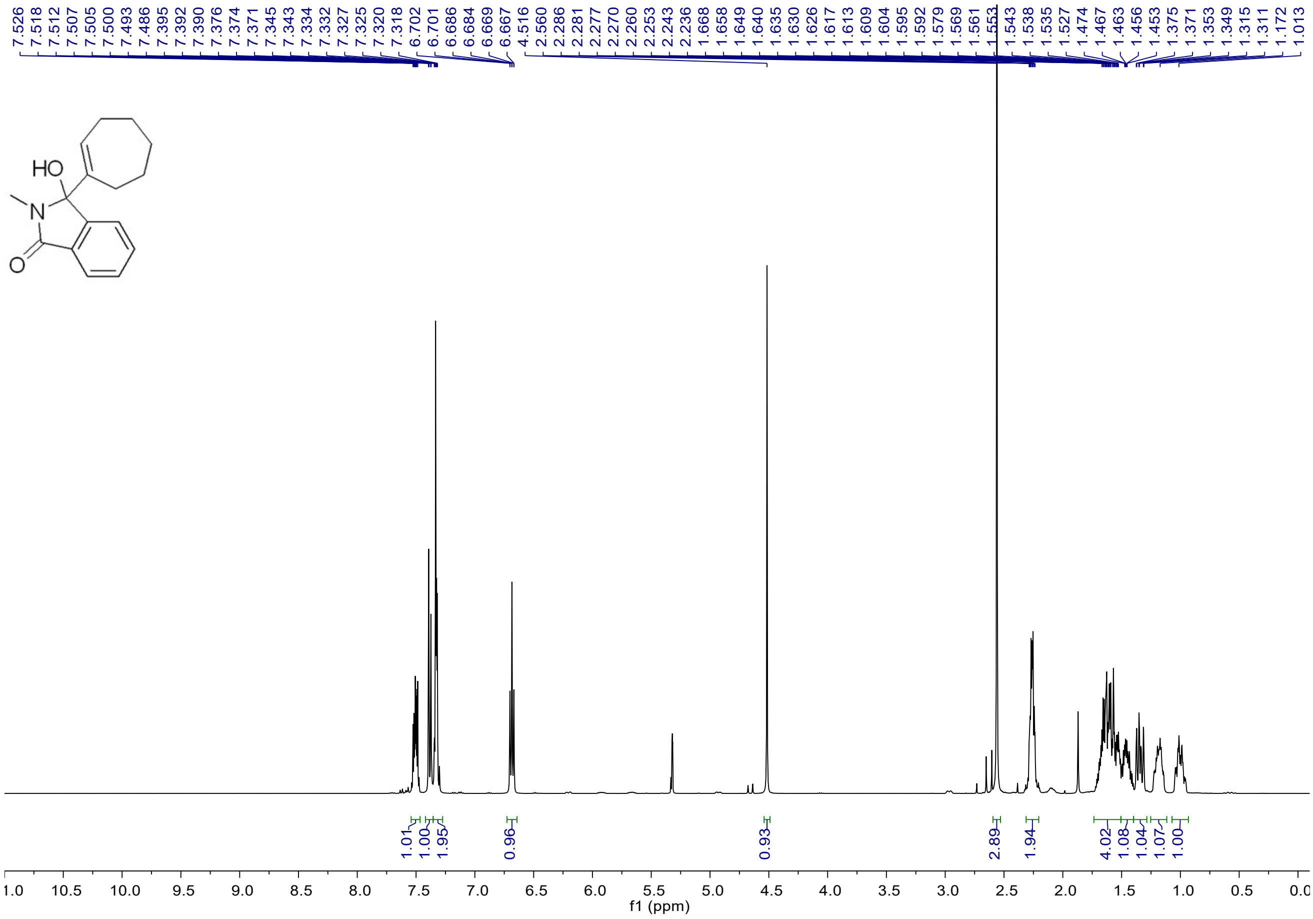

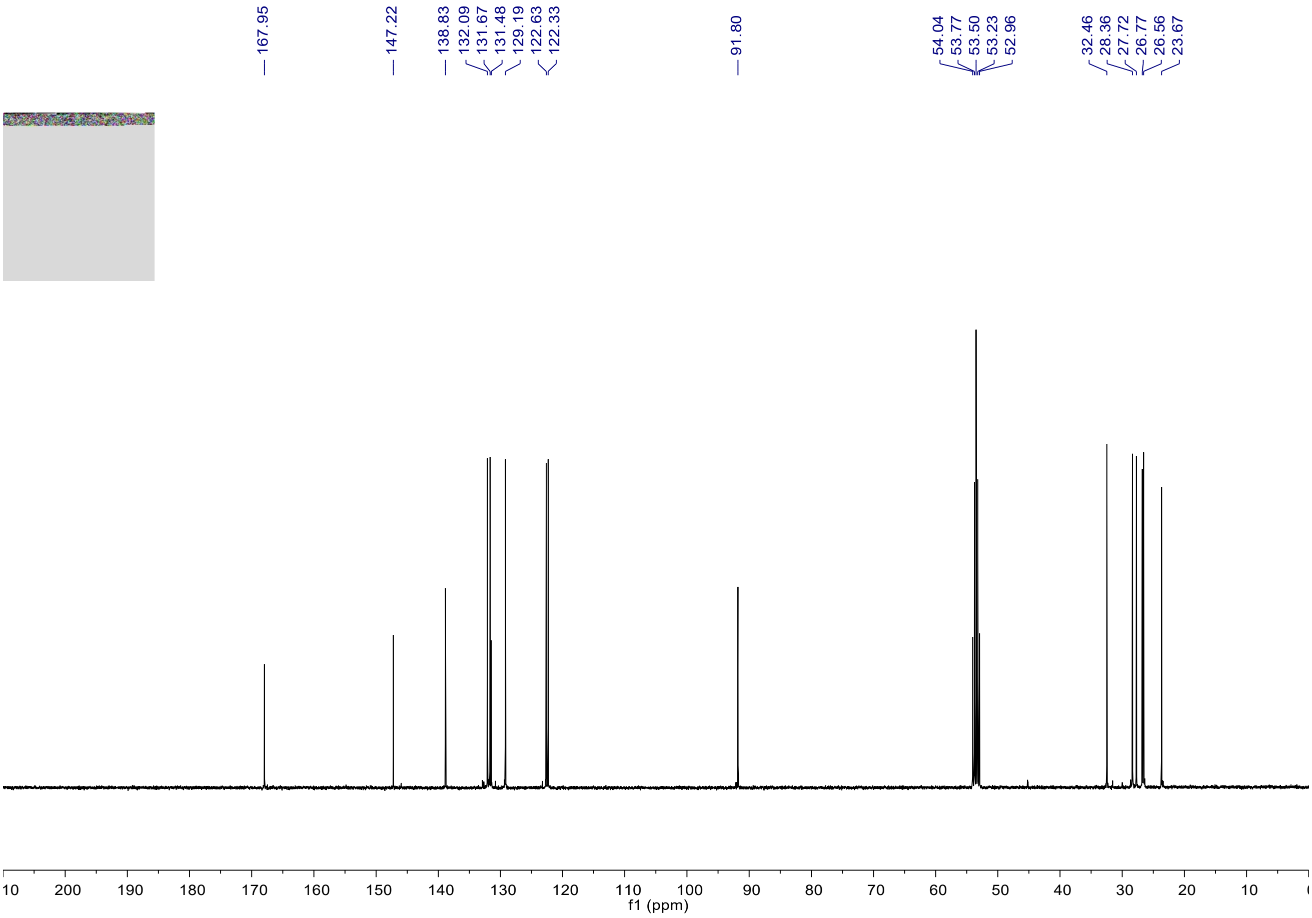

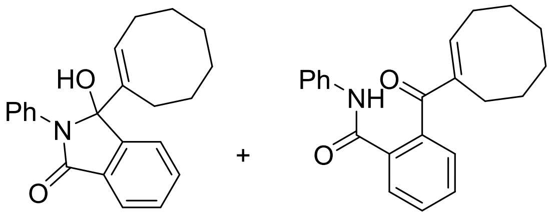

(E)-3-(cyclooct-1-en-1-yl)-3-hydroxy-2-phenylisoindolin-1-one (3ca). A white solid, 53 mg, 80% yield; M.p.: 152-153 °C; ^{1}H NMR (CD_2Cl_2, 400 MHz, TMS) δ 7.96 (s, 1H), 7.63-7.56 (m, 1H), 7.54-7.46 (m, 4H), 7.46-7.29 (m, 6H), 7.21 (t, J = 7.7 Hz, 5H), 7.11 (t, J = 7.4 Hz, 1H), 7.02 (t, J = 7.4 Hz, 1H), 6.31 (t, J = 8.4 Hz, 2H), 3.89 (s, 1H), 2.47-2.39 (m, 2H), 2.22-2.12 (m, 2H), 2.07-1.97 (m, 2H), 1.88-1.75 (m, 1H), 1.69-1.58 (m, 1H), 1.55-1.35 (m, 6H), 1.35-1.23 (m, 5H), 1.23-1.10 (m, 2H), 1.10-1.04 (m, 1H), 1.04-0.95 (m, 1H), 0.46-0.31 (m, 1H); ^{13}C NMR (CD_2Cl_2, 100 MHz, TMS) δ 199.0, 167.3, 165.9, 149.83, 149.80, 146.9, 143.5, 139.8, 138.1, 136.4, 136.1, 135.5, 132.6, 130.8, 130.3, 130.21, 130.18, 129.7, 129.5, 128.9, 128.4, 128.2, 127.6, 126.0, 125.1, 124.4, 123.4, 122.7, 119.8, 94.14, 29.08, 29.1, 28.92, 28.87, 27.6, 26.3, 26.2, 26.1, 26.0, 25.9, 25.5, 23.7; IR (neat) ν

3288, 2927, 2846, 1675, 1593, 1494, 1363, 1228, 1204, 1228, 1137, 1114, 1051, 960 cm^{-1}; HRMS (ESI) Calcd. for $C_{22}H_{23}NO_2Na^+$ Requires: 356.1621, Found: 356.1621.

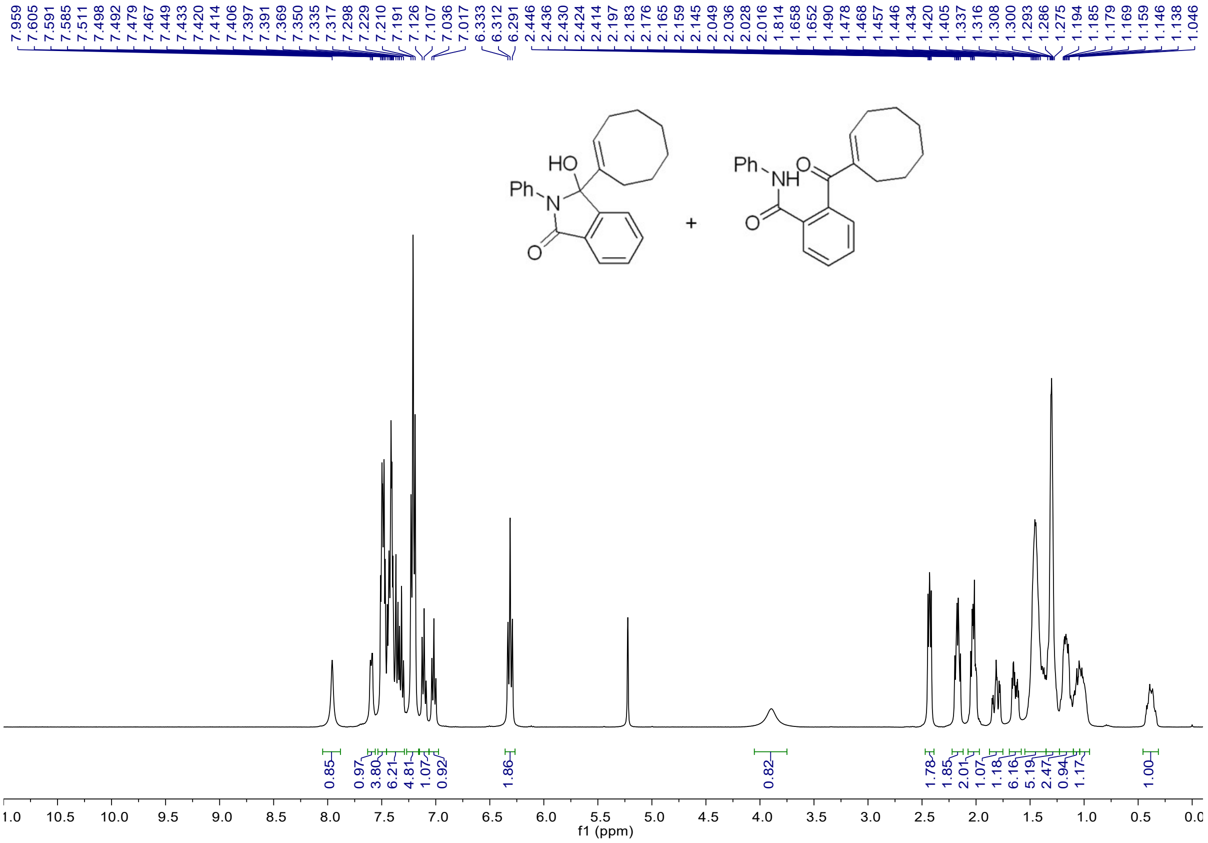

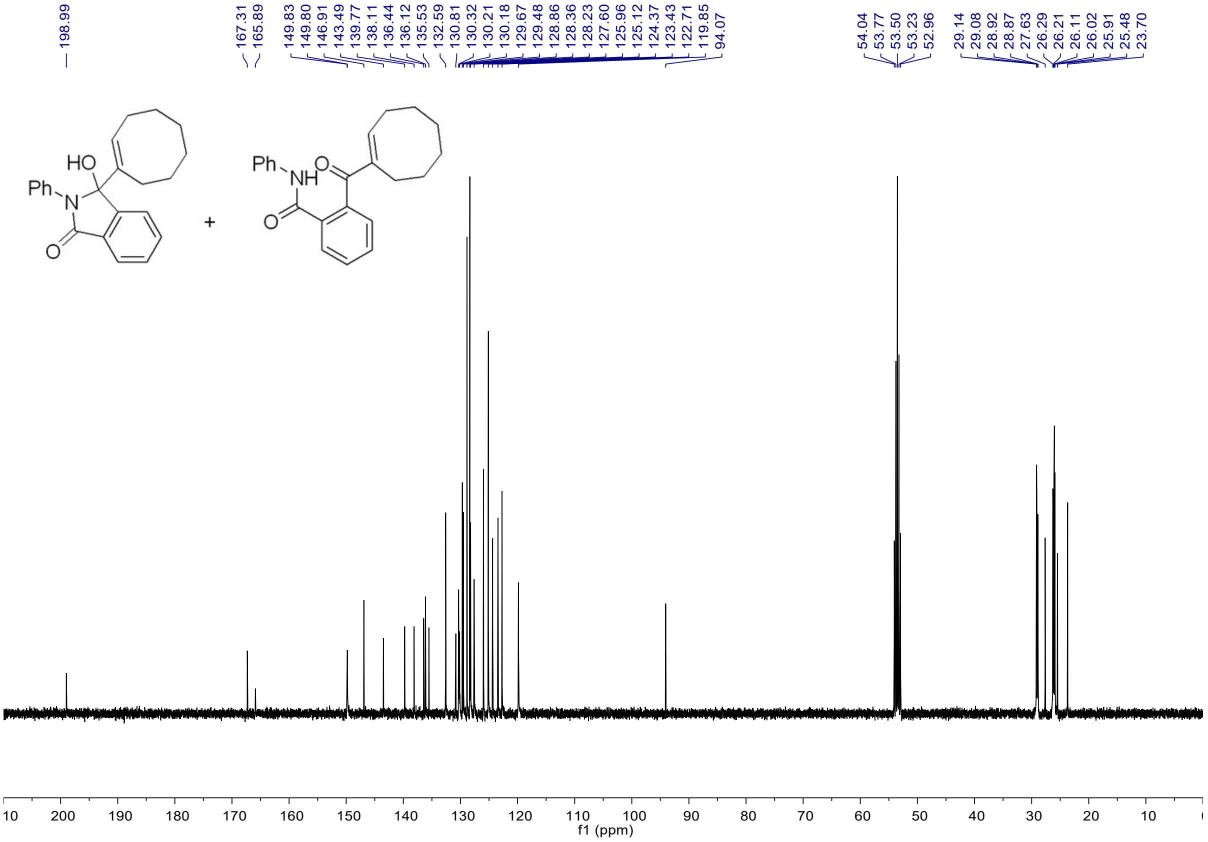

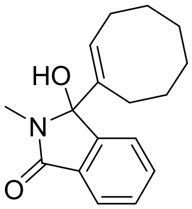

(E)-3-(cyclooct-1-en-1-yl)-3-hydroxy-2-methylisoindolin-1-one (3cb). A white solid, 47 mg, 87% yield; M.p.: 152-153 °C; ^{1}H NMR (CD_2Cl_2, 400 MHz, TMS) δ 7.56-7.47 (m, 1H), 7.47-7.41 (m, 1H), 7.41-7.30 (m, 2H), 6.45 (t, J = 8.4 Hz, 1H), 4.49 (s, 1H), 2.56 (s, 3H), 2.29-2.19 (m, 2H), 1.98-1.88 (m, 1H), 1.71-1.60 (m, 1H), 1.60-1.41 (m, 4H), 1.40-1.26 (m, 2H), 1.21-1.10 (m, 1H), 0.60-0.46 (m, 1H); ^{13}C NMR (CD_2Cl_2, 100 MHz, TMS) δ 167.8, 147.5, 135.5, 131.8, 131.2, 129.8, 129.2, 122.7, 91.7, 29.22, 29.16, 26.3, 26.2, 26.1, 24.7, 23.7; IR (neat) ν 3272, 2928, 2853, 1667, 1613, 1424, 1231, 1180, 1084, 1030, 946, 828, 762 cm^{-1}; HRMS (ESI) Calcd. for $C_{17}H_{21}NO_2Na^+$ Requires: 294.1465, Found: 294.1463.

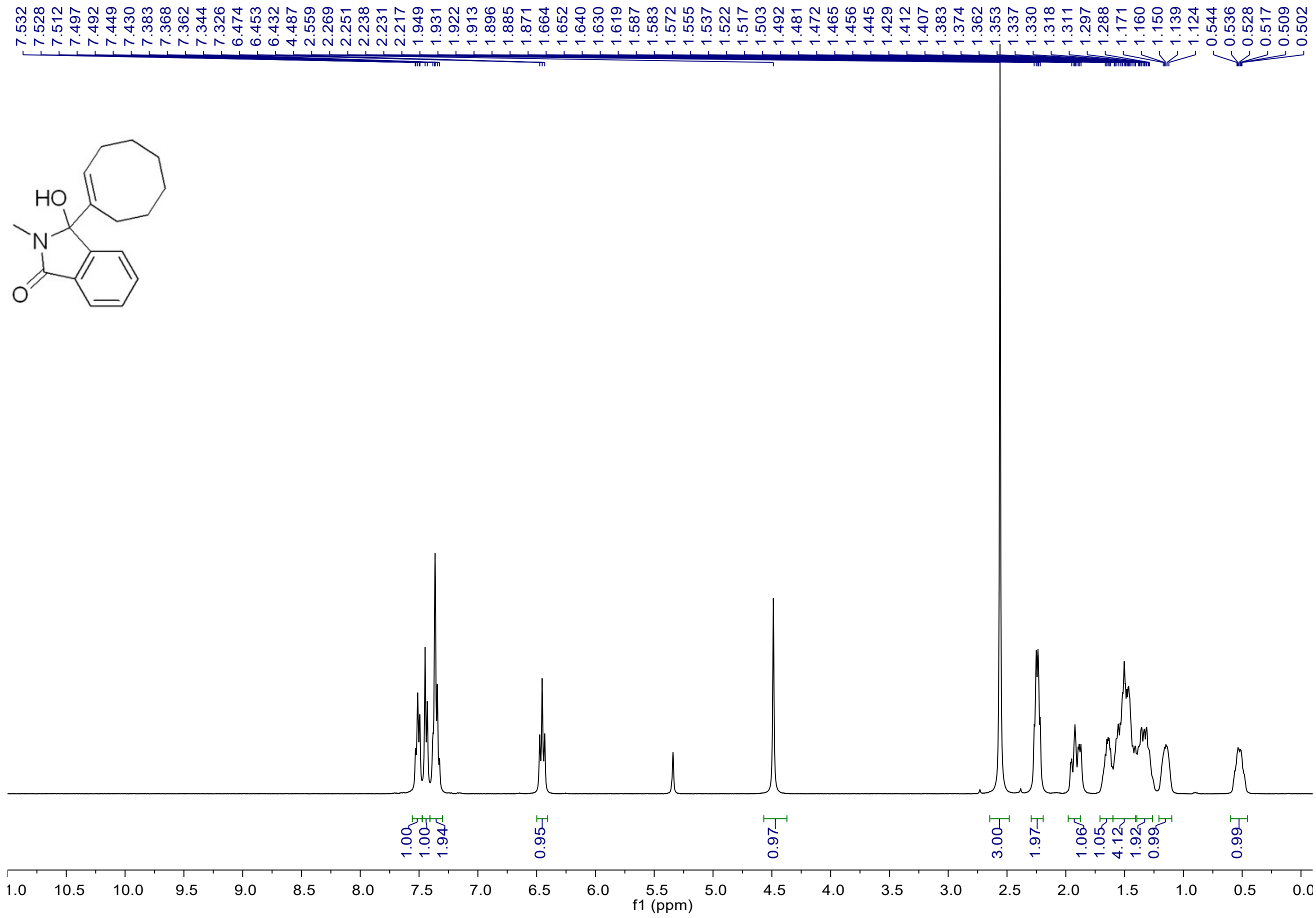

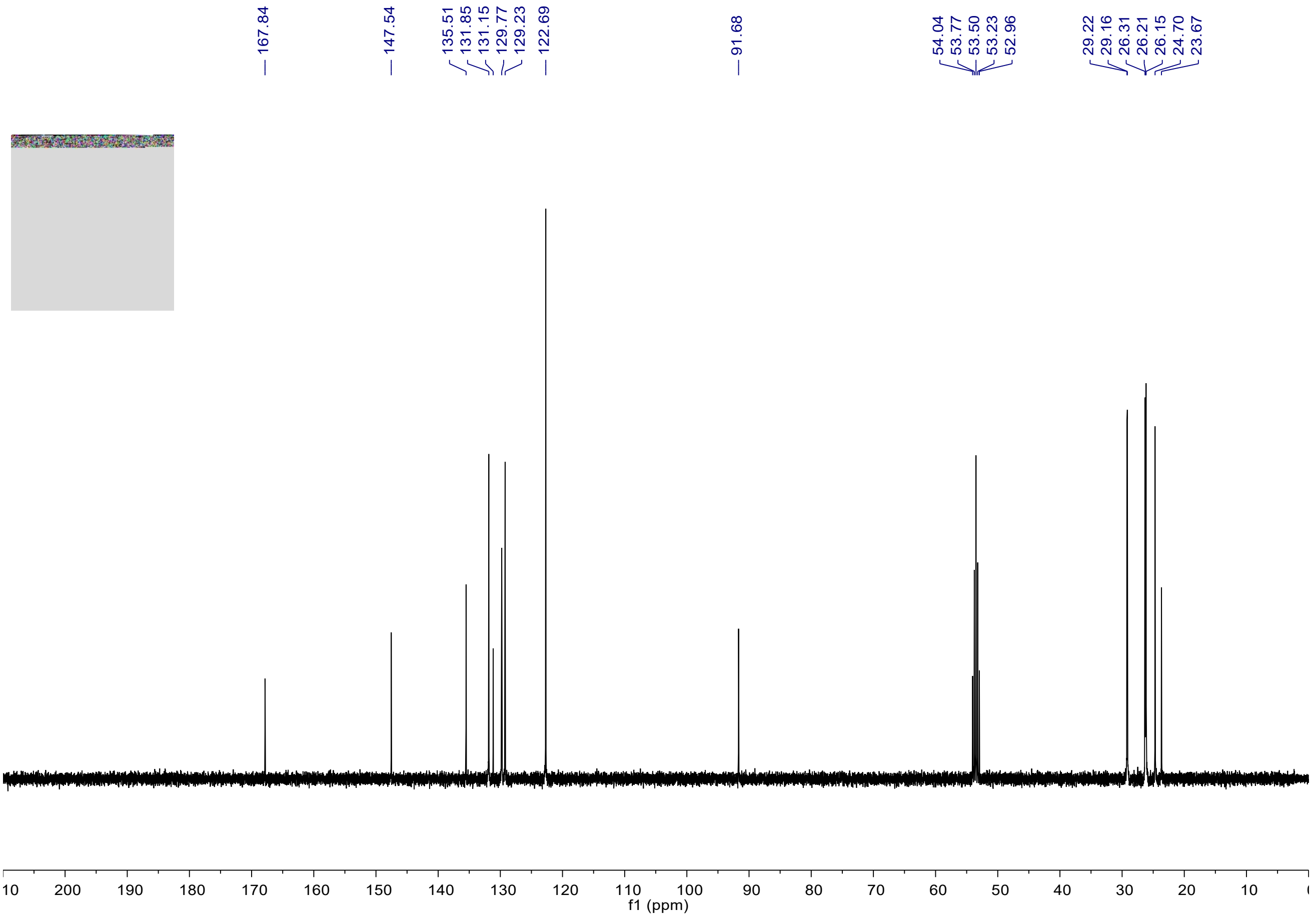

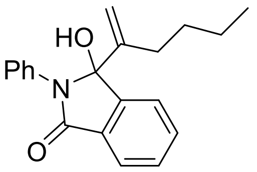

3-(hex-1-en-2-yl)-3-hydroxy-2-phenylisoindolin-1-one (3da). A white solid, 39 mg, 63% yield; M.p.: 130-131 °C; ^{1}H NMR (CD_2Cl_2, 400 MHz, TMS) δ 7.53-7.41 (m, 3H), 7.37 (d, J = 7.5 Hz, 1H), 7.30 (d, J = 7.5 Hz, 1H), 7.24 (t, J = 7.5 Hz, 2H), 7.17 (t, J = 7.8 Hz, 2H), 7.08 (t, J = 7.3 Hz, 1H), 5.75 (s, 1H), 5.12-5.06 (m, 1H), 4.31 (s, 1H), 1.54-1.41 (m, 1H), 1.27-1.15 (m, 1H), 1.06-0.93 (m, 2H), 0.96-0.82 (m, 2H), 0.55 (t, J = 7.2 Hz, 3H); ^{13}C NMR (CD_2Cl_2, 100 MHz, TMS) δ 167.5, 146.8, 145.6, 136.4, 133.0, 130.6, 129.8, 128.4, 125.8, 124.3, 123.5, 122.1, 113.9, 93.8, 29.6, 29.4, 22.2, 13.6; IR (neat) ν 3246, 2951, 2926, 1677, 1606, 1497, 1456, 1363, 1334, 1234, 1141, 1102, 1047, 933, 874, 769 cm^{-1}; HRMS (ESI) Calcd. for $C_{20}H_{21}NO_2Na^+$ Requires: 330.1465, Found: 330.1463.

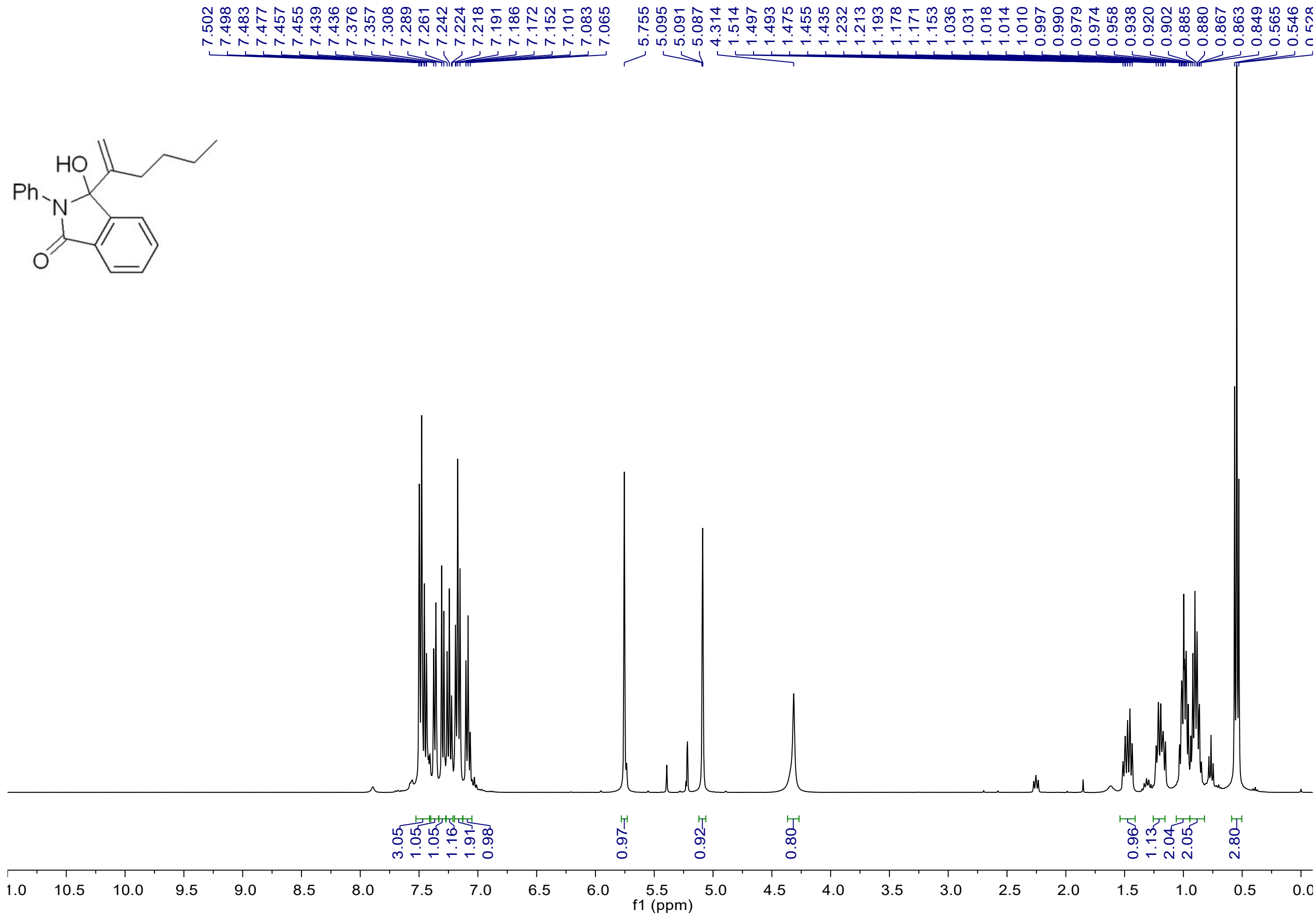

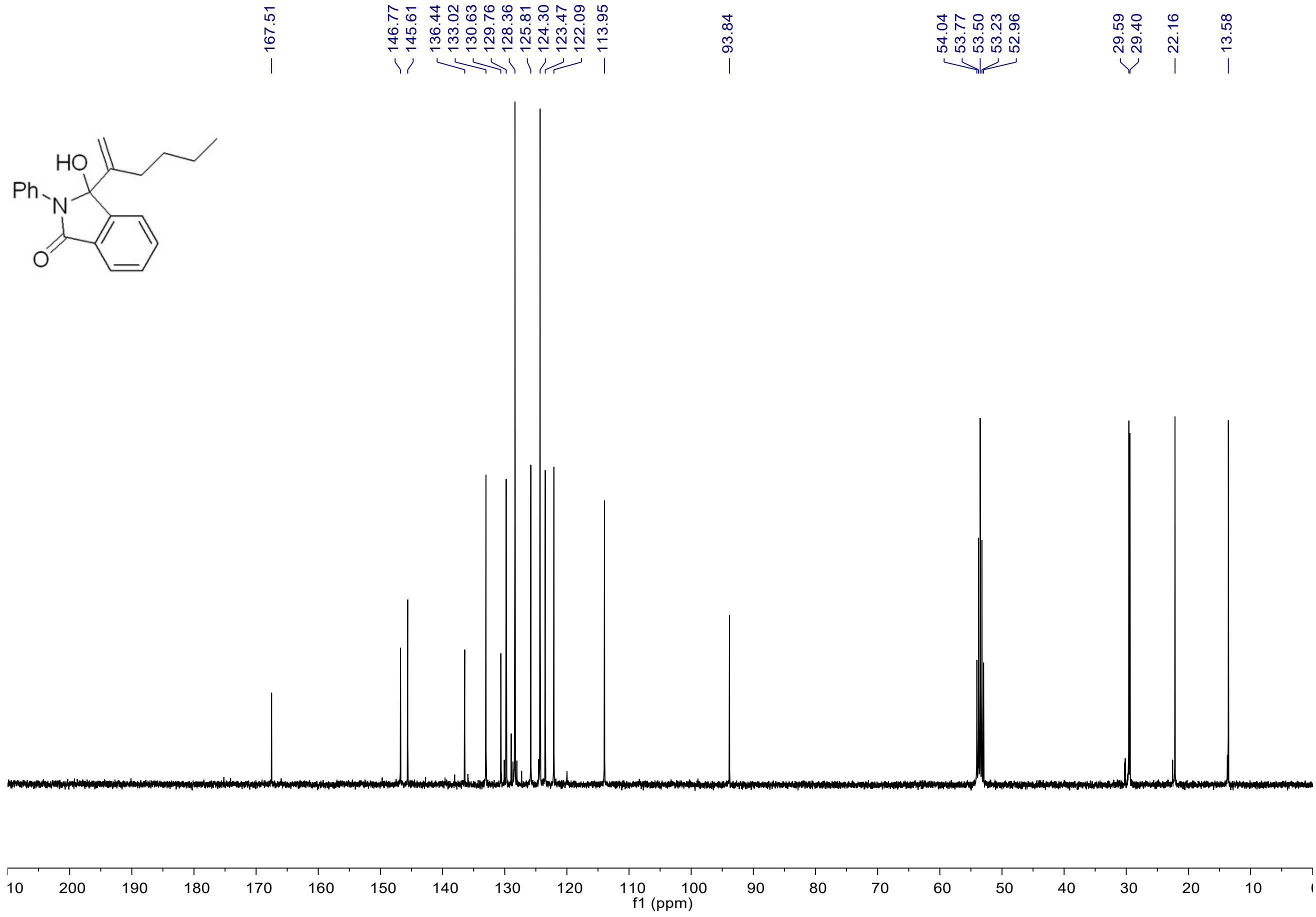

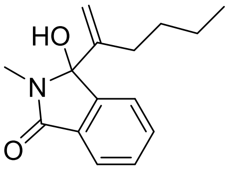

3-(hex-1-en-2-yl)-3-hydroxy-2-methylisoindolin-1-one (3db). A colorless oil, 19 mg, 39% yield; ^{1}H NMR (CD_2Cl_2, 400 MHz, TMS) δ 7.44 (td, *J* = 7.4, 1.4 Hz, 1H), 7.38-7.24 (m, 3H), 5.76 (t, *J* = 1.3 Hz, 1H), 5.22-5.16 (m, 1H), 4.10 (s, 1H), 2.54 (s, 3H), 1.48-1.36 (m, 1H), 1.23-1.11 (m, 3H), 1.06-0.93 (m, 2H), 0.63 (t, *J* = 7.2 Hz, 3H); ^{13}C NMR (CD_2Cl_2, 100 MHz, TMS) δ 167.7, 147.2, 145.0, 132.3, 131.3, 129.5, 122.8, 122.2, 113.7, 91.3, 29.9, 29.3, 23.5, 22.3, 13.6; IR (neat) ν 3298, 2952, 2853, 1688, 1614, 1466, 1379, 1241, 1116, 1033, 947, 909, 848, 724 cm^{-1}; HRMS (ESI) Calcd. for $C_{15}H_{19}NO_2Na^+$ Requires: 268.1308, Found: 268.1308.

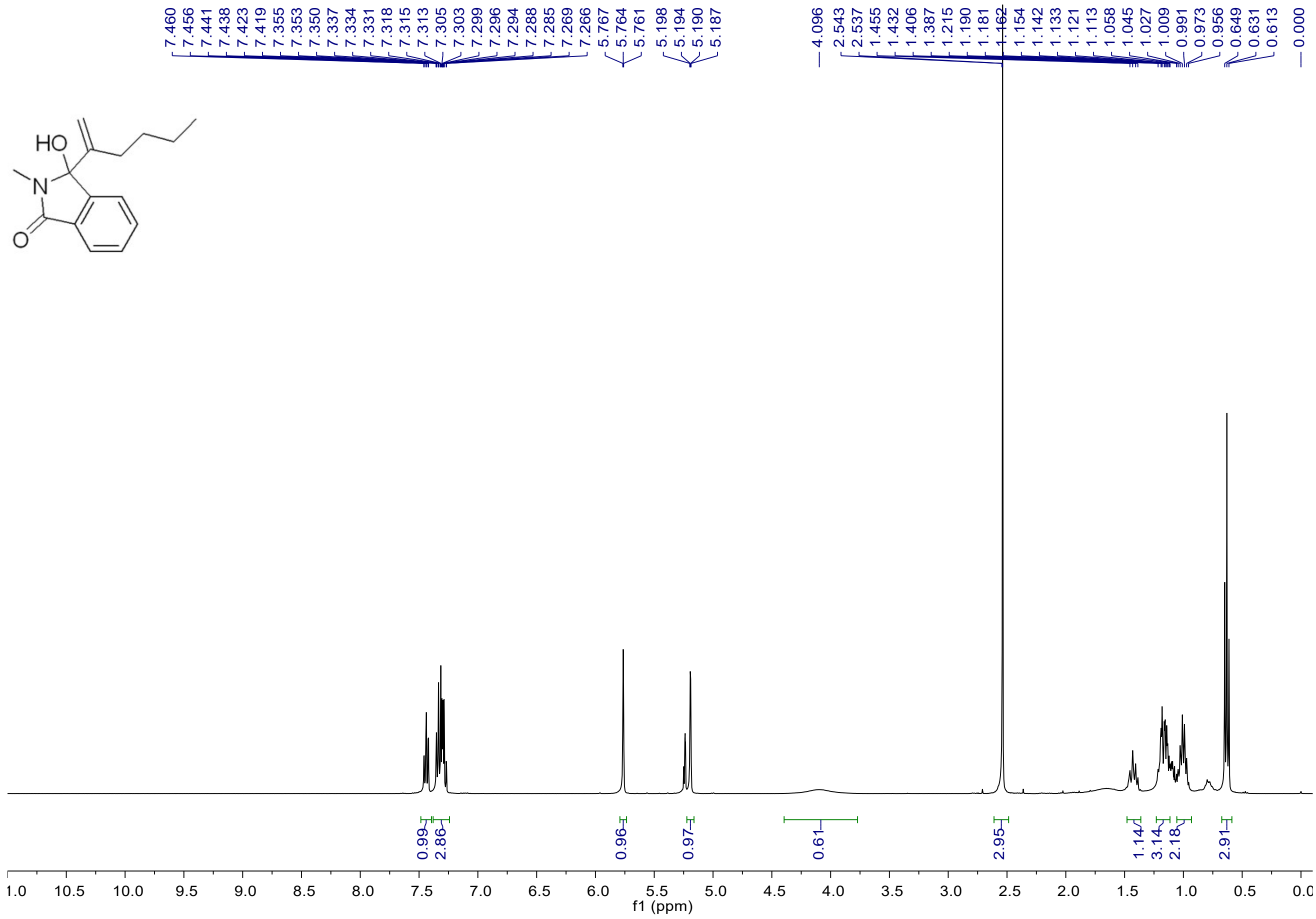

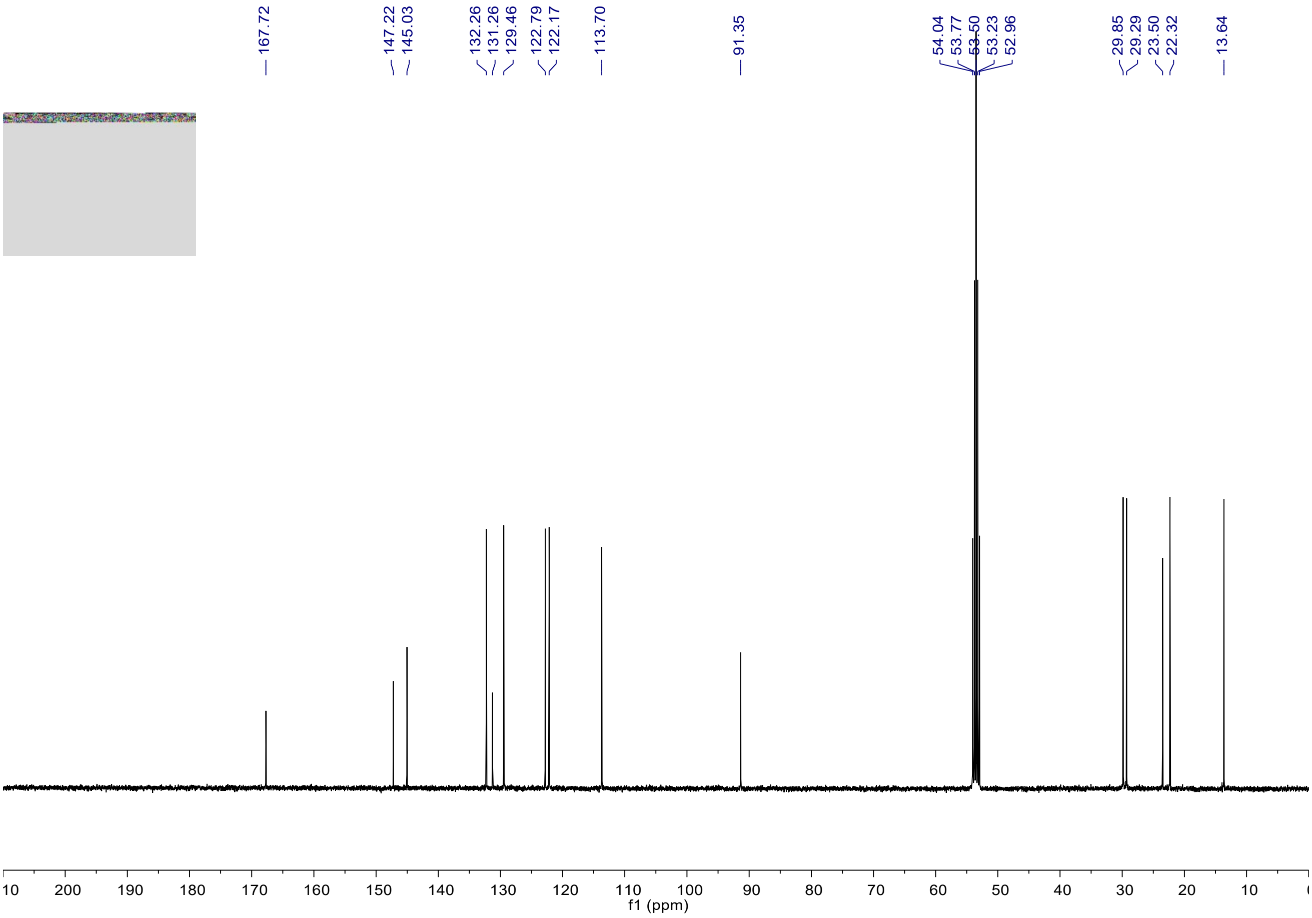

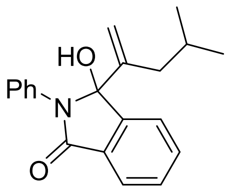

3-hydroxy-3-(4-methylpent-1-en-2-yl)-2-phenylisoindolin-1-one (3ea). A white solid, 33 mg, 54% yield; M.p.: 154-155 °C; ^{1}H NMR (CD_2Cl_2, 400 MHz, TMS) δ 7.58-7.51 (m, 2H), 7.48-7.40 (m, 2H), 7.33-7.24 (m, 2H), 7.21-7.14 (m, 2H), 7.11-7.04 (m, 1H), 5.84 (s, 1H), 5.12-5.07 (m, 1H), 4.21 (s, 1H), 1.42-1.28 (m, 2H), 1.16-1.04 (m, 1H), 0.44 (d, J = 6.0 Hz, 6H); ^{13}C NMR (CD_2Cl_2, 100 MHz, TMS) δ 167.4, 146.5, 143.9, 136.5, 133.0, 130.6, 129.8, 128.3, 125.7, 124.1, 123.5, 122.2, 115.0, 93.8, 39.3, 25.5, 22.0, 21.9; IR (neat) ν 3296, 2924, 2864, 1674, 1598, 1497, 1423, 1365, 1203, 1161, 1111, 992, 953, 798, 727 cm^{-1}; HRMS (ESI) Calcd. for $C_{20}H_{21}NO_2Na^+$ Requires: 330.1465, Found: 330.1459.

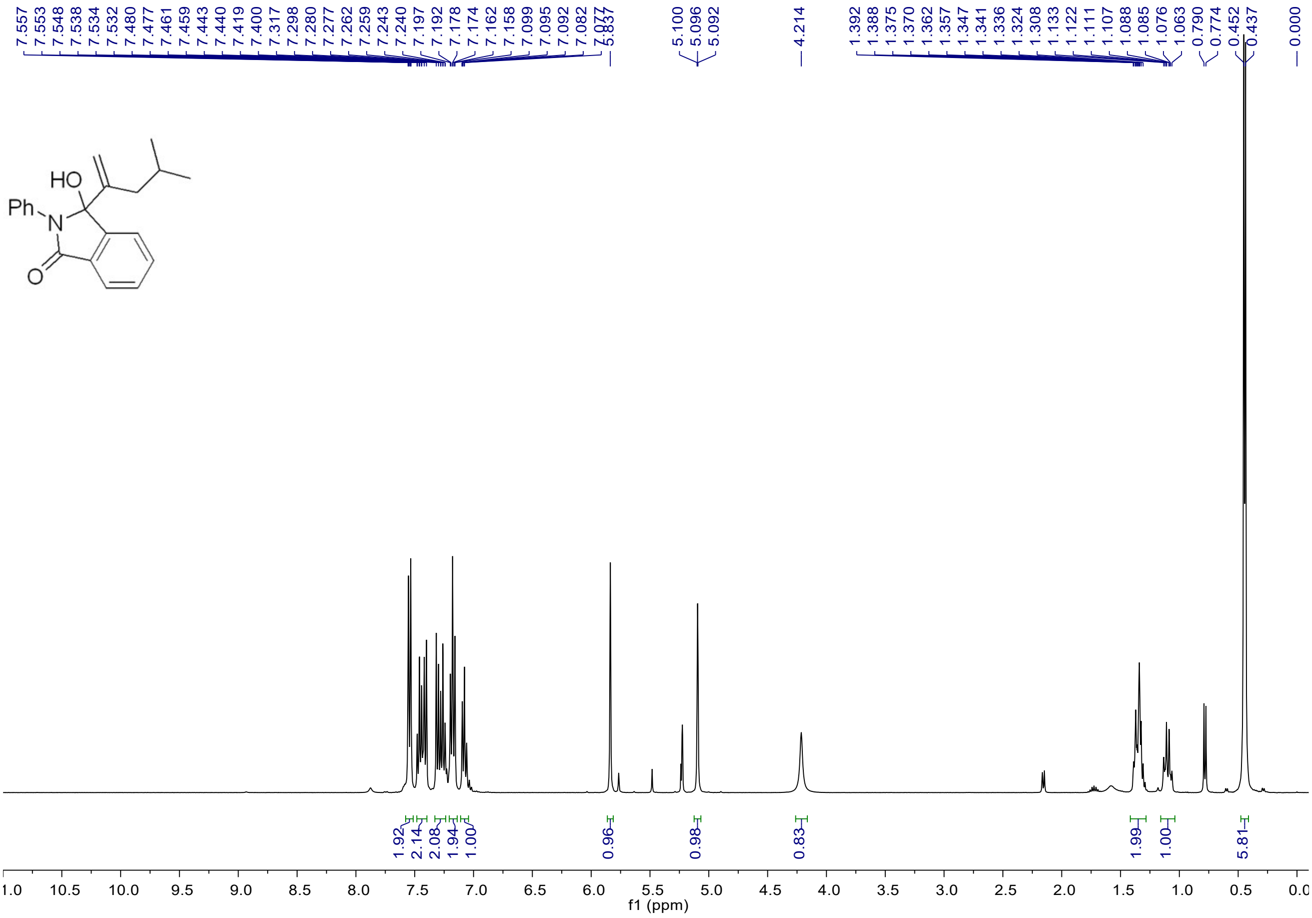
7.557 7.553 7.548 7.538 7.534 7.532 7.480 7.477 7.461 7.459 7.443 7.440 7.419 7.400 7.317 7.298 7.280 7.277 7.262 7.259 7.243 7.240 7.197 7.192 7.178 7.174 7.162 7.158 7.099 7.095 7.092 7.082 7.077 5.837
5.100 5.096 5.092
4.214
1.392 1.388 1.375 1.370 1.362 1.357 1.347 1.341 1.336 1.324 1.308 1.133 1.122 1.111 1.107 1.088 1.085 1.076 1.063 0.790 0.774 0.452 0.437
0.000
HO
Ph
N
O
1.92 2.14 2.08 1.94 1.00 0.96 0.98 0.83 1.99 1.00 5.81
f1 (ppm)

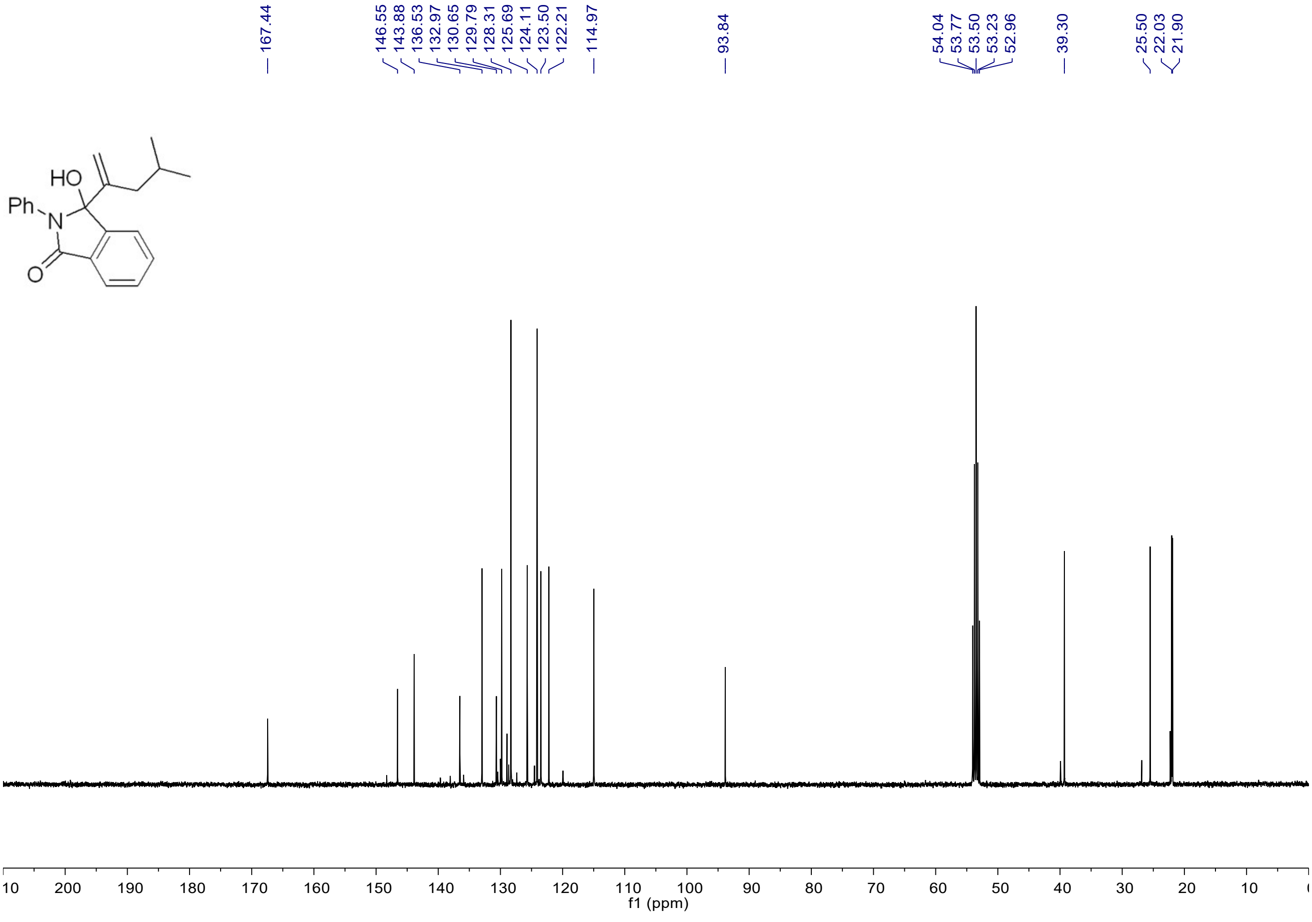
167.44
146.55 143.88 136.53 132.97 130.65 129.79 128.31 125.69 124.11 123.50 122.21
114.97
93.84
54.04 53.77 53.50 53.23 52.96
39.30
25.50 22.03 21.90
HO
Ph
N
O
f1 (ppm)

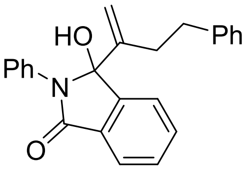

3-hydroxy-2-phenyl-3-(4-phenylbut-1-en-2-yl)isoindolin-1-one (3fa). A white solid, 21 mg, 30% yield; M.p.: 132-133 °C; ^{1}H NMR (CD_2Cl_2, 400 MHz, TMS) δ 7.55-7.45 (m, 4H), 7.34-7.26 (m, 2H), 7.23 (t, *J* = 7.8 Hz, 2H), 7.13 (t, *J* = 7.3 Hz, 1H), 7.06-6.95 (m, 3H), 6.76-6.69 (m, 2H), 5.82 (s, 1H), 5.19 (s, 1H), 3.88 (s, 1H), 2.38-2.28 (m, 2H), 1.90-1.76 (m, 1H), 1.55 (dd, *J* = 16.5, 8.3 Hz, 1H); ^{13}C NMR (CD_2Cl_2, 100 MHz, TMS) δ 167.3, 146.4, 144.9, 141.4, 136.4, 133.2, 130.8, 130.0, 128.5, 128.2, 128.1, 126.1, 125.8, 124.6, 123.6, 122.2, 114.8, 93.6, 33.9, 31.5; IR (neat) ν 3269, 3028, 1679, 1600, 1496, 1454, 1365, 1332, 1229, 1143, 1069, 993, 798, 753 cm^{-1}; HRMS (ESI) Calcd. for $C_{24}H_{21}NO_2Na^+$ Requires: 378.1465, Found: 378.1465.

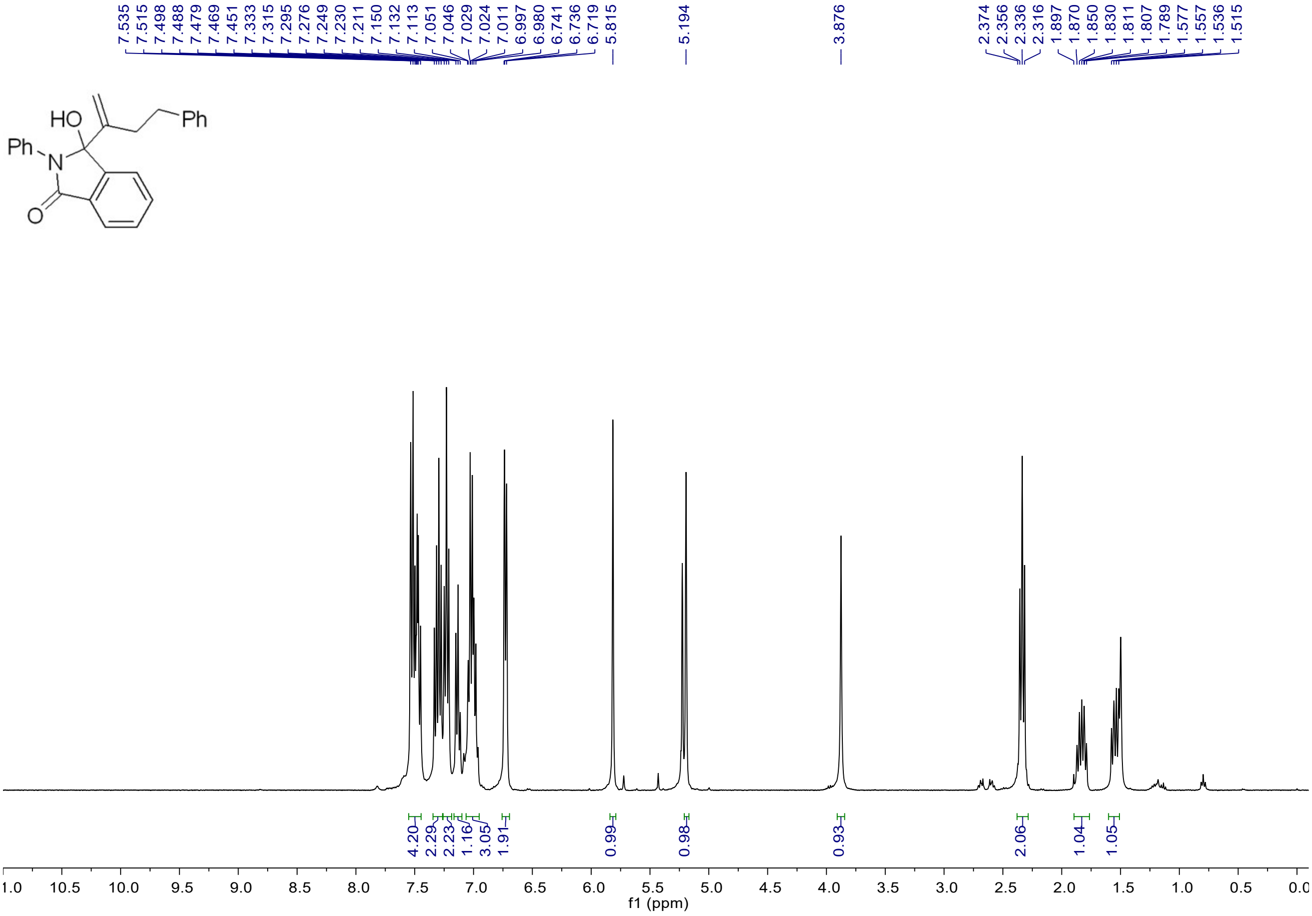

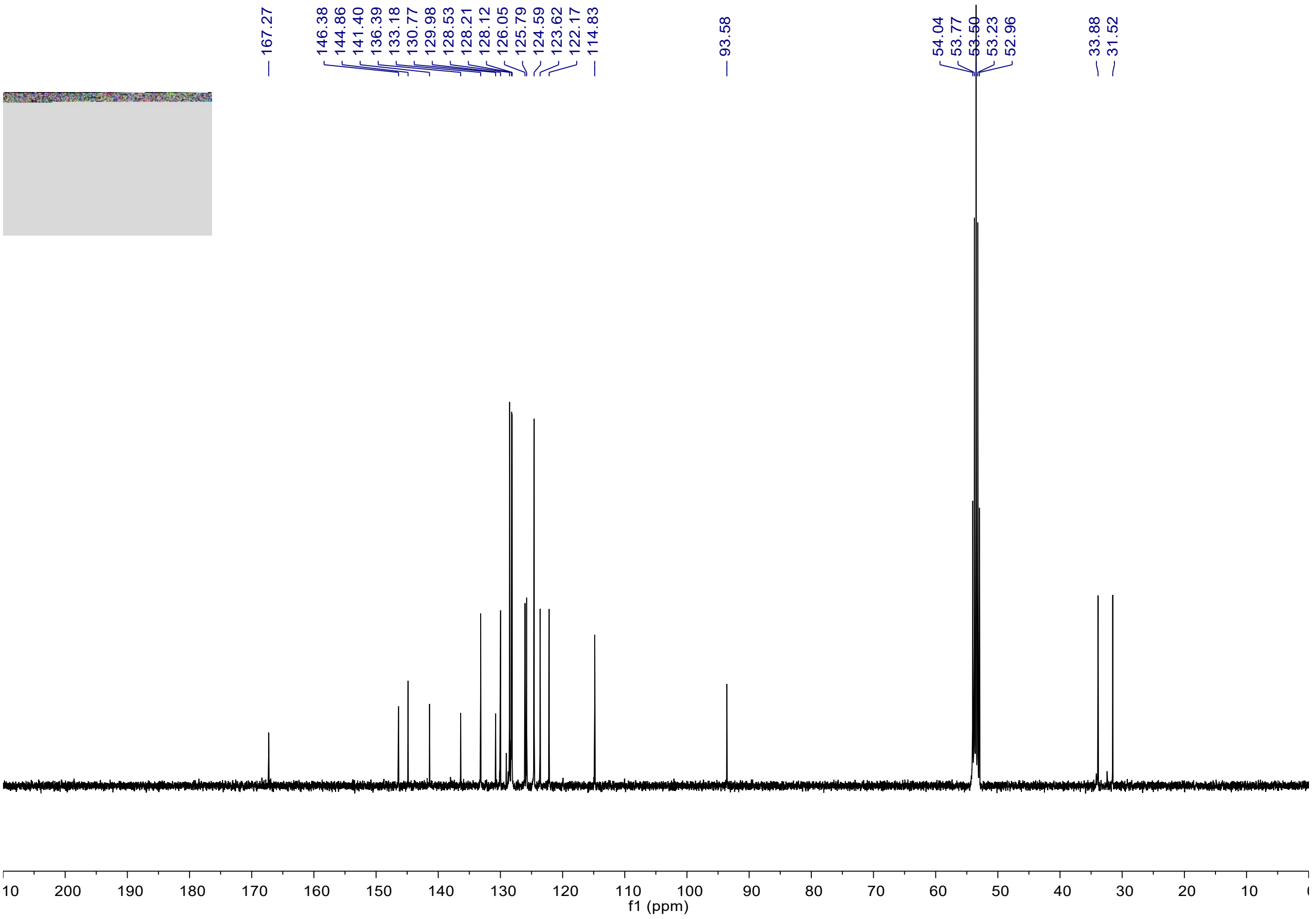

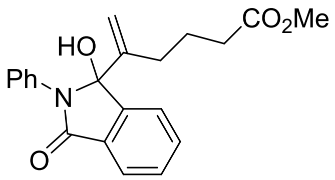

methyl 5-(1-hydroxy-3-oxo-2-phenylisoindolin-1-yl) hex-5-enoate (3ga). A white solid, 23 mg, 32% yield; M.p.: 129-130 °C; ^{1}H NMR (CD_2Cl_2, 400 MHz, TMS) δ 7.61-7.53 (m, 3H), 7.50-7.45 (m, 1H), 7.42-7.33 (m, 2H), 7.30-7.24 (m, 2H), 7.22-7.16 (m, 1H), 5.92-5.86 (m, 1H), 5.20 (q, J = 1.4 Hz, 1H), 4.60 (s, 1H), 3.47 (s, 3H), 1.97-1.91 (m, 2H), 1.64-1.53 (m, 1H), 1.46-1.37 (m, 2H), 1.37-1.28 (m, 1H); ^{13}C NMR (CD_2Cl_2, 100 MHz, TMS) δ 173.4, 167.4, 146.6, 144.6, 136.3, 133.1, 130.6, 129.8, 128.4, 125.9, 124.3, 123.5, 122.1, 114.6, 93.6, 51.3, 33.0, 29.0, 22.6; IR (neat) ν 3227, 2954, 1732, 1676, 1595, 1496, 1431, 1361, 1295, 1141, 1066, 1008, 917, 873, 774 cm^{-1}; HRMS (ESI) Calcd. for $C_{21}H_{21}NO_4Na^+$ Requires: 374.1363, Found: 374.1366.

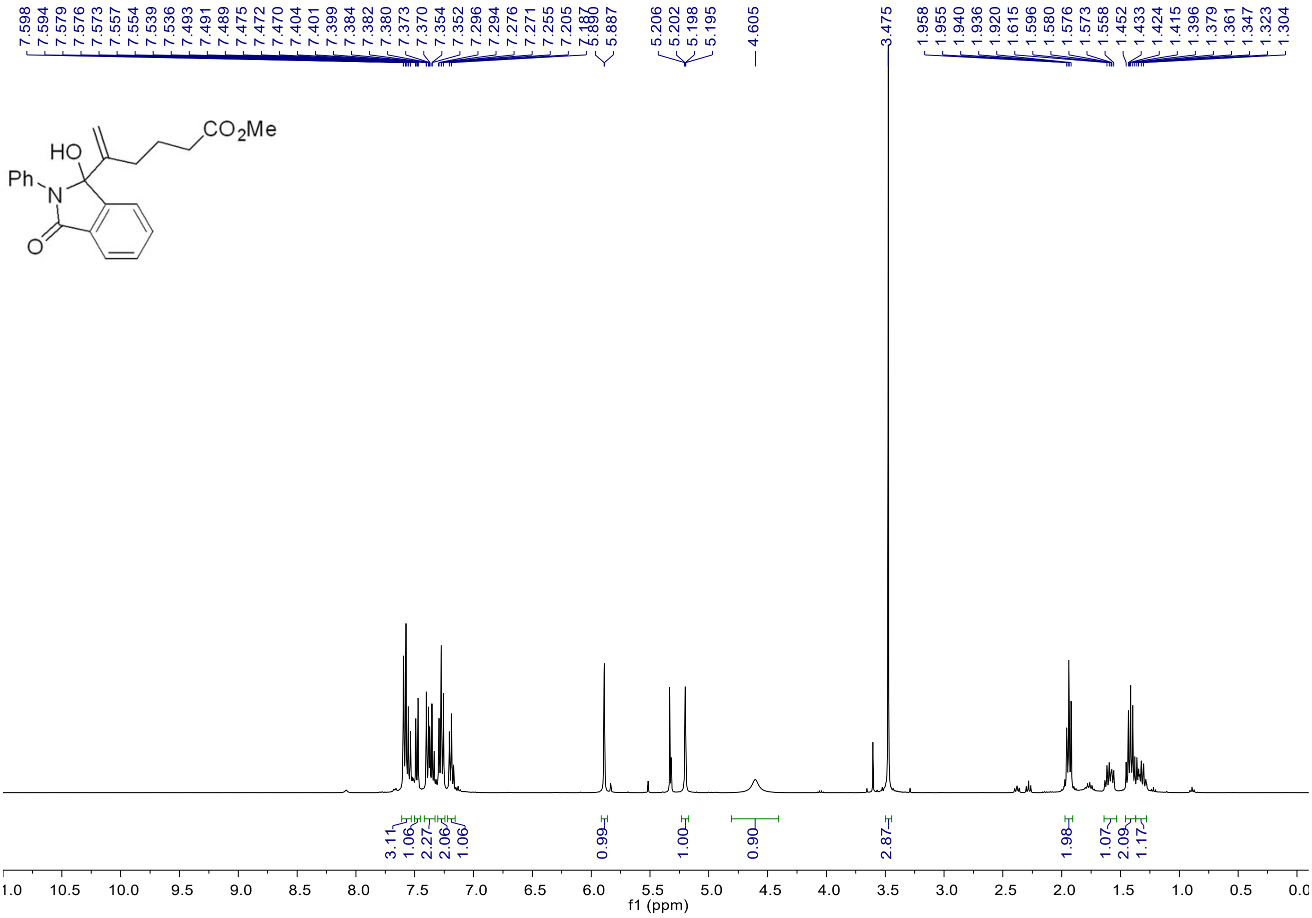
CO2Me
HO
Ph
N
O
f1 (ppm)

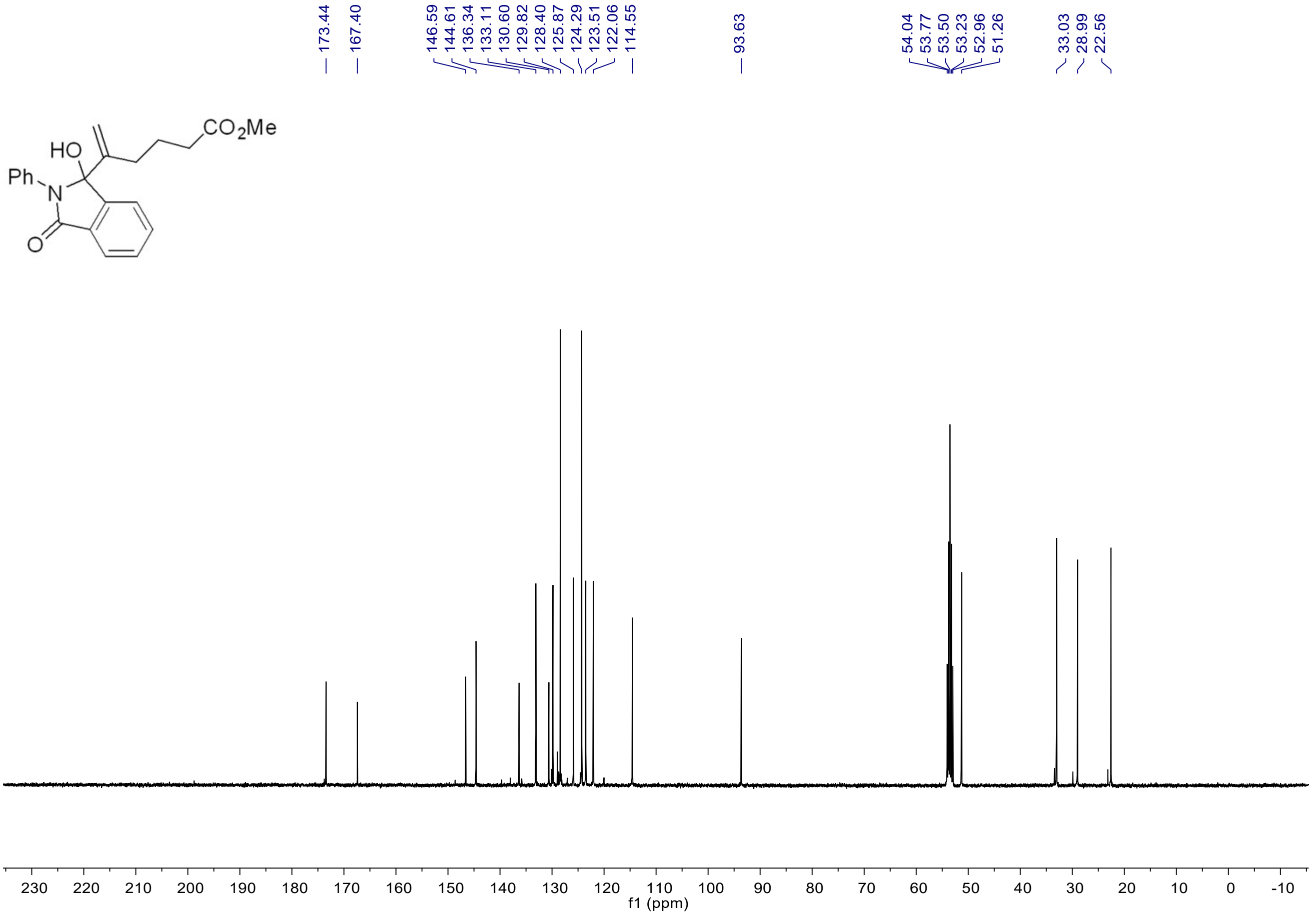
CO2Me
HO
Ph
N
O
f1 (ppm)

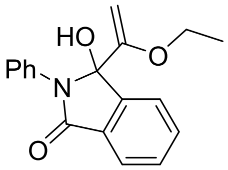

3-(1-ethoxyvinyl)-3-hydroxy-2-phenylisoindolin-1-one (3ha). A white solid, 56 mg, 95% yield; M.p.: 163-164 °C; ^{1}H NMR ($CDCl_3$, 400 MHz, TMS) δ 7.56-7.48 (m, 1H), 7.48-7.38 (m, 4H), 7.37-7.29 (m, 1H), 7.29-7.16 (m, 3H), 4.74 (s, 1H), 4.69 (s, 1H), 4.19-4.04 (m, 1H), 3.51-3.39 (m, 2H), 0.92 (t, J = 7.0 Hz, 3H); ^{13}C NMR ($CDCl_3$, 100 MHz, TMS) δ 167.9, 157.6, 146.2, 135.7, 132.5, 130.9, 129.5, 128.3, 126.5, 126.3, 123.3, 121.8, 91.2, 85.0, 63.4, 13.9; IR (neat) ν 3298, 2979, 2848, 1674, 1637, 1496, 1365, 1285, 1202, 1091, 1077, 976 cm^{-1}; HRMS (ESI) Calcd. for $C_{18}H_{17}NO_3Na^+$ Requires: 318.1101, Found: 318.1110.

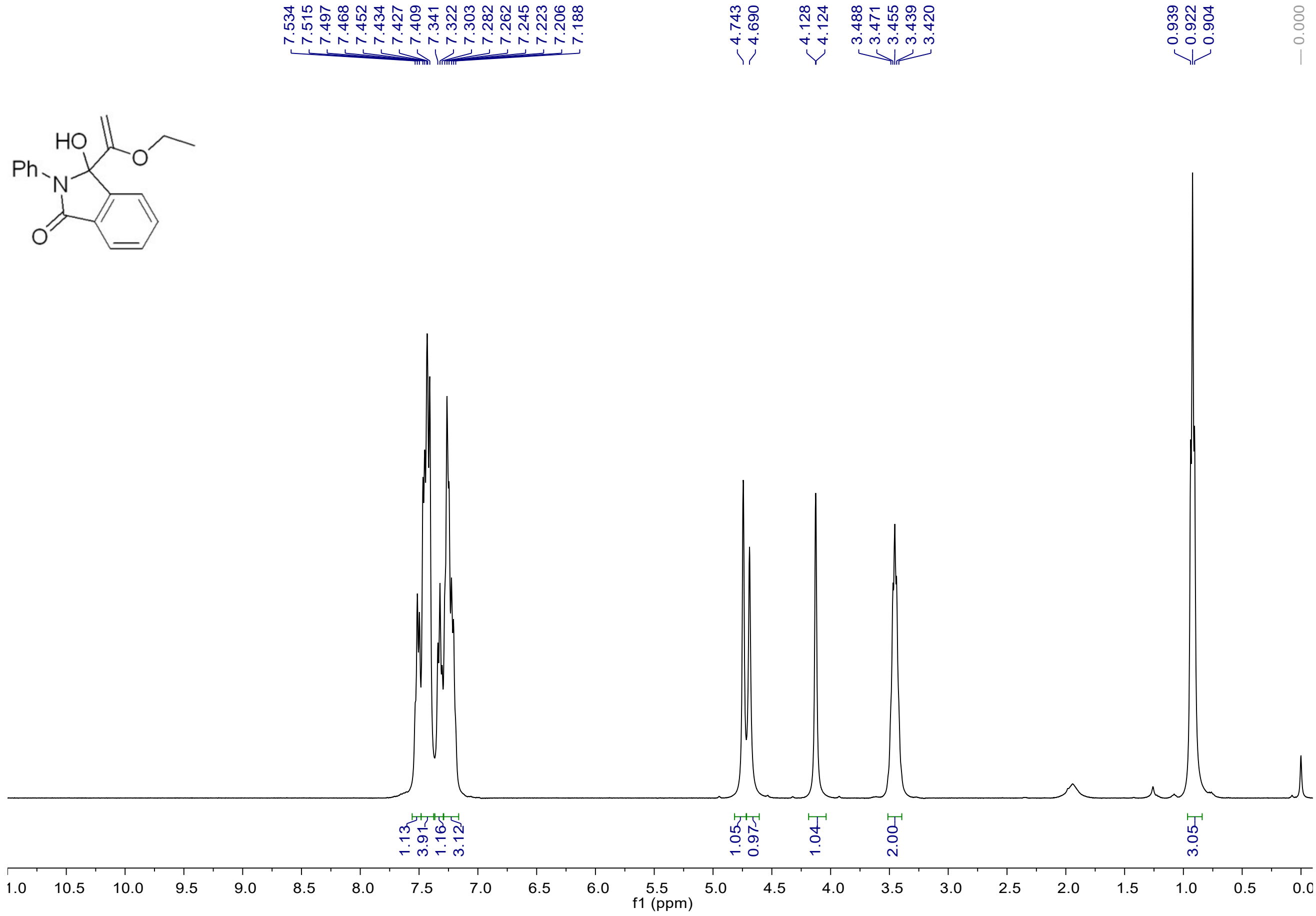

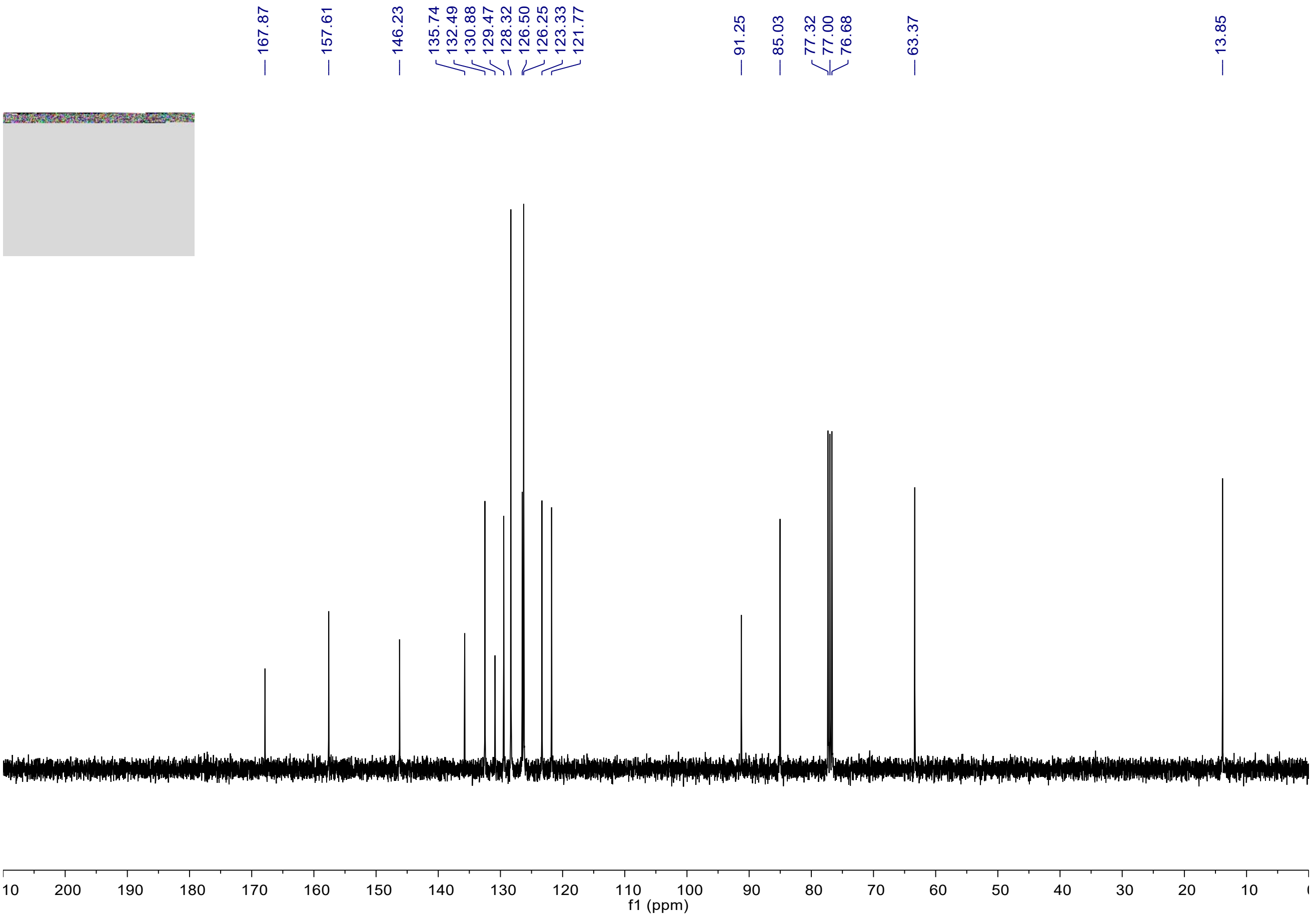

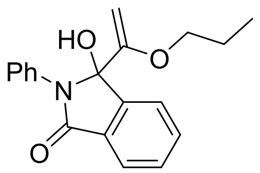

3-hydroxy-2-phenyl-3-(1-propoxyvinyl) isoindolin-1-one (3ia). A white solid, 57 mg, 92% yield; M.p.: 138-139 °C; ^{1}H NMR (CD_2Cl_2, 400 MHz, TMS) δ 7.47 (td, J = 7.3, 1.3 Hz, 1H), 7.41-7.34 (m, 2H), 7.34-7.25 (m, 3H), 7.25-7.11 (m, 3H), 4.66 (d, J = 2.4 Hz, 1H), 4.48 (s, 1H), 4.09 (d, J = 2.5 Hz, 1H), 3.39-3.23 (m, 2H), 1.27 (hept, J = 6.7 Hz, 2H), 0.48 (t, J = 7.4 Hz, 3H); ^{13}C NMR (CD_2Cl_2, 100 MHz, TMS) δ 167.7, 158.1, 146.6, 136.0, 132.7, 131.1, 129.7, 128.5, 126.8, 126.7, 123.2, 121.9, 91.3, 84.8, 69.5, 21.9, 10.0; IR (neat) ν 3278, 2966, 2933, 2871, 1674, 1496, 1426, 1366, 1336, 1206, 1095, 1051, 915 cm^{-1}; HRMS (ESI) Calcd. for $C_{19}H_{19}NO_3Na^+$ Requires: 332.1257, Found: 332.1250.

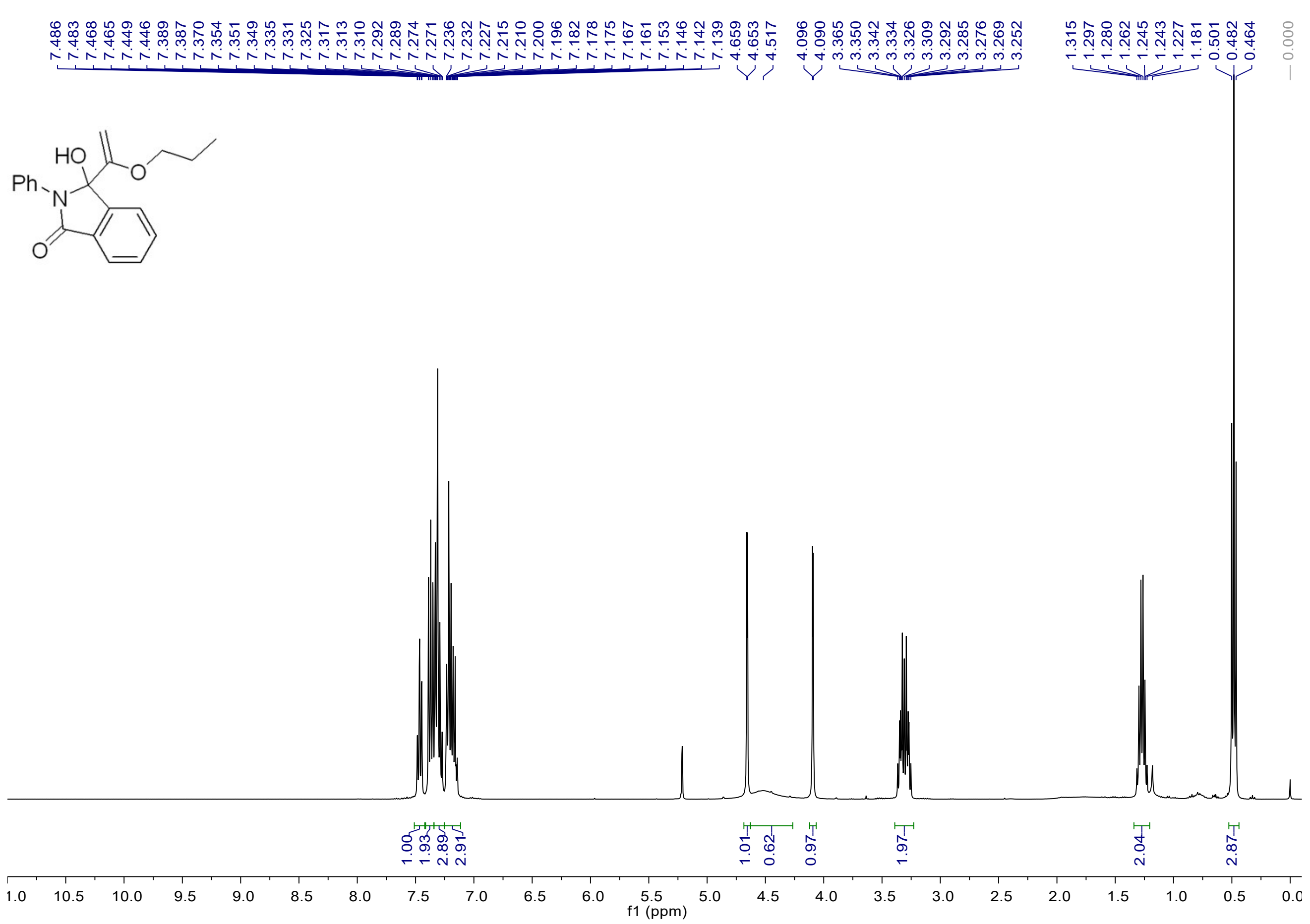
7.486 7.483 7.468 7.465 7.449 7.446 7.389 7.387 7.370 7.354 7.351 7.349 7.335 7.331 7.325 7.317 7.313 7.310 7.292 7.289 7.274 7.271 7.236 7.232 7.227 7.215 7.210 7.200 7.196 7.182 7.178 7.175 7.167 7.161 7.153 7.146 7.142 7.139
4.659 4.653 4.517
4.096 4.090 3.365 3.350 3.342 3.334 3.326 3.309 3.292 3.285 3.276 3.269 3.252
1.315 1.297 1.280 1.262 1.245 1.243 1.227 1.181 0.501 0.482 0.464
0.000
HO
Ph
N
O
1.00 1.93 2.89 2.91
1.01 0.62 0.97
1.97
2.04
2.87
f1 (ppm)

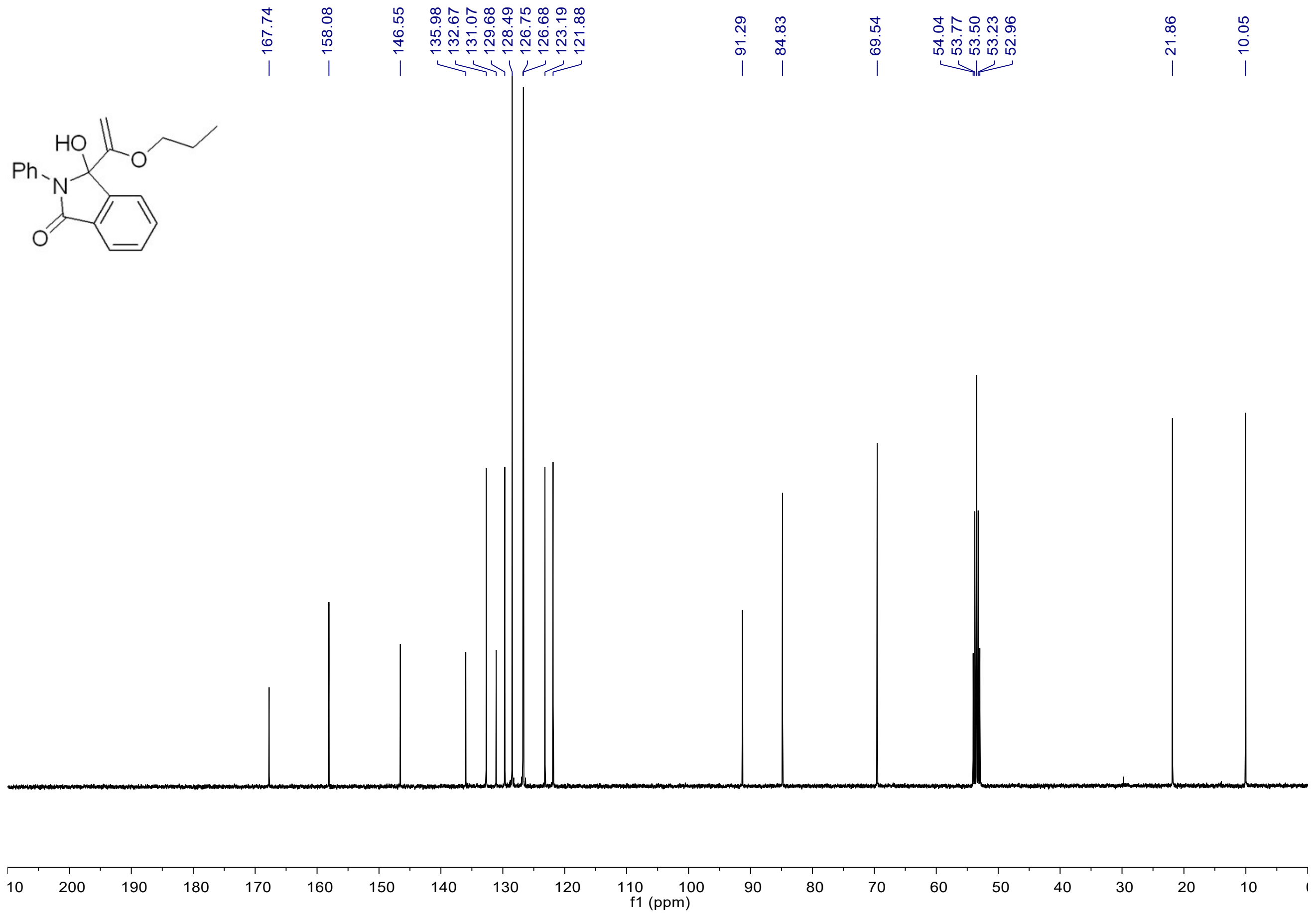
167.74
158.08
146.55
135.98 132.67 131.07 129.68 128.49 126.75 126.68 123.19 121.88
91.29
84.83
69.54
54.04 53.77 53.50 53.23 52.96
21.86
10.05
HO
Ph
N
O
f1 (ppm)

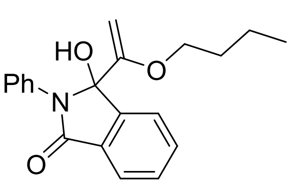

3-(1-butoxyvinyl)-3-hydroxy-2-phenylisoindolin-1-one (3ja). A white solid, 59 mg, 92% yield; M.p.: 123-124 °C; ^{1}H NMR (CD_2Cl_2, 400 MHz, TMS) δ 7.45 (td, *J* = 7.3, 1.6 Hz, 1H), 7.37 (d, *J* = 7.5 Hz, 1H), 7.35-7.24 (m, 4H), 7.24-7.10 (m, 3H), 4.66 (d, *J* = 2.4 Hz, 1H), 4.56 (s, 1H), 4.09 (d, *J* = 2.5 Hz, 1H), 3.42-3.27 (m, 2H), 1.28-1.18 (m, 2H), 0.89 (h, *J* = 7.4 Hz, 2H), 0.57 (t, *J* = 7.4 Hz, 3H); ^{13}C NMR (CD_2Cl_2, 100 MHz, TMS) δ 167.8, 158.1, 146.6, 136.0, 132.7, 131.0, 129.7, 128.5, 126.7, 126.6, 123.2, 121.9, 91.3, 84.8, 67.7, 30.4, 18.9, 13.3; IR (neat) ν 3286, 2953, 2930, 1673, 1594, 1496, 1363, 1202, 1117, 1092, 1054, 940, 826 cm^{-1}; HRMS (ESI) Calcd. for $C_{20}H_{21}NO_3Na^+$ Requires: 346.1414, Found: 346.1421.

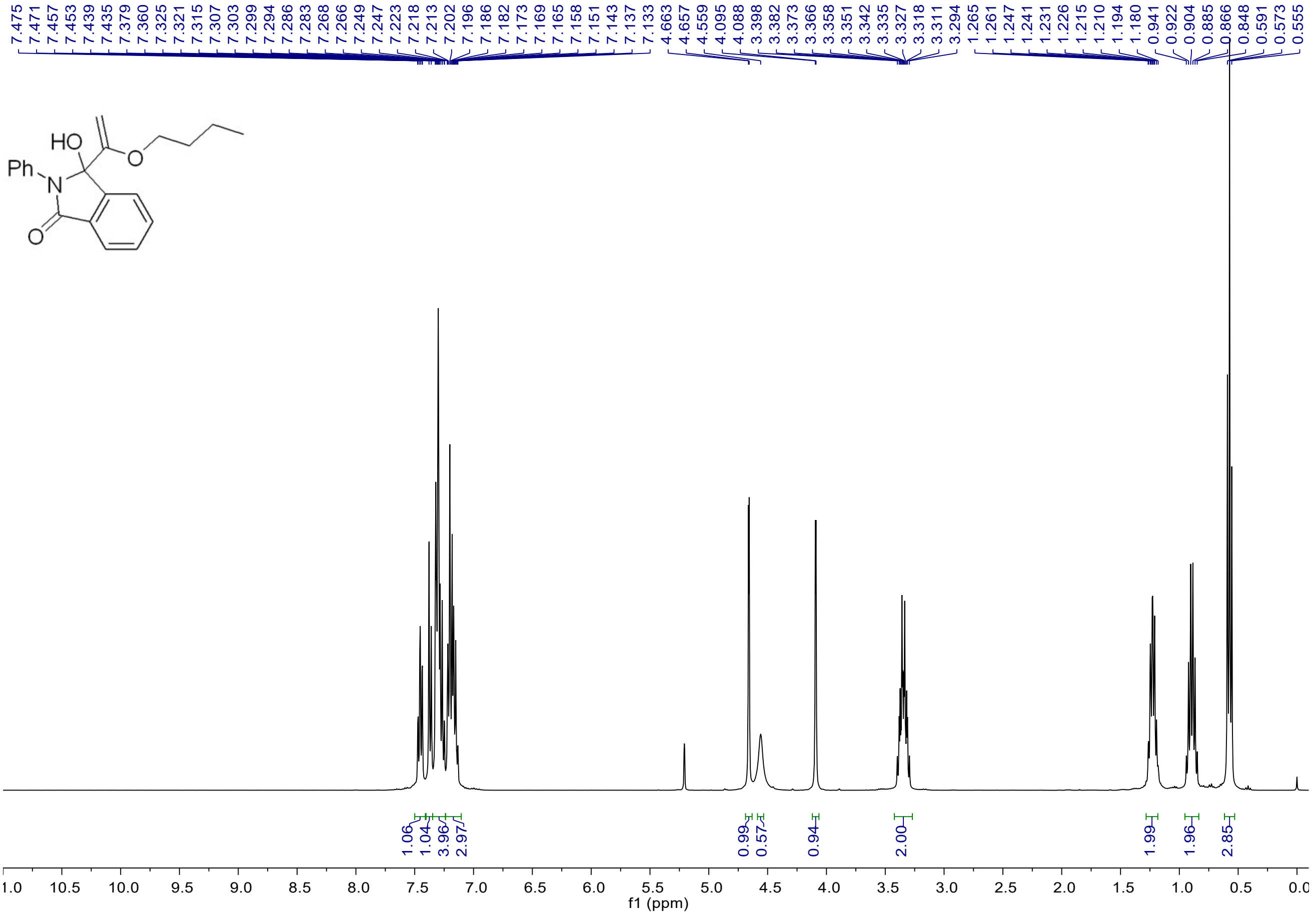

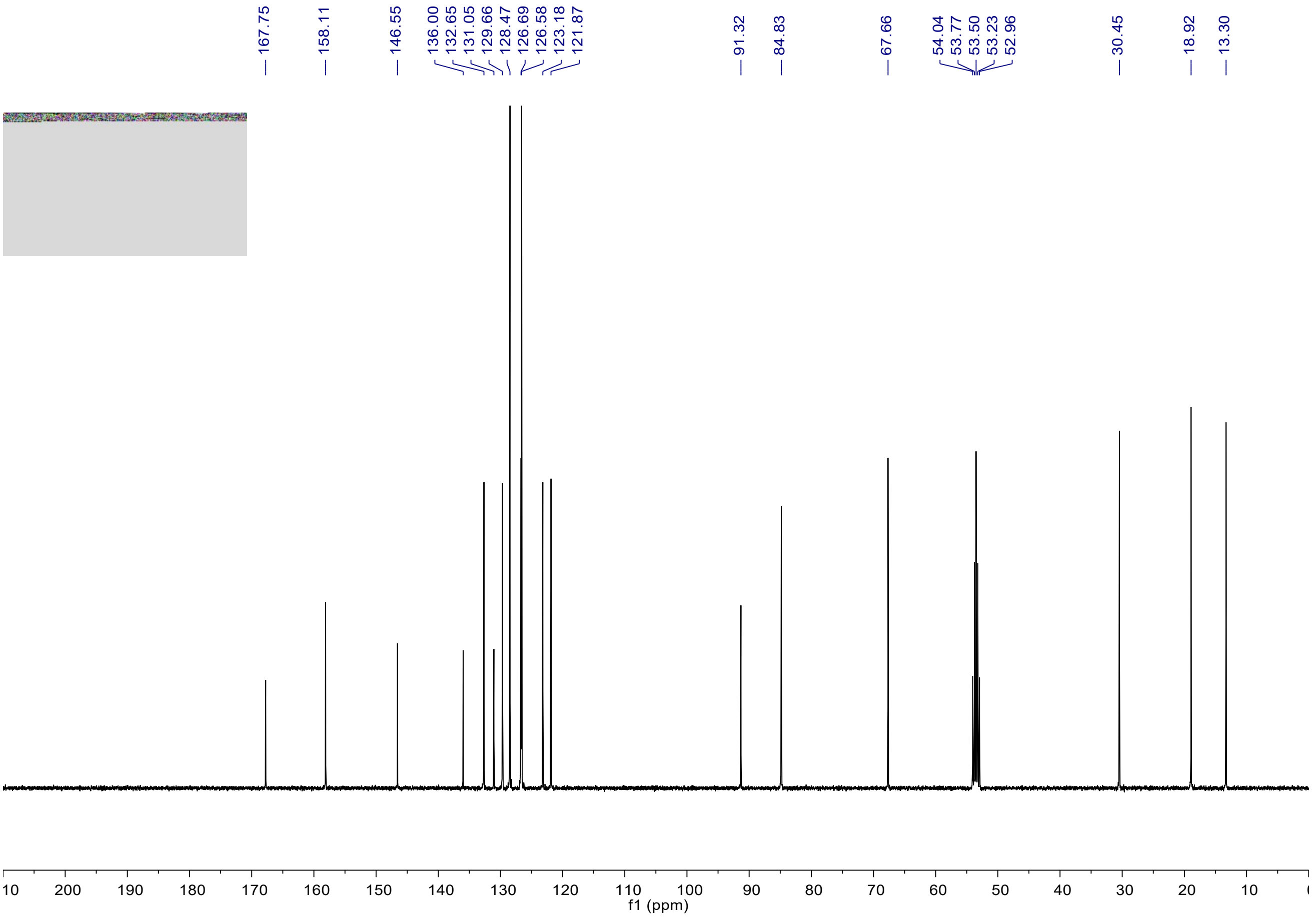

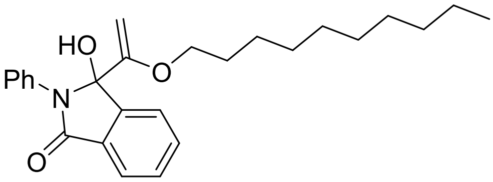

3-(1-(decyloxy)vinyl)-3-hydroxy-2-phenylisoindolin-1-one (3ka). A colorless oil, 71 mg, 87% yield; ^{1}H NMR (CD_2Cl_2, 400 MHz, TMS) δ 7.46 (t, *J* = 7.4 Hz, 1H), 7.41-7.35 (m, 1H), 7.35-7.24 (m, 4H), 7.24-7.11 (m, 3H), 4.66 (s, 1H), 4.54 (s, 1H), 4.09 (s, 1H), 3.42-3.26 (m, 2H), 1.28-1.11 (m, 8H), 1.10-0.91 (m, 6H), 0.89-0.73 (m, 5H); ^{13}C NMR (CD_2Cl_2, 100 MHz, TMS) δ 167.7, 158.0, 146.5, 136.0, 132.6, 131.0, 129.7, 128.5, 126.7, 126.6, 123.2, 121.8, 91.3, 84.8, 67.9, 32.0, 29.5, 29.5, 29.4, 29.0, 28.4, 25.7, 22.8, 14.0; IR (neat) ν 3315, 2923, 2853, 1686, 1500, 1366, 1128, 1094, 824, 755, 696 cm^{-1}; HRMS (ESI) Calcd. for $C_{26}H_{33}NO_3Na^+$ Requires: 430.2353, Found: 430.2349.

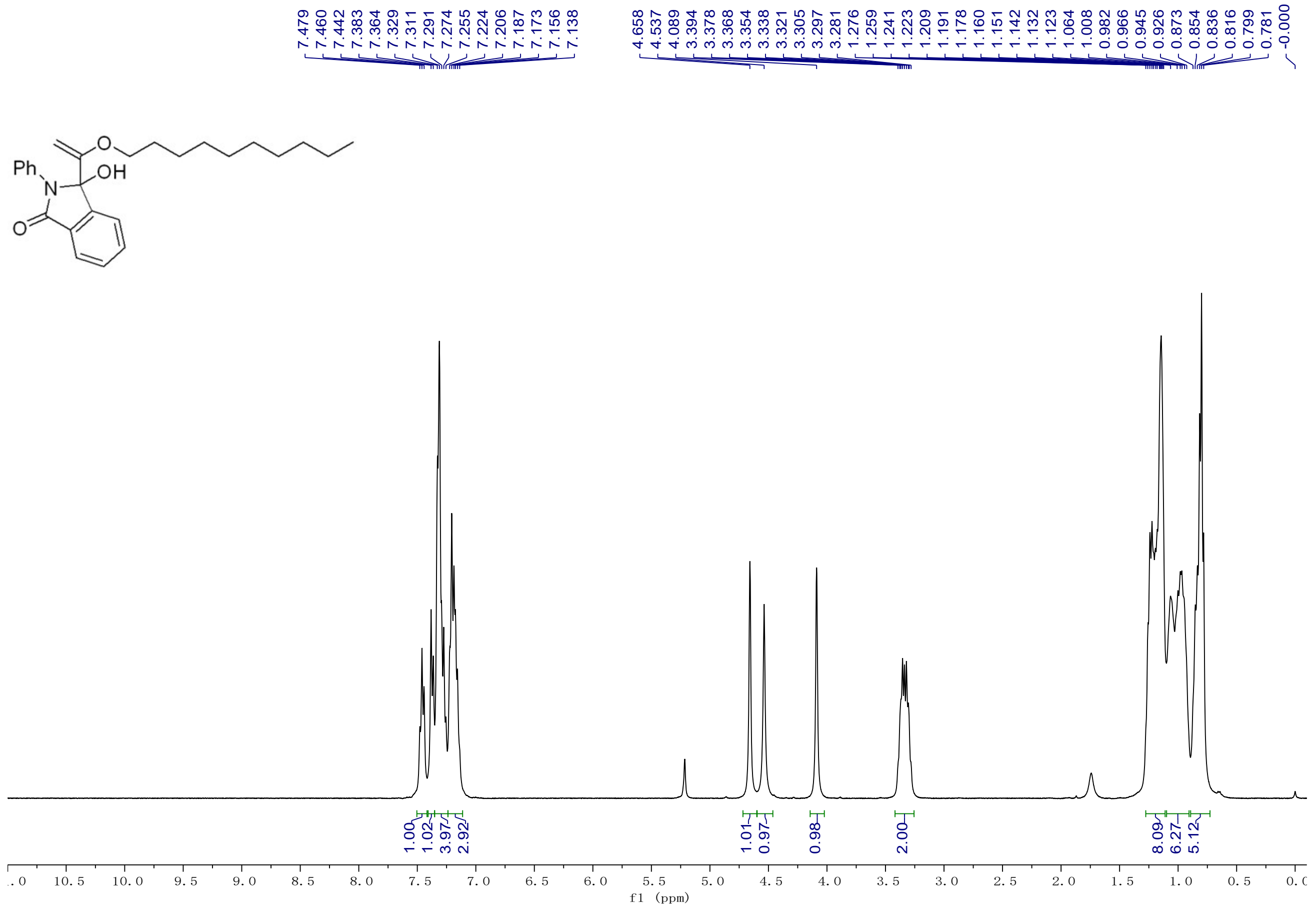
Ph
N
OH
O
f1 (ppm)

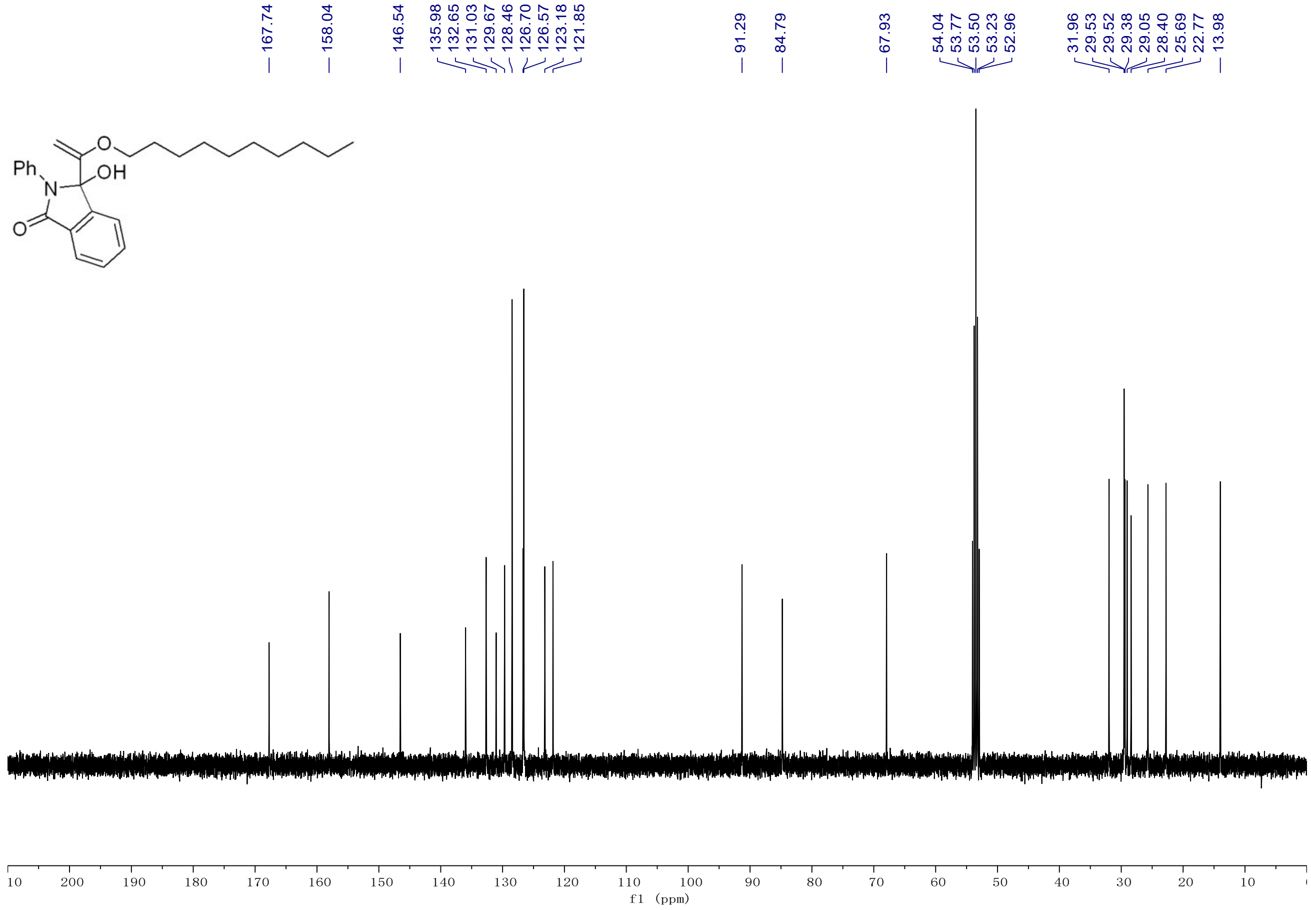
Ph
N
OH
O
f1 (ppm)

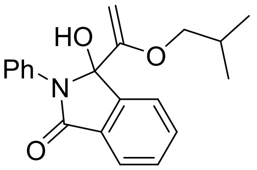

3-hydroxy-3-(1-isobutoxyvinyl)-2-phenylisoindolin-1-one (3la). A white solid, 58 mg, 90% yield; M.p.: 158-159 °C; ^{1}H NMR (CD_2Cl_2, 400 MHz, TMS) δ 7.46 (td, J = 7.4, 1.3 Hz, 1H), 7.37 (d, J = 7.5 Hz, 1H), 7.35-7.25 (m, 4H), 7.25-7.11 (m, 3H), 4.79-4.46 (m, 2H), 4.08 (d, J = 2.4 Hz, 1H), 3.18 (dd, J = 9.2, 6.0 Hz, 1H), 3.06 (dd, J = 9.2, 6.6 Hz, 1H), 1.59-1.46 (m, 1H), 0.51 (d, J = 6.7 Hz, 3H), 0.46 (d, J = 6.7 Hz, 3H); ^{13}C NMR (CD_2Cl_2, 100 MHz, TMS) δ 167.8, 158.1, 146.6, 136.0, 132.6, 131.1, 129.6, 128.5, 126.7, 126.6, 123.1, 121.8, 91.3, 84.7, 74.2, 27.7, 18.6; IR (neat) ν 3256, 2955, 2911, 2872, 1675, 1614, 1495, 1424, 1365, 1287, 1117, 1095, 1053, 989 cm^{-1}; HRMS (ESI) Calcd. for $C_{20}H_{21}NO_3Na^+$ Requires: 346.1414, Found: 346.1415.

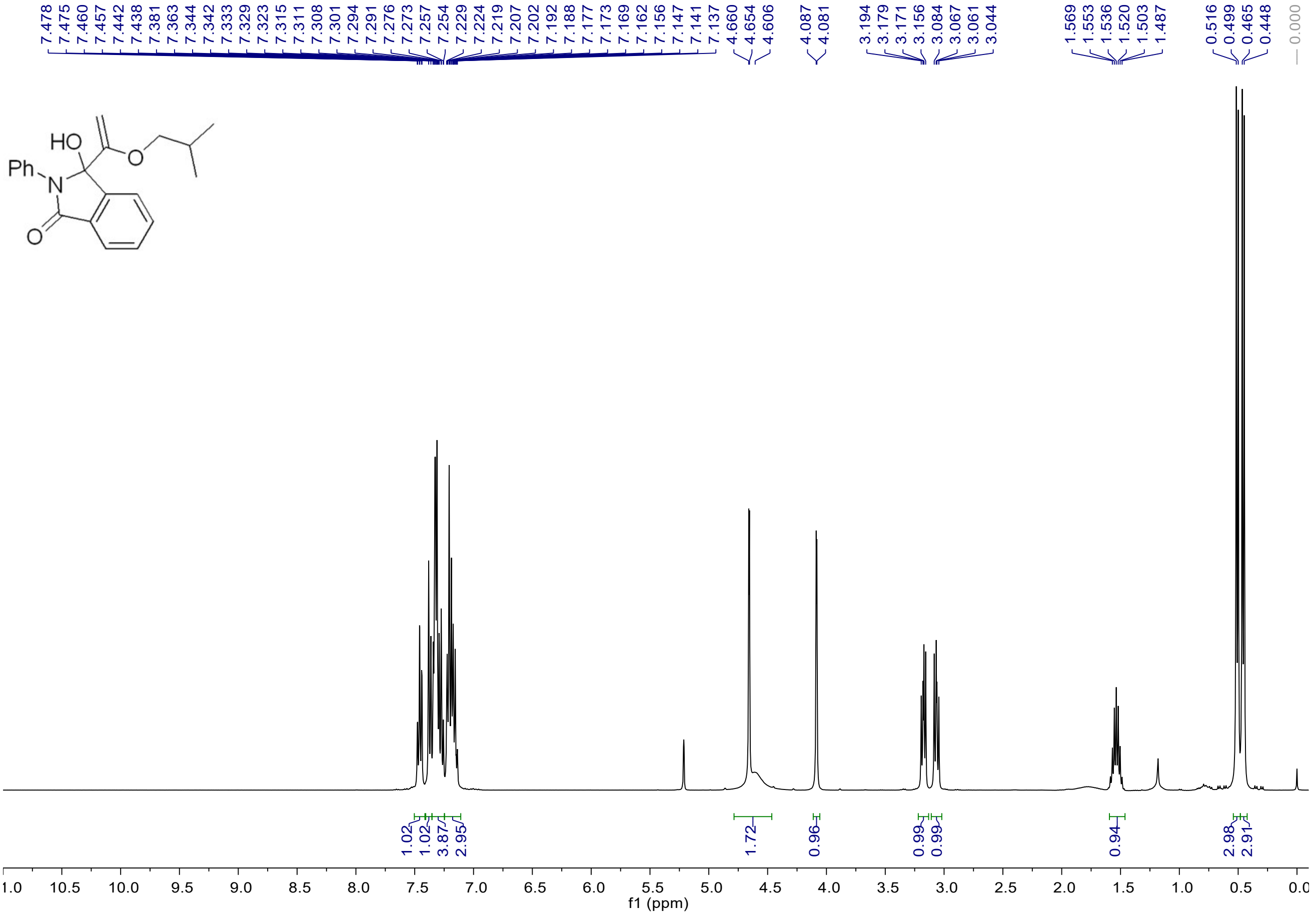

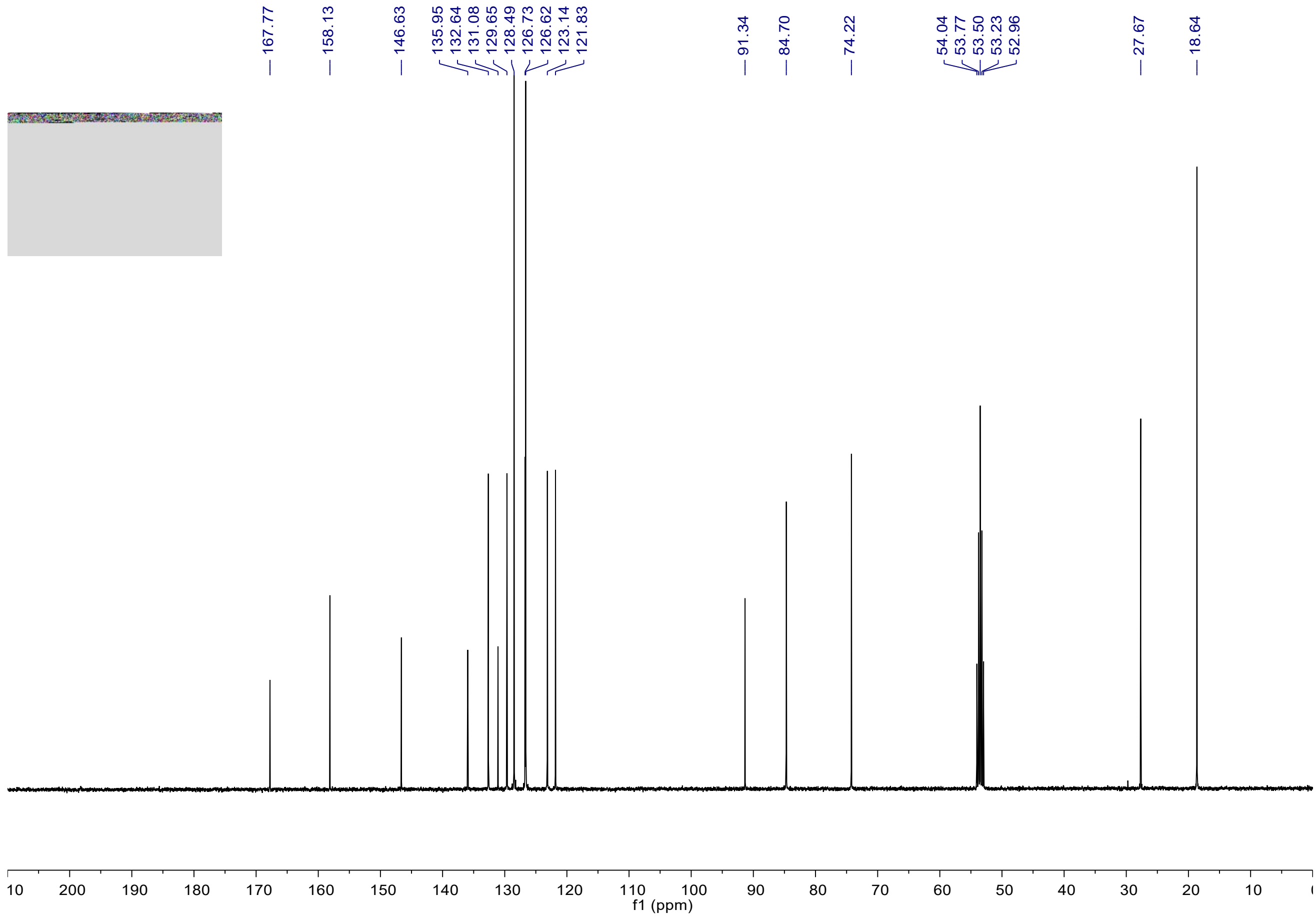

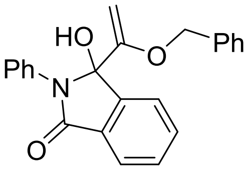

3-(1-(benzyloxy) vinyl)-3-hydroxy-2-phenylisoindolin-1-one (3ma). A white solid, 68 mg, 95% yield; M.p.: 132-133 ^{o}C; ^{1}H NMR (CD_2Cl_2, 400 MHz, TMS) δ 7.44 (td, *J* = 7.4, 1.3 Hz, 1H), 7.39-7.35 (m, 1H), 7.35-7.22 (m, 4H), 7.22-7.13 (m, 3H), 7.13-7.01 (m, 3H), 6.77-6.69 (m, 2H), 5.10-4.61 (m, 2H), 4.43 (d, *J* = 1.8 Hz, 2H), 4.20 (d, *J* = 2.7 Hz, 1H); ^{13}C NMR (CD_2Cl_2, 100 MHz, TMS) δ 167.9, 157.6, 146.4, 136.4, 136.0, 132.8, 130.9, 129.8, 128.5, 128.3, 127.6, 126.7, 126.7, 126.4, 123.3, 122.0, 91.4, 86.5, 69.8; IR (neat) ν 3296, 2923, 2853, 1672, 1500, 1420, 1367, 1278, 1136, 1092, 1049, 953, 902 cm^{-1}; HRMS (ESI) Calcd. for $C_{23}H_{19}NO_3Na^+$ Requires: 380.1257, Found: 380.1255.

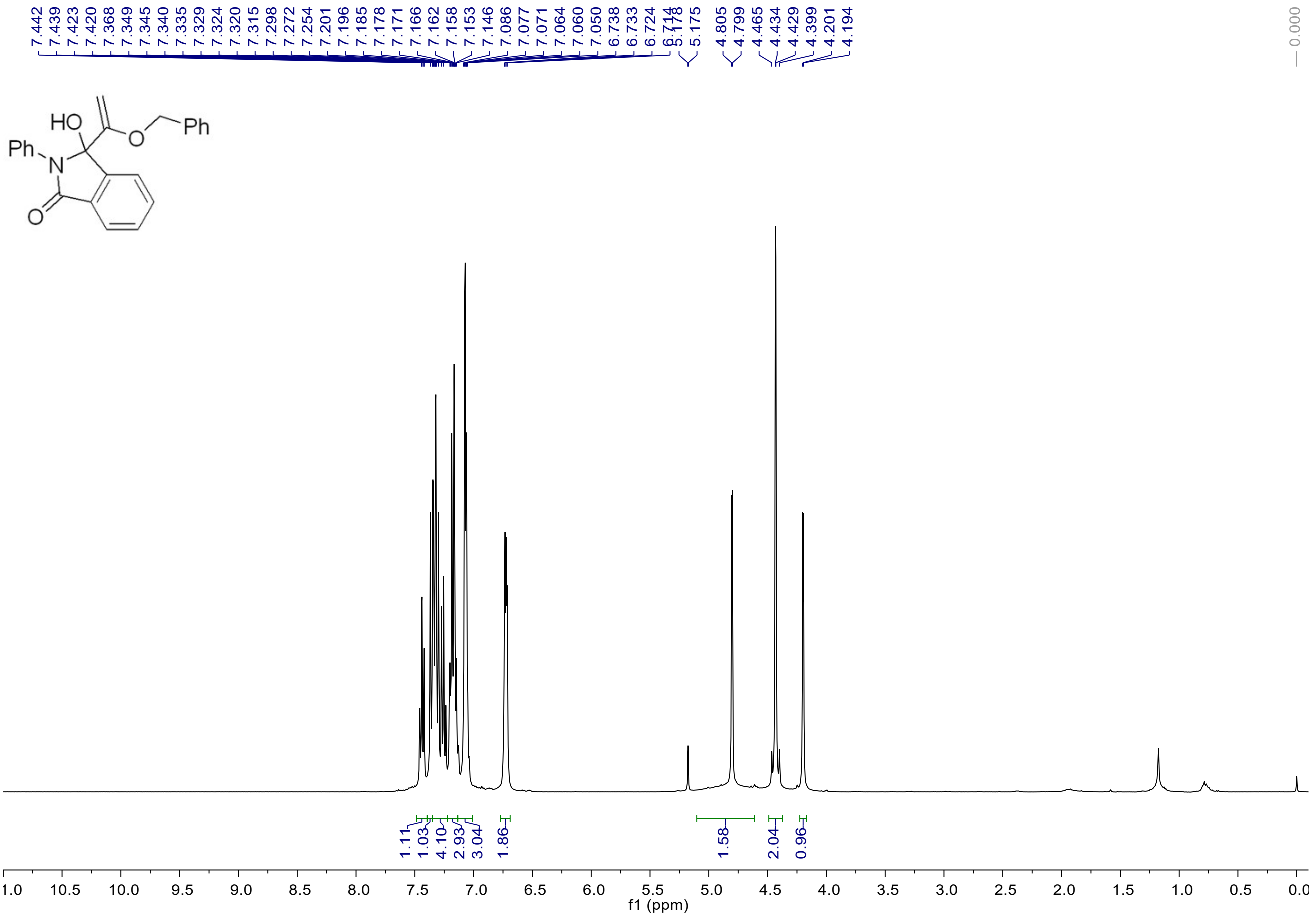

7.442
7.439
7.423
7.420
7.368
7.349
7.345
7.340
7.335
7.329
7.324
7.320
7.315
7.298
7.272
7.254
7.201
7.196
7.185
7.178
7.171
7.166
7.162
7.158
7.153
7.146
7.086
7.077
7.071
7.064
7.060
7.050
6.738
6.733
6.724
6.714
5.178
5.175
4.805
4.799
4.465
4.434
4.429
4.399
4.201
4.194
0.000
HO
O
Ph
Ph
N
O
1.11
1.03
4.10
2.93
3.04
1.86
1.58
2.04
0.96
f1 (ppm)

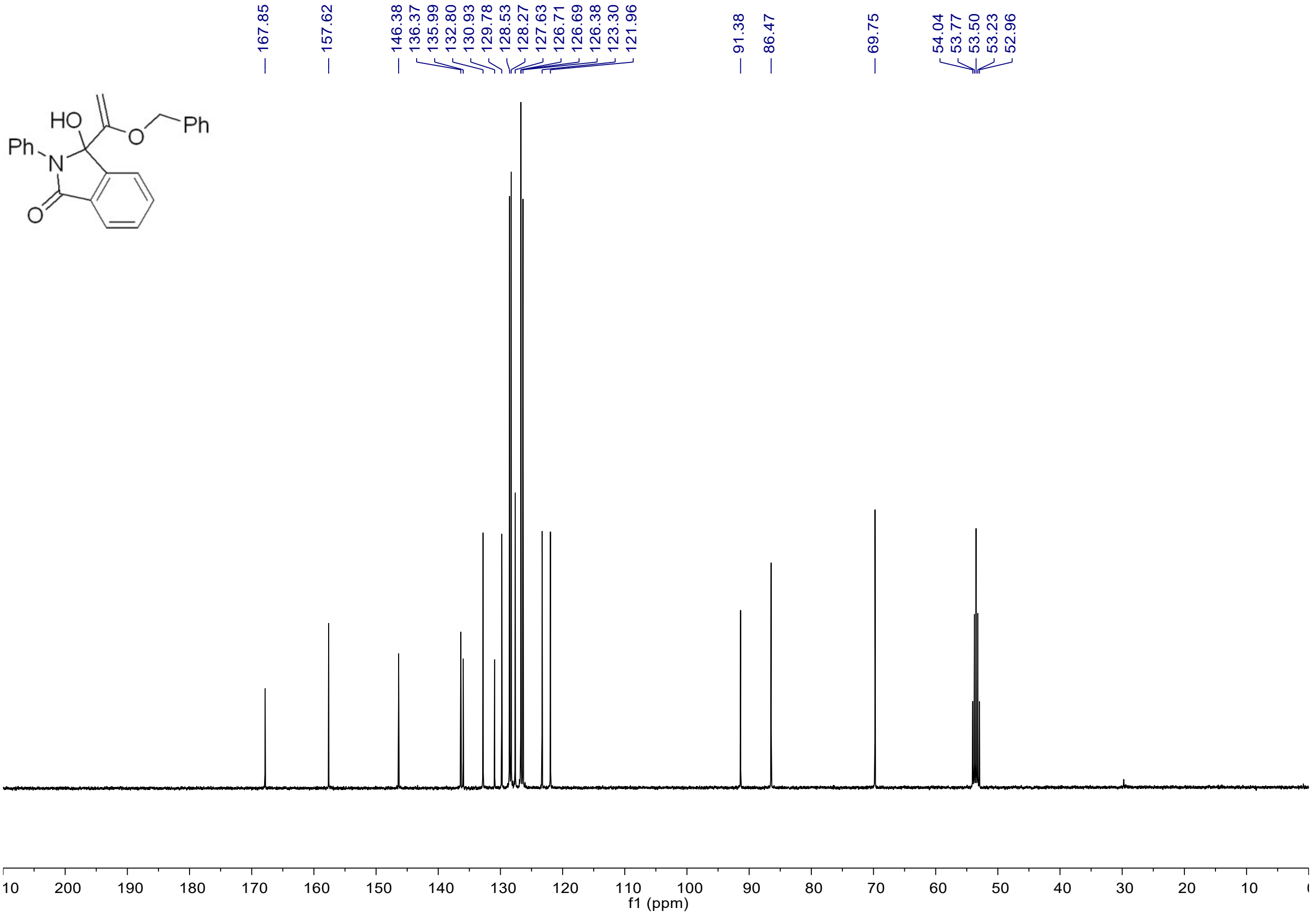

167.85
157.62
146.38
136.37
135.99
132.80
130.93
129.78
128.53
128.27
127.63
126.71
126.69
126.38
123.30
121.96
91.38
86.47
69.75
54.04
53.77
53.50
53.23
52.96
HO
O
Ph
Ph
N
O
f1 (ppm)

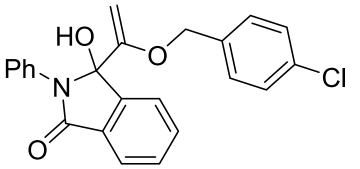

3-(1-((4-chlorobenzyl)oxy)vinyl)-3-hydroxy-2-phenylisoindolin-1-one (3na). A white solid, 53 mg, 68% yield; M.p.: 170-172 °C; ^{1}H NMR (Acetone-d_6, 400 MHz, TMS) δ 7.79-7.71 (m, 3H), 7.70-7.63 (m, 1H), 7.61-7.51 (m, 2H), 7.42-7.33 (m, 2H), 7.29-7.18 (m, 3H), 6.94-6.86 (m, 2H), 6.44-6.37 (m, 1H), 5.09 (d, J = 2.4 Hz, 1H), 4.63 (s, 2H), 4.44 (d, J = 2.4 Hz, 1H); ^{13}C NMR (Acetone-d_6, 100 MHz, TMS) δ 166.7, 158.3, 146.9, 137.2, 135.8, 132.7, 132.6, 131.7, 129.7, 128.3, 128.2, 128.2, 125.8, 125.5, 122.9, 122.0, 91.0, 86.3, 68.6; IR (neat) ν 3286, 1671, 1598, 1492, 1424, 1363, 1121, 1078, 1015, 939, 874, 836, 816, 755, 691 cm^{-1}; HRMS (ESI) Calcd. for $C_{23}H_{18}NO_3NaCl^+$ Requires: 414.0867, Found: 414.0863.

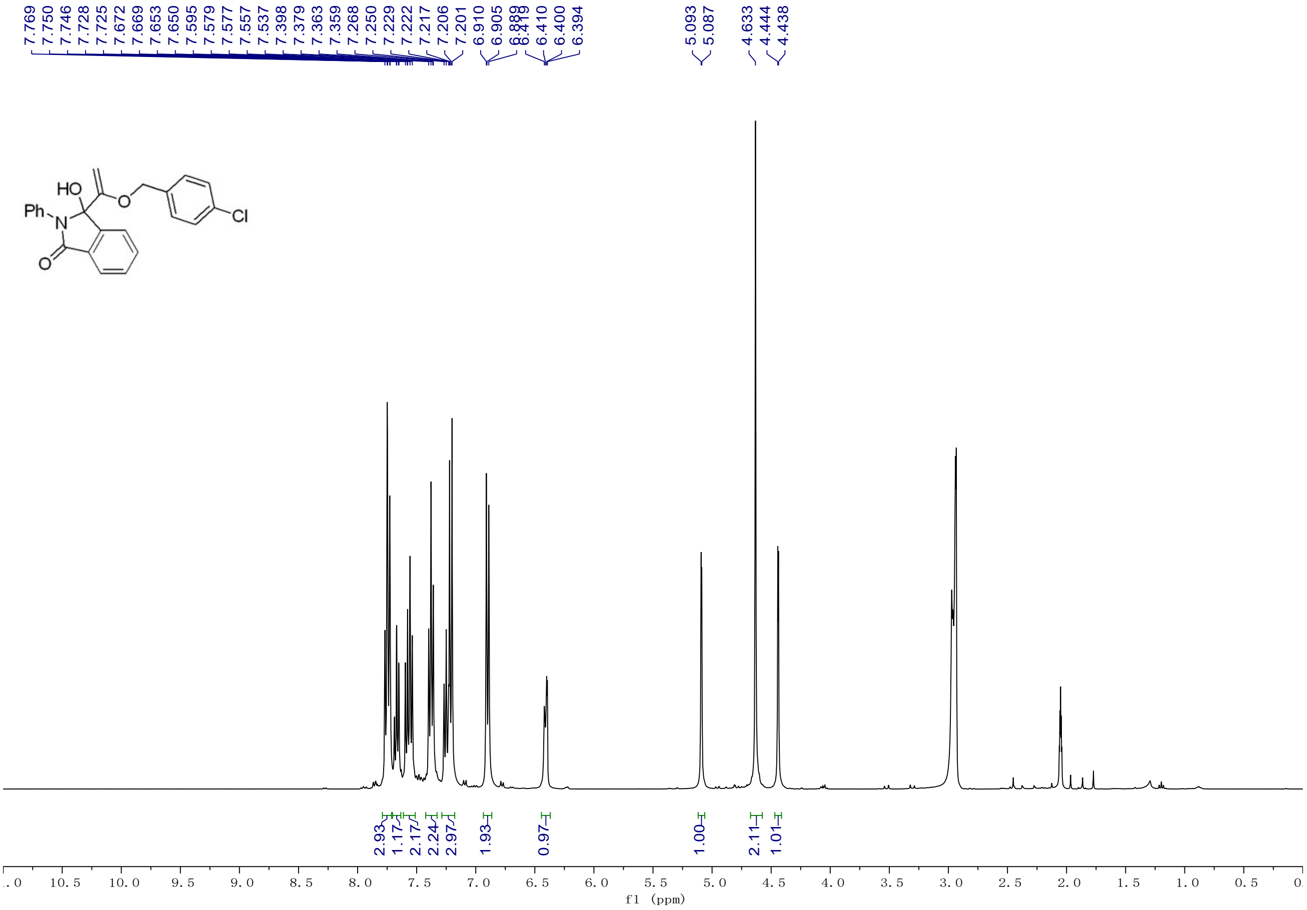

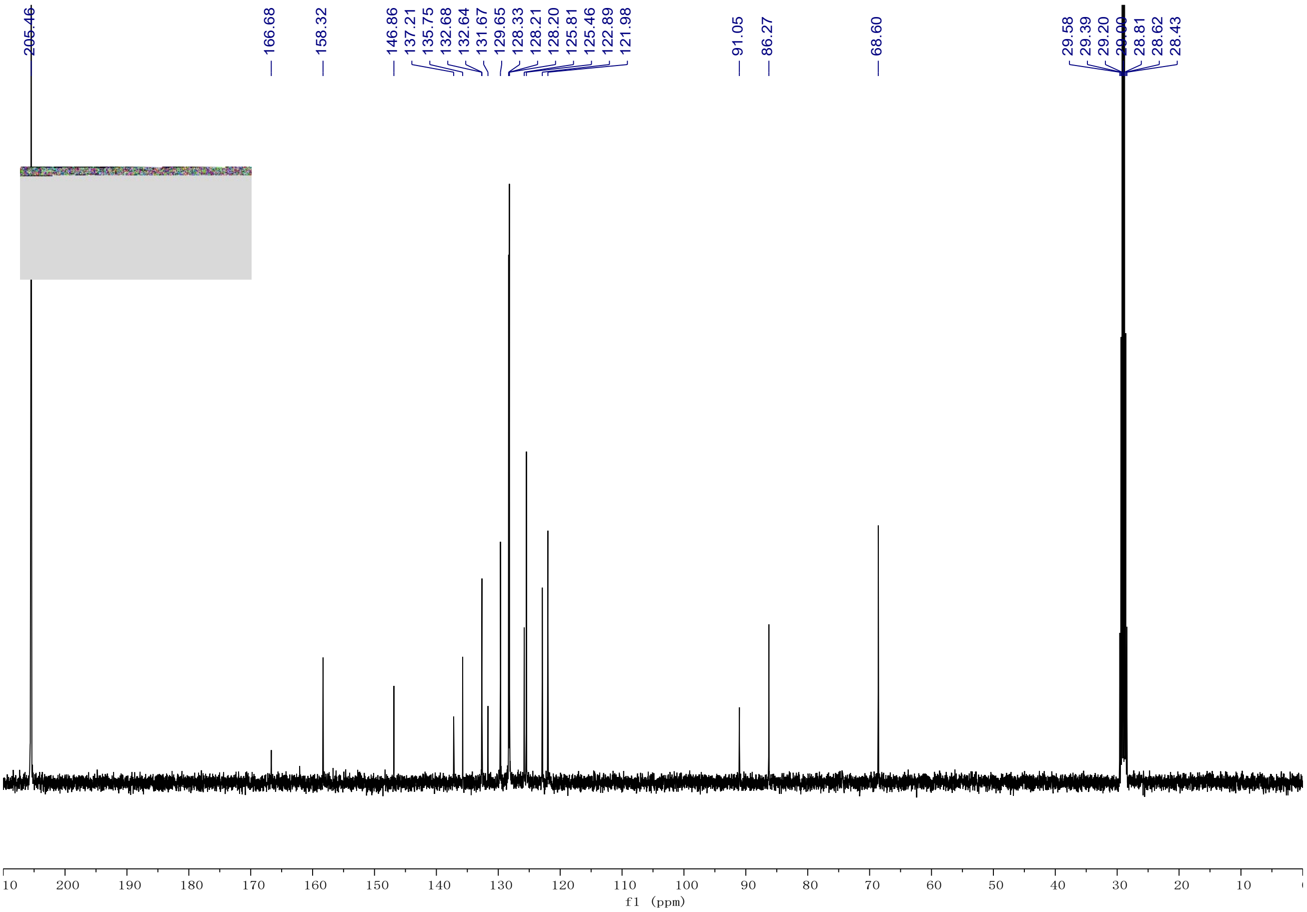

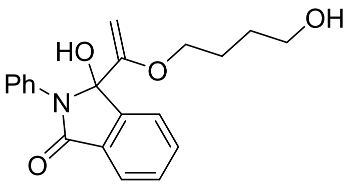

3-hydroxy-3-(1-(4-hydroxybutoxy) vinyl)-2-phenylisoindolin-1-one (3oa). A colorless oil, 55 mg, 81% yield; ^{1}H NMR (CD_2Cl_2, 400 MHz, TMS) δ 7.50-7.39 (m, 2H), 7.39-7.26 (m, 4H), 7.26-7.19 (m, 2H), 7.19-7.11 (m, 1H), 4.69 (d, J = 2.5 Hz, 1H), 4.10 (d, J = 2.4 Hz, 1H), 3.42-3.26 (m, 2H), 3.07 (t, J = 6.5 Hz, 2H), 2.17 (s, 1H), 1.32-1.20 (m, 2H), 1.07-0.95 (m, 2H); ^{13}C NMR (CD_2Cl_2, 100 MHz, TMS) δ 167.9, 158.1, 146.7, 135.9, 132.8, 131.0, 129.6, 128.5, 126.8, 126.6, 123.2, 121.9, 91.4, 85.0, 67.7, 61.7, 28.9, 24.9; IR (neat) ν 3321, 2921, 2872, 1682, 1493, 1366, 1328, 1128, 1095, 984, 874, 758 cm^{-1}; HRMS (ESI) Calcd. for $C_{20}H_{21}NO_4Na^+$ Requires: 362,1363, Found: 362.1355.

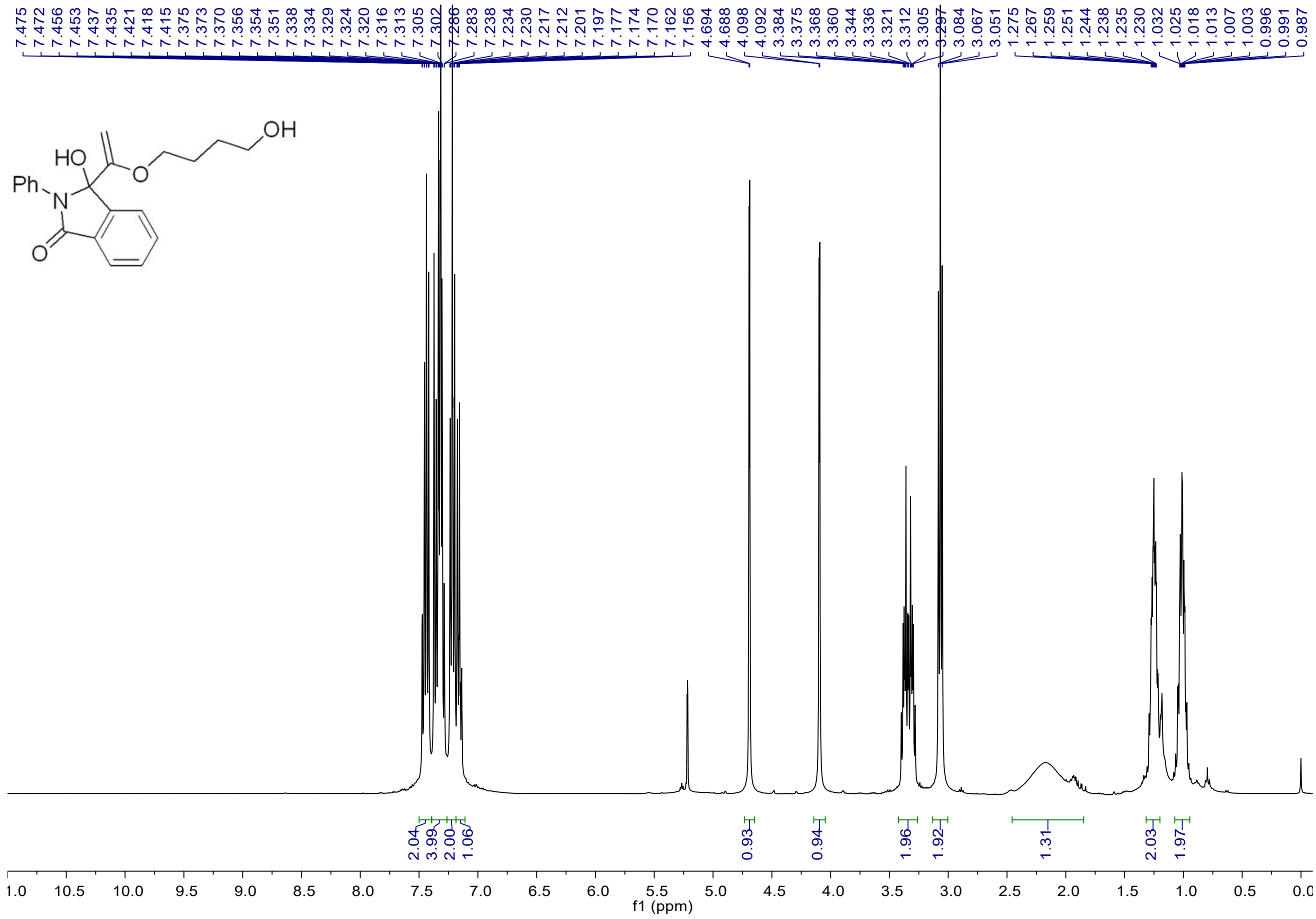

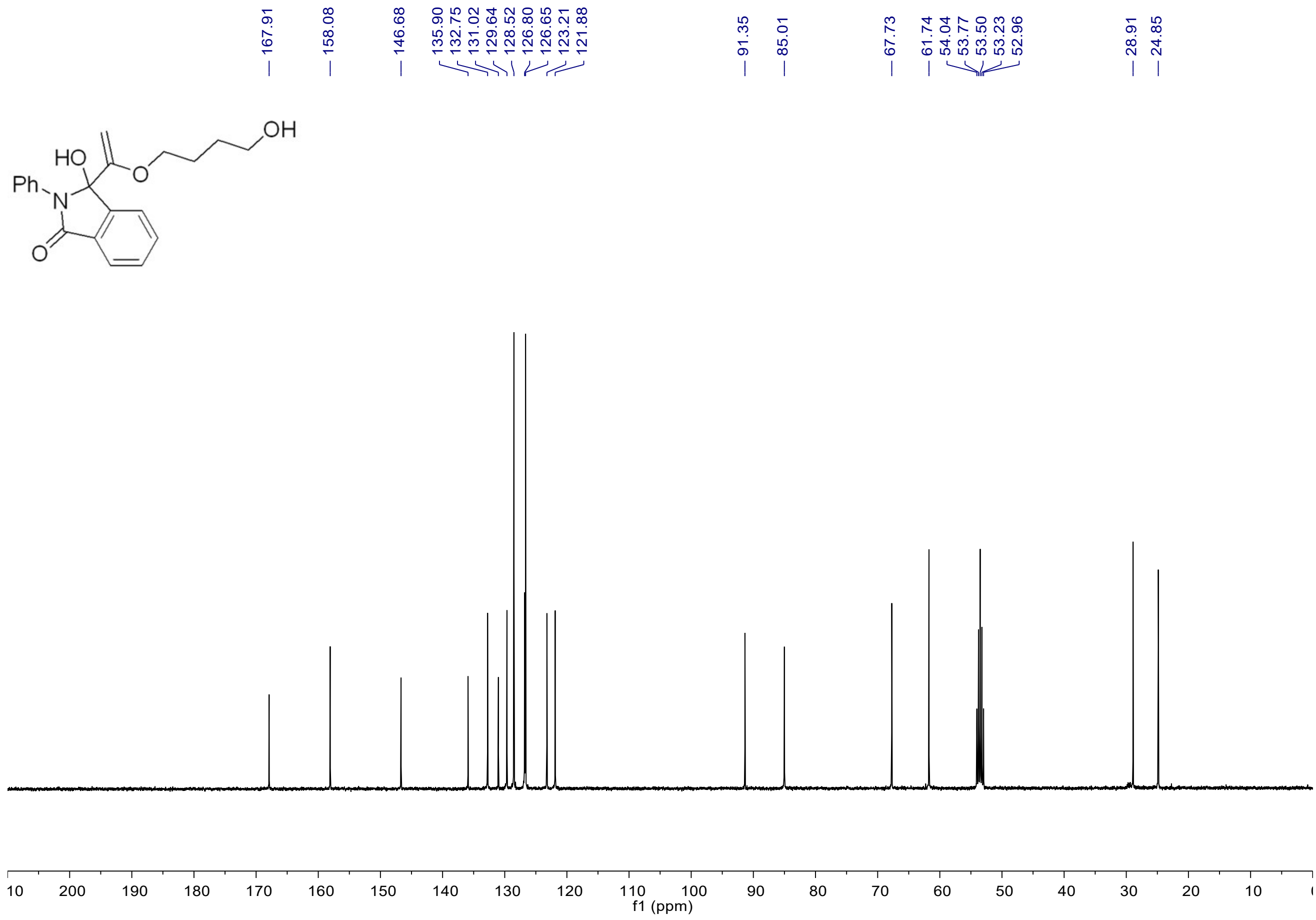

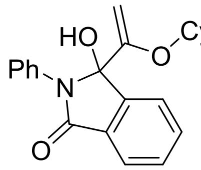

3-(1-(cyclohexyloxy) vinyl)-3-hydroxy-2-phenylisoindolin-1-one (3pa). A white solid, 64 mg, 92% yield; M.p.: 152-153 °C; ^{1}H NMR (CD_2Cl_2, 400 MHz, TMS) δ 7.45 (td, *J* = 7.4, 1.3 Hz, 1H), 7.41-7.31 (m, 4H), 7.27 (td, *J* = 7.4, 1.1 Hz, 1H), 7.24-7.10 (m, 3H), 4.71 (d, *J* = 2.5 Hz, 2H), 4.08 (d, *J* = 2.4 Hz, 1H), 3.76-3.66 (m, 1H), 1.53-1.42 (m, 1H), 1.38-1.26 (m, 2H), 1.26-1.16 (m, 1H), 1.13-0.85 (m, 6H); ^{13}C NMR (CD_2Cl_2, 100 MHz, TMS) δ 167.8, 156.0, 146.8, 136.1, 132.6, 131.1, 129.6, 128.4, 126.6, 123.1, 121.8, 91.5, 84.7, 74.5, 31.1, 29.7, 25.5, 23.1, 22.9; IR (neat) ν 3274, 2935, 2856, 1677, 1634, 1495, 1466, 1370, 1278, 1122, 1094, 1024, 929 cm^{-1}; HRMS (ESI) Calcd. for $C_{22}H_{23}NO_3Na^+$ Requires: 372.1570, Found: 372.1579.

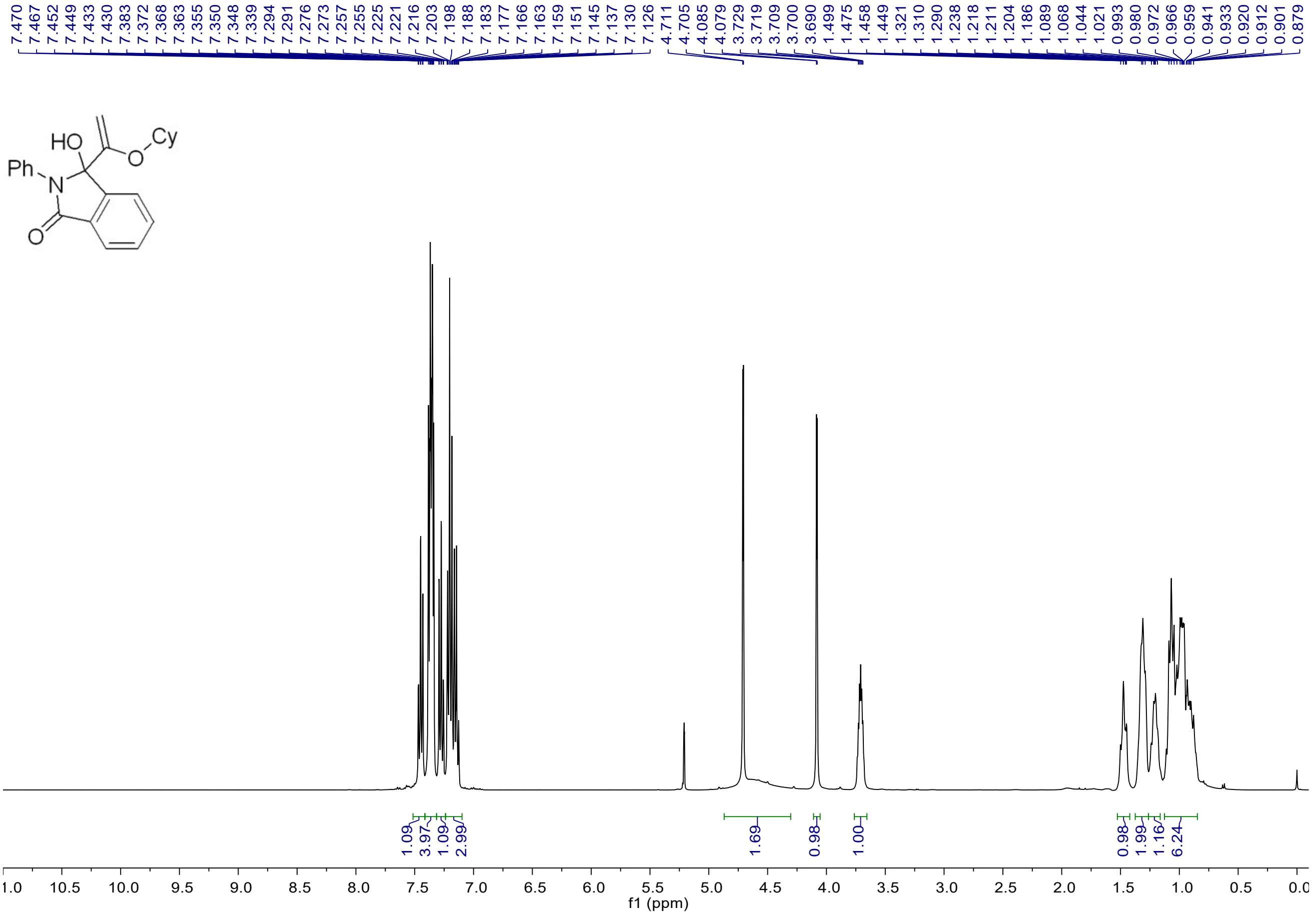

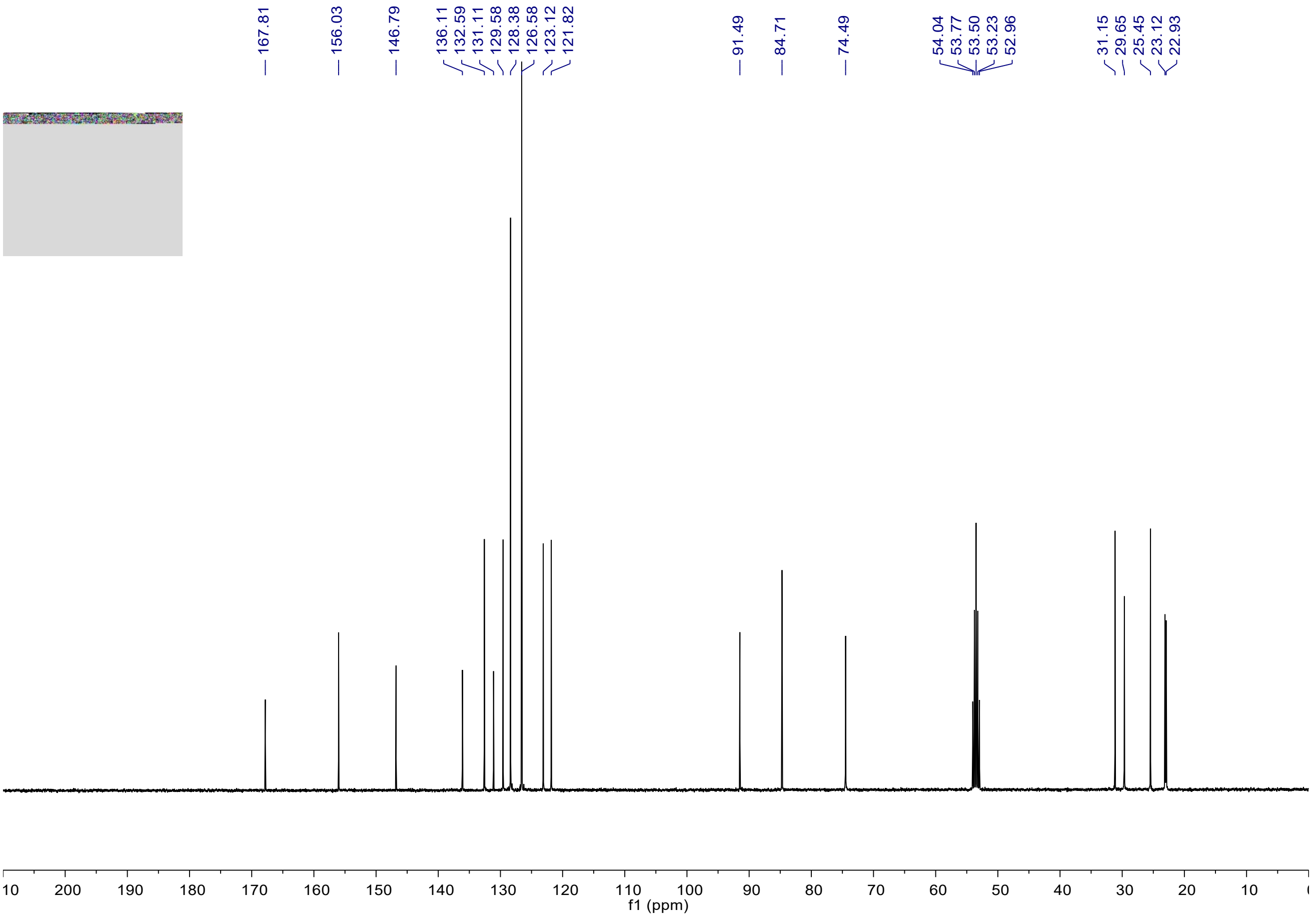

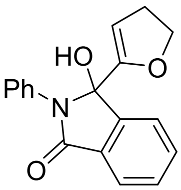

3-(4,5-dihydrofuran-2-yl)-3-hydroxy-2-phenylisoindolin-1-one (3qa). A white solid, 39 mg, 66% yield; M.p.: 197-198 °C; ^{1}H NMR (DMSO-d_6, 400 MHz, TMS) δ 7.75 (d, J = 7.4 Hz, 1H), 7.73-7.64 (m, 1H), 7.63-7.50 (m, 2H), 7.52-7.45 (m, 2H), 7.41 (t, J = 7.8 Hz, 2H), 7.35-7.24 (m, 2H), 5.27 (t, J = 2.5 Hz, 1H), 4.18-4.01 (m, 2H), 2.62-2.38 (m, 2H); ^{13}C NMR ((DMSO-d_6, 100 MHz, TMS) δ 166.6, 155.6, 146.9, 136.6, 133.4, 131.3, 130.3, 129.0, 127.1, 127.0, 123.3, 122.9, 100.1, 89.1, 70.6, 29.9; IR (neat) ν 3256, 2927, 1668, 1494, 1363, 1204, 1086, 1048, 939, 819, 760 cm^{-1}; HRMS (ESI) Calcd. for $C_{18}H_{15}NO_3Na^+$ Requires: 316.0944, Found: 316.0942.

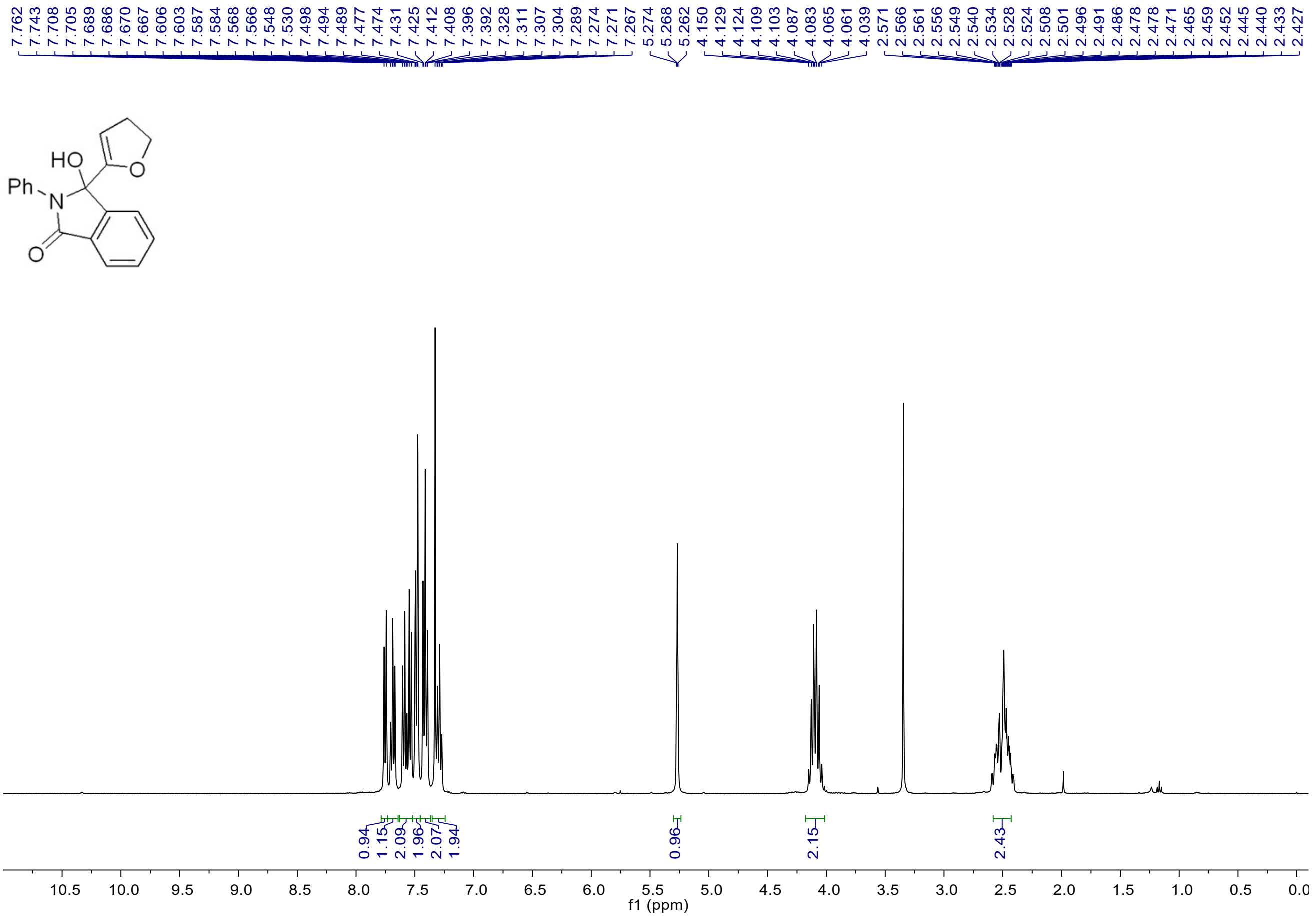
7.762 7.743 7.708 7.705 7.689 7.686 7.670 7.667 7.606 7.603 7.587 7.584 7.568 7.566 7.548 7.530 7.498 7.494 7.489 7.477 7.474 7.431 7.425 7.412 7.408 7.396 7.392 7.328 7.311 7.307 7.304 7.289 7.274 7.271 7.267
5.274 5.268 5.262
4.150 4.129 4.124 4.109 4.103 4.087 4.083 4.065 4.061 4.039
2.571 2.566 2.561 2.556 2.549 2.540 2.534 2.528 2.524 2.508 2.501 2.496 2.491 2.486 2.478 2.471 2.465 2.459 2.452 2.445 2.440 2.433 2.427
HO
Ph
N
O
O
0.94 1.15 2.09 1.96 2.07 1.94
0.96
2.15
2.43
f1 (ppm)

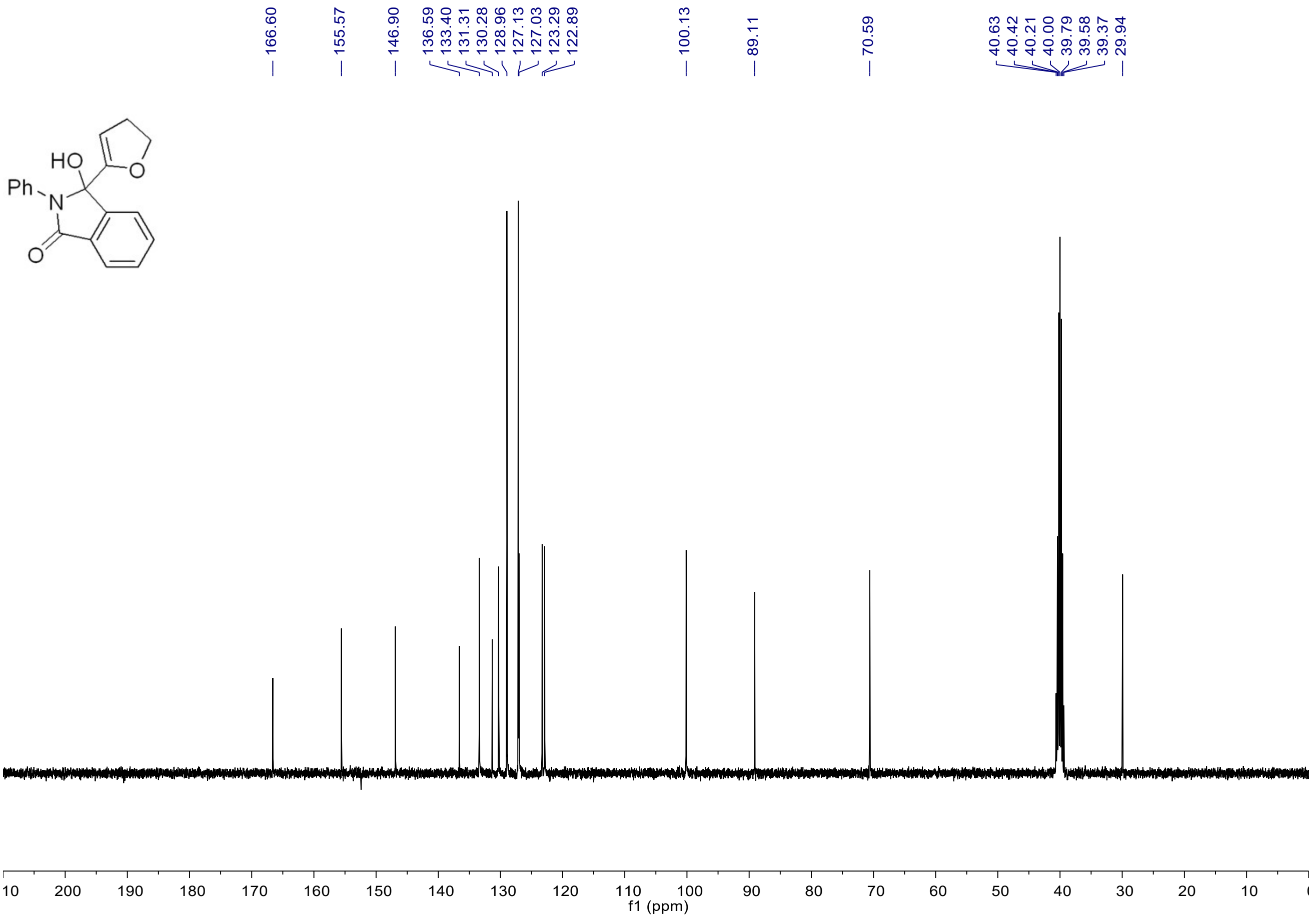
166.60
155.57
146.90
136.59 133.40 131.31 130.28 128.96 127.13 127.03 123.29 122.89
100.13
89.11
70.59
40.63 40.42 40.21 40.00 39.79 39.58 39.37 29.94
HO
Ph
N
O
O
f1 (ppm)

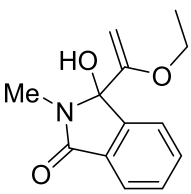

3-(1-ethoxyvinyl)-3-hydroxy-2-methylisoindolin-1-one (3hb). A white solid, 43 mg, 93% yield; M.p.: 119-120 °C; ^{1}H NMR (CD_2Cl_2, 400 MHz, TMS) δ 7.46-7.33 (m, 2H), 7.33-7.22 (m, 2H), 4.77 (d, *J* = 2.3 Hz, 2H), 4.22 (d, *J* = 2.3 Hz, 1H), 3.64-3.47 (m, 2H), 2.52 (s, 3H), 0.95 (t, *J* = 7.0 Hz, 3H); ^{13}C NMR (CD_2Cl_2, 100 MHz, TMS) δ 168.2, 157.6, 147.0, 132.1, 131.1, 129.3, 122.6, 121.8, 89.5, 84.5, 63.7, 23.7, 13.9; IR (neat) ν 3216, 2982, 2933, 1656, 1615, 1478, 1431, 1333, 1248, 1119, 1085, 1033, 937, 872 cm^{-1}; HRMS (ESI) Calcd. for $C_{13}H_{15}NO_3Na^+$ Requires: 256.0944, Found: 256.0941.

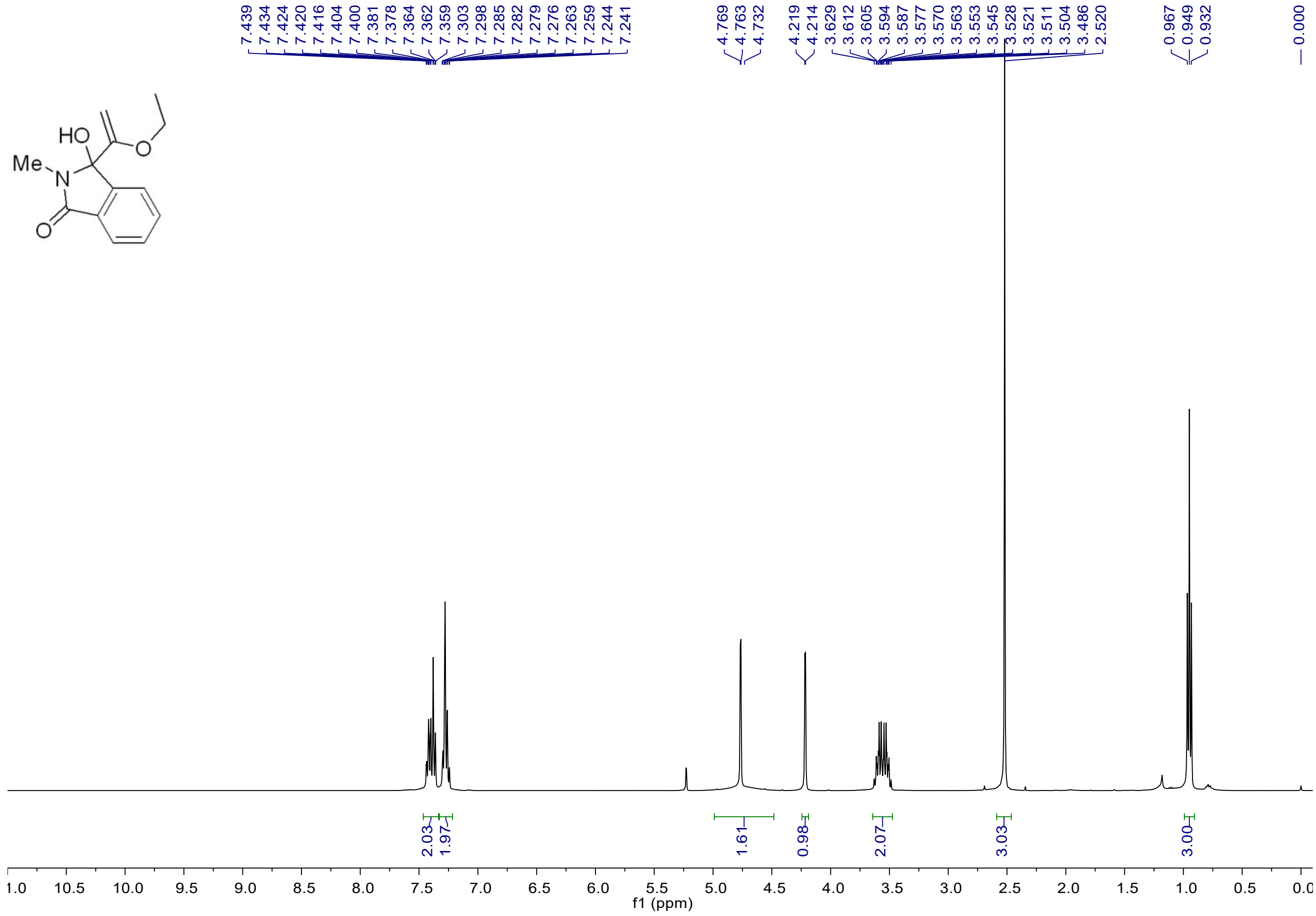

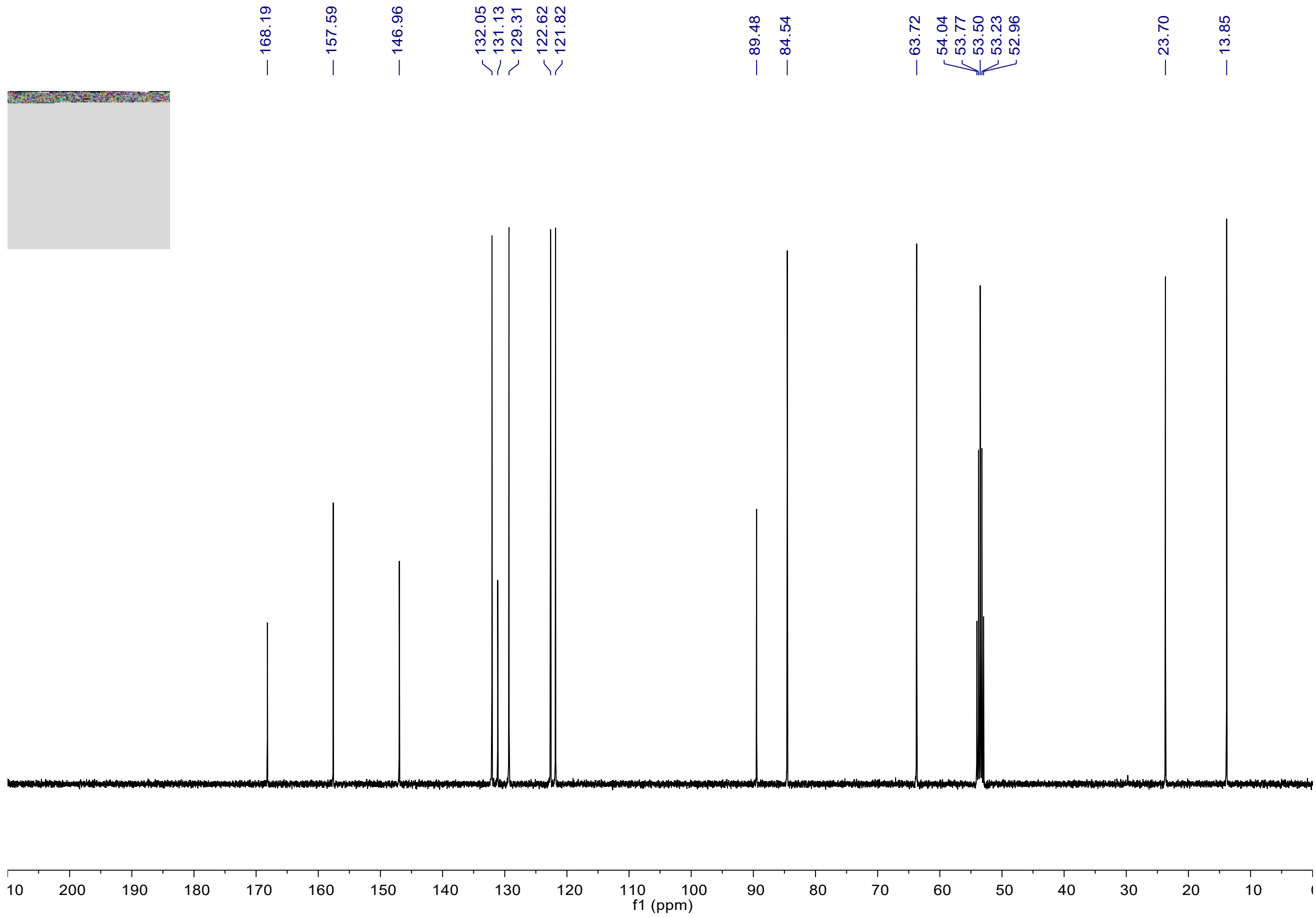

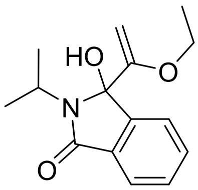

3-(1-ethoxyvinyl)-3-hydroxy-2-isopropylisoindolin-1-one (3hc). A white solid, 52 mg, 99% yield; M.p.: 149-150 °C; ^{1}H NMR (CD_2Cl_2, 400 MHz, TMS) δ 7.41-7.31 (m, 2H), 7.31-7.23 (m, 2H), 4.80 (d, *J* = 2.3 Hz, 1H), 4.26 (s, 1H), 4.22 (d, *J* = 2.3 Hz, 1H), 3.69-3.49 (m, 3H), 1.26 (t, *J* = 6.6 Hz, 6H), 0.98 (t, *J* = 7.0 Hz, 3H); ^{13}C NMR (CD_2Cl_2, 100 MHz, TMS) δ 167.4, 158.6, 146.7, 132.6, 131.7, 129.3, 122.4, 121.5, 90.1, 84.4, 63.6, 44.5, 20.8, 19.4, 13.9; IR (neat) ν 3244, 2980, 2941, 1672, 1634, 1454, 1379, 1345, 1279, 1204, 1122, 1085, 1049, 996, 873, 770 cm^{-1}; HRMS (ESI) Calcd. for $C_{15}H_{19}NO_3Na^+$ Requires: 284.1257, Found: 284.1255.

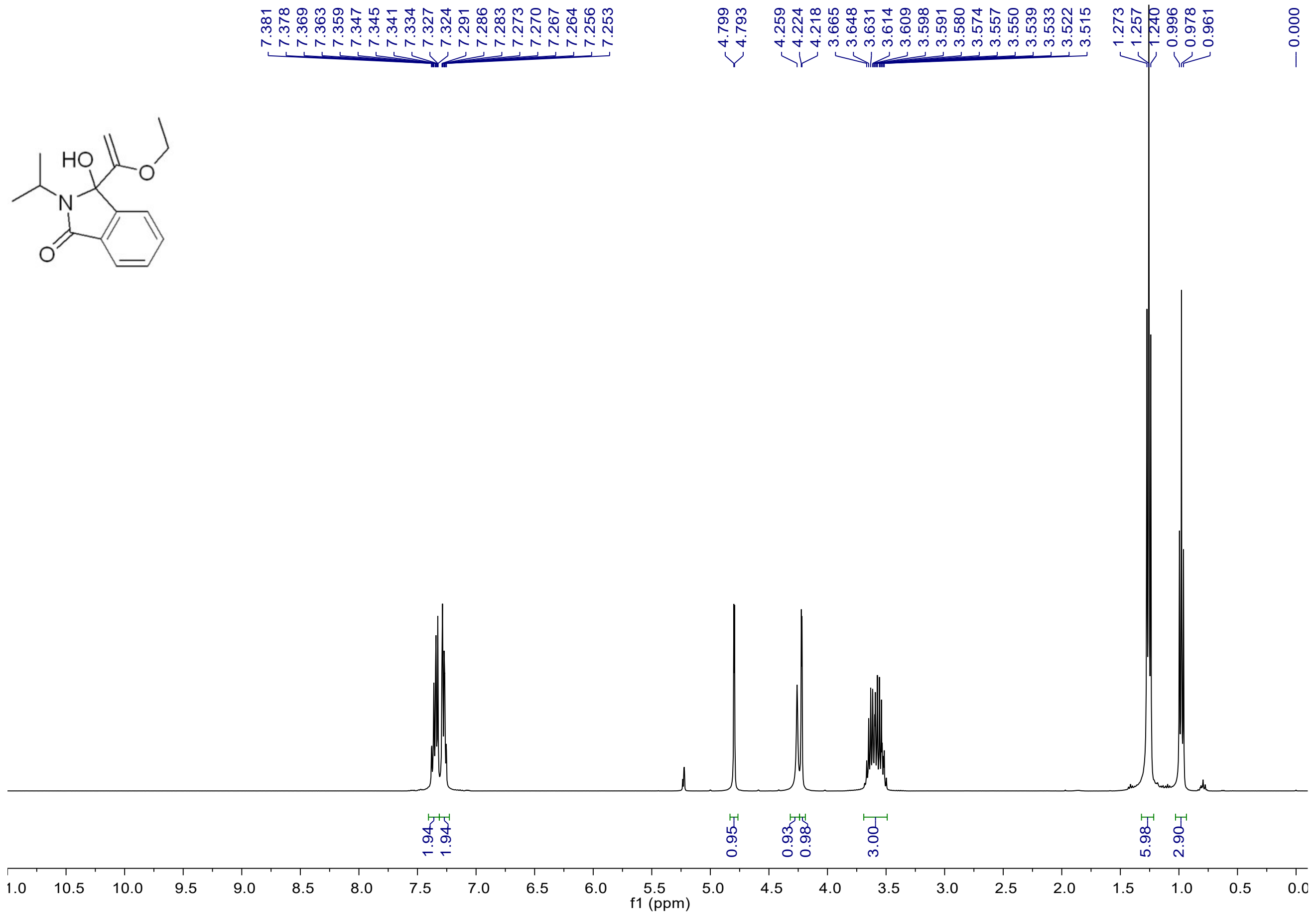
7.381 7.378 7.369 7.363 7.359 7.347 7.345 7.341 7.334 7.327 7.324 7.291 7.286 7.283 7.273 7.270 7.267 7.264 7.256 7.253
4.799 4.793
4.259 4.224 4.218 3.665 3.648 3.631 3.614 3.609 3.598 3.591 3.580 3.574 3.557 3.550 3.539 3.533 3.522 3.515
1.273 1.257 1.240 0.996 0.978 0.961
0.000
HO
N
O
1.94 1.94 0.95 0.93 0.98 3.00 5.98 2.90
f1 (ppm)

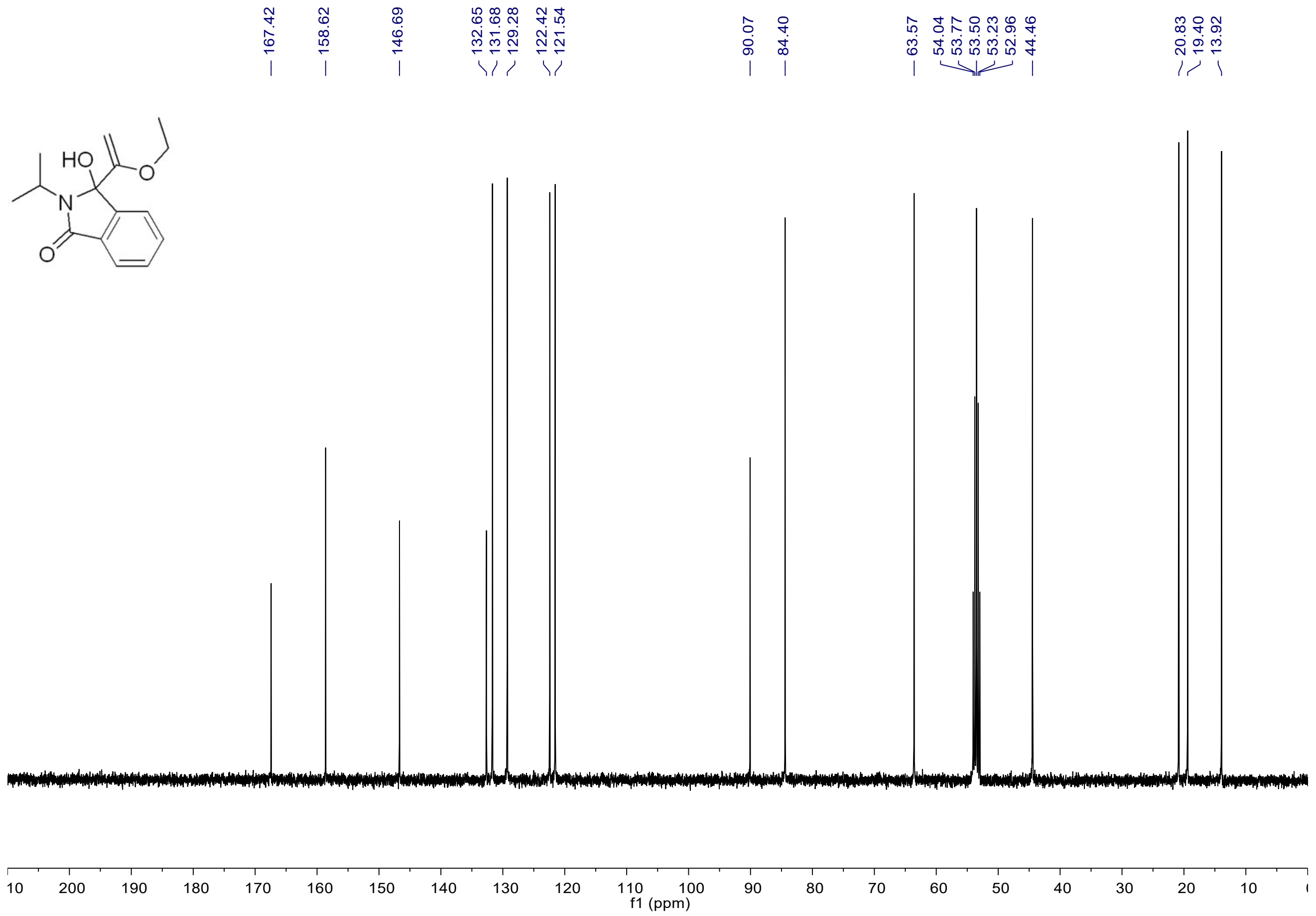
167.42 158.62 146.69 132.65 131.68 129.28 122.42 121.54 90.07 84.40 63.57 54.04 53.77 53.50 53.23 52.96 44.46 20.83 19.40 13.92
HO
N
O
f1 (ppm)

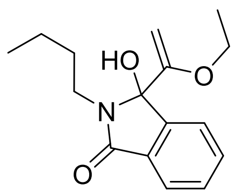

2-butyl-3-(1-ethoxyvinyl)-3-hydroxyisoindolin-1-one (3hd). A white solid, 54 mg, 98% yield; M.p.: 119-120 °C; ^{1}H NMR (CD_2Cl_2, 400 MHz, TMS) δ 7.48-7.25 (m, 4H), 4.80-4.74 (m, 1H), 4.50-4.03 (m, 2H), 3.62-3.49 (m, 2H), 3.26-3.14 (m, 1H), 2.97-2.85 (m, 1H), 1.52-1.30 (m, 2H), 1.24-1.14 (m, 2H), 0.96 (t, J = 6.9 Hz, 3H), 0.80 (t, J = 7.3 Hz, 3H); ^{13}C NMR (CD_2Cl_2, 100 MHz, TMS) δ 168.1, 158.3, 147.0, 132.0, 131.5, 129.3, 122.7, 121.7, 89.6, 84.3, 63.6, 39.0, 30.7, 20.6, 13.9, 13.6; IR (neat) ν 3240, 2966, 2933, 2869, 1671, 1626, 1435, 1382, 1276, 1216, 1084, 977, 812, 776 cm^{-1}; HRMS (ESI) Calcd. for $C_{16}H_{21}NO_3Na^+$ Requires: 298.1414, Found: 298.1409.

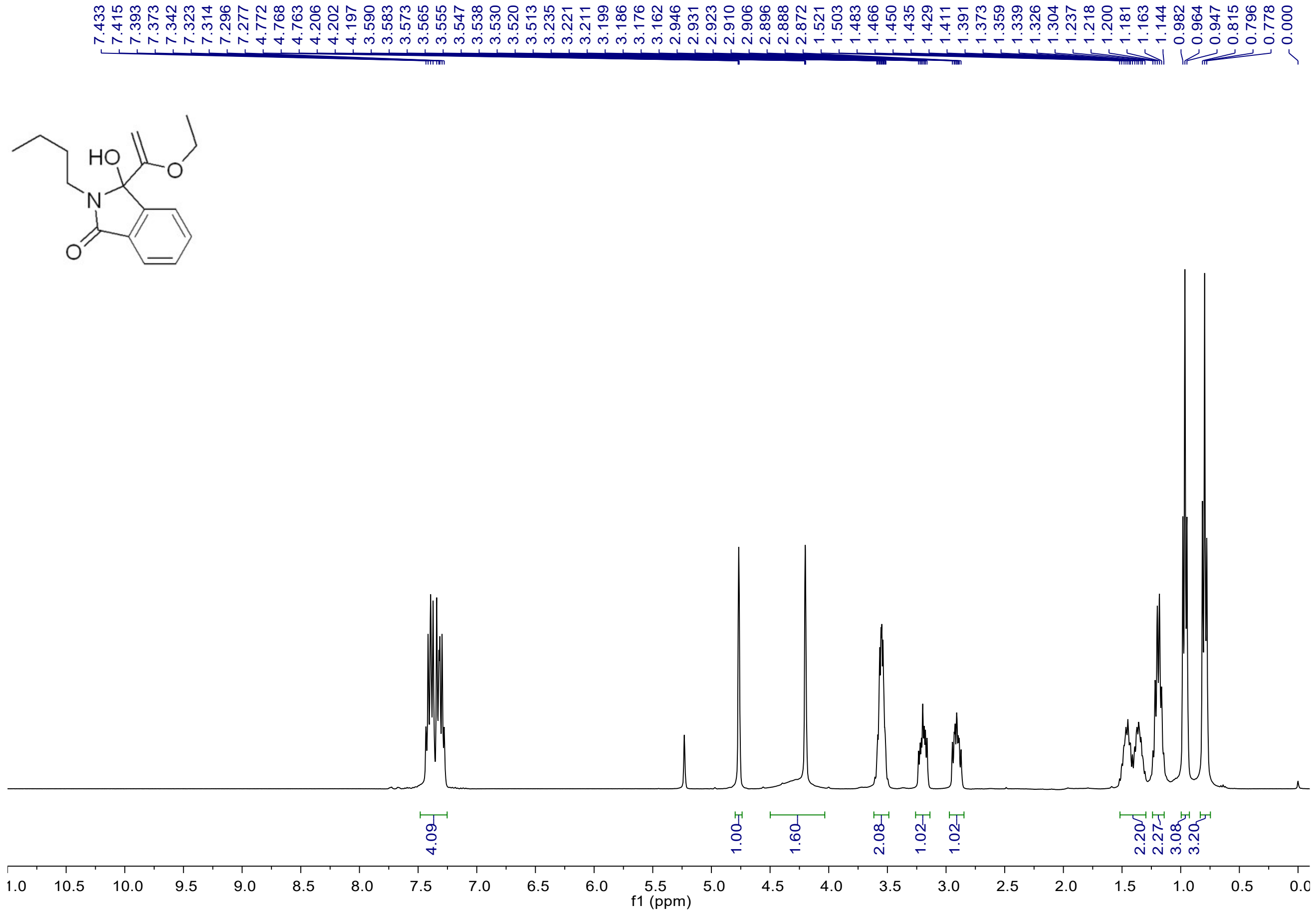

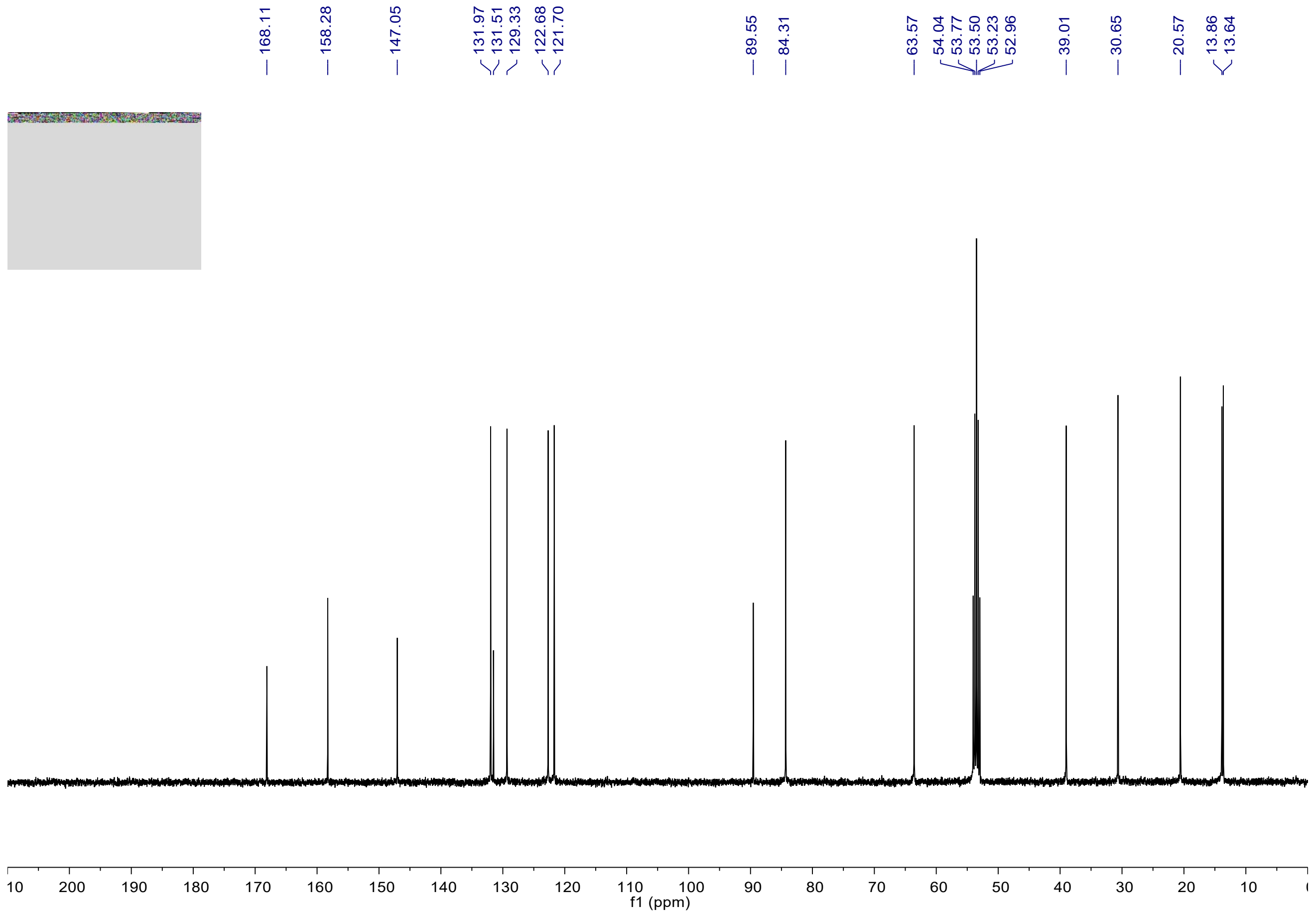

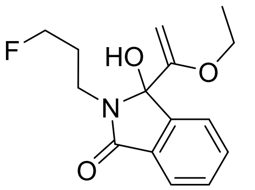

3-(1-ethoxyvinyl)-2-(3-fluoropropyl)-3-hydroxyisoindolin-1-one (3he). A white solid, 55 mg, 98% yield; M.p.: 103-105 ^{o}C; ^{1}H NMR (DMSO-d_6, 400 MHz) δ 8.12-8.06 (m, 1H), 8.04 (td, J = 7.4, 1.3 Hz, 1H), 8.00-7.88 (m, 2H), 6.20 (s, 1H), 5.50 (d, J = 2.0 Hz, 1H), 5.05 (td, J = 6.2, 1.7 Hz, 1H), 4.93 (td, J = 6.2, 1.7 Hz, 1H), 4.89 (d, J = 2.0 Hz, 1H), 4.23-4.01 (m, 3H), 3.81 (dt, J = 14.3, 7.1 Hz, 1H), 2.60-2.44 (m, 2H), 1.47 (t, J = 7.0 Hz, 3H); ^{13}C NMR (Acetone-d_6, 100 MHz) δ 167.4, 158.8, 147.3, 132.0, 131.8, 129.2, 122.2, 121.7, 89.3, 84.3 (d, J = 3.5 Hz), 82.2 (d, J = 162.4 Hz), 63.2, 35.1 (d, J = 7.0 Hz), 29.8 (d, J = 19.5 Hz), 13.5; ^{19}F NMR (376 MHz, Acetone-d_6) δ -220.8 (m, 1F). IR (neat) ν 3256, 2983, 2897, 1670, 1609, 1470, 1405, 13801278, 1121, 1080, 1052, 977, 909, 821, 770, 702 cm^{-1}; HRMS (ESI) Calcd. for $C_{15}H_{18}NO_3FNa^+$ Requires: 302.1163, Found: 302.1162.

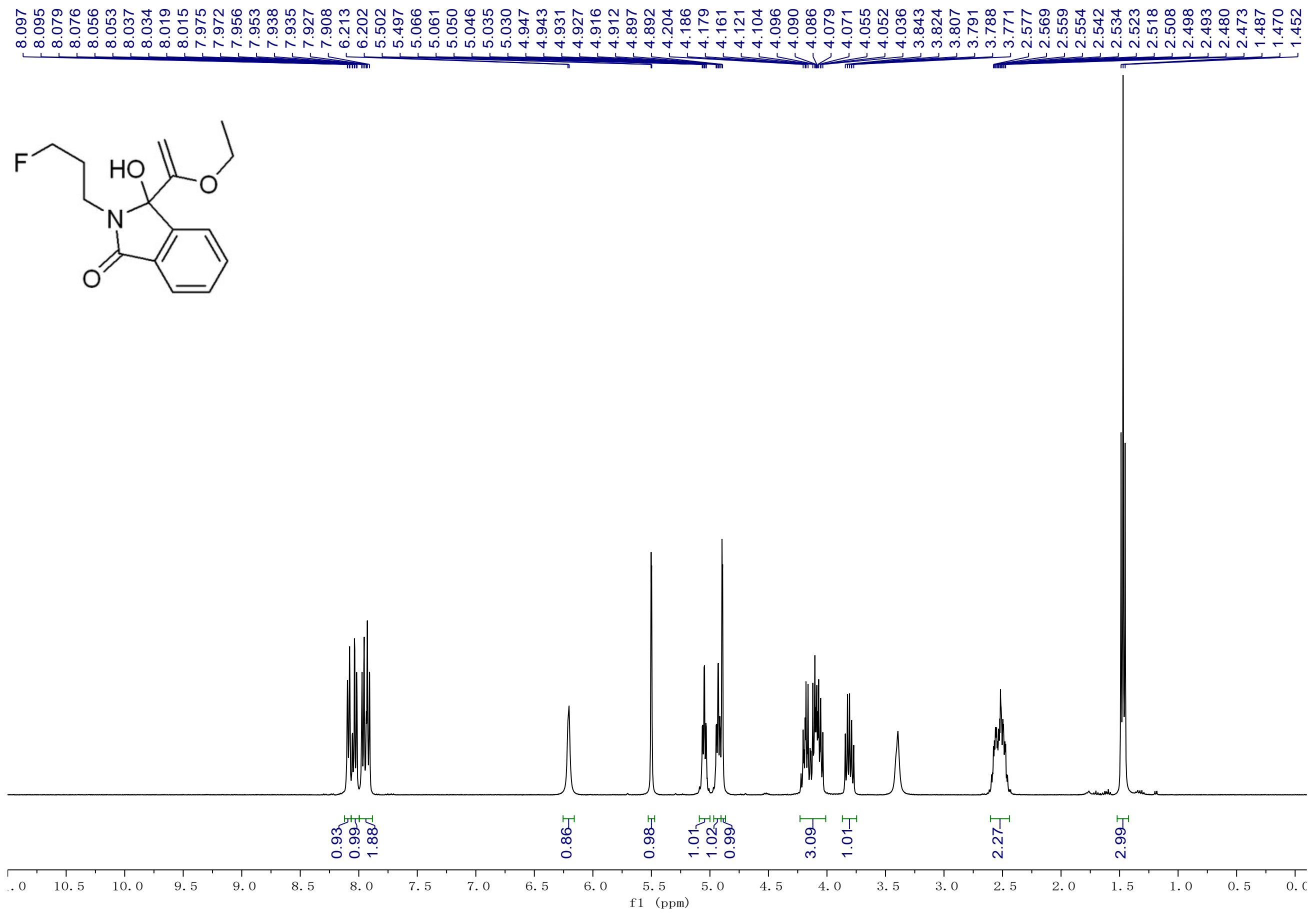

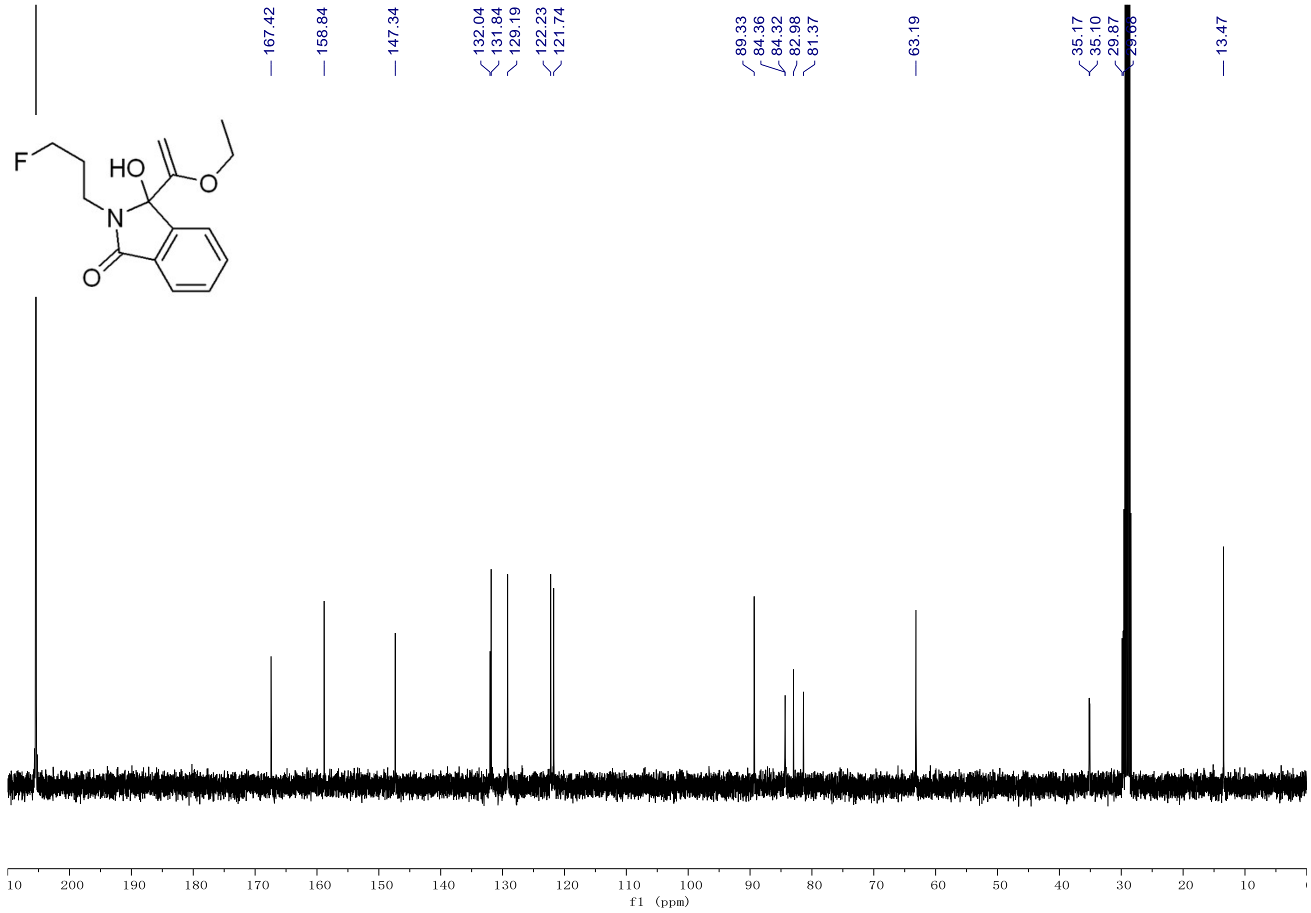

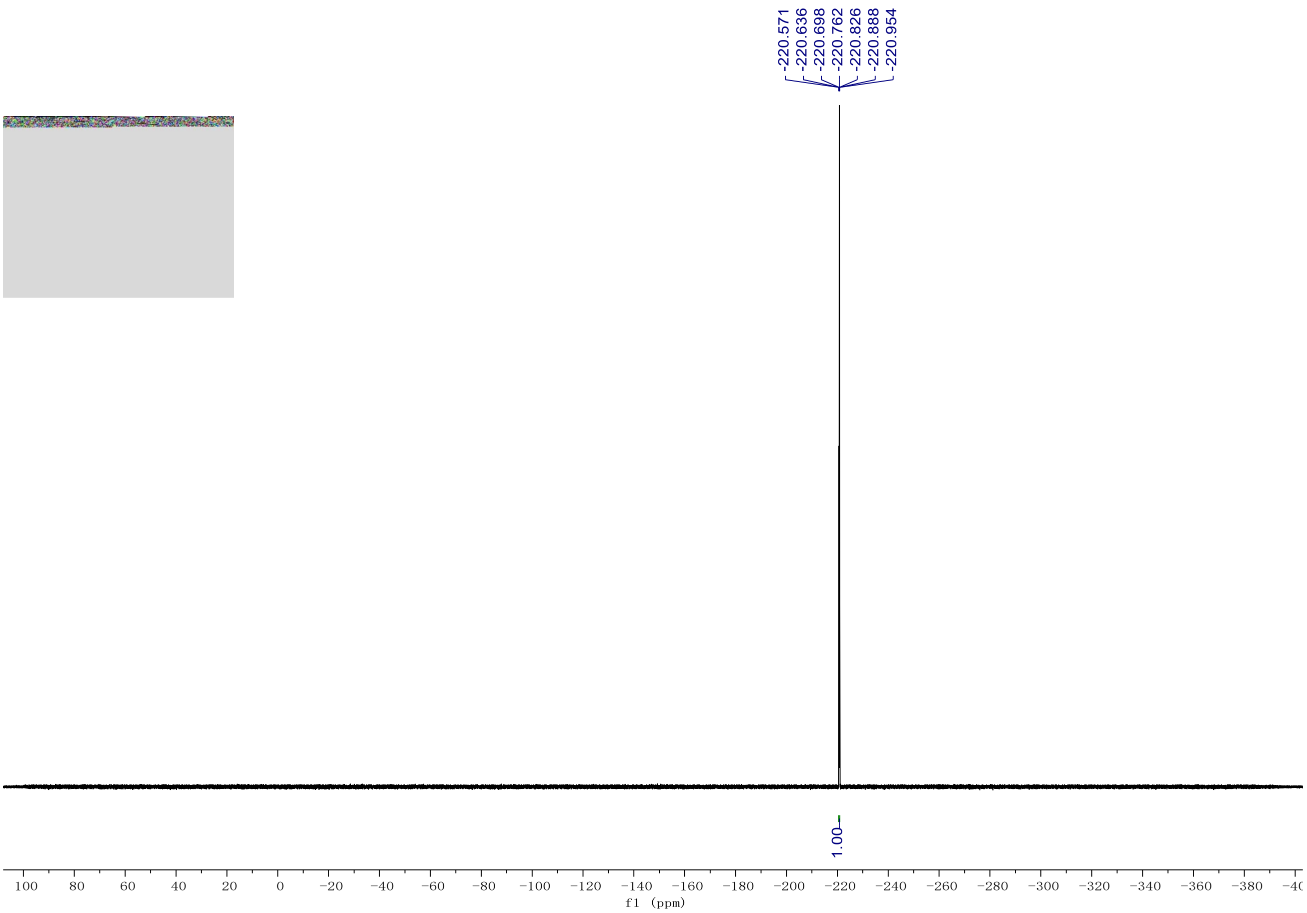

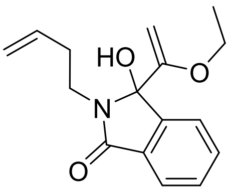

2-(but-3-en-1-yl)-3-(1-ethoxyvinyl)-3-hydroxyisoindolin-1-one (3hf). A white solid, 54 mg, 99% yield; M.p.: 108-109 °C; ^{1}H NMR (CD_2Cl_2, 400 MHz, TMS) δ 7.46-7.25 (m, 4H), 5.77-5.62 (m, 1H), 4.99-4.86 (m, 2H), 4.79 (d, J = 2.4 Hz, 1H), 4.31 (s, 1H), 4.22 (d, J = 2.4 Hz, 1H), 3.64-3.47 (m, 2H), 3.37-3.24 (m, 1H), 3.03-2.91 (m, 1H), 2.34-2.09 (m, 2H), 0.96 (t, J = 7.0 Hz, 3H); ^{13}C NMR (CD_2Cl_2, 100 MHz, TMS) δ 168.1, 158.1, 147.0, 136.2, 132.1, 131.4, 129.4, 122.7, 121.8, 115.8, 89.6, 84.6, 63.6, 38.7, 32.7, 13.9; IR (neat) ν 3242, 2984, 2939, 1671, 1631, 1470, 1403, 1323, 1275, 1155, 1081, 978, 910, 818, 701 cm^{-1}; HRMS (ESI) Calcd. for $C_{16}H_{19}NO_3Na^+$ Requires: 296.1257, Found: 296.1257.

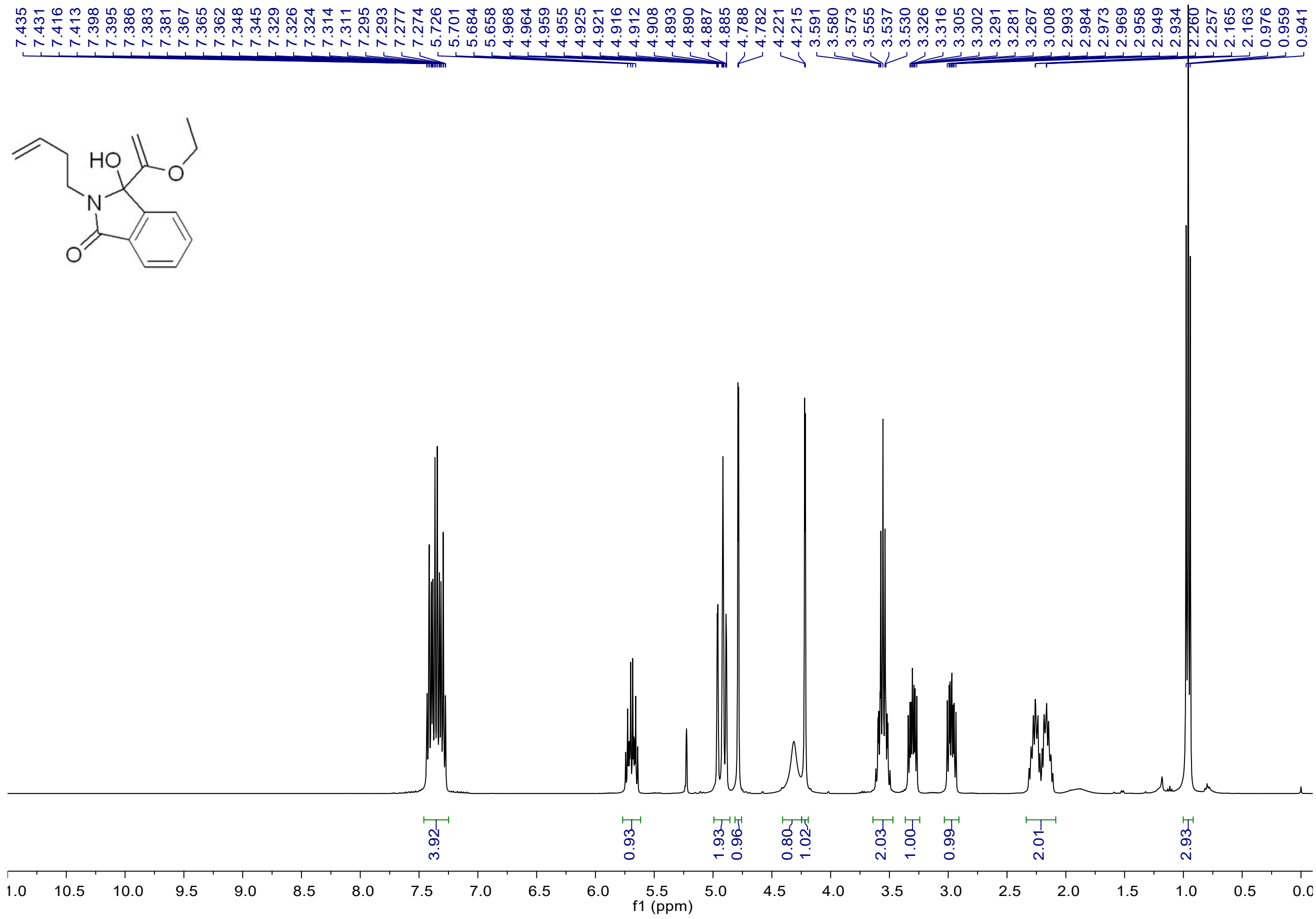
7.435
7.431
7.416
7.413
7.398
7.395
7.386
7.383
7.381
7.367
7.365
7.362
7.348
7.345
7.329
7.326
7.324
7.314
7.311
7.295
7.293
7.277
7.274
5.726
5.701
5.684
5.658
4.968
4.964
4.959
4.955
4.925
4.921
4.916
4.912
4.908
4.893
4.890
4.887
4.885
4.788
4.782
4.221
4.215
3.591
3.580
3.573
3.555
3.537
3.530
3.326
3.316
3.305
3.302
3.291
3.281
3.267
3.008
2.993
2.984
2.973
2.969
2.958
2.949
2.934
2.260
2.257
2.165
2.163
0.976
0.959
0.941
HO
N
O
O
3.92
0.93
1.93
0.96
0.80
1.02
2.03
1.00
0.99
2.01
2.93
f1 (ppm)

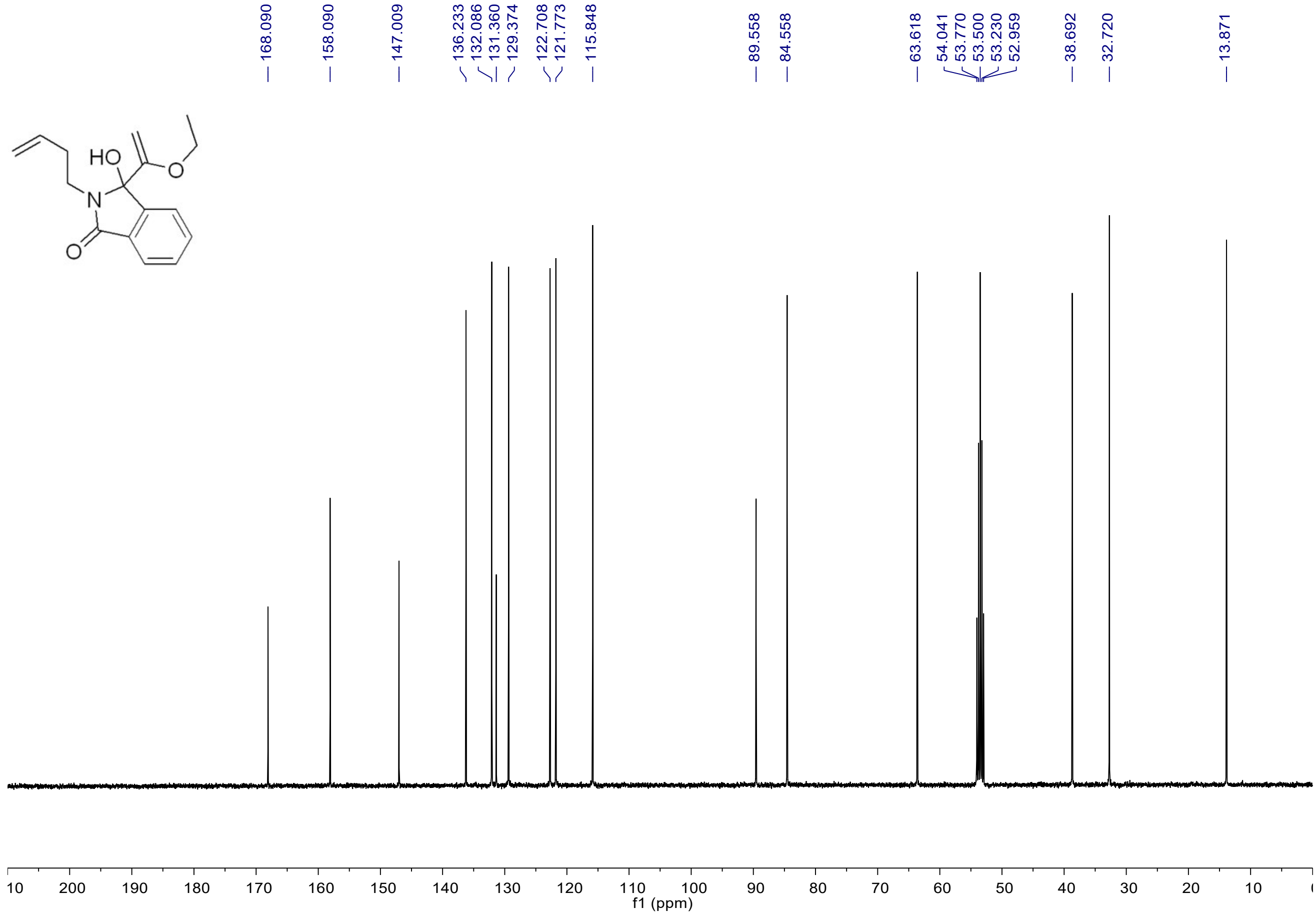
168.090
158.090
147.009
136.233
132.086
131.360
129.374
122.708
121.773
115.848
89.558
84.558
63.618
54.041
53.770
53.500
53.230
52.959
38.692
32.720
13.871
HO
N
O
O
f1 (ppm)

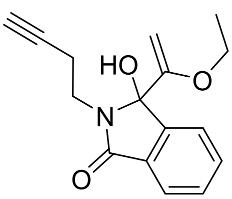

2-(but-3-yn-1-yl)-3-(1-ethoxyvinyl)-3-hydroxyisoindolin-1-one (3hg). A white solid, 52 mg, 96% yield; M.p.: 104-105 °C; ^{1}H NMR (CD_2Cl_2, 400 MHz, TMS) δ 7.62-7.51 (m, 2H), 7.51-7.41 (m, 2H), 4.94 (d, *J* = 2.5 Hz, 1H), 4.44 (s, 1H), 4.38 (d, *J* = 2.4 Hz, 1H), 3.79-3.64 (m, 2H), 3.64-3.54 (m, 1H), 3.31-3.19 (m, 1H), 2.65-2.51 (m, 1H), 2.50-2.36 (m, 1H), 2.11-2.04 (m, 1H), 1.10 (t, *J* = 7.0 Hz, 3H); ^{13}C NMR (CD_2Cl_2, 100 MHz, TMS) δ 168.1, 157.8, 146.9, 132.4, 131.0, 129.5, 122.8, 121.8, 89.5, 84.9, 82.1, 69.5, 63.7, 38.2, 18.2, 13.9; IR (neat) ν 3302, 3241, 2989, 2944, 1667, 1614, 1441, 1374, 1289, 1244, 1173, 1131, 1081, 954, 809 cm^{-1}; HRMS (ESI) Calcd. for $C_{16}H_{17}NO_3Na^+$ Requires: 294.1101, Found: 294.1099.

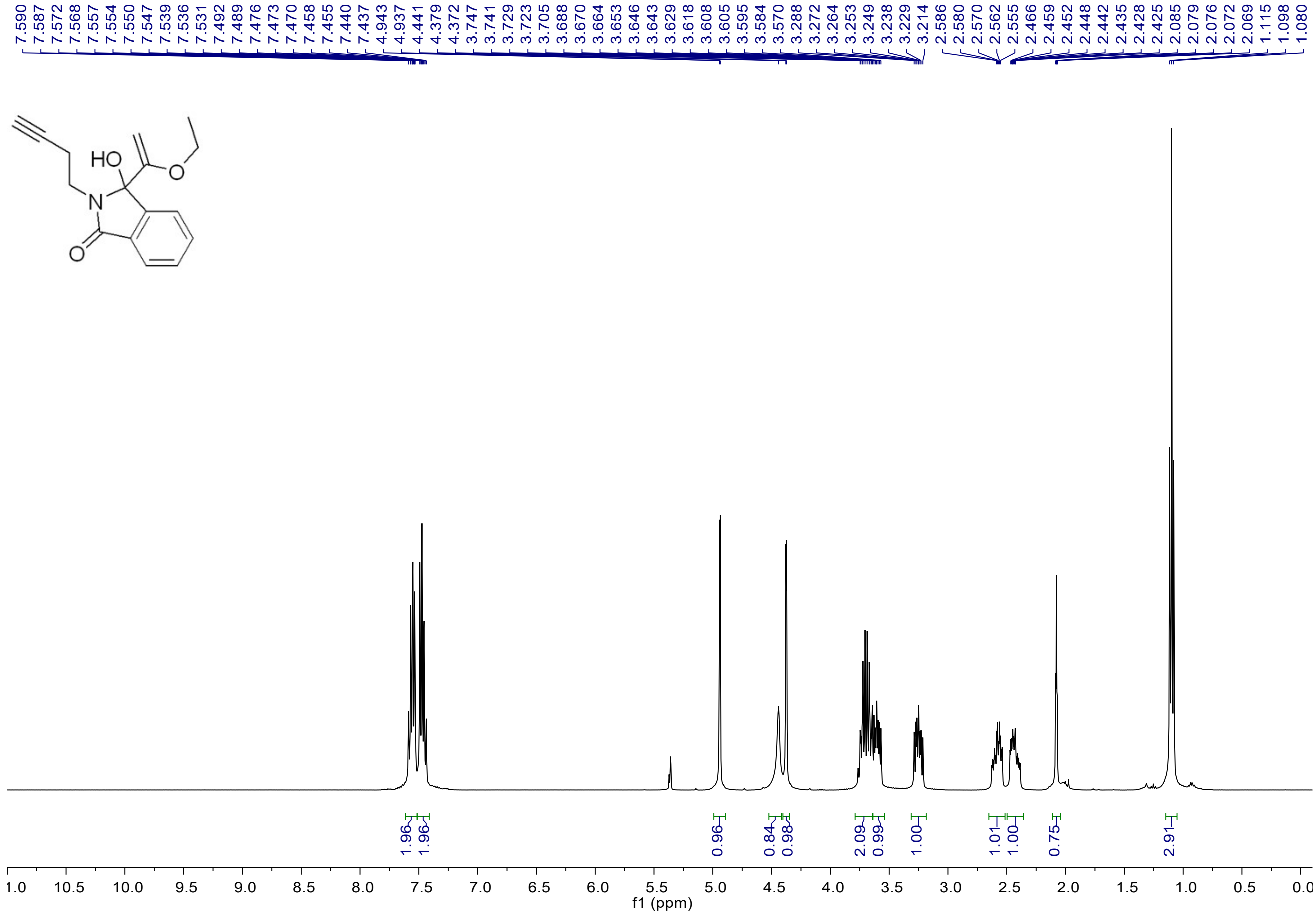

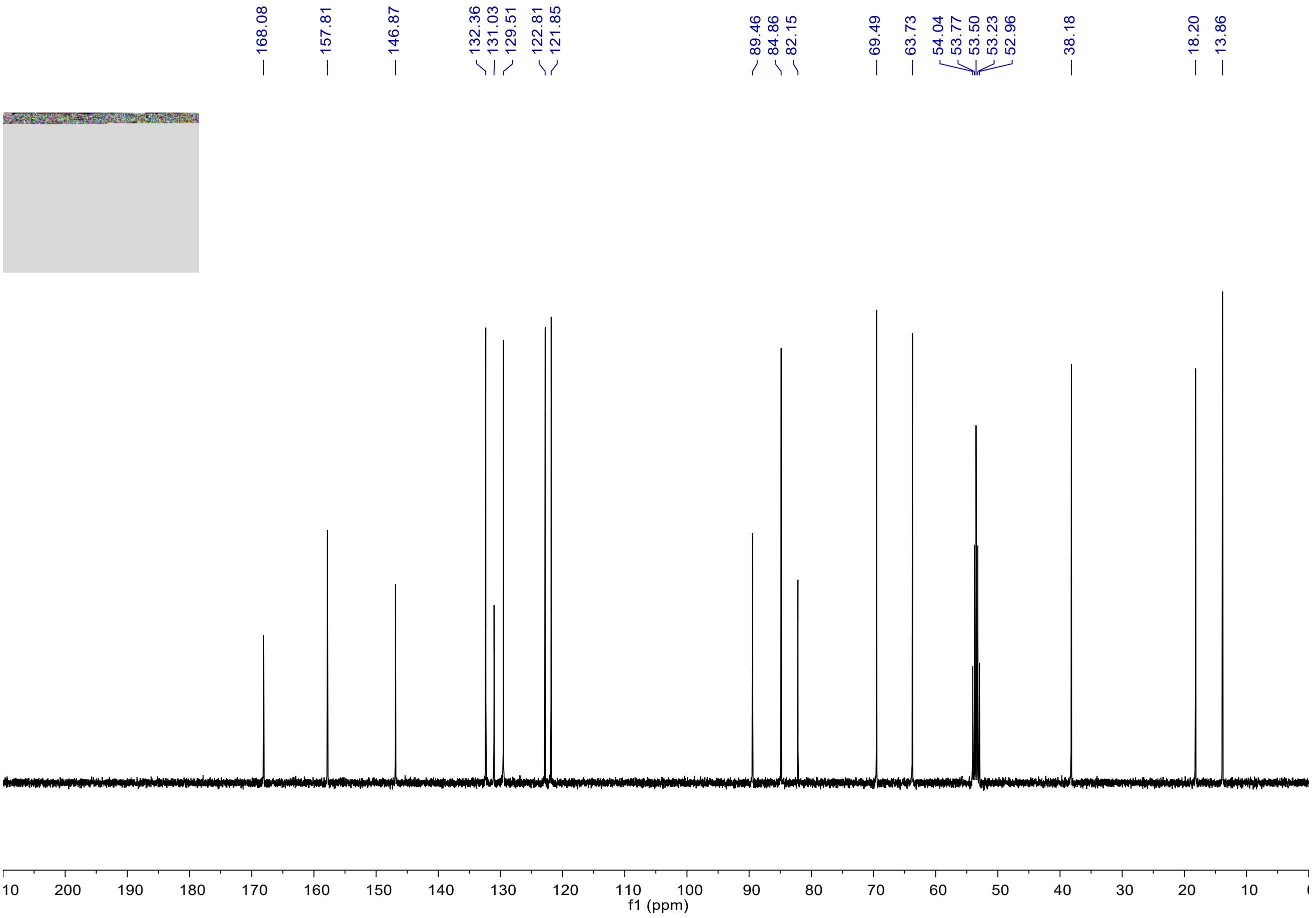

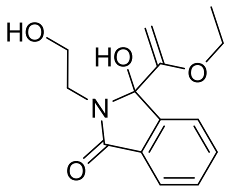

3-(1-ethoxyvinyl)-3-hydroxy-2-(2-hydroxyethyl) isoindolin-1-one (3hi). A white solid, 47 mg, 89% yield; M.p.: 43-44 ^{o}C; ^{1}H NMR (CD_2Cl_2, 400 MHz, TMS) δ 7.50-7.39 (m, 2H), 7.36-7.28 (m, 2H), 4.84 (d, J = 2.4 Hz, 1H), 4.28 (d, J = 2.5 Hz, 1H), 3.73-3.64 (m, 1H), 3.64-3.45 (m, 4H), 3.07-2.96 (m, 1H), 0.94 (t, J = 7.1 Hz, 3H); ^{13}C NMR (CD_2Cl_2, 100 MHz, TMS) δ 169.1, 158.1, 146.8, 132.4, 130.9, 122.8, 121.8, 89.4, 85.4, 63.8, 61.3, 42.2, 13.8; IR (neat) ν 3432, 3260, 2977, 2926, 1671, 1632, 1476, 1438, 1402, 1314, 1278, 1130, 1086, 1004, 951 cm^{-1}; HRMS (ESI) Calcd. for $C_{14}H_{17}NO_4Na^+$ Requires: 286.1050, Found: 286.1051.

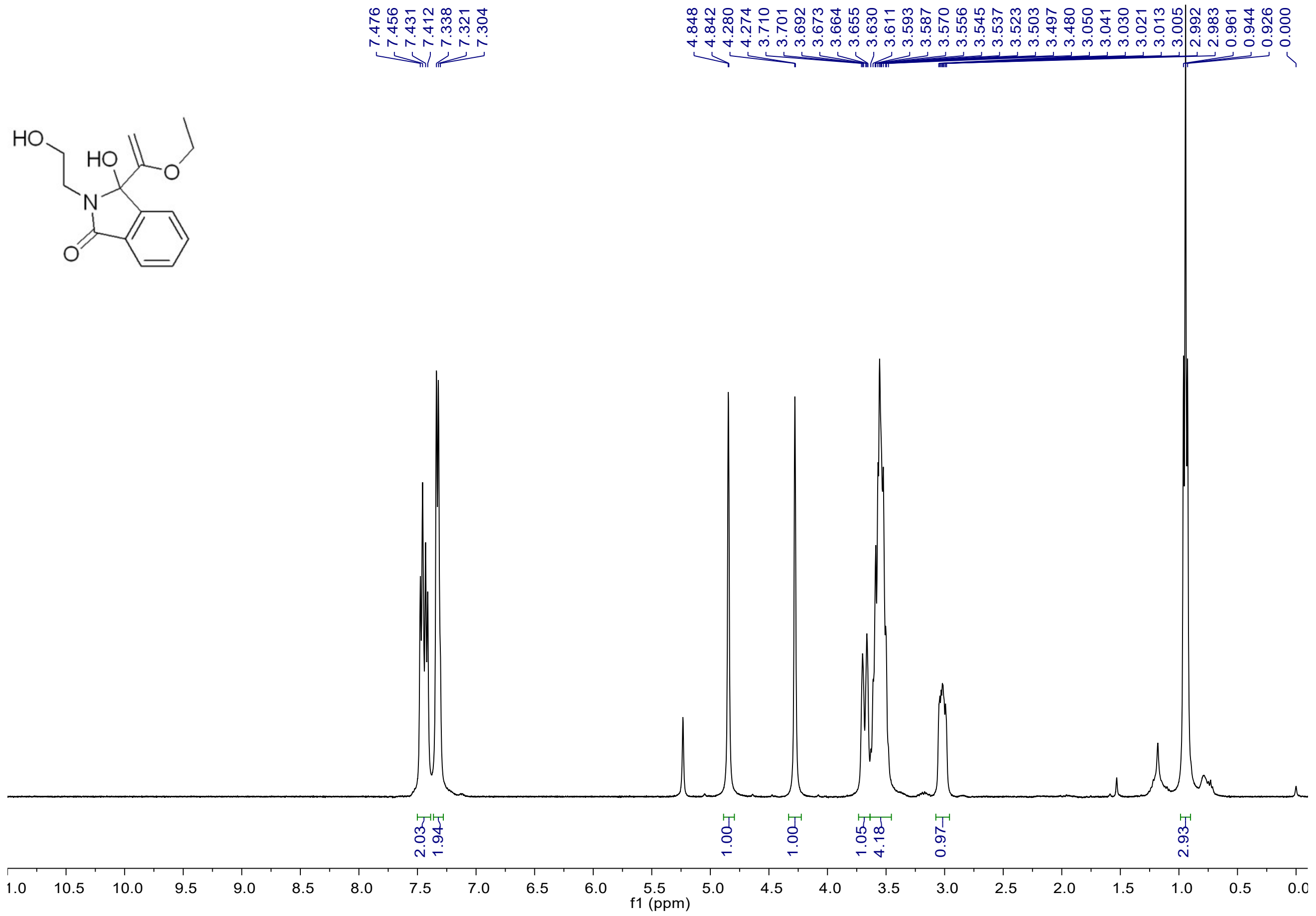

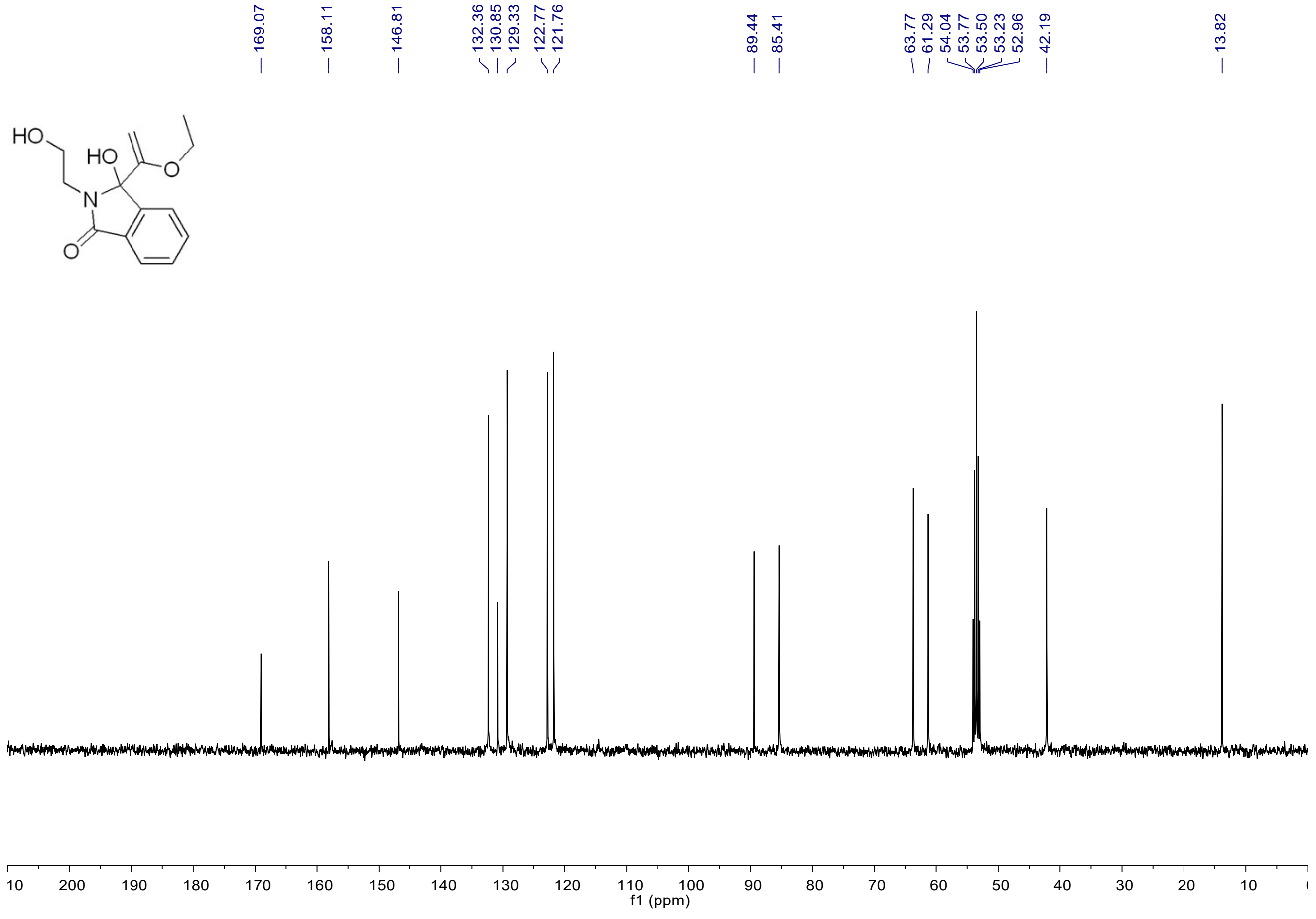

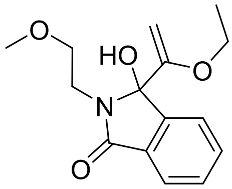

3-(1-ethoxyvinyl)-3-hydroxy-2-(2-methoxyethyl) isoindolin-1-one (3hi). A white solid, 55 mg, quant. yield; M.p.: 60-61 ^{o}C; ^{1}H NMR (CD_2Cl_2, 400 MHz, TMS) δ 7.69 (d, J = 7.4 Hz, 1H), 7.59-7.49 (m, 1H), 7.51-7.39 (m, 2H), 5.66 (d, J = 1.2 Hz, 1H), 5.02-4.96 (m, 1H), 4.40 (d, J = 2.3 Hz, 1H), 4.11-4.01 (m, 1H), 3.75-3.57 (m, 2H), 3.62-3.49 (m, 3H), 3.40 (s, 3H), 3.16-3.02 (m, 1H), 1.04 (t, J = 7.0 Hz, 3H); ^{13}C NMR (CD_2Cl_2, 100 MHz, TMS) δ 167.9, 159.2, 146.8, 132.1, 131.2, 129.1, 122.7, 121.7, 88.8, 85.5, 71.7, 63.8, 58.8, 39.3, 13.8; IR (neat) ν 3246, 2983, 2883, 1678, 1639, 1603, 1473, 1402, 1356, 1320, 1197, 1120, 1086, 1002, 871 cm^{-1}; HRMS (ESI) Calcd. for $C_{15}H_{19}NO_4Na^+$ Requires: 300.1206, Found: 300.1206.

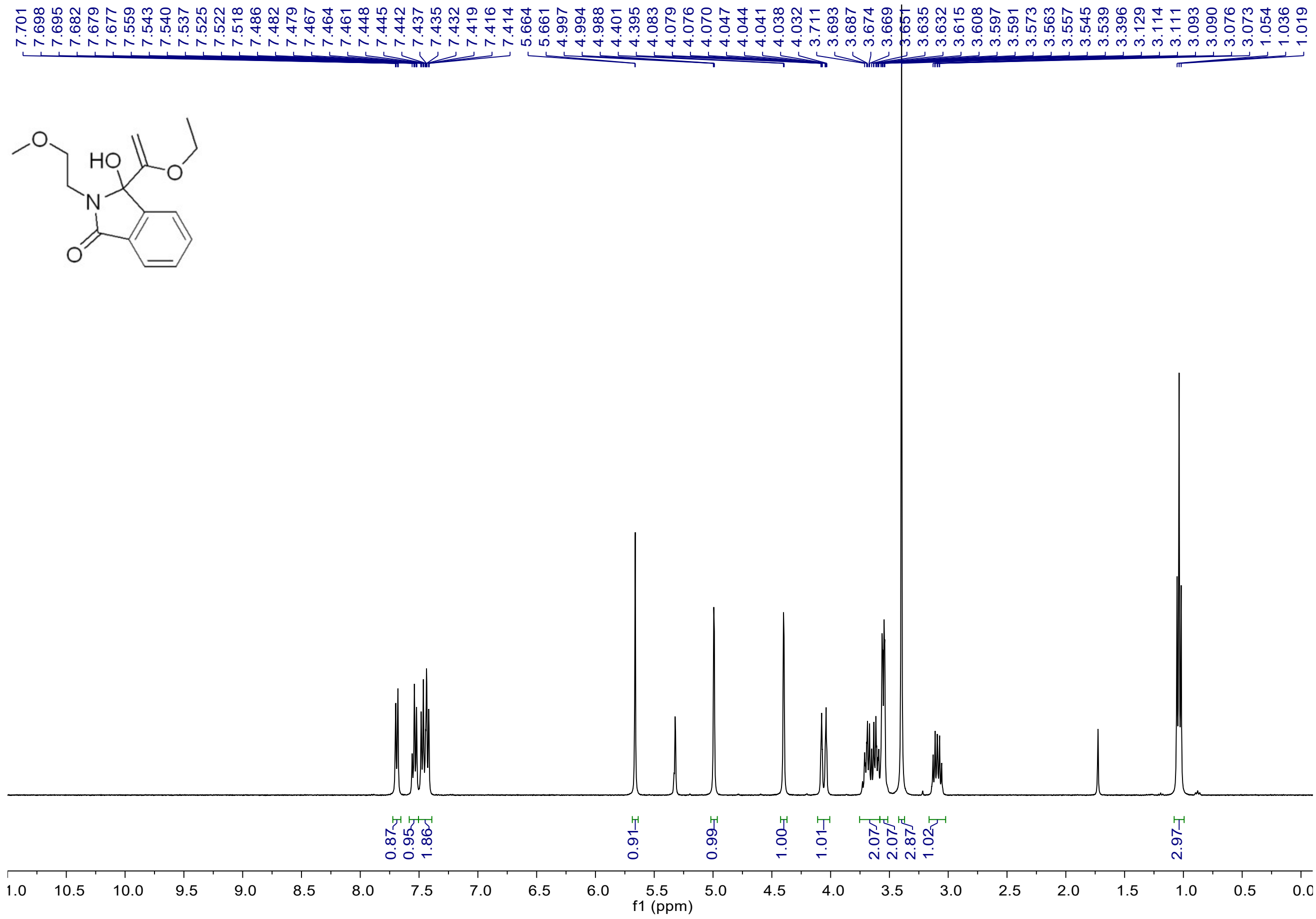

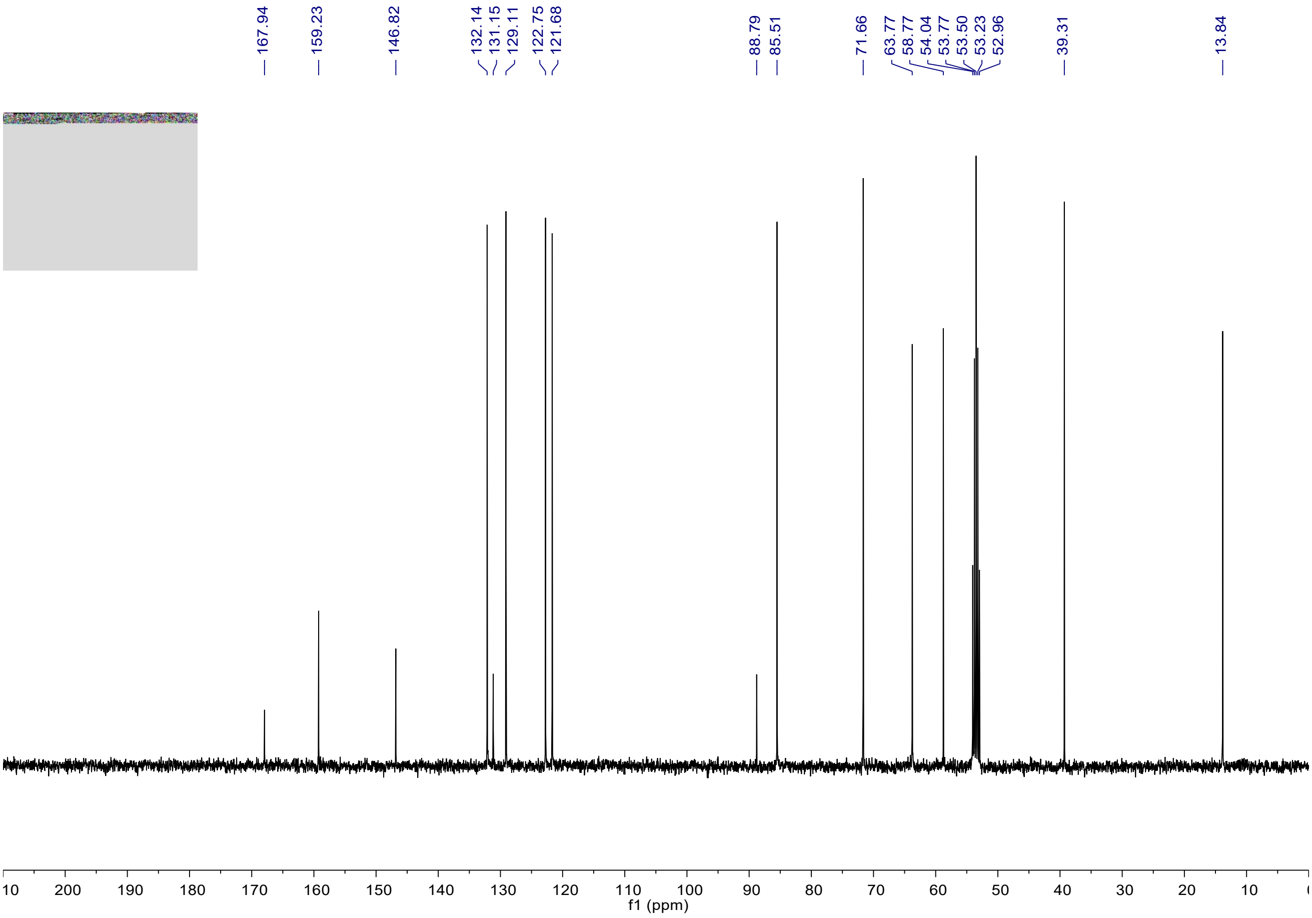

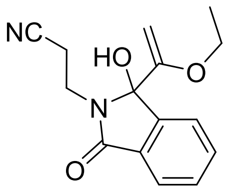

3-(1-(1-ethoxyvinyl)-1-hydroxy-3-oxoisoindolin-2-yl) propanenitrile (3hj). A white solid, 54 mg, 99% yield; M.p.: 136-138 °C; ^{1}H NMR (CD_2Cl_2, 400 MHz, TMS) δ 7.52-7.43 (m, 2H), 7.42-7.33 (m, 2H), 4.85 (d, J = 2.6 Hz, 1H), 4.59 (s, 1H), 4.28 (d, J = 2.5 Hz, 1H), 3.66-3.49 (m, 3H), 3.31-3.19 (m, 1H), 2.67-2.55 (m, 1H), 2.55-2.44 (m, 1H), 0.96 (t, J = 7.0 Hz, 3H); ^{13}C NMR (CD_2Cl_2, 100 MHz, TMS) δ 168.3, 157.3, 146.7, 132.7, 130.7, 129.7, 122.9, 122.0, 118.2, 89.6, 85.3, 63.8, 35.1, 17.0, 13.8; IR (neat) ν 3275, 2980, 2935, 2251, 1678, 1636, 1471, 1422, 1398, 1323, 1275, 1219, 1159, 1122, 952 cm^{-1}; HRMS (ESI) Calcd. for $C_{15}H_{16}N_2O_3Na^+$ Requires: 295.1053, Found: 295.1053.

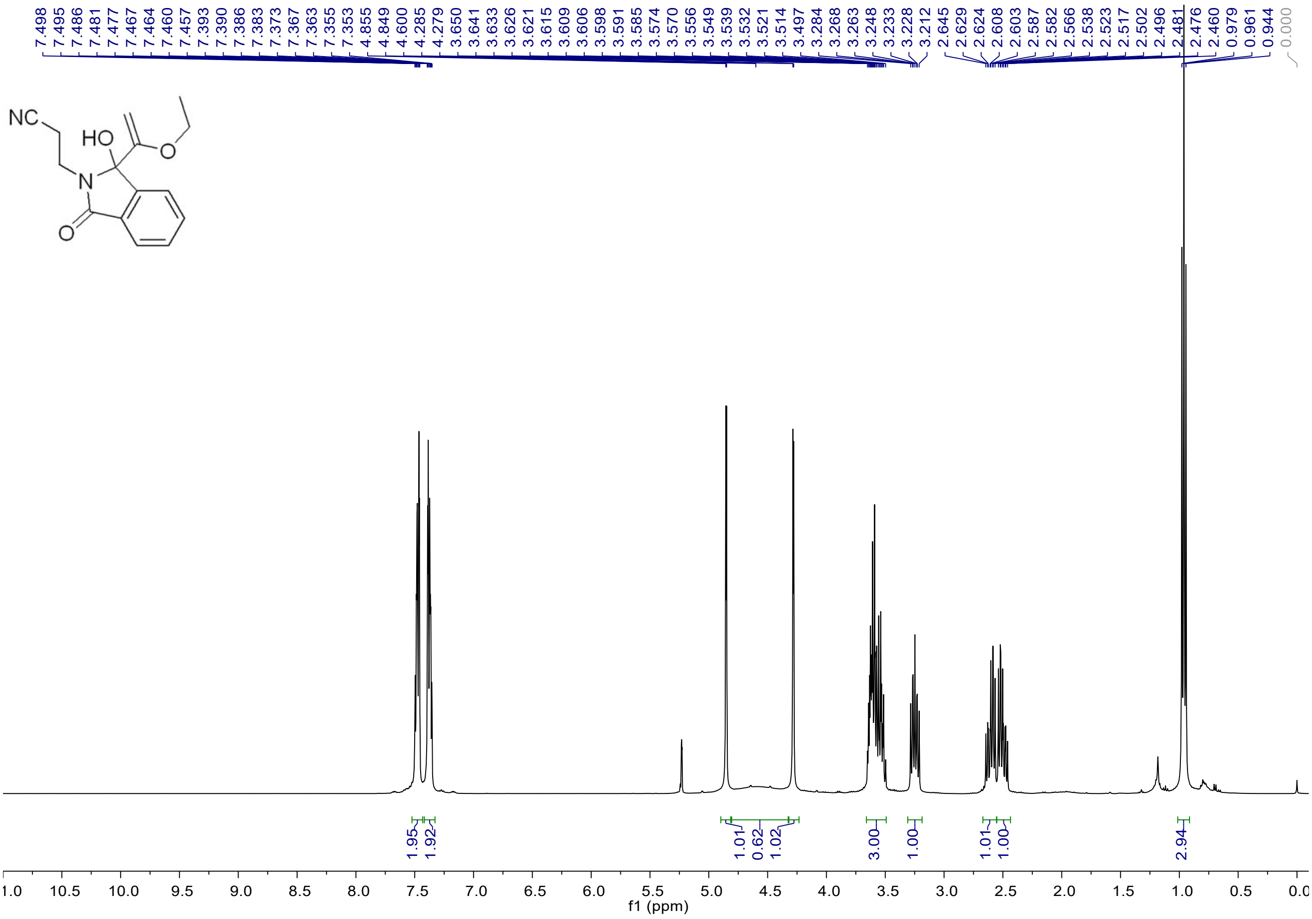
7.498 7.495 7.486 7.481 7.477 7.467 7.464 7.460 7.457 7.393 7.390 7.386 7.383 7.373 7.367 7.363 7.355 7.353 4.855 4.849 4.600 4.285 4.279 3.650 3.641 3.633 3.626 3.621 3.615 3.609 3.606 3.598 3.591 3.585 3.574 3.570 3.556 3.549 3.539 3.532 3.521 3.514 3.497 3.284 3.268 3.263 3.248 3.233 3.228 3.212 2.645 2.629 2.624 2.608 2.603 2.587 2.582 2.566 2.538 2.523 2.517 2.502 2.496 2.481 2.476 2.460 0.979 0.961 0.944 0.000
NC
HO
N
O
1.95
1.92
1.01
0.62
1.02
3.00
1.00
1.01
1.00
2.94
f1 (ppm)
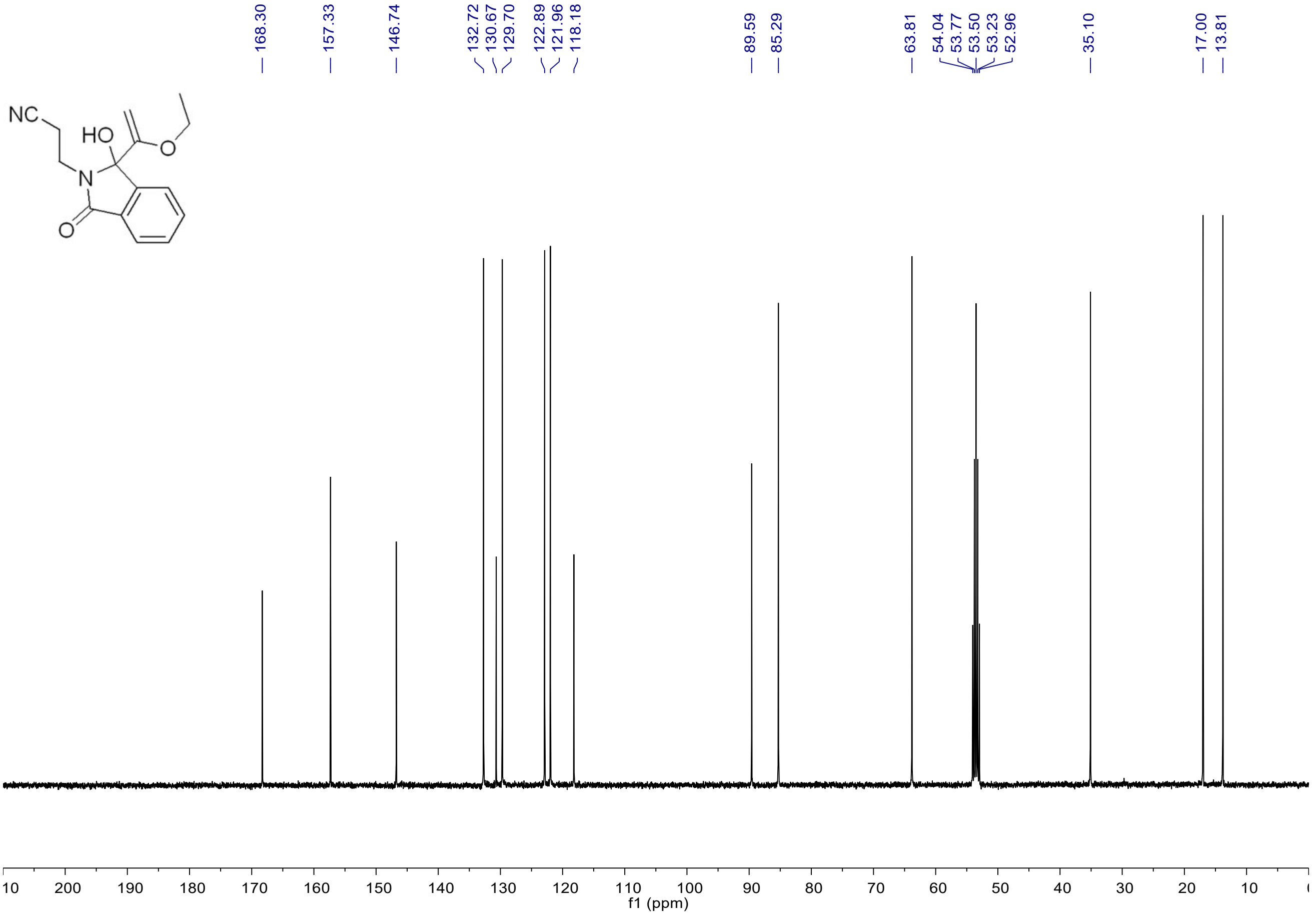
168.30 157.33 146.74 132.72 130.67 129.70 122.89 121.96 118.18 89.59 85.29 63.81 54.04 53.77 53.50 53.23 52.96 35.10 17.00 13.81
NC
HO
N
O
f1 (ppm)

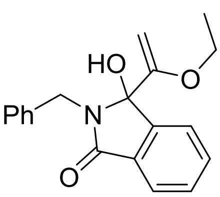

2-benzyl-3-(1-ethoxyvinyl)-3-hydroxyisoindolin-1-one (3hk). A white solid, 61 mg, 99% yield; M.p.: 154-155 °C; ^{1}H NMR (CD_2Cl_2, 400 MHz, TMS) δ 7.49-7.37 (m, 2H), 7.36-7.27 (m, 2H), 7.22-7.15 (m, 2H), 7.15-7.02 (m, 3H), 4.75 (d, J = 2.4 Hz, 1H), 4.40 (s, 1H), 4.23 (q, J = 15.4 Hz, 2H), 4.03 (d, J = 2.4 Hz, 1H), 3.30-3.18 (m, 1H), 3.01-2.89 (m, 1H), 0.71 (t, J = 7.0 Hz, 3H); ^{13}C NMR (CD_2Cl_2, 100 MHz, TMS) δ 168.4, 157.6, 147.0, 138.1, 132.3, 131.2, 129.5, 128.4, 127.9, 126.9, 123.0, 122.0, 89.5, 84.8, 63.2, 42.4, 13.7; IR (neat) ν 3211, 3060, 2973, 2929, 1672, 1615, 1433, 1396, 1350, 1289, 1216, 1086, 1018, 936 cm^{-1}; HRMS (ESI) Calcd. for $C_{19}H_{19}NO_3Na^+$ Requires: 332.1257, Found: 332.1254.

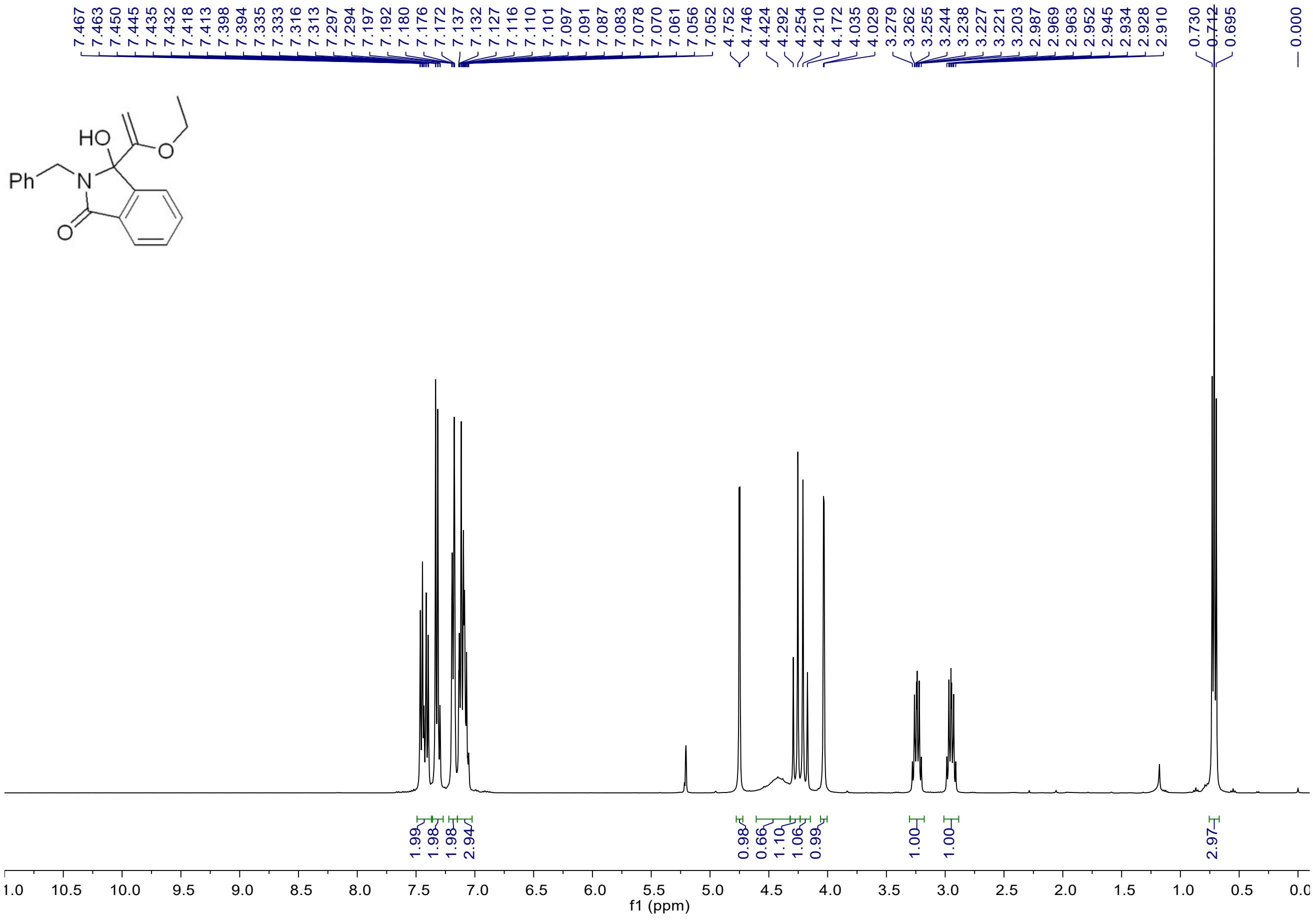

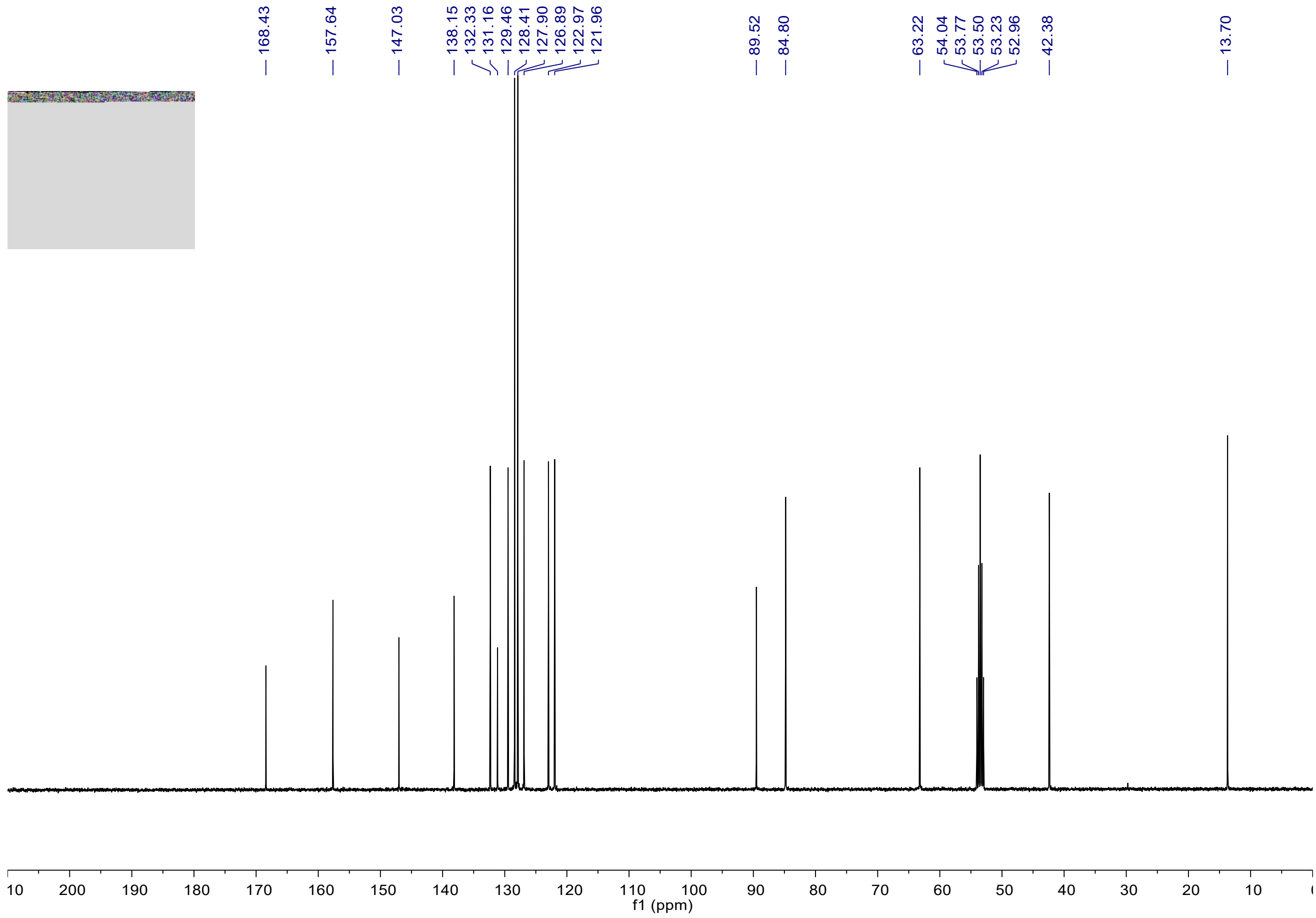

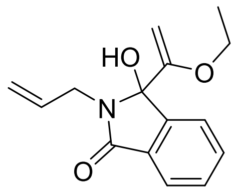

2-allyl-3-(1-ethoxyvinyl)-3-hydroxyisoindolin-1-one (3hl). A white solid, 51 mg, 99% yield; M.p.: 94-95 °C; ^{1}H NMR (CD_2Cl_2, 400 MHz, TMS) δ 7.47-7.26 (m, 4H), 5.76-5.61 (m, 1H), 5.04 (dq, *J* = 17.2, 1.6 Hz, 1H), 4.92 (dq, *J* = 10.2, 1.5 Hz, 1H), 4.75 (d, *J* = 2.4 Hz, 1H), 4.39 (s, 1H), 4.18 (d, *J* = 2.4 Hz, 1H), 3.86-3.75 (m, 1H), 3.59-3.47 (m, 3H), 0.95 (t, *J* = 7.0 Hz, 3H); ^{13}C NMR (CD_2Cl_2, 100 MHz, TMS) δ 167.9, 157.9, 146.9, 134.0, 132.2, 131.2, 129.4, 122.8, 121.9, 116.1, 89.5, 84.6, 63.5, 41.5, 13.8; IR (neat) ν 3240, 2974, 2935, 2892, 1678, 1614, 1427, 1355, 1295, 1191, 1083, 1020, 933, 871 cm^{-1}; HRMS (ESI) Calcd. for $C_{15}H_{17}NO_3Na^+$ Requires: 282.1101, Found: 282.1102.

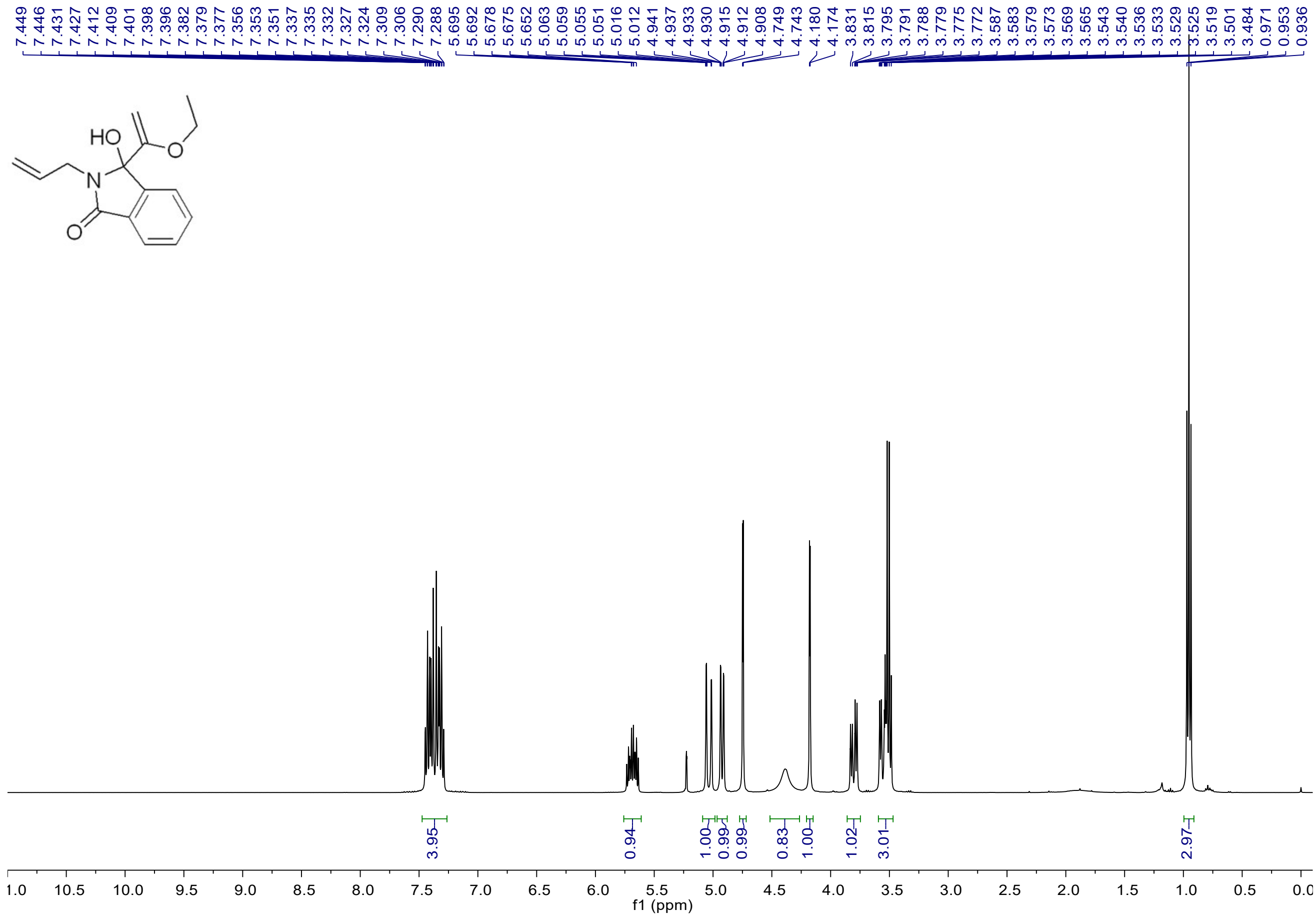
7.449 7.446 7.431 7.427 7.412 7.409 7.401 7.398 7.396 7.382 7.379 7.377 7.356 7.353 7.351 7.337 7.335 7.332 7.327 7.324 7.309 7.306 7.290 7.288 5.695 5.692 5.678 5.675 5.652 5.063 5.059 5.055 5.051 5.016 5.012 4.941 4.937 4.933 4.930 4.915 4.912 4.908 4.749 4.743 4.180 4.174 3.831 3.815 3.795 3.791 3.788 3.779 3.775 3.772 3.587 3.583 3.579 3.573 3.569 3.565 3.543 3.540 3.536 3.533 3.529 3.525 3.519 3.501 3.484 0.971 0.953 0.936
HO
N
O
3.95
0.94
1.00
0.99
0.99
0.83
1.00
1.02
3.01
2.97
f1 (ppm)

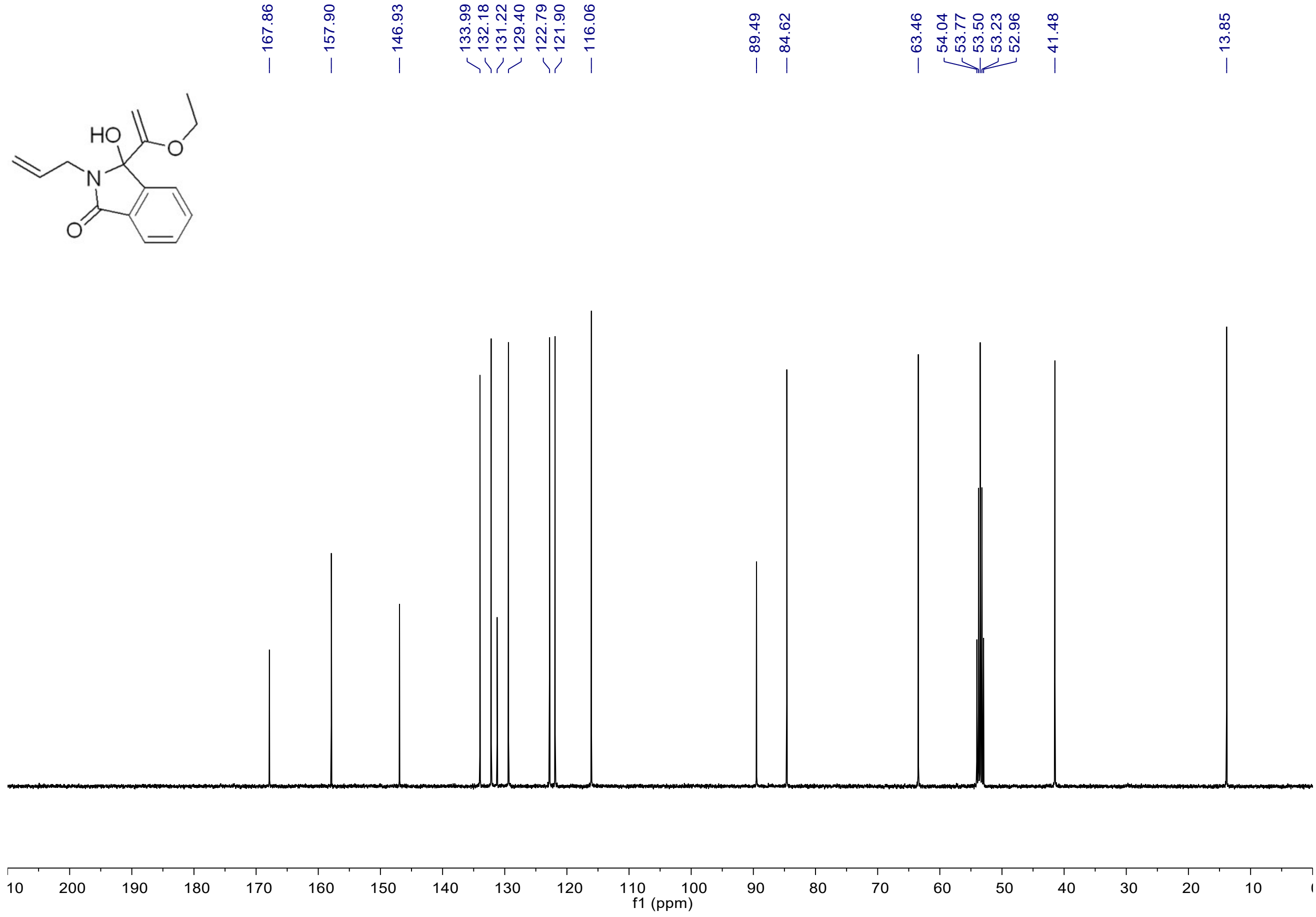
167.86
157.90
146.93
133.99 132.18 131.22 129.40 122.79 121.90
116.06
89.49
84.62
63.46
54.04 53.77 53.50 53.23 52.96
41.48
13.85
HO
N
O
f1 (ppm)

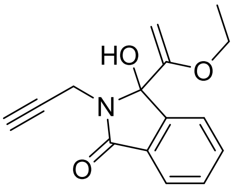

3-(1-ethoxyvinyl)-3-hydroxy-2-(prop-2-yn-1-yl) isoindolin-1-one (3hm). A white solid, 50 mg, 97% yield; M.p.: 117-119 ^{o}C; ^{1}H NMR (CD_2Cl_2, 400 MHz, TMS) δ 7.47 (t, *J* = 7.4 Hz, 2H), 7.42-7.31 (m, 2H), 4.76 (d, *J* = 2.6 Hz, 1H), 4.28 (s, 1H), 4.23 (d, *J* = 2.6 Hz, 1H), 4.02 (dd, *J* = 17.7, 2.6 Hz, 1H), 3.71 (dd, *J* = 17.7, 2.5 Hz, 1H), 3.66-3.50 (m, 2H), 2.05 (t, *J* = 2.5 Hz, 1H), 0.99 (t, *J* = 7.0 Hz, 3H); ^{13}C NMR (CD_2Cl_2, 100 MHz, TMS) δ 167.4, 157.4, 146.7, 132.6, 130.7, 129.6, 123.0, 122.1, 89.4, 84.8, 79.3, 69.9, 63.7, 27.5, 13.9; IR (neat) ν 3309, 2984, 1676, 1634, 1611, 1468, 1396, 1372, 1298, 1272, 1124, 1084, 1001, 941, 869, 770 cm^{-1}; HRMS (ESI) Calcd. for $C_{15}H_{15}NO_3Na^+$ Requires: 280.0944, Found: 280.0942.

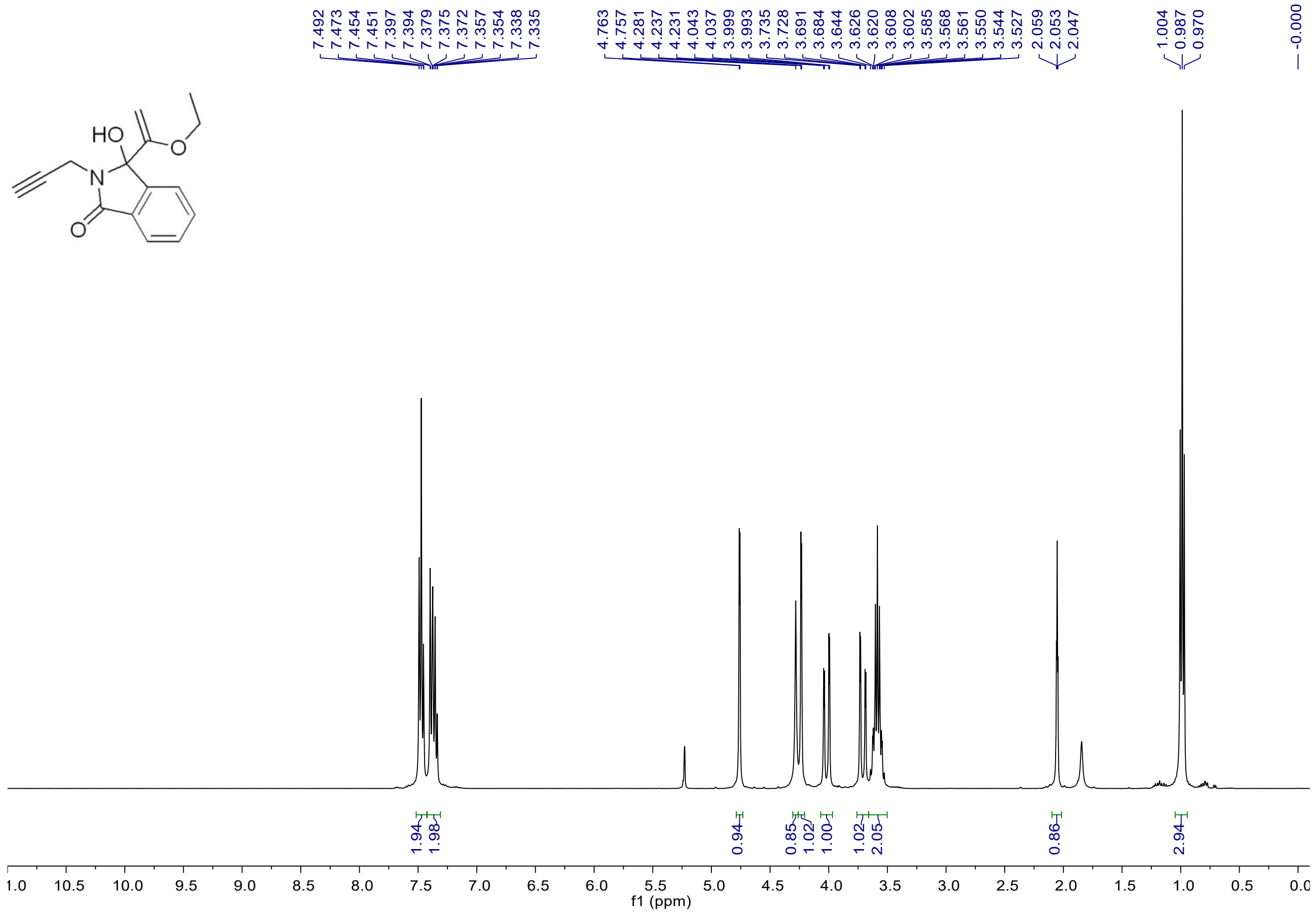

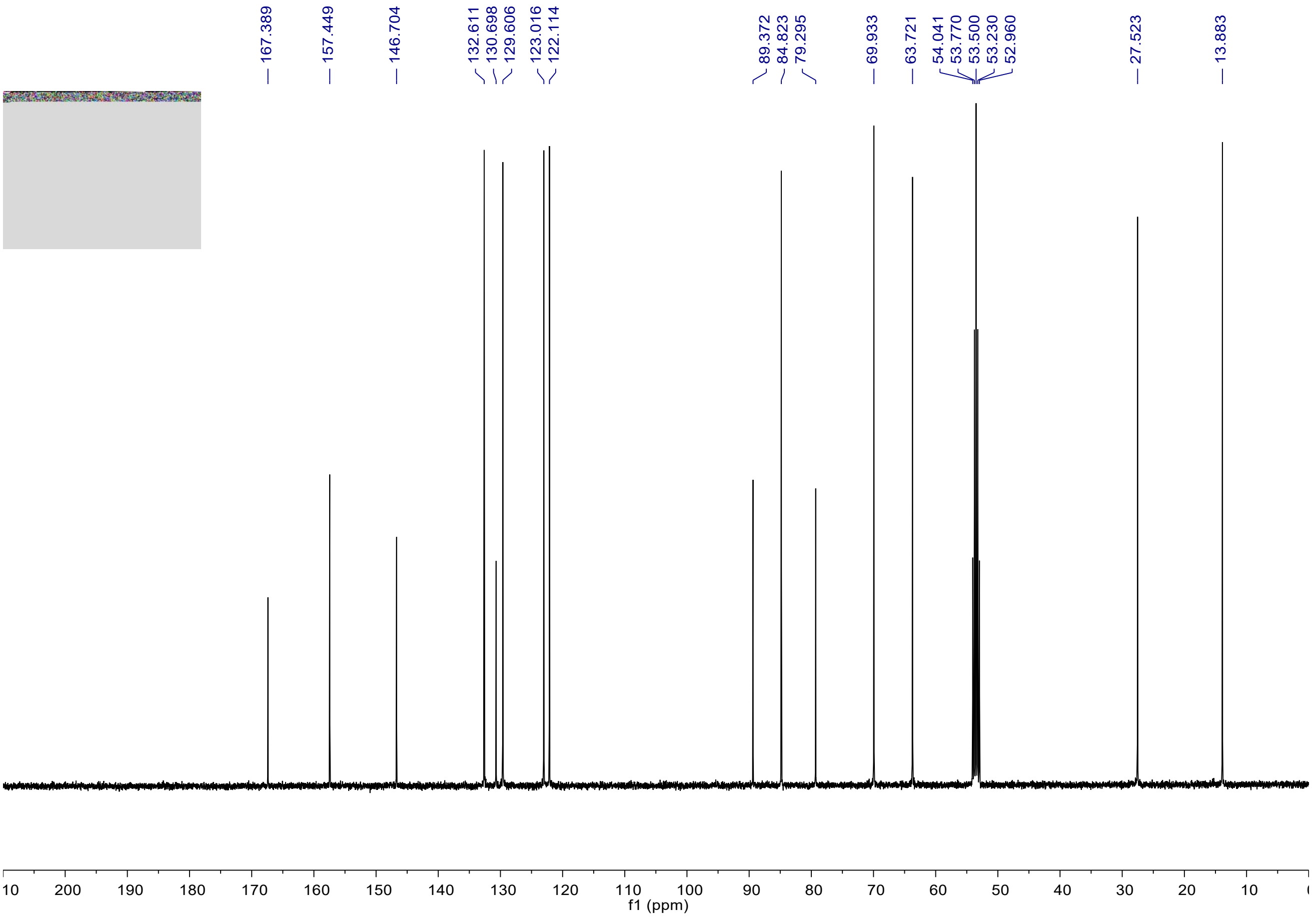

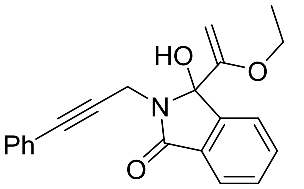

3-(1-ethoxyvinyl)-3-hydroxy-2-(3-phenylprop-2-yn-1-yl) isoindolin-1-one (3hn). A white solid, 59 mg, 89% yield; M.p.: 146-148 ^{o}C; ^{1}H NMR (CD_2Cl_2, 400 MHz, TMS) δ 7.48 (dd, *J* = 12.8, 7.2 Hz, 2H), 7.43-7.31 (m, 2H), 7.31-7.25 (m, 2H), 7.23-7.13 (m, 3H), 4.81 (d, *J* = 2.6 Hz, 1H), 4.42 (s, 1H), 4.28-4.17 (m, 2H), 3.94 (d, *J* = 17.7 Hz, 1H), 3.64-3.48 (m, 2H), 0.92 (t, *J* = 7.0 Hz, 3H); ^{13}C NMR (CD_2Cl_2, 100 MHz, TMS) δ 167.5, 157.6, 146.8, 132.6, 131.6, 130.8, 129.6, 128.3, 128.3, 123.0, 122.9, 122.1, 89.4, 84.9, 84.8, 81.5, 63.7, 28.3, 13.9; IR (neat) ν 3246, 2976, 2872, 1677, 1612, 1490, 1395, 1343, 1277, 1132, 1030, 958, 827, 730 cm^{-1}; HRMS (ESI) Calcd. for $C_{21}H_{19}NO_3Na^+$ Requires: 356.1257, Found: 356.1258.

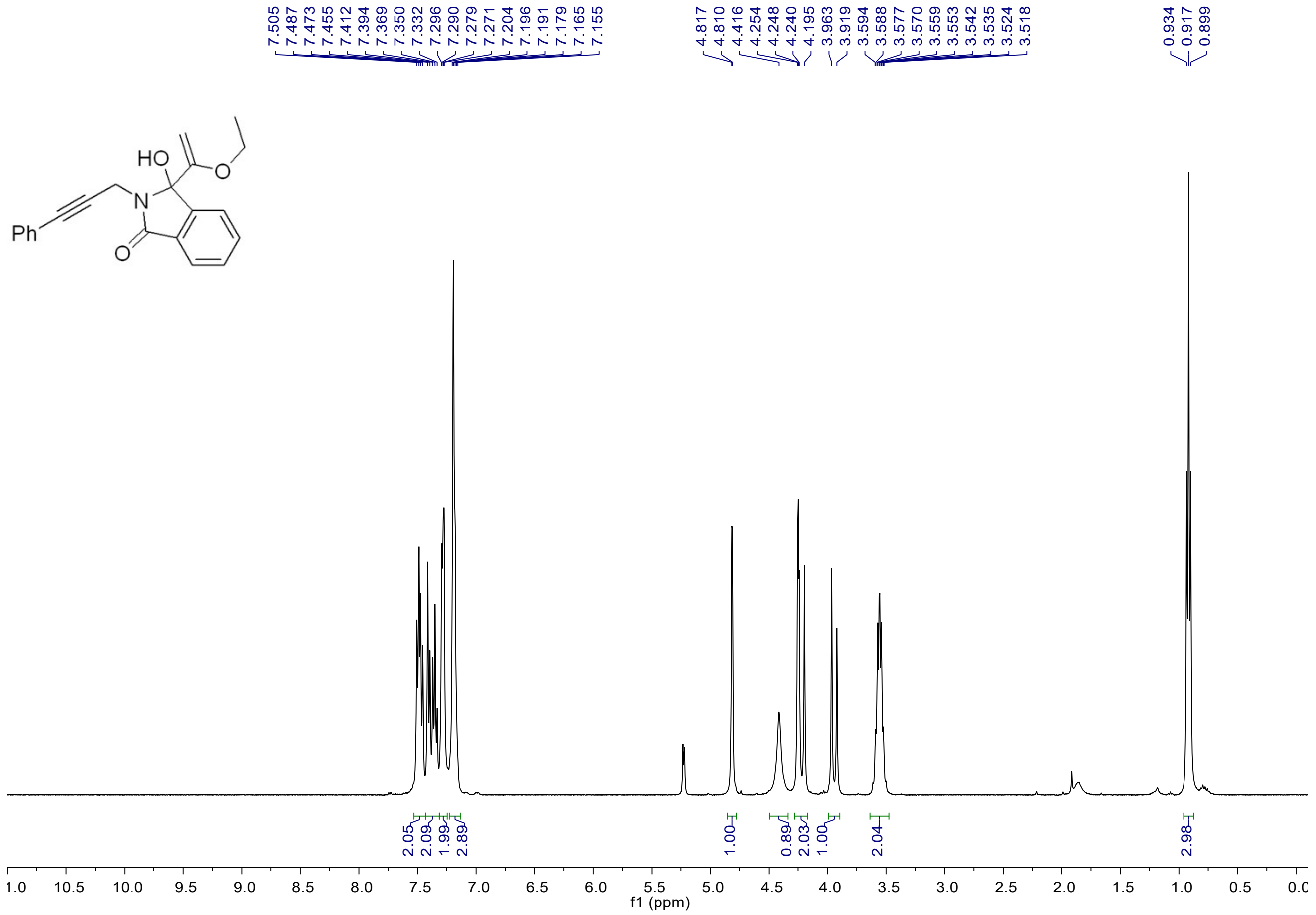

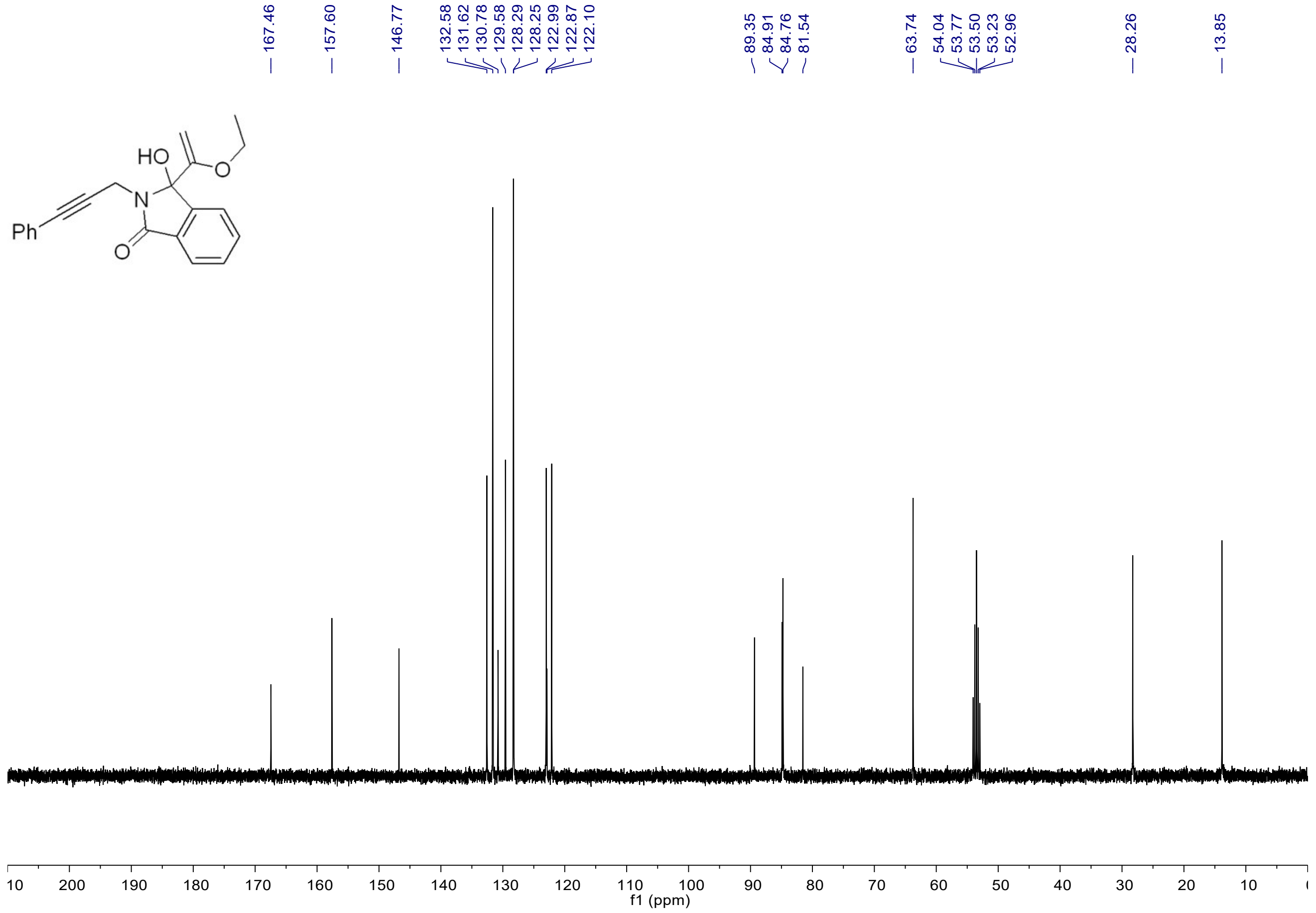

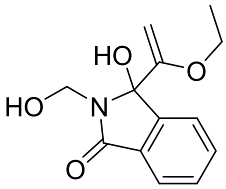

3-(1-ethoxyvinyl)-3-hydroxy-2-(hydroxymethyl) isoindolin-1-one (3ho). A white solid, 31 mg, 63% yield; M.p.: 154-155 °C; ^{1}H NMR (Acetone-d_6, 400 MHz, TMS) δ 7.71-7.56 (m, 2H), 7.55-7.44 (m, 2H), 5.66 (s, 1H), 5.08-4.98 (m, 2H), 4.82 (d, J = 11.0 Hz, 1H), 4.49-4.32 (m, 2H), 3.78-3.59 (m, 2H), 1.01 (t, J = 7.0 Hz, 3H); ^{13}C NMR (Acetone-d_6, 100 MHz, TMS) δ 167.3, 159.2, 147.5, 132.3, 131.7, 129.3, 122.6, 122.1, 89.0, 84.2, 63.4, 62.9, 13.5; IR (neat) ν 3245, 3160, 2976, 2901, 1691, 1632, 1479, 1393, 1156, 1085, 1020, 1011, 975, 947, 846, 803 cm^{-1}; HRMS (ESI) Calcd. for $C_{13}H_{15}NO_4Na^+$ Requires: 272.0893, Found: 272.0893.

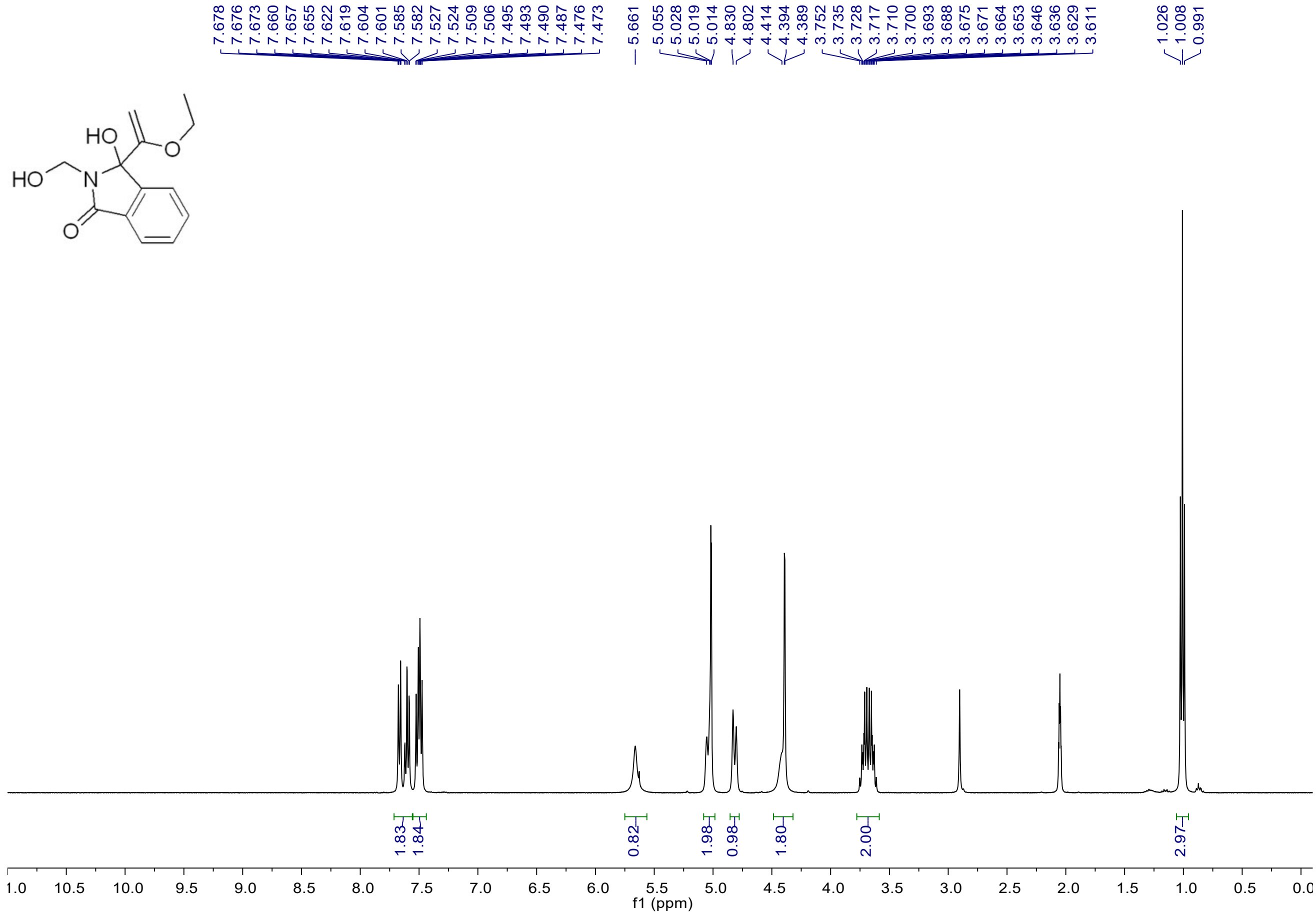

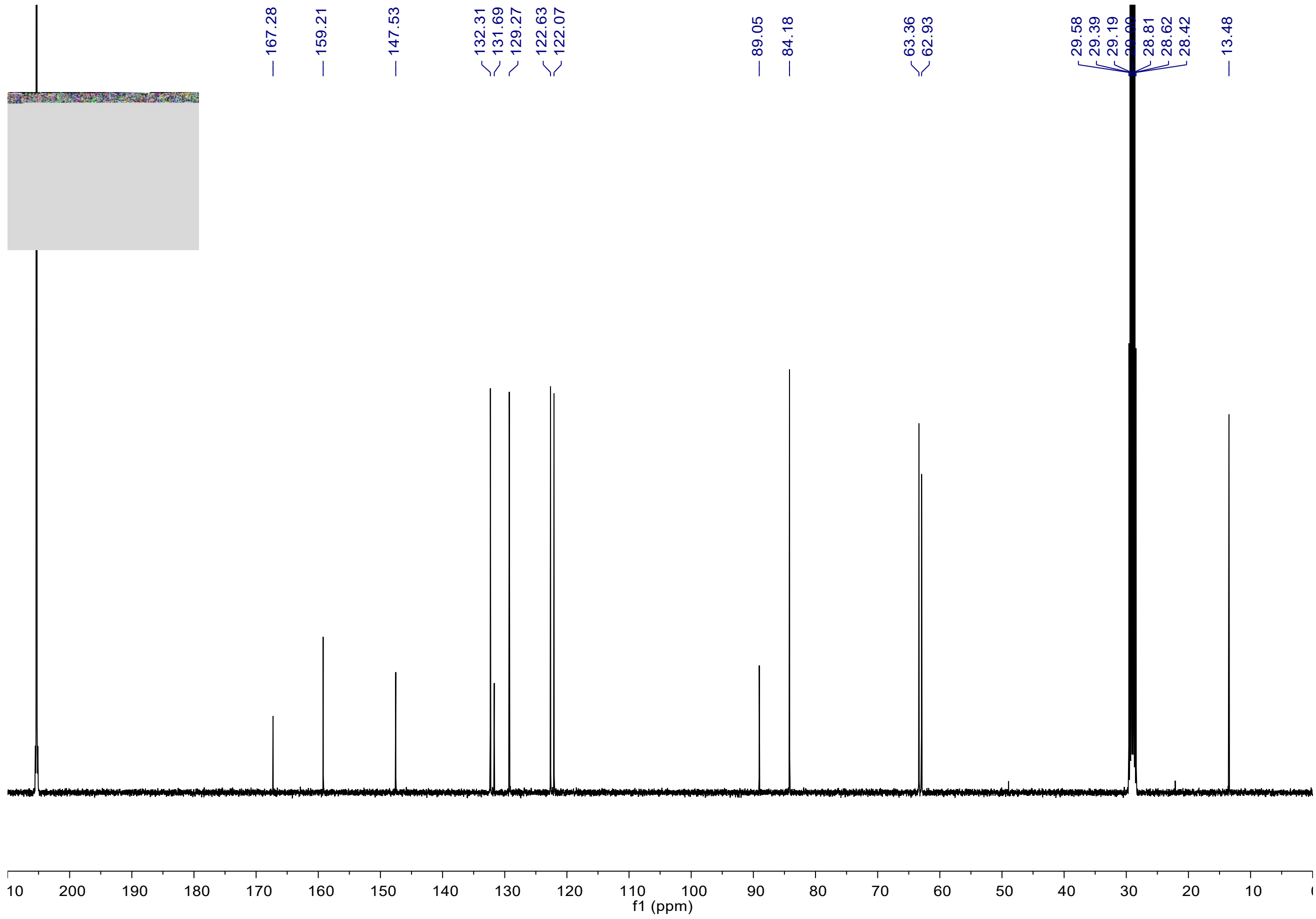

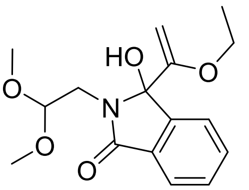

2-(2,2-dimethoxyethyl)-3-(1-ethoxyvinyl)-3-hydroxyisoindolin-1-one (3hp). A white solid, 61 mg, quant. yield; M.p.: 94-95 °C; ^{1}H NMR (CD_2Cl_2, 400 MHz, TMS) δ 7.69 (d, J = 7.4 Hz, 1H), 7.59-7.51 (m, 1H), 7.51-7.39 (m, 2H), 5.15 (s, 1H), 5.00 (d, J = 1.9 Hz, 1H), 4.56 (dd, J = 6.6, 3.2 Hz, 1H), 4.40 (d, J = 1.8 Hz, 1H), 4.00 (dd, J = 14.8, 3.2 Hz, 1H), 3.75-3.54 (m, 2H), 3.43 (s, 3H), 3.40 (s, 3H), 3.02 (dd, J = 14.8, 6.7 Hz, 1H), 1.03 (t, J = 7.0 Hz, 3H); ^{13}C NMR (CD_2Cl_2, 100 MHz, TMS) δ 168.3, 158.8, 146.8, 132.3, 130.9, 129.2, 122.8, 121.7, 102.6, 89.4, 85.7, 63.8, 55.0, 54.4, 41.5, 13.8; IR (neat) ν 3202, 2983, 2832, 1682, 1613, 1411, 1373, 1278, 1124, 1088, 1025, 1002, 953, 914, 875 cm^{-1}; HRMS (ESI) Calcd. for $C_{16}H_{21}NO_5Na^+$ Requires: 330.1312, Found: 330.1310.

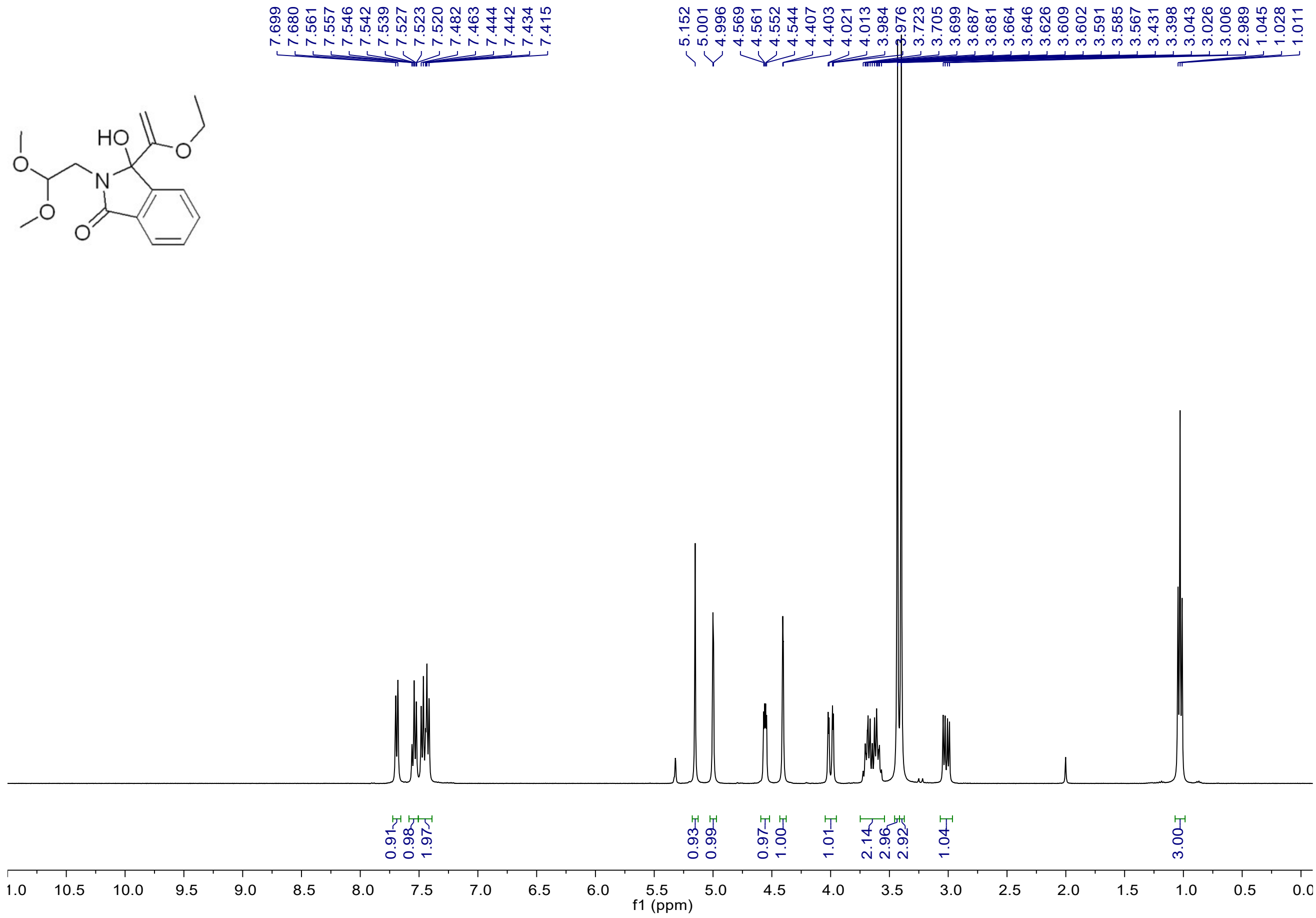
7.699
7.680
7.561
7.557
7.546
7.542
7.539
7.527
7.523
7.520
7.482
7.463
7.444
7.442
7.434
7.415
5.152
5.001
4.996
4.569
4.561
4.552
4.544
4.407
4.403
4.021
4.013
3.984
3.976
3.723
3.705
3.699
3.687
3.681
3.664
3.646
3.626
3.609
3.602
3.591
3.585
3.567
3.431
3.398
3.043
3.026
3.006
2.989
1.045
1.028
1.011
HO
N
O
0.91
0.98
1.97
0.93
0.99
0.97
1.00
1.01
2.14
2.96
2.92
1.04
3.00
f1 (ppm)

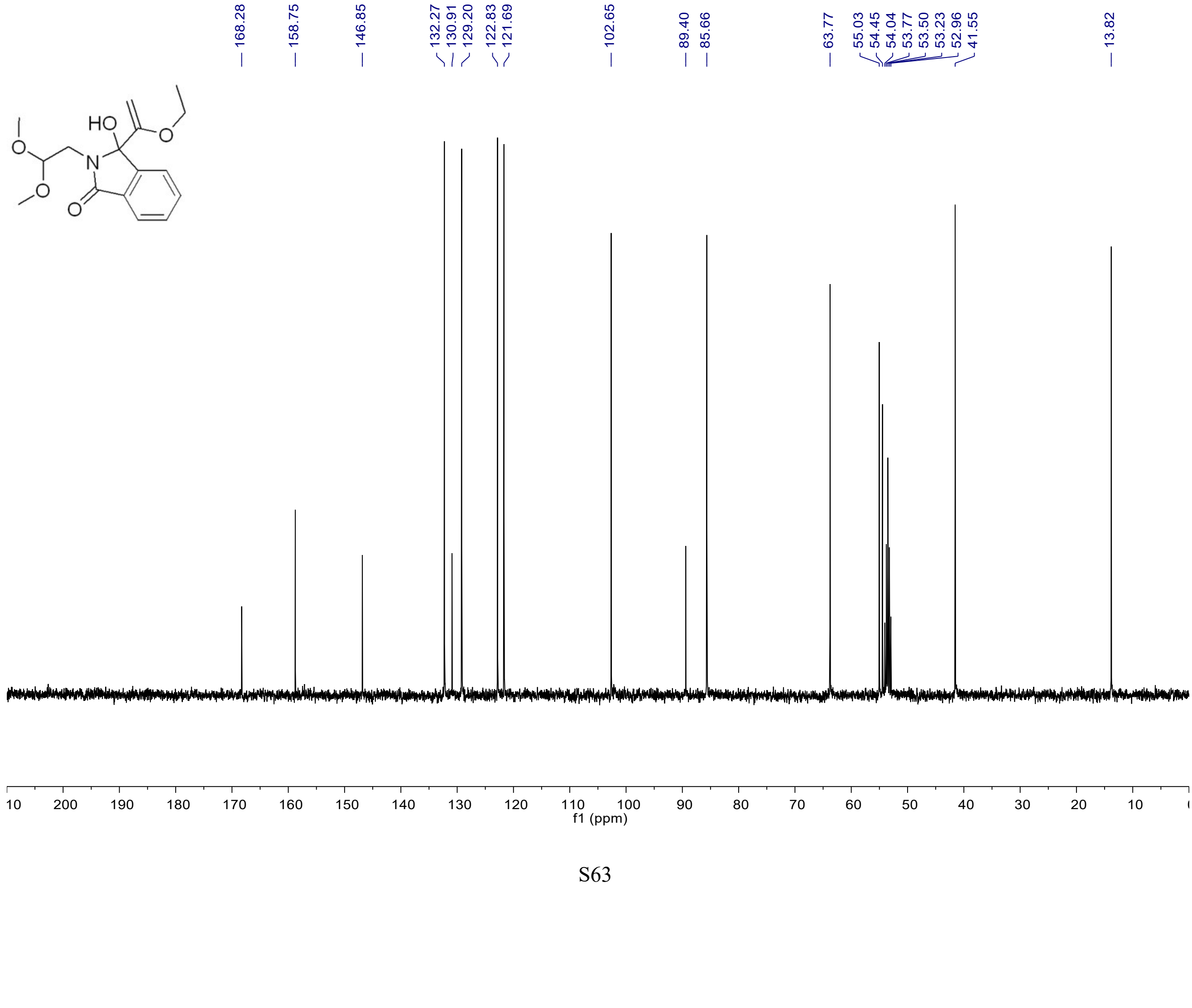
168.28
158.75
146.85
132.27
130.91
129.20
122.83
121.69
102.65
89.40
85.66
63.77
55.03
54.45
54.04
53.77
53.50
53.23
52.96
41.55
13.82
HO
N
O
f1 (ppm)

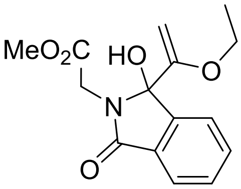

methyl 2-(1-(1-ethoxyvinyl)-1-hydroxy-3-oxoisoindolin-2-yl) acetate (3hq). A white solid, 47 mg, 81% yield; M.p.: 83-85 °C; ^{1}H NMR (CD_2Cl_2, 400 MHz, TMS) δ 7.65-7.58 (m, 1H), 7.53-7.44 (m, 1H), 7.44-7.36 (m, 2H), 4.75 (d, *J* = 2.5 Hz, 1H), 4.37 (d, *J* = 17.7 Hz, 1H), 4.24 (d, *J* = 2.5 Hz, 1H), 3.72 (d, *J* = 17.7 Hz, 1H), 3.63 (s, 4H), 3.63-3.48 (m, 4H), 0.98 (t, *J* = 7.0 Hz, 3H); ^{13}C NMR (CD_2Cl_2, 100 MHz, TMS) δ 171.3, 167.9, 157.5, 147.1, 132.6, 130.6, 129.6, 123.1, 122.1, 89.4, 85.3, 63.8, 52.6, 40.7, 13.8; IR (neat) ν 3304, 2984, 2941, 1761, 1678, 1633, 1468, 1413, 1371, 1279, 1225, 1180, 1112, 994 cm^{-1}; HRMS (ESI) Calcd. for $C_{15}H_{17}NO_5Na^+$ Requires: 314.0999, Found: 314.0996.

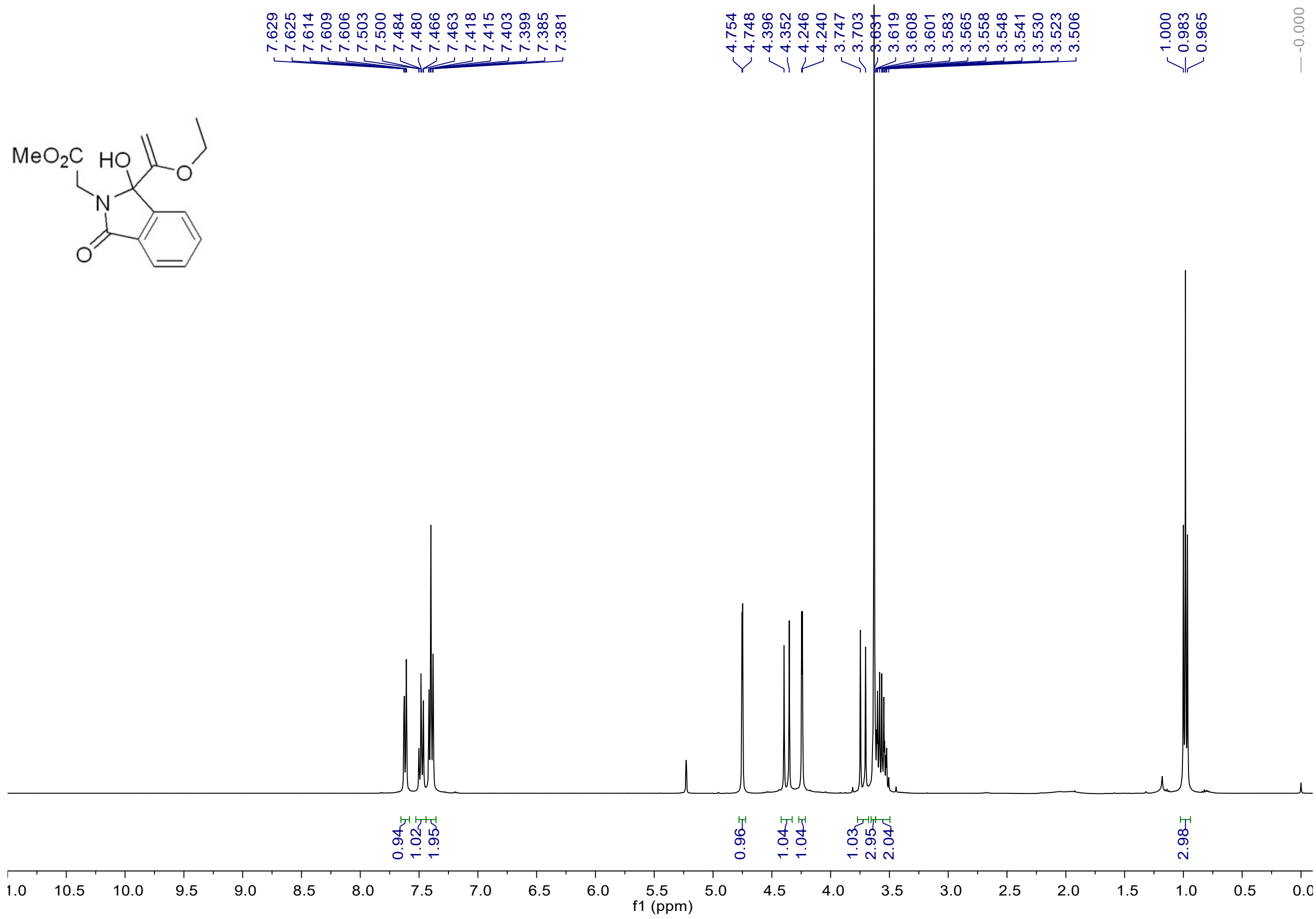

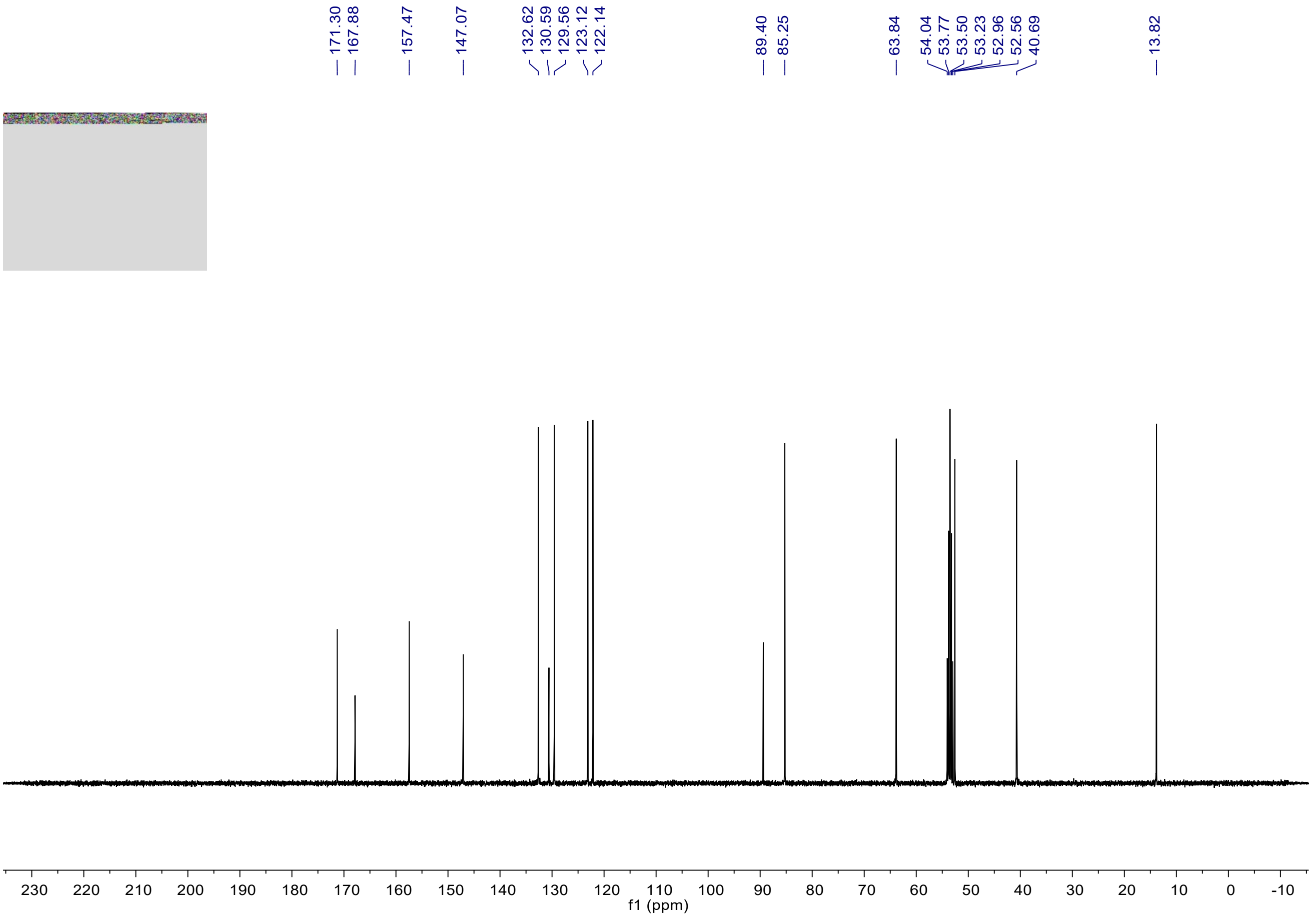

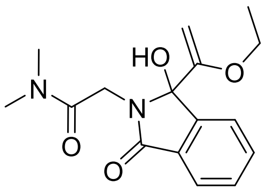

2-(1-(1-ethoxyvinyl)-1-hydroxy-3-oxoisoindolin-2-yl)-*N*,*N*-dimethylacetamide (3hr). A white solid, 61 mg, quant. yield; M.p.: 125-126 ^{o}C; ^{1}H NMR (CD_2Cl_2, 400 MHz, TMS) δ 7.62 (d, *J* = 7.5 Hz, 1H), 7.48 (t, *J* = 7.4 Hz, 1H), 7.38 (t, *J* = 7.3 Hz, 2H), 6.74 (s, 1H), 4.96-4.84 (m, 2H), 4.32 (d, *J* = 2.3 Hz, 1H), 3.69-3.49 (m, 3H), 3.03 (s, 3H), 2.87 (s, 3H), 0.96 (t, *J* = 6.9 Hz, 3H); ^{13}C NMR (CD_2Cl_2, 100 MHz, TMS) δ 170.3, 168.3, 158.5, 148.3, 132.5, 130.3, 129.0, 122.9, 121.8, 89.3, 85.8, 63.7, 41.5, 37.0, 36.0, 29.7, 13.9; IR (neat) ν 3316, 2927, 1710, 1654, 1626, 1466, 1422, 1382, 1318, 1218, 1136, 1056, 984, 838, 708 cm^{-1}; HRMS (ESI) Calcd. for $C_{16}H_{20}N_2O_4Na^+$ Requires: 327.1315, Found: 327.1313.

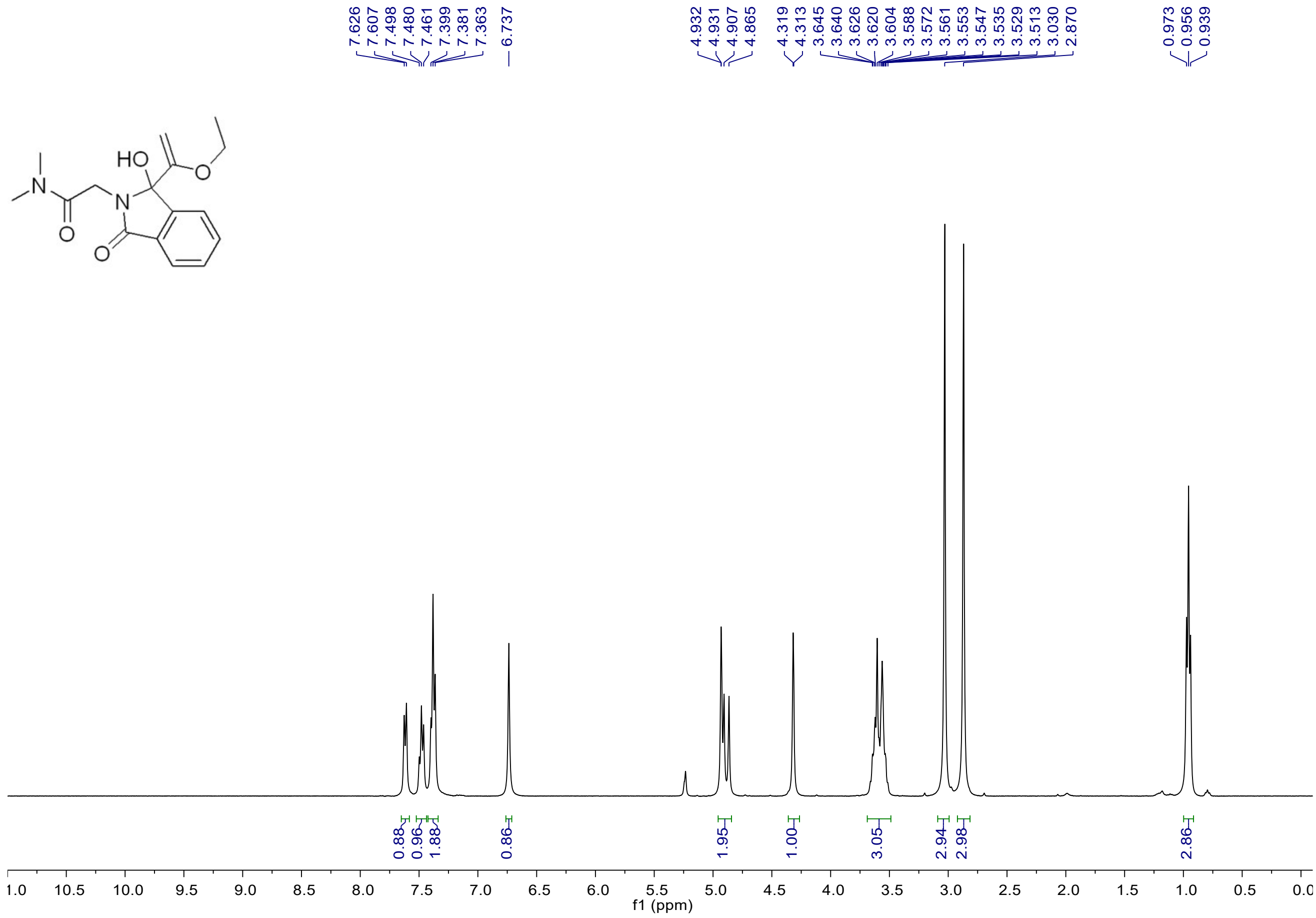
7.626 7.607 7.498 7.480 7.461 7.399 7.381 7.363 6.737
4.932 4.931 4.907 4.865 4.319 4.313 3.645 3.640 3.626 3.620 3.604 3.588 3.572 3.561 3.553 3.547 3.535 3.529 3.513 3.030 2.870
0.973 0.956 0.939
HO
N
O
0.88 0.96 1.88 0.86 1.95 1.00 3.05 2.94 2.98 2.86
f1 (ppm)

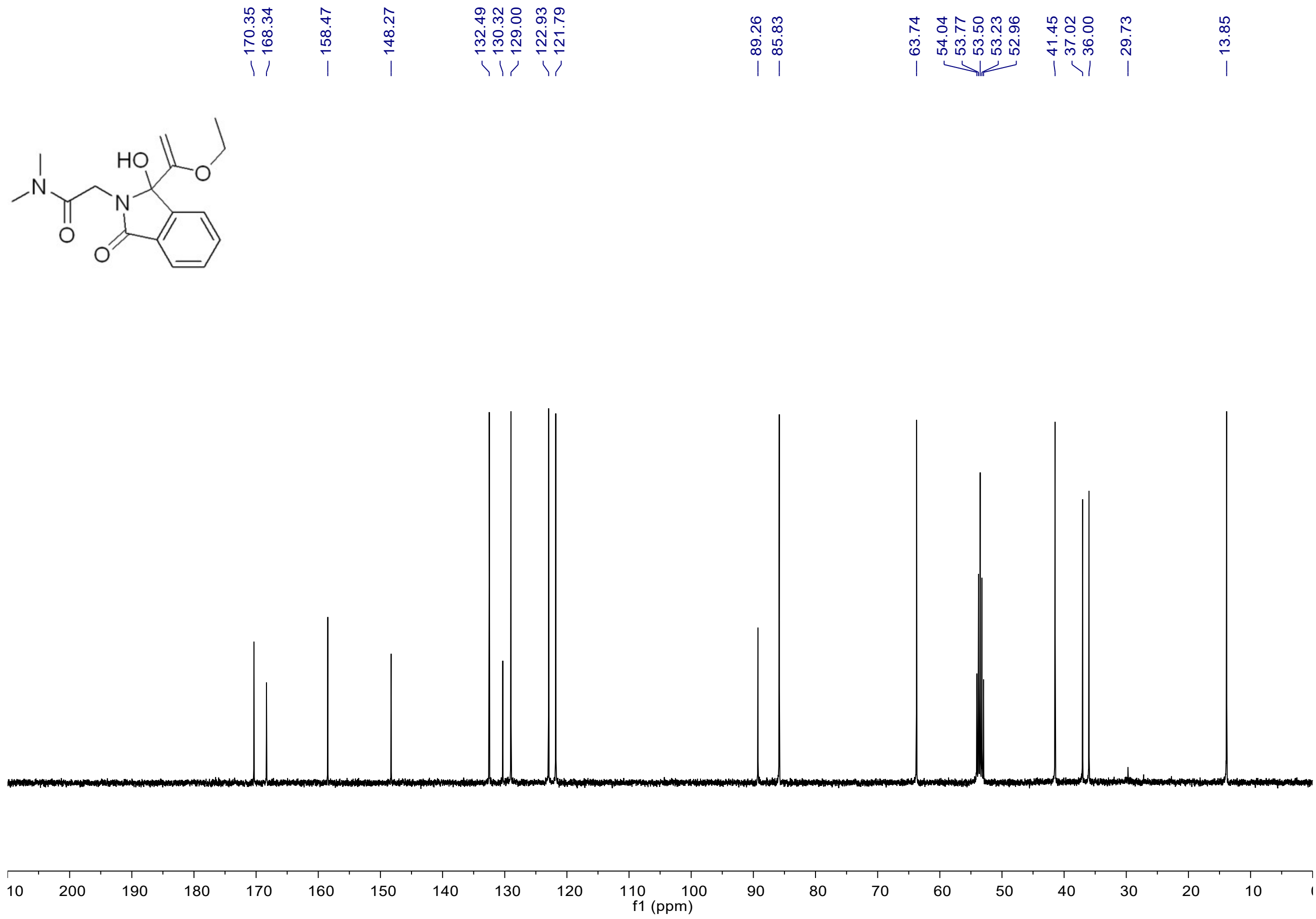
170.35 168.34 158.47 148.27 132.49 130.32 129.00 122.93 121.79 89.26 85.83 63.74 54.04 53.77 53.50 53.23 52.96 41.45 37.02 36.00 29.73 13.85
HO
N
O
f1 (ppm)

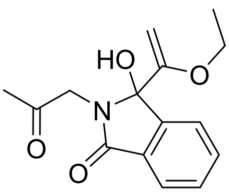

3-(1-ethoxyvinyl)-3-hydroxy-2-(2-oxopropyl)isoindolin-1-one (3hs). A colorless oil, 52 mg, 95% yield; ^{1}H NMR (CD_2Cl_2, 400 MHz, TMS) δ 7.63-7.56 (m, 1H), 7.47 (td, J = 7.1, 6.6, 1.2 Hz, 1H), 7.43-7.35 (m, 2H), 5.24 (s, 1H), 4.73 (d, J = 2.4 Hz, 1H), 4.45 (d, J = 18.2 Hz, 1H), 4.23 (d, J = 2.5 Hz, 1H), 3.73 (d, J = 18.3 Hz, 1H), 3.65-3.50 (m, 2H), 2.10 (s, 3H), 0.98 (t, J = 7.0 Hz, 3H); ^{13}C NMR (CD_2Cl_2, 100 MHz, TMS) δ 205.8, 168.0, 157.6, 147.2, 132.6, 130.5, 129.5, 123.0, 122.1, 89.3, 85.2, 63.8, 49.3, 27.0, 13.8; IR (neat) ν 3339, 2981, 2907, 1683, 1635, 1417, 1355, 1273, 1172, 1123, 1085, 1004, 951, 825 cm^{-1}; HRMS (ESI) Calcd. for $C_{15}H_{17}NO_4Na^+$ Requires: 298.1050, Found: 298.1052.

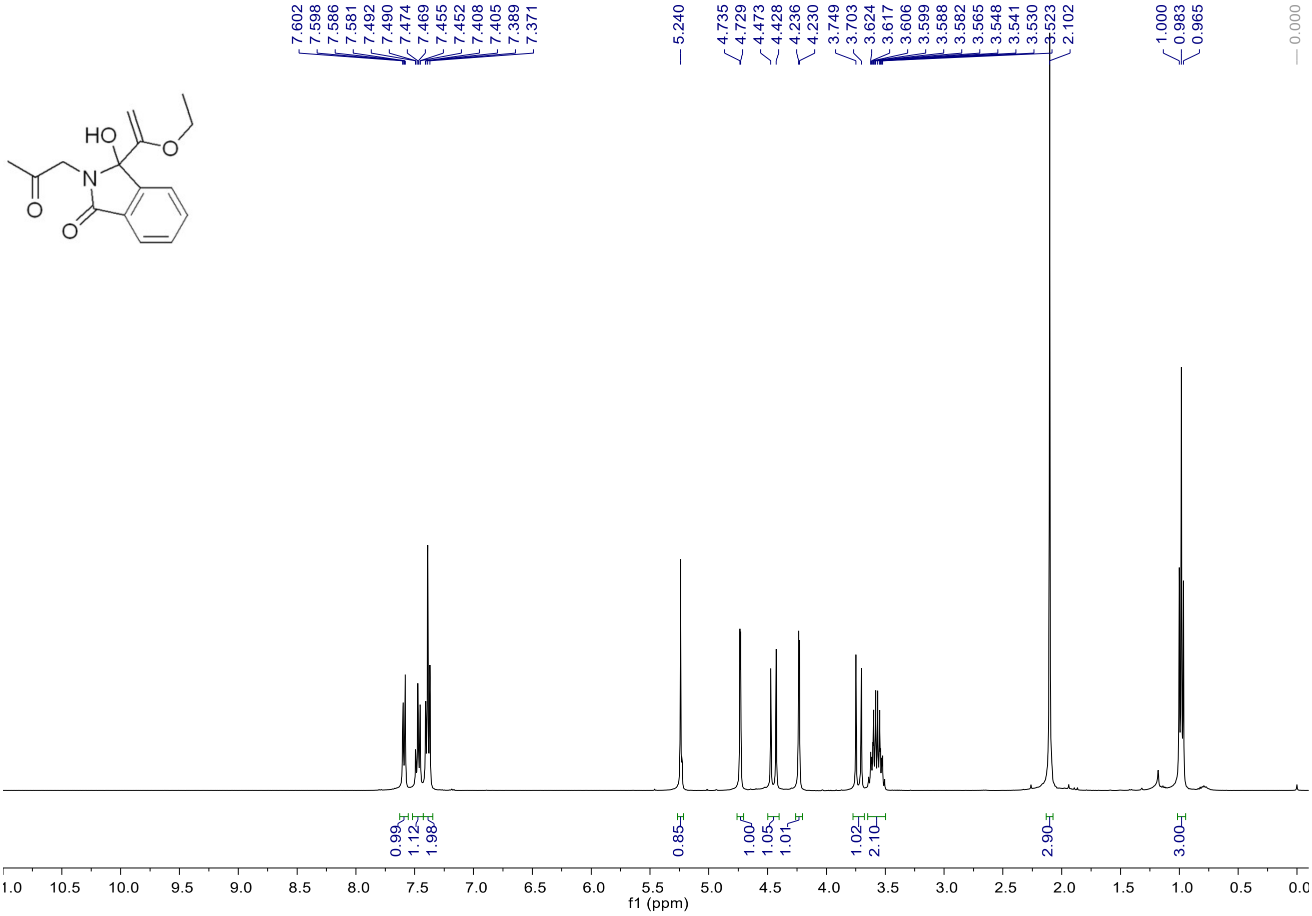

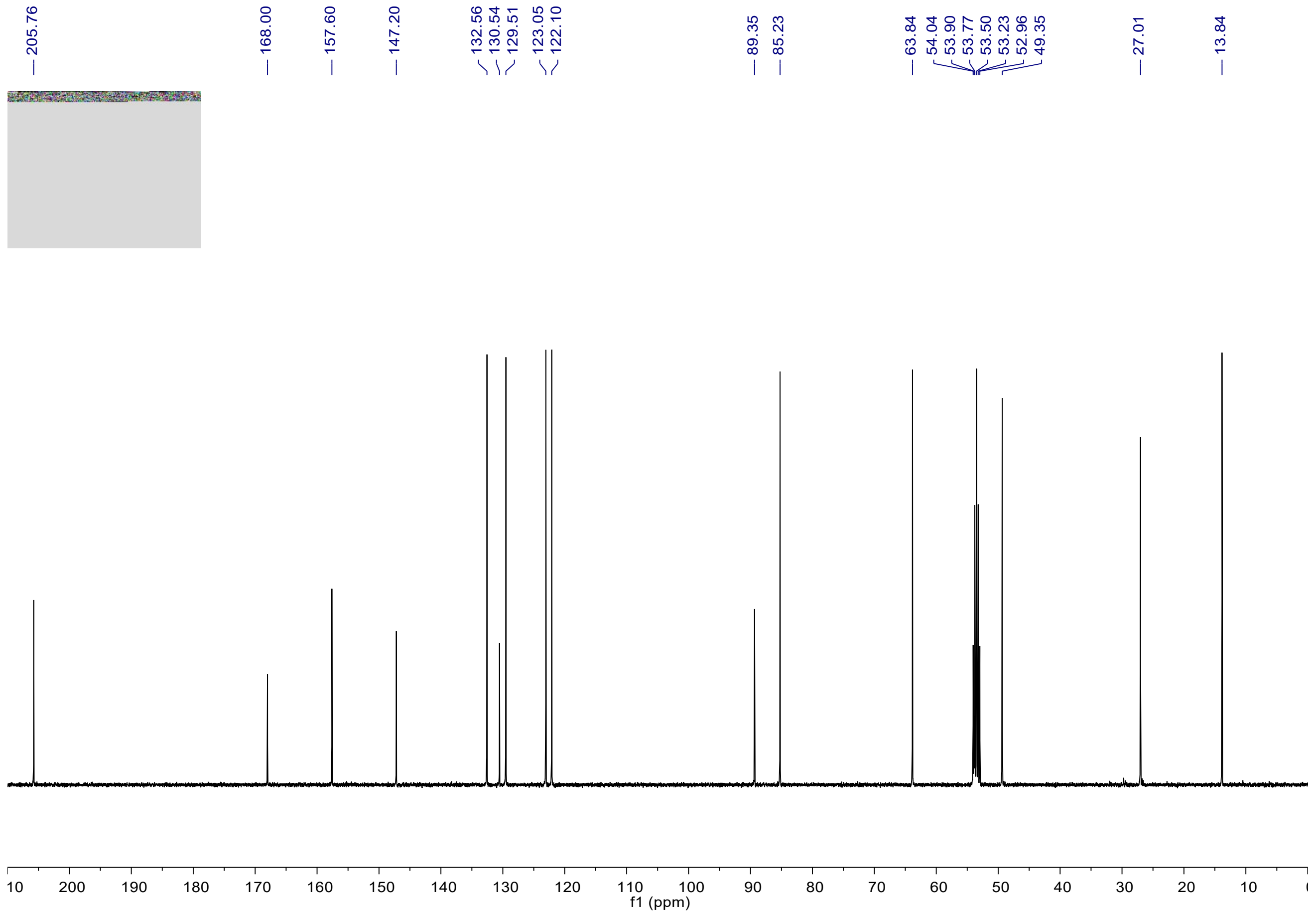

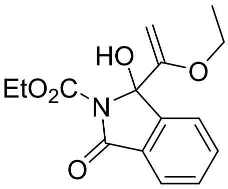

ethyl 1-(1-ethoxyvinyl)-1-hydroxy-3-oxoisoindoline-2-carboxylate (3ht). A white solid, 44 mg, 75% yield; M.p.: 144-146 °C; ^{1}H NMR (CD_2Cl_2, 400 MHz, TMS) δ 7.76-7.69 (m, 1H), 7.62-7.53 (m, 1H), 7.51-7.42 (m, 2H), 4.80 (d, J = 2.9 Hz, 1H), 4.68 (s, 1H), 4.34-4.22 (m, 2H), 4.19 (d, J = 3.1 Hz, 1H), 3.69-3.56 (m, 1H), 3.56-3.44 (m, 1H), 1.28 (t, J = 7.1 Hz, 3H), 0.98 (t, J = 7.0 Hz, 3H); ^{13}C NMR (CD_2Cl_2, 100 MHz, TMS) δ 164.9, 160.5, 151.9, 145.0, 134.2, 130.3, 129.7, 124.2, 122.6, 90.0, 82.9, 64.2, 62.9, 14.1, 13.8; IR (neat) ν 3376, 2978, 1756, 1680, 1609, 1444, 1396, 1341, 1256, 1178, 1145, 1021, 975, 891, 780 cm^{-1}; HRMS (ESI) Calcd. for $C_{20}H_{21}NO_5Na^+$ Requires: 314.0999, Found: 314.0994.

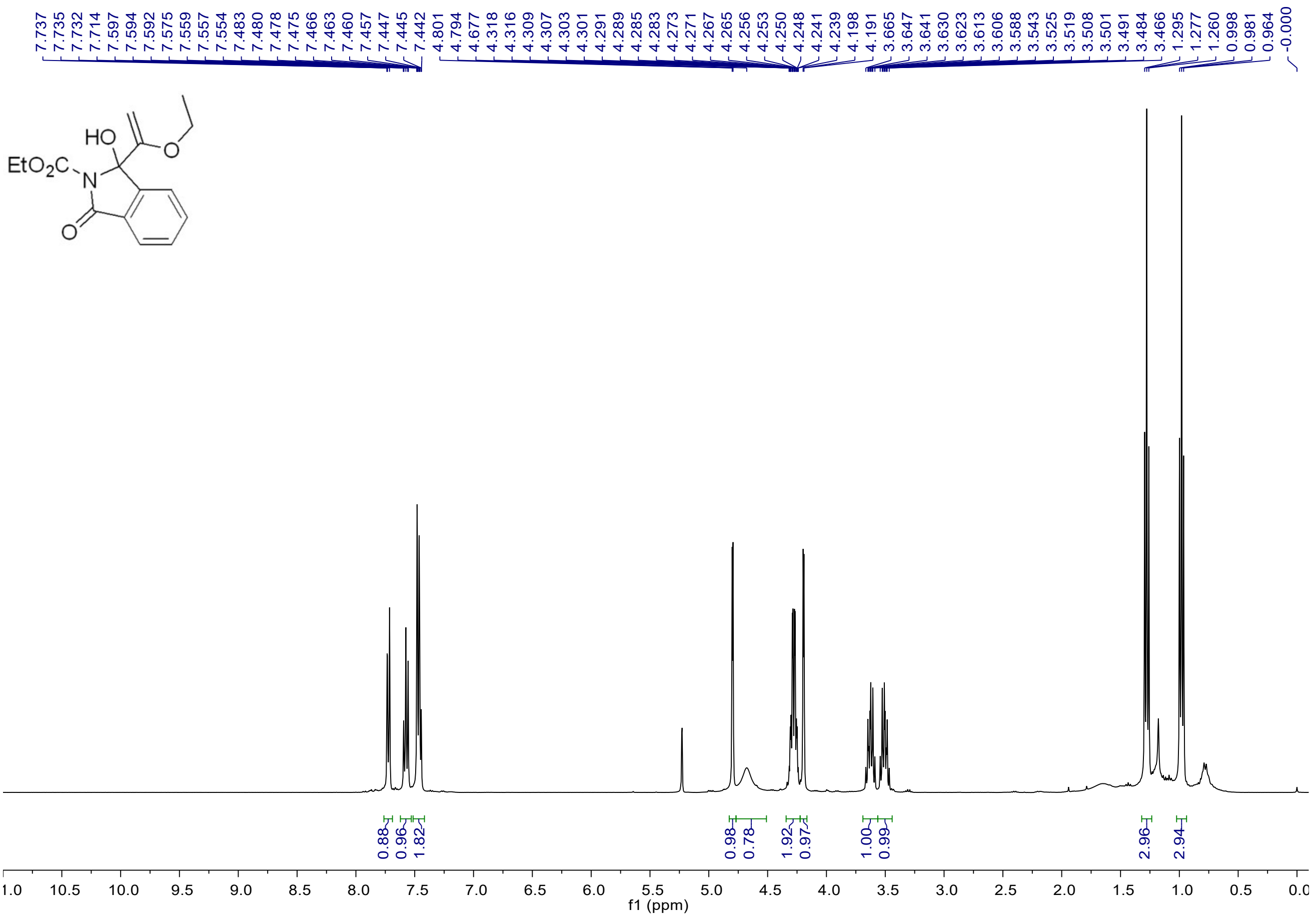
7.737
7.735
7.732
7.714
7.597
7.594
7.592
7.575
7.559
7.557
7.554
7.483
7.480
7.478
7.475
7.466
7.463
7.460
7.457
7.447
7.445
7.442
4.801
4.794
4.677
4.318
4.316
4.309
4.307
4.303
4.301
4.291
4.289
4.285
4.283
4.273
4.271
4.267
4.265
4.256
4.253
4.250
4.248
4.241
4.239
4.198
4.191
3.665
3.647
3.641
3.630
3.623
3.613
3.606
3.588
3.543
3.525
3.519
3.508
3.501
3.491
3.484
3.466
1.295
1.277
1.260
0.998
0.981
0.964
-0.000
HO
EtO2C
N
O
O
0.88
0.96
1.82
0.98
0.78
1.92
0.97
1.00
0.99
2.96
2.94
f1 (ppm)

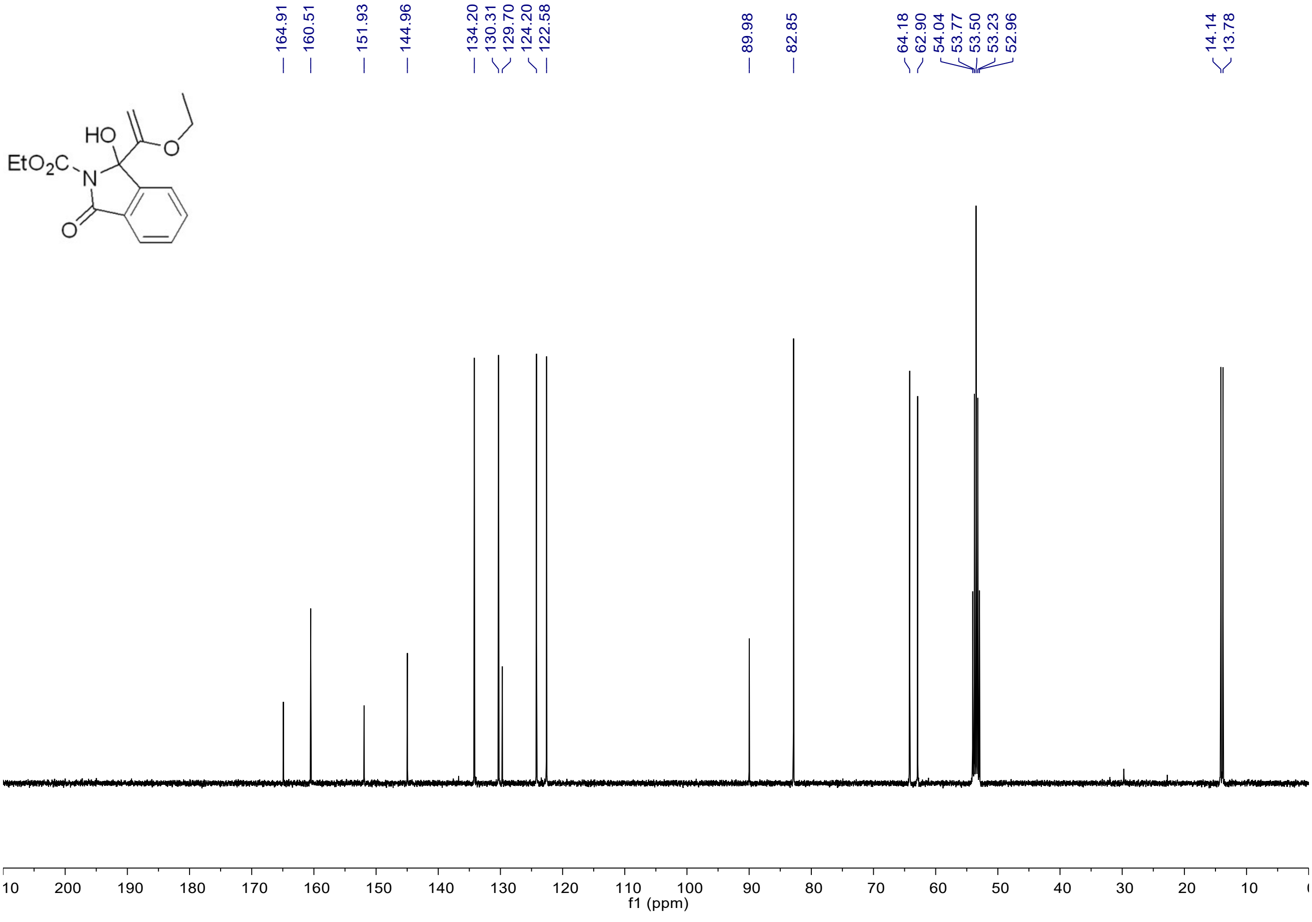
164.91
160.51
151.93
144.96
134.20
130.31
129.70
124.20
122.58
89.98
82.85
64.18
62.90
54.04
53.77
53.50
53.23
52.96
14.14
13.78
HO
EtO2C
N
O
O
f1 (ppm)

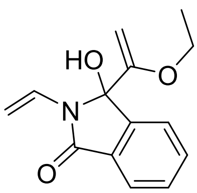

3-(1-ethoxyvinyl)-3-hydroxy-2-vinylisoindolin-1-one (3hu). A white solid, 36 mg, 74% yield; M.p.: 120-122 °C; ^{1}H NMR (CD_2Cl_2, 400 MHz, TMS) δ 7.54-7.38 (m, 3H), 7.38-7.30 (m, 1H), 6.55 (dd, *J* = 16.5, 9.8 Hz, 1H), 5.08 (d, *J* = 16.5 Hz, 1H), 4.83 (d, *J* = 2.6 Hz, 1H), 4.44 (d, *J* = 9.8 Hz, 1H), 4.26 (d, *J* = 2.6 Hz, 1H), 4.18 (s, 1H), 3.66-3.48 (m, 2H), 0.95 (t, *J* = 7.0 Hz, 3H); ^{13}C NMR (CD_2Cl_2, 100 MHz, TMS) δ 166.2, 157.7, 147.0, 133.1, 129.9, 129.8, 126.0, 123.5, 121.8, 97.9, 89.5, 84.6, 64.0, 13.8; IR (neat) ν 3322, 2977, 1678, 1637, 1612, 1467, 1419, 1354, 1272, 1244, 1131, 1084, 985, 866 cm^{-1}; HRMS (ESI) Calcd. for $C_{14}H_{15}NO_3Na^+$ Requires: 268.0944, Found: 268.0949.

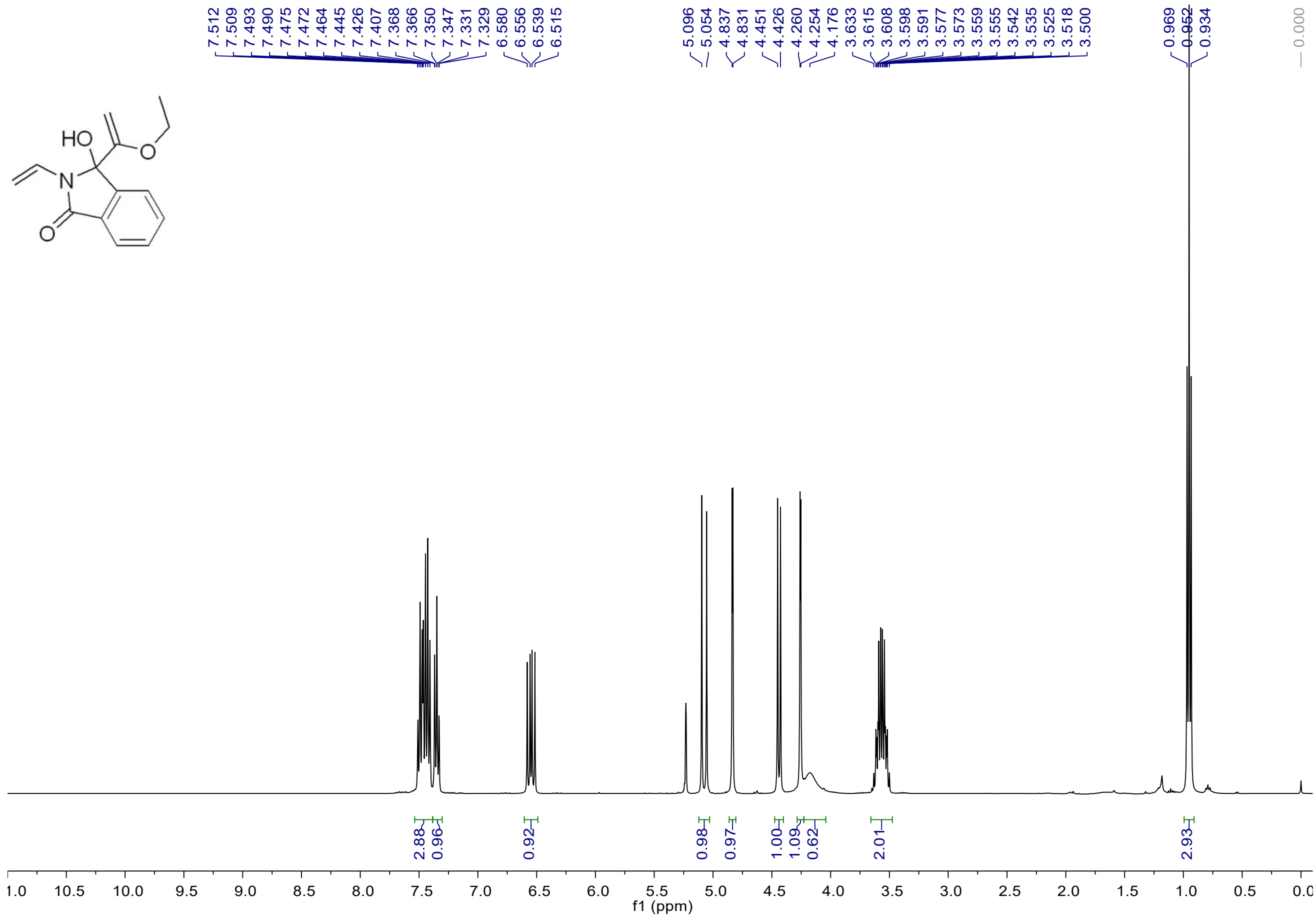

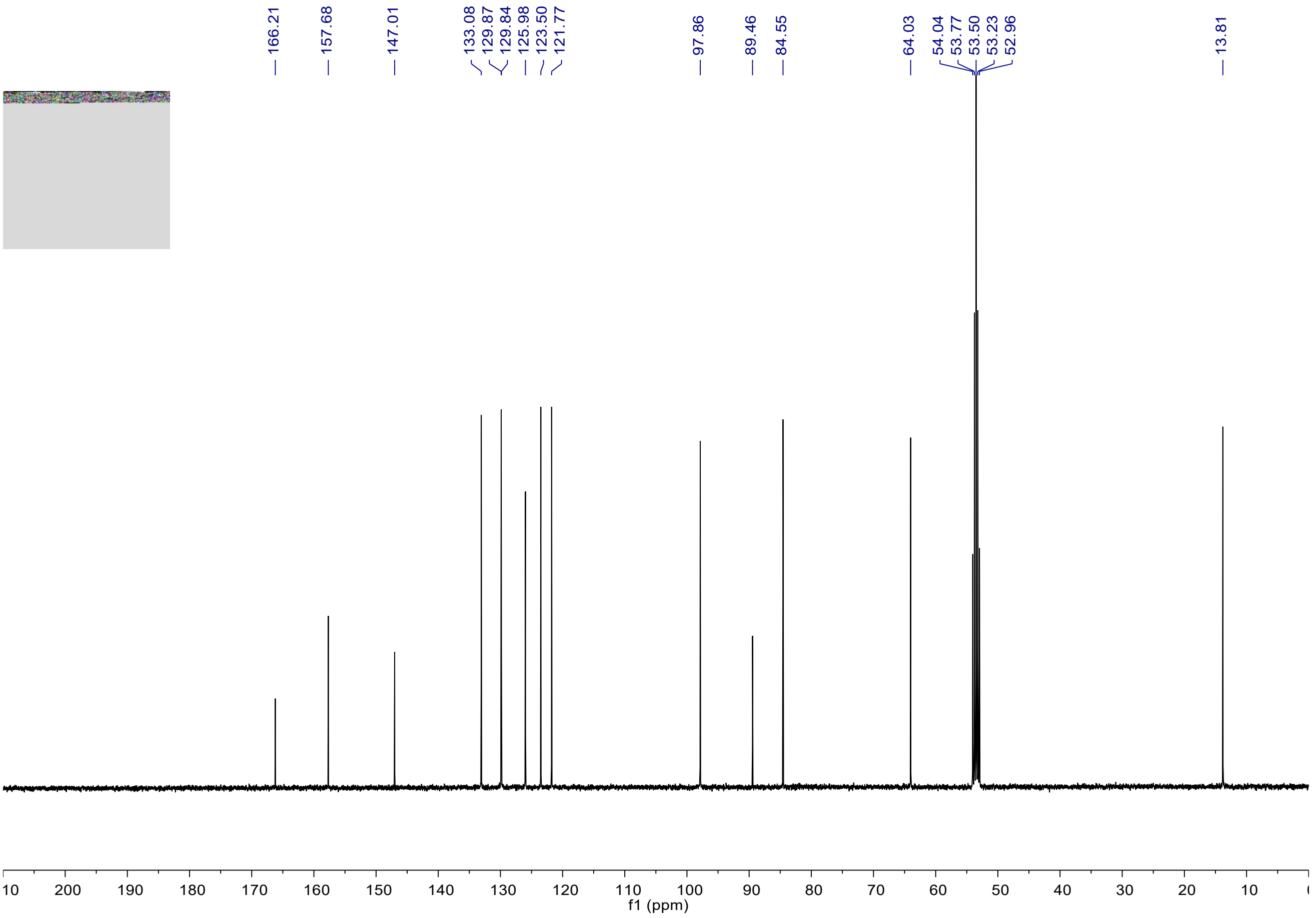

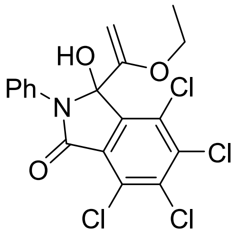

4,5,6,7-tetrachloro-3-(1-ethoxyvinyl)-3-hydroxy-2-phenylisoindolin-1-one (3hv). A white solid, 64 mg, 74% yield; M.p.: 158-160 °C; ^{1}H NMR ($CDCl_3$, 400 MHz, TMS) δ 7.24-7.12 (m, 5H), 5.02 (s, 1H), 4.69 (d, J = 2.9 Hz, 1H), 4.18 (d, J = 2.9 Hz, 1H), 3.61-3.48 (m, 1H), 3.48-3.35 (m, 1H), 0.99 (t, J = 7.0 Hz, 3H); ^{13}C NMR ($CDCl_3$, 100 MHz, TMS) δ 163.8, 153.7, 143.0, 138.0, 135.4, 133.9, 128.7, 128.6, 128.3, 127.5, 127.1, 127.1, 90.0, 87.8, 63.6, 14.0; IR (neat) ν 3345, 2921, 1690, 1495, 1391, 1350, 1288, 1219, 1180, 1123, 1057, 868, 734, 689 cm^{-1}; HRMS (ESI) Calcd. for $C_{18}H_{13}NO_3NaCl_4^+$ Requires: 453.9542, Found: 453.9541.

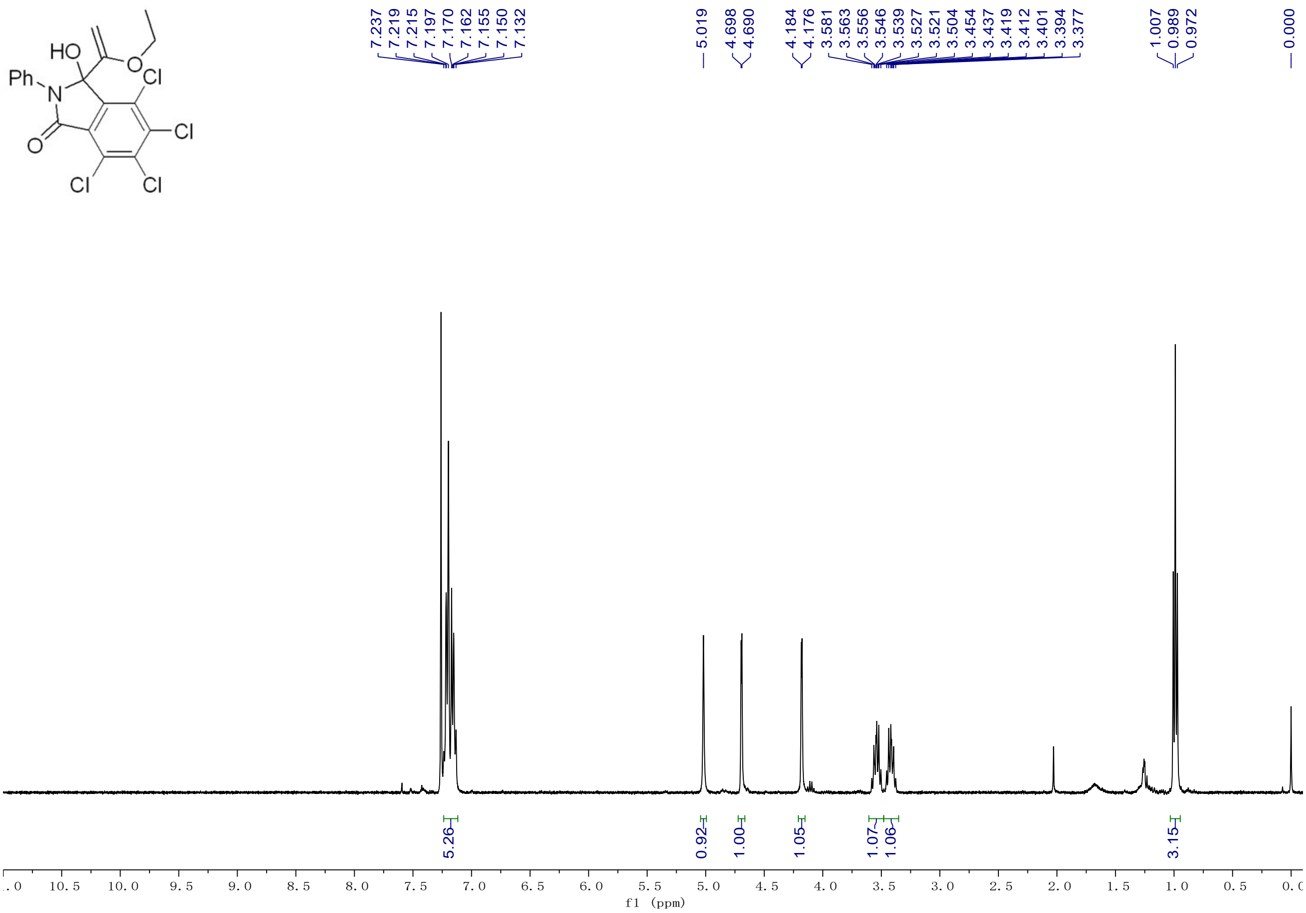
HO
Ph
N
O
Cl
Cl
Cl
Cl
O
7.237
7.219
7.215
7.197
7.170
7.162
7.155
7.150
7.132
5.019
4.698
4.690
4.184
4.176
3.581
3.563
3.556
3.546
3.539
3.527
3.521
3.504
3.454
3.437
3.419
3.412
3.401
3.394
3.377
1.007
0.989
0.972
0.000
5.26
0.92
1.00
1.05
1.07
1.06
3.15
f1 (ppm)

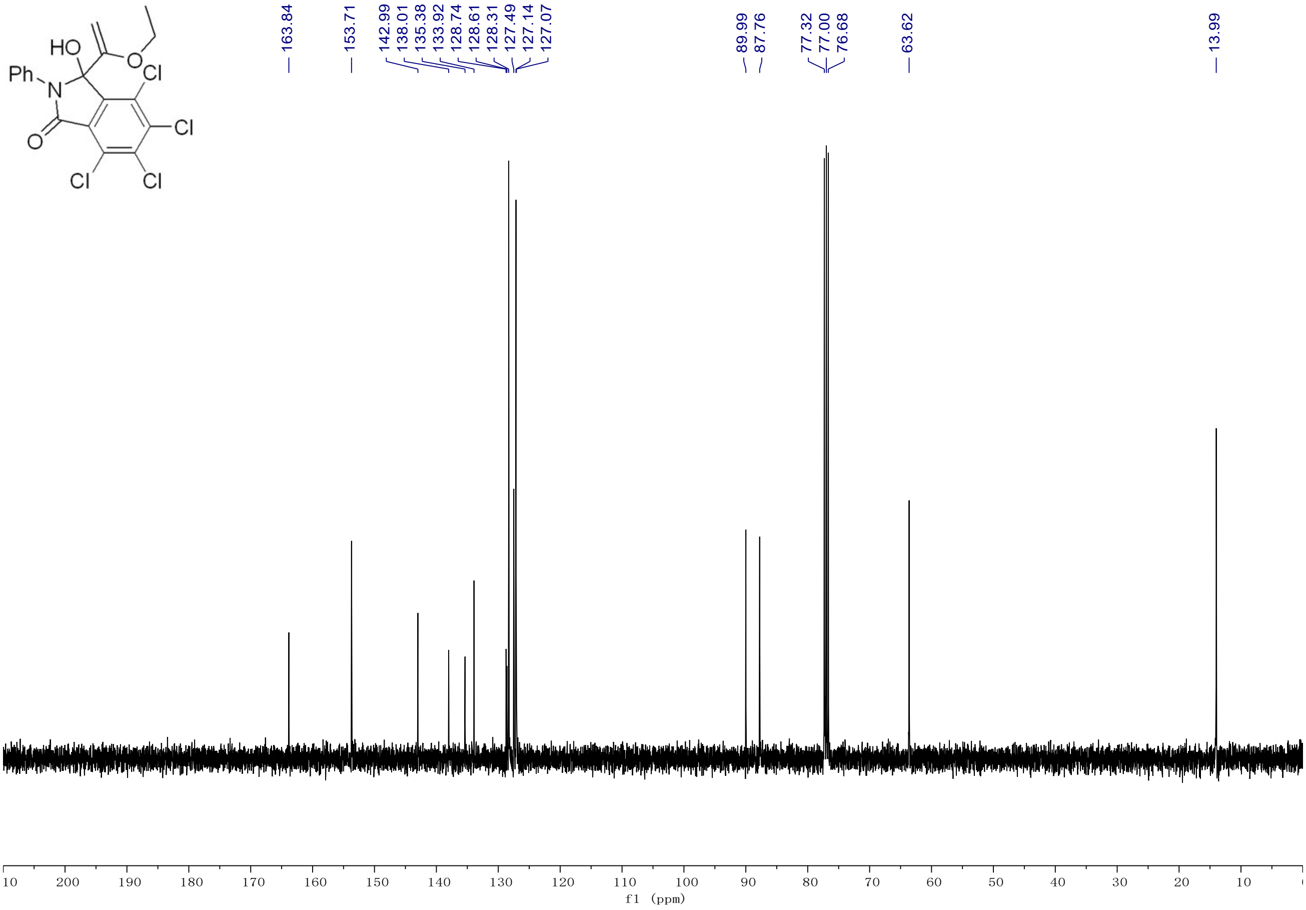
HO
Ph
N
O
Cl
Cl
Cl
Cl
O
163.84
153.71
142.99
138.01
135.38
133.92
128.74
128.61
128.31
127.49
127.14
127.07
89.99
87.76
77.32
77.00
76.68
63.62
13.99
f1 (ppm)

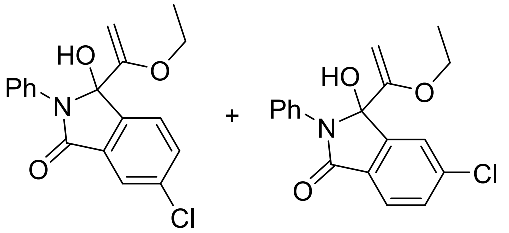

A mixture of 6-chloro-3-(1-ethoxyvinyl)-3-hydroxy-2-phenylisoindolin-1-one and 5-chloro-3-(1-ethoxyvinyl)-3-hydroxy-2-phenylisoindolin-1-one (3hw). A white solid, 49 mg, 75% yield; M.p.: 146-148 °C; ^{1}H NMR (Acetone-d_6, 400 MHz) δ 7.78-7.68 (m, 1H), 7.68-7.57 (m, 3H), 7.57-7.49 (m, 1H), 7.38 (t, J = 7.8 Hz, 2H), 7.30-7.21 (m, 1H), 6.45-6.36 (m, 1H), 5.03-4.87 (m, 1H), 4.36-4.24 (m, 1H), 3.66-3.46 (m, 2H), 1.00-0.93 (m, 3H); ^{13}C NMR (Acetone-d_6, 400 MHz) δ 165.7, 165.3, 158.05, 157.96, 149.0, 145.6, 138.0, 136.73, 136.69, 135.0, 133.7, 132.5, 130.4, 129.9, 128.20, 128.18, 126.3, 126.2, 126.09, 126.06, 124.6, 123.8, 122.6, 122.2, 90.8, 90.5, 85.1, 85.0, 63.30, 63.27, 13.4; IR (neat) ν 3297, 2989, 1677, 1601, 1496, 1420, 1363, 1127, 1095, 1067, 978, 894, 815, 749, 693 cm^{-1}; HRMS (ESI) Calcd. for $C_{18}H_{16}NO_3NaCl^+$ Requires: 352.0711, Found: 352.0714.

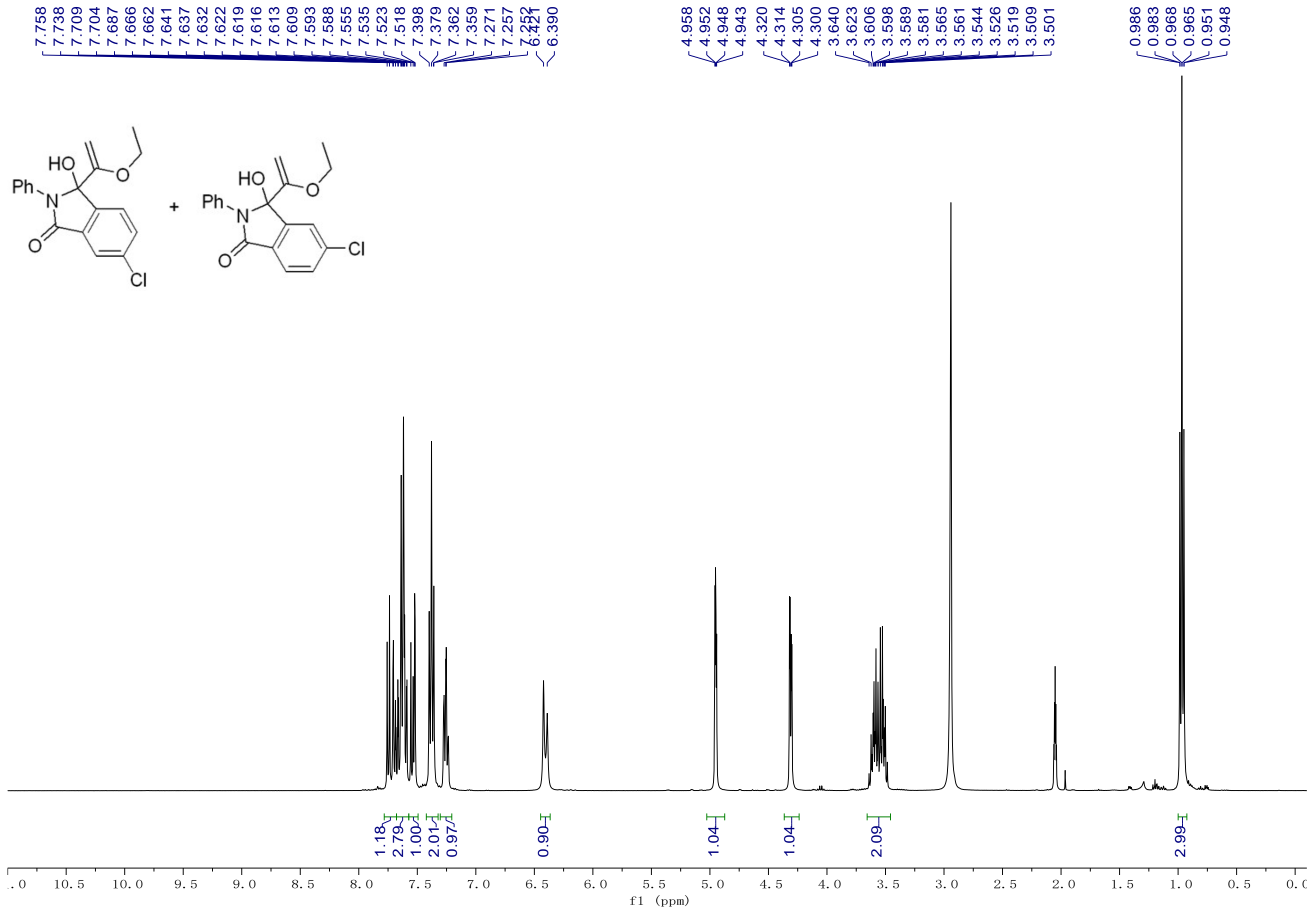

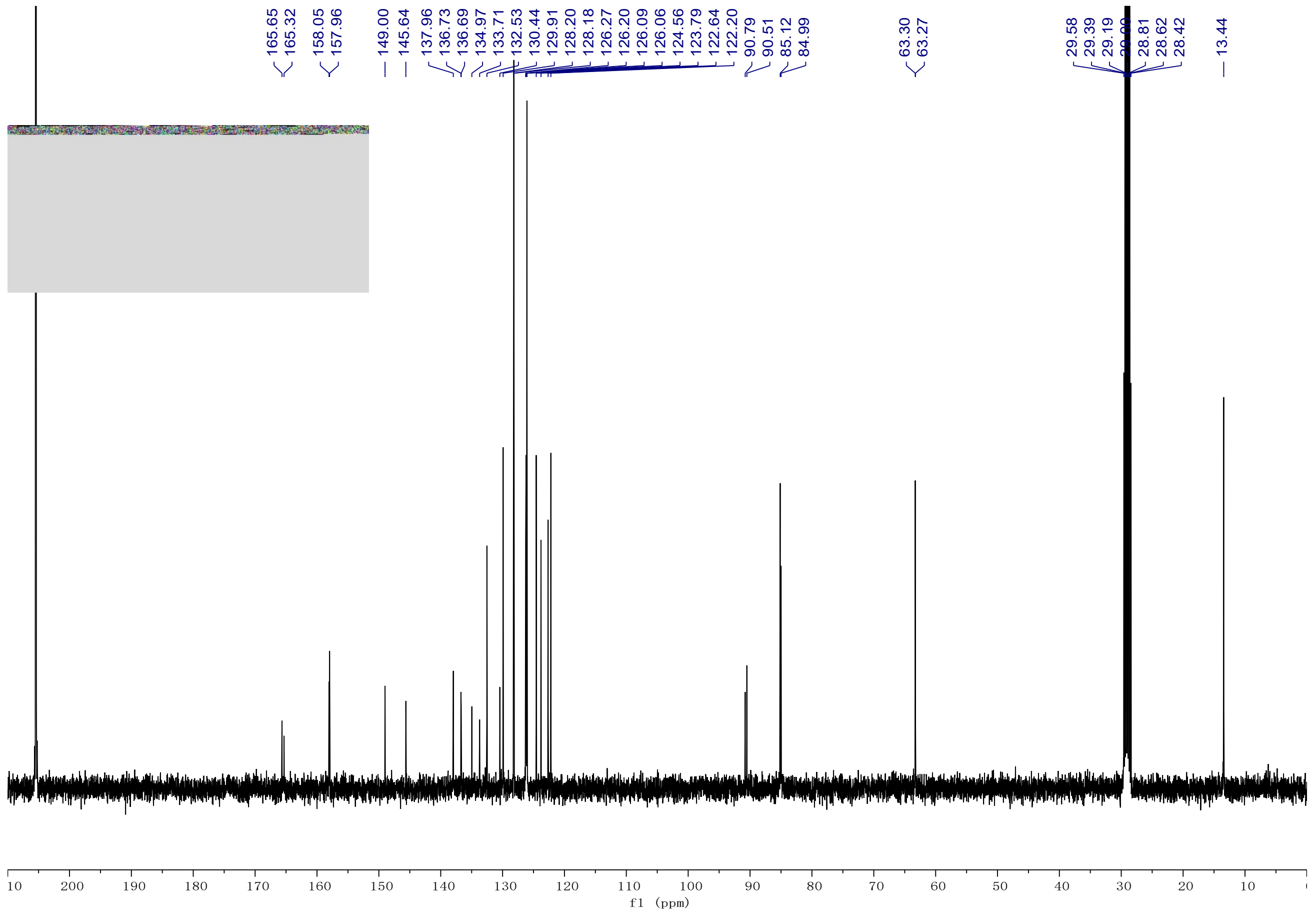

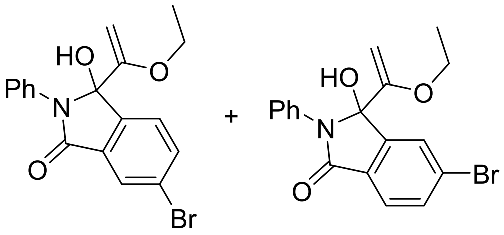

A mixture of 6-bromo-3-(1-ethoxyvinyl)-3-hydroxy-2-phenylisoindolin-1-one and 5-bromo-3-(1-ethoxyvinyl)-3-hydroxy-2-phenylisoindolin-1-one (3hx). A white solid, 54 mg, 72% yield; M.p.: 138-140 °C; ^{1}H NMR (Acetone-d_6, 400 MHz) δ 7.88-7.72 (m, 3H), 7.71-7.59 (m, 6H), 7.49 (d, J = 8.0 Hz, 1H), 7.42-7.33 (m, 4H), 7.30-7.21 (m, 2H), 6.42-6.34 (m, 2H), 4.99-4.92 (m, 2H), 4.35-4.28 (m, 2H), 3.67-3.46 (m, 4H), 0.97 (t, J = 7.1 Hz, 6H); ^{13}C NMR (CD_2Cl_2, 100 MHz) δ 165.8, 165.2, 158.0, 157.9, 149.1, 146.1, 136.7, 136.6, 135.4, 133.9, 132.82, 130.83, 128.19, 128.18, 126.30, 126.27, 126.2, 126.1, 126.0, 125.7, 125.2, 124.8, 124.1, 122.9, 90.8, 90.5, 85.2, 85.0, 63.30, 63.27, 13.4; IR (neat) ν 3312, 2989, 1676, 1596, 1494, 1418, 1357, 1280, 1229, 1093, 1057, 976, 887, 816, 766, 738, 692 cm^{-1}; HRMS (ESI) Calcd. for $C_{18}H_{16}NO_3NaBr^+$ Requires: 396.0206, Found: 396.0204.

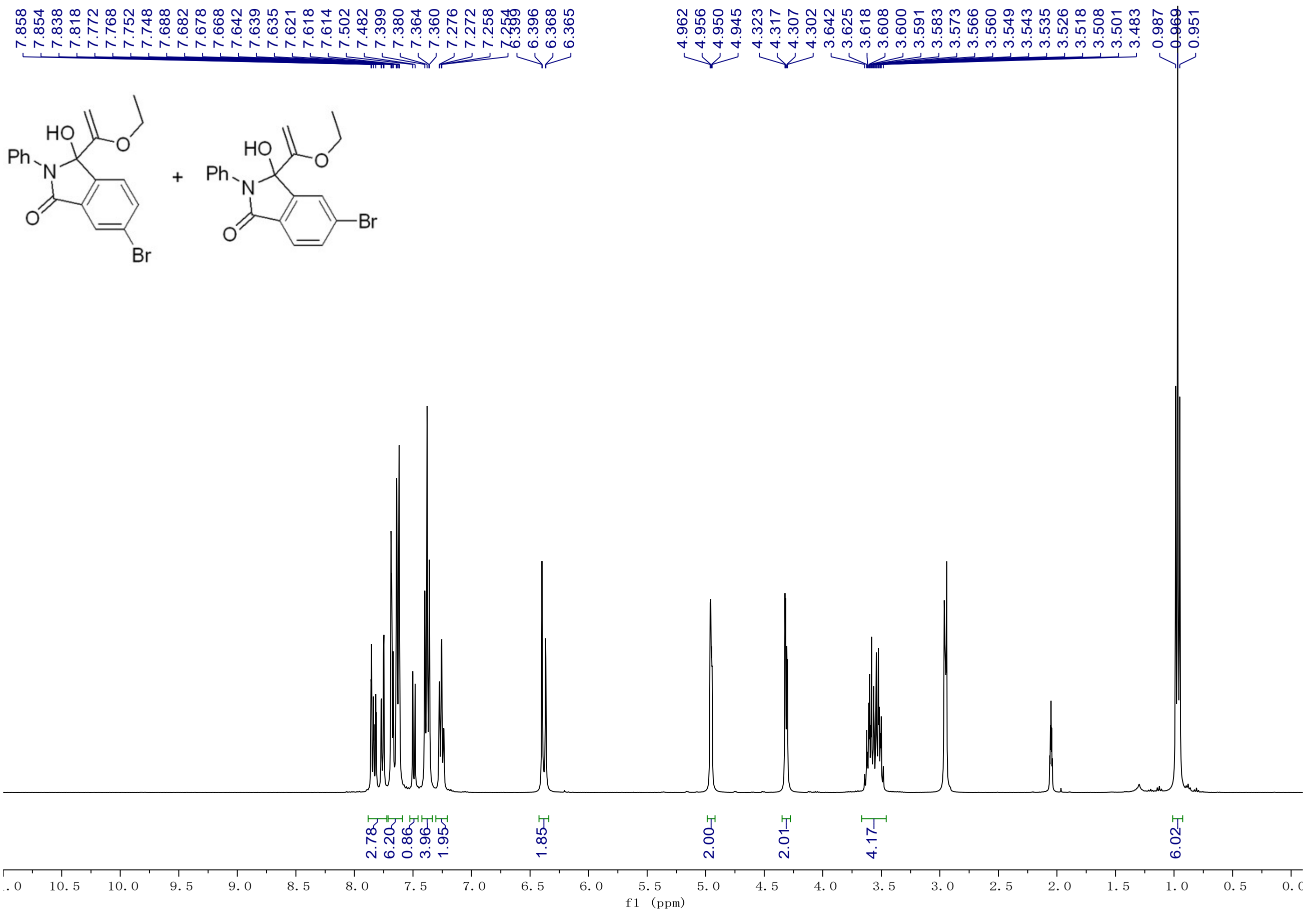

7.858
7.854
7.838
7.818
7.772
7.768
7.752
7.748
7.688
7.682
7.678
7.668
7.642
7.639
7.635
7.621
7.618
7.614
7.502
7.482
7.399
7.380
7.364
7.360
7.276
7.272
7.258
6.396
6.368
6.365
4.962
4.956
4.950
4.945
4.323
4.317
4.307
4.302
3.642
3.625
3.618
3.608
3.600
3.591
3.583
3.573
3.566
3.560
3.549
3.543
3.535
3.526
3.518
3.508
3.501
3.483
0.987
0.951
HO
O
Ph
N
O
Br
+
HO
O
Ph
N
O
Br
2.78
6.20
0.86
3.96
1.95
1.85
2.00
2.01
4.17
6.02
f1 (ppm)

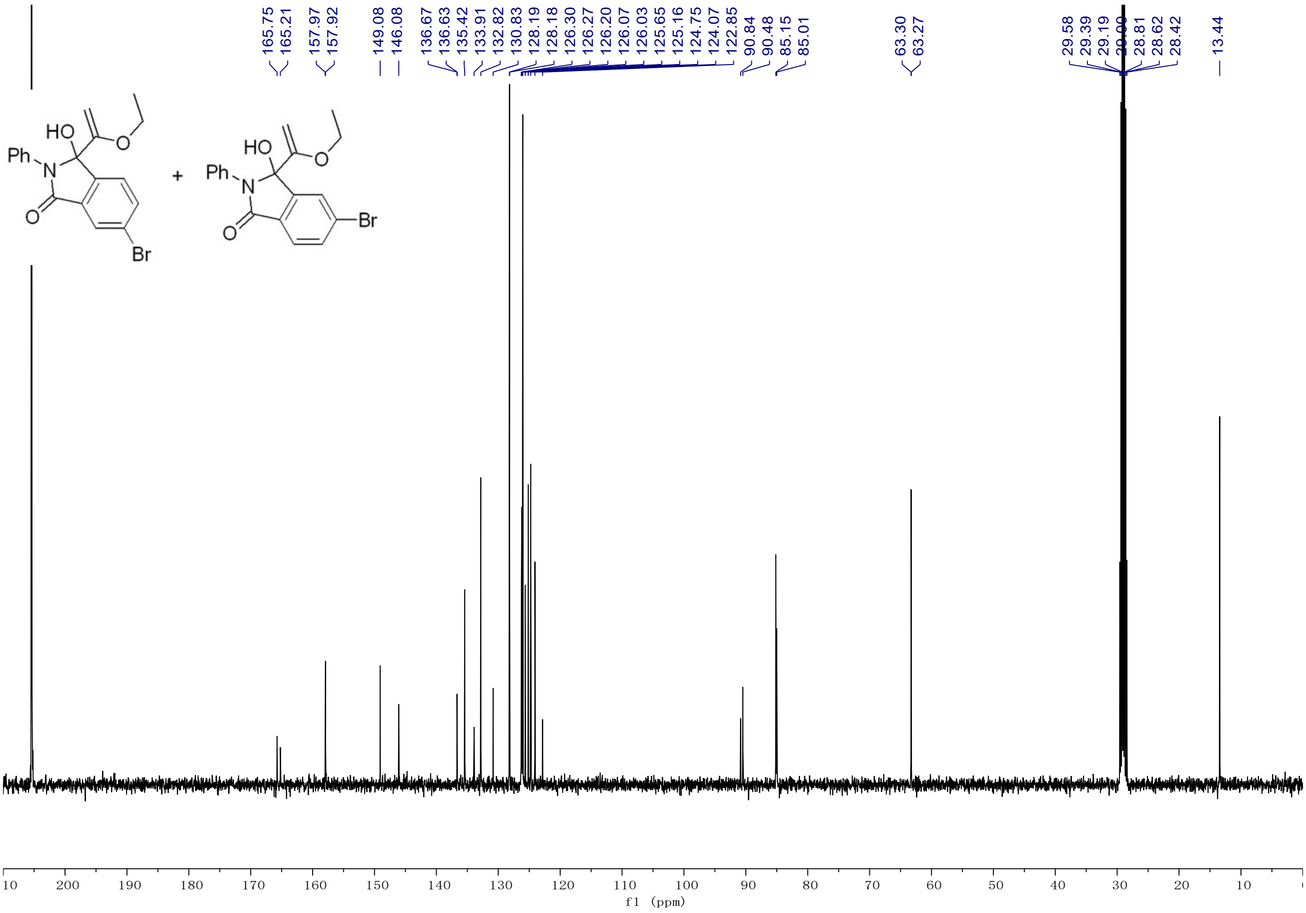

165.75
165.21
157.97
157.92
149.08
146.08
136.67
136.63
135.42
133.91
132.82
130.83
128.19
128.18
126.30
126.27
126.20
126.07
126.03
125.65
125.16
124.75
124.07
122.85
90.84
90.48
85.15
85.01
63.30
63.27
29.58
29.39
29.19
28.81
28.62
28.42
13.44
HO
O
Ph
N
O
Br
+
HO
O
Ph
N
O
Br
f1 (ppm)

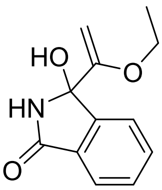

3-(1-ethoxyvinyl)-3-hydroxyisoindolin-1-one (3hy). A white solid, 23 mg, 53% yield; M.p.: 130-131 °C; ^{1}H NMR (CD_2Cl_2, 400 MHz, TMS) δ 7.51-7.42 (m, 3H), 7.39-7.29 (m, 1H), 7.27 (s, 1H), 4.61 (s, 1H), 4.45 (d, J = 2.8 Hz, 1H), 4.01 (d, J = 2.9 Hz, 1H), 3.74-3.60 (m, 2H), 1.15 (t, J = 6.9 Hz, 3H); ^{13}C NMR (CD_2Cl_2, 100 MHz, TMS) δ 169.8, 159.5, 147.5, 132.7, 130.6, 129.6, 123.3, 122.9, 86.7, 82.6, 64.1, 14.0; IR (neat) ν 3283, 2985, 1685, 1664, 1612, 1469, 1321, 1267, 1209, 1102, 1065, 979, 955, 815, 767, 703 cm^{-1}; HRMS (ESI) Calcd. for $C_{12}H_{13}NO_3Na^+$ Requires: 242.0788, Found: 242.0788.

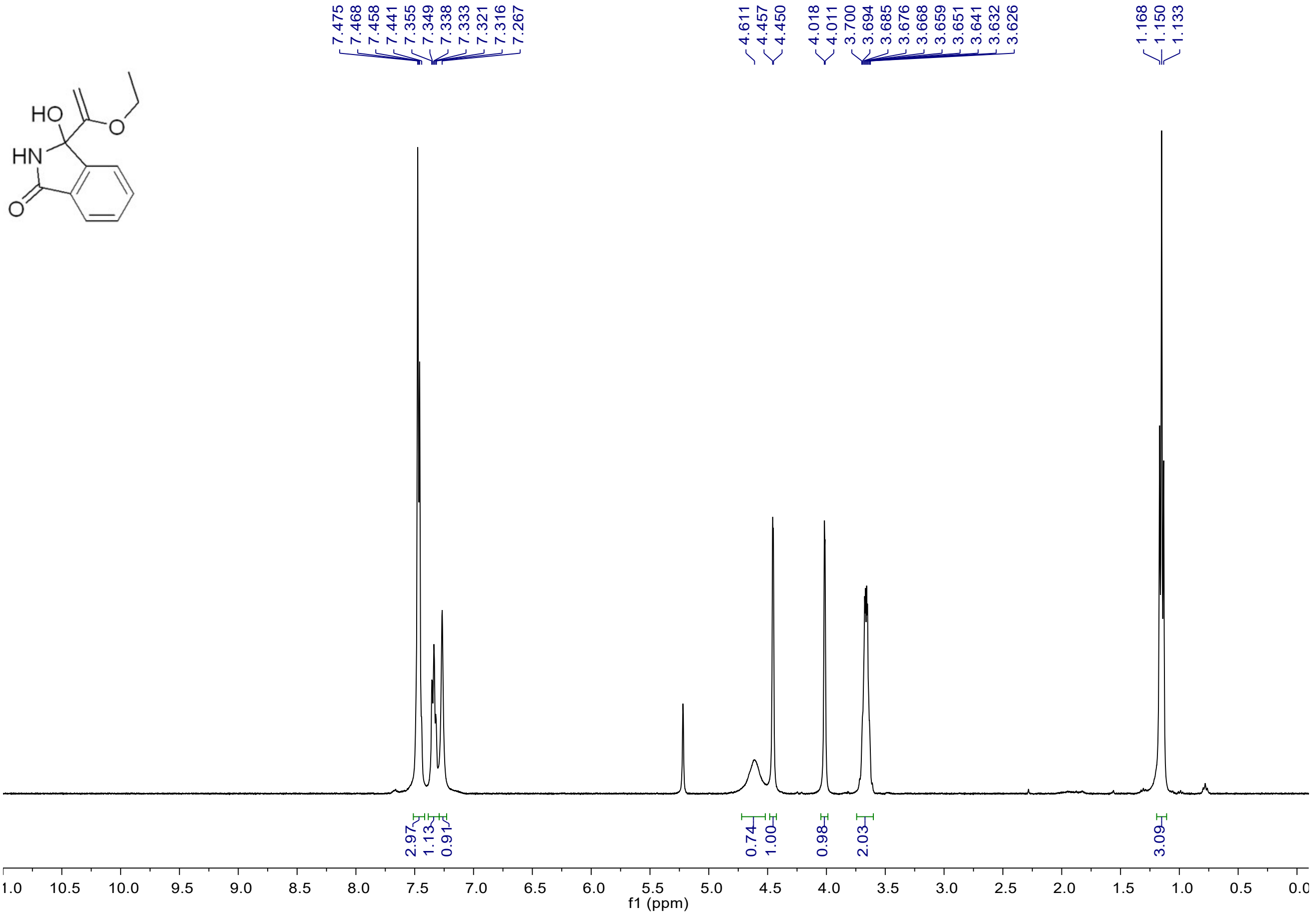

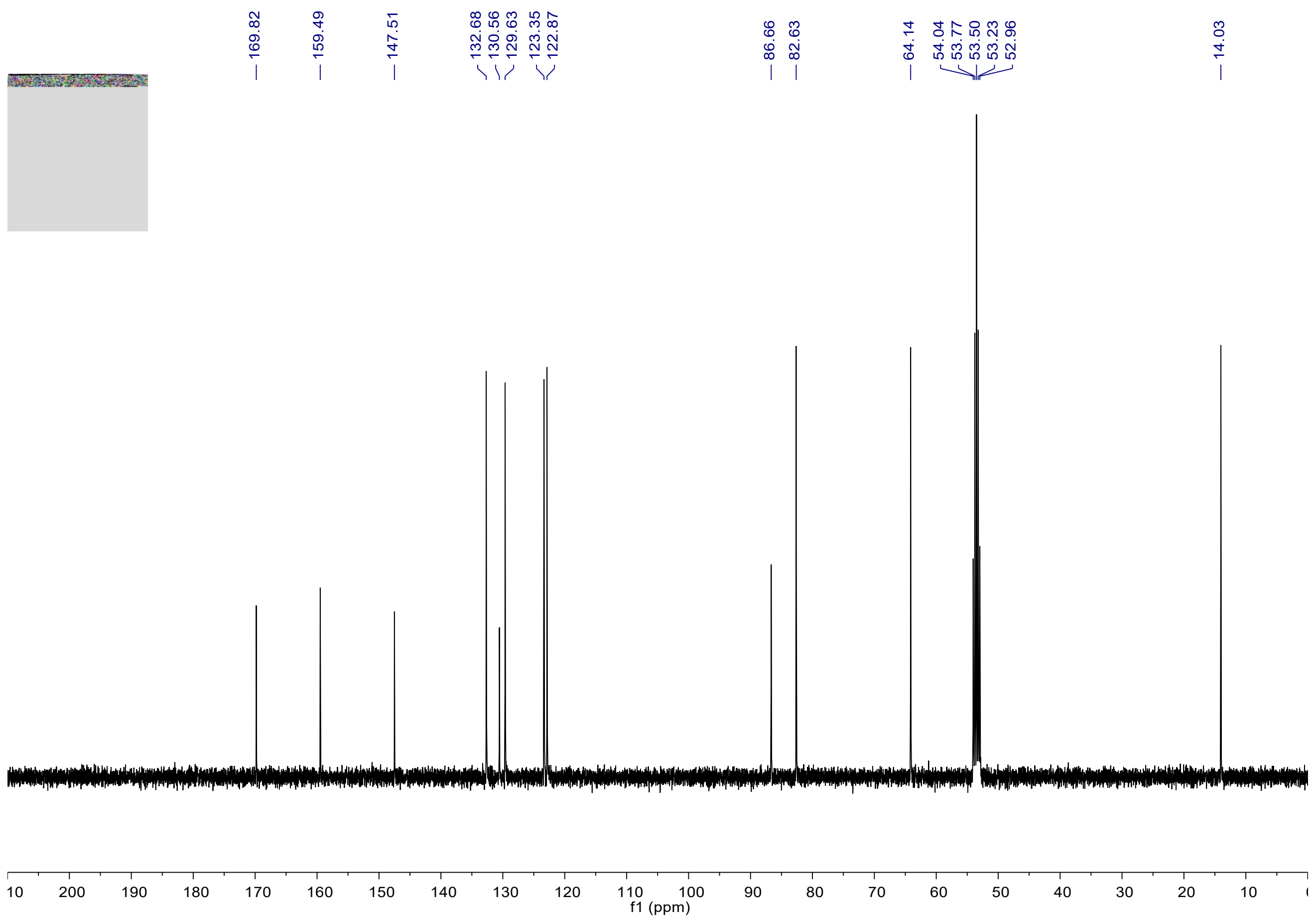

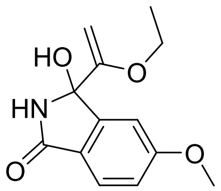

3-(1-ethoxyvinyl)-3-hydroxy-5-methoxyisoindolin-1-one (3hz). A white solid, 12 mg, 24% yield; M.p.: 129-131 °C; ^{1}H NMR (Acetone-d_6, 400 MHz) δ 7.69 (s, 1H), 7.53 (d, J = 8.3 Hz, 1H), 7.07 (d, J = 2.3 Hz, 1H), 7.02 (dd, J = 8.3, 2.3 Hz, 1H), 5.60 (s, 1H), 4.73 (d, J = 2.2 Hz, 1H), 4.17 (d, J = 2.2 Hz, 1H), 3.87 (s, 3H), 3.79-3.69 (m, 2H), 1.17 (t, J = 7.0 Hz, 3H); ^{13}C NMR (Acetone-d_6, 100 MHz) δ 168.5, 163.3, 160.9, 151.0, 124.1, 124.0, 115.3, 107.5, 85.7, 81.8, 63.4, 55.2, 13.6; IR (neat) ν 3333, 3172, 2983, 1697, 1615, 1435, 1354, 1273, 1249, 1100, 1051, 1024, 975, 788, 747, 699 cm^{-1}; HRMS (ESI) Calcd. for $C_{13}H_{15}NO_4Na^+$ Requires: 272.0893, Found: 272.0901.

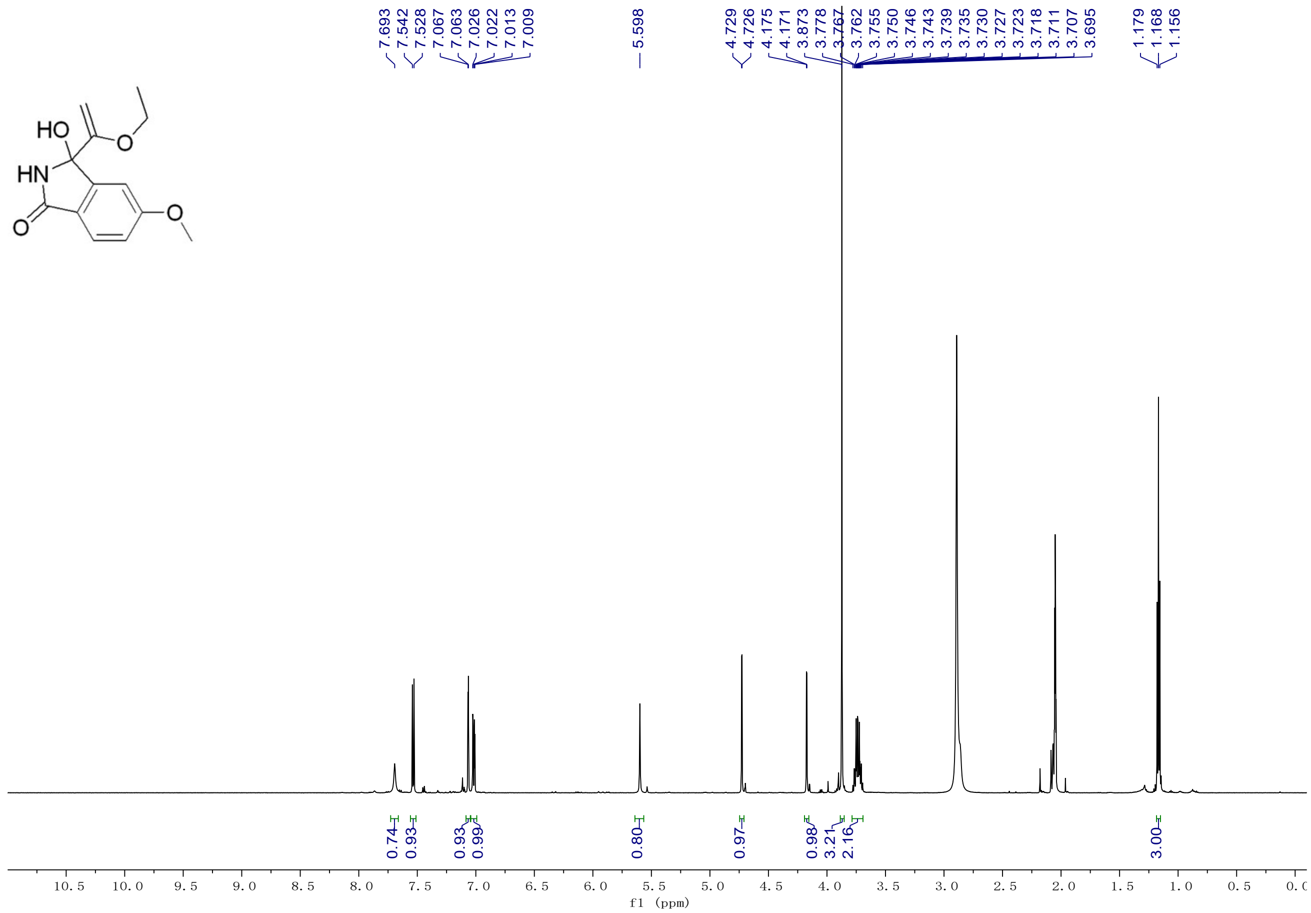
7.693
7.542
7.528
7.067
7.063
7.026
7.022
7.013
7.009
5.598
4.729
4.726
4.175
4.171
3.873
3.778
3.762
3.755
3.750
3.746
3.743
3.739
3.735
3.730
3.727
3.723
3.718
3.711
3.707
3.695
1.179
1.168
1.156
HO
HN
O
O
O
0.74
0.93
0.93
0.99
0.80
0.97
0.98
3.21
2.16
3.00
10.5 10.0 9.5 9.0 8.5 8.0 7.5 7.0 6.5 6.0 5.5 5.0 4.5 4.0 3.5 3.0 2.5 2.0 1.5 1.0 0.5
f1 (ppm)

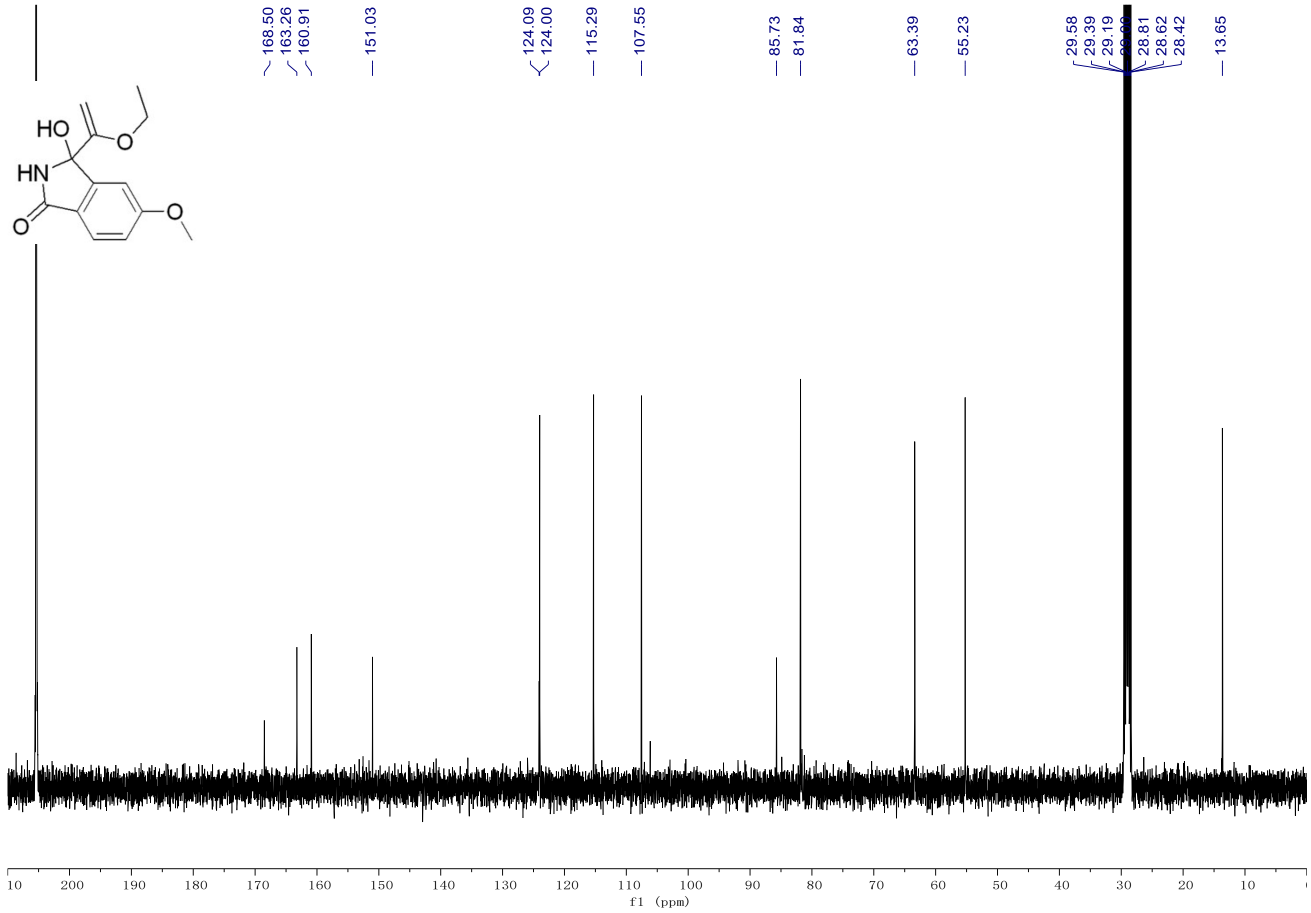
168.50
163.26
160.91
151.03
124.09
124.00
115.29
107.55
85.73
81.84
63.39
55.23
29.58
29.39
29.19
28.81
28.62
28.42
13.65
HO
HN
O
O
O
200 190 180 170 160 150 140 130 120 110 100 90 80 70 60 50 40 30 20 10
f1 (ppm)

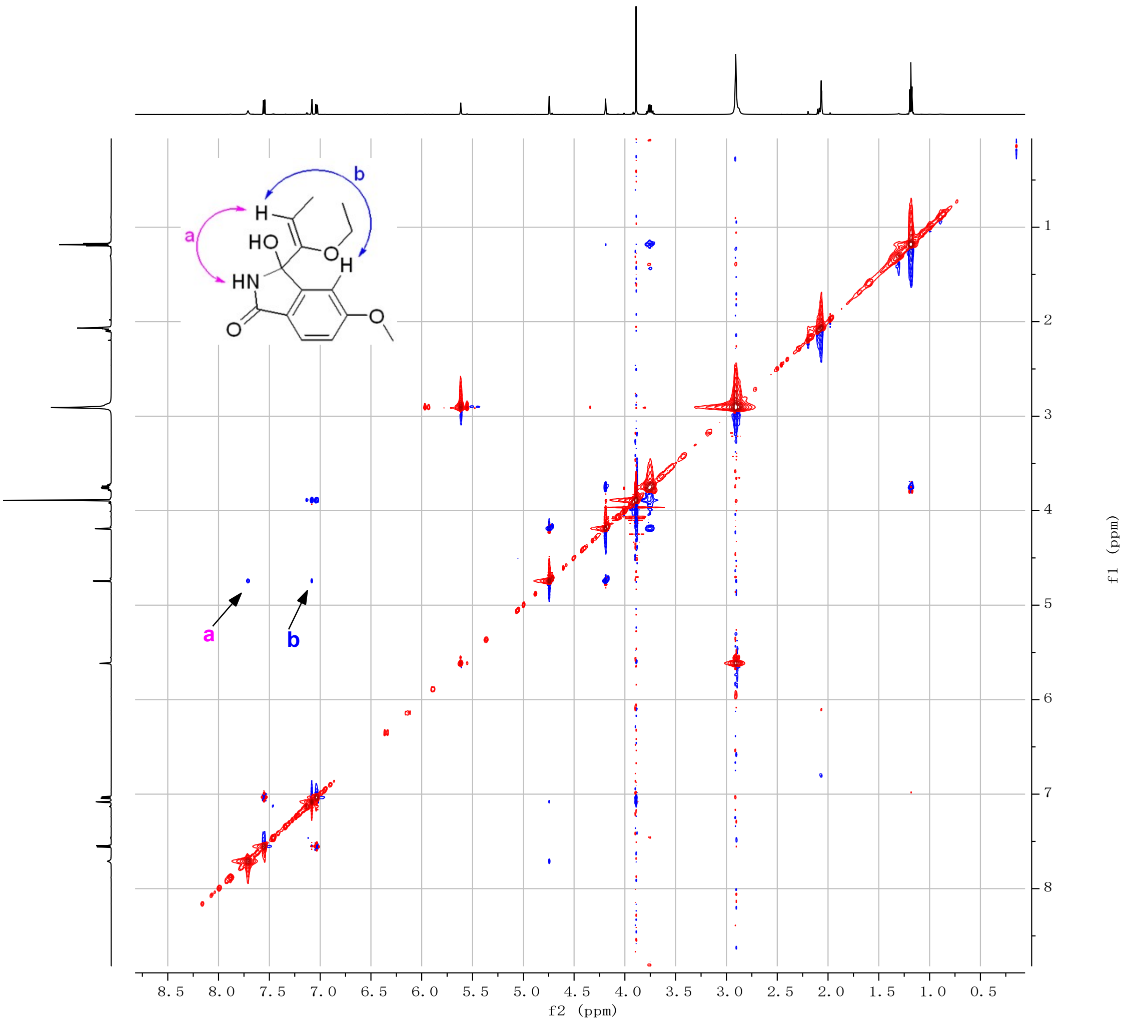

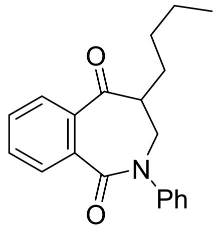

4-butyl-2-phenyl-3,4-dihydro-1H-benzo[c]azepine-1,5(2H)-dione (4). A white solid, 35 mg, 56% yield; M.p.: 69-70 °C; ^{1}H NMR (CD_2Cl_2, 400 MHz, TMS) δ 7.92 (dd, J = 7.4, 1.7 Hz, 1H), 7.74-7.60 (m, 2H), 7.54 (dd, J = 7.4, 1.7 Hz, 1H), 7.48 (t, J = 7.7 Hz, 2H), 7.39-7.31 (m, 3H), 4.09 (dd, J = 15.2, 10.2 Hz, 1H), 3.87 (dd, J = 15.1, 3.8 Hz, 1H), 3.13-3.01 (m, 1H), 1.77-1.64 (m, 1H), 1.60-1.46 (m, 1H), 1.33-1.20 (m, 4H), 0.84 (t, J = 6.9 Hz, 3H); ^{13}C NMR (CD_2Cl_2, 100 MHz, TMS) δ 206.0, 168.3, 142.8, 136.8, 133.5, 132.0, 131.8, 129.9, 129.4, 127.5, 127.1, 126.6, 54.9, 52.0, 29.8, 28.8, 22.6, 13.5; IR (neat) ν 3059, 2952, 2867, 1683, 1633, 1566, 1492, 1409, 1343, 1266, 1231, 1201, 1086, 934 cm^{-1}; HRMS (ESI) Calcd. for $C_{20}H_{21}NO_3Na^+$ Requires: 308.1645, Found: 308.1641.

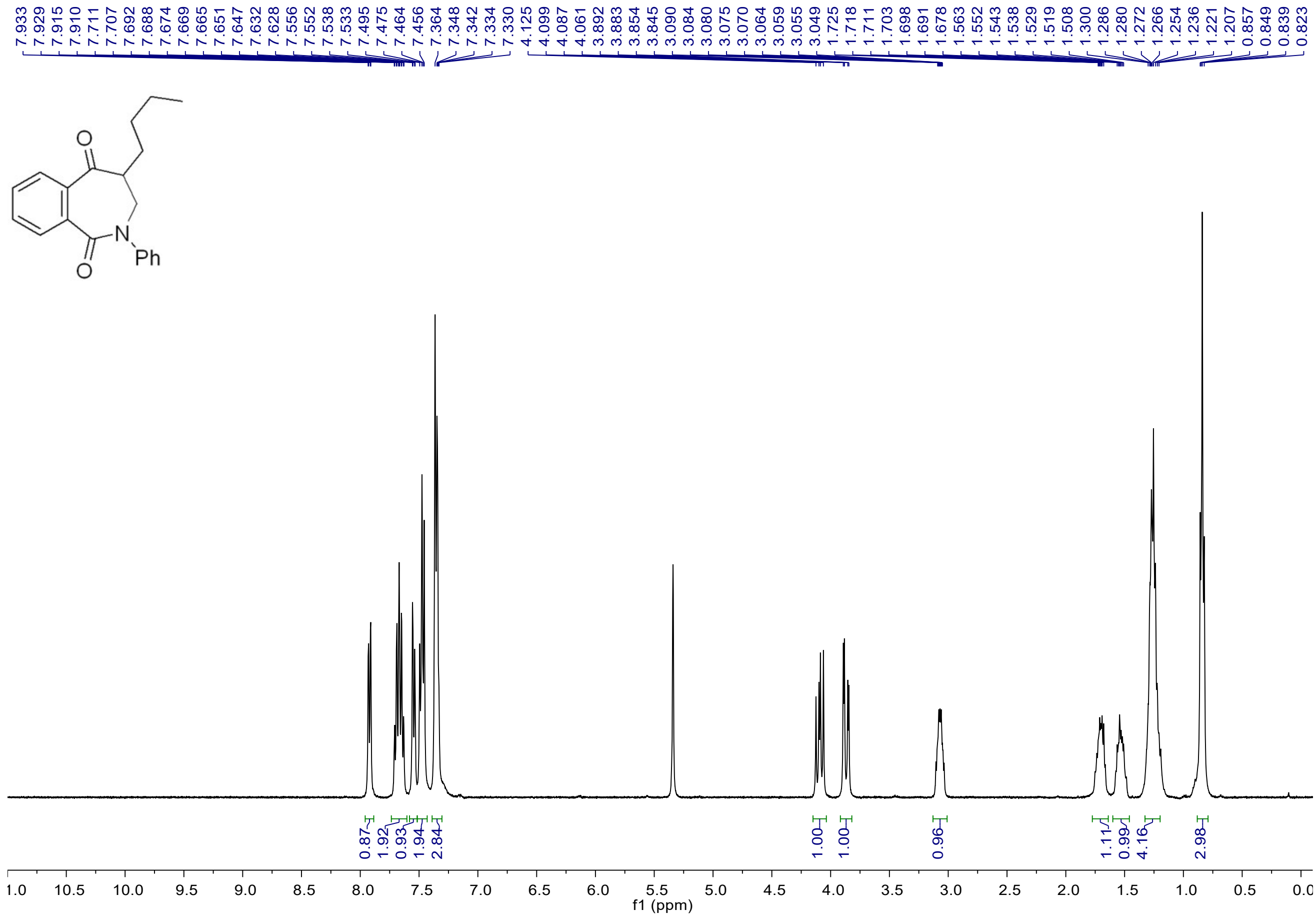
7.933 7.929 7.915 7.910 7.711 7.707 7.692 7.688 7.674 7.669 7.665 7.651 7.647 7.632 7.628 7.556 7.552 7.538 7.533 7.495 7.475 7.464 7.456 7.364 7.348 7.342 7.334 7.330 4.125 4.099 4.087 4.061 3.892 3.883 3.854 3.845 3.090 3.084 3.080 3.075 3.070 3.064 3.059 3.055 3.049 1.725 1.718 1.711 1.703 1.698 1.691 1.678 1.563 1.552 1.543 1.538 1.529 1.519 1.508 1.300 1.286 1.280 1.272 1.266 1.254 1.236 1.221 1.207 0.857 0.849 0.839 0.823
O
N
Ph
O
0.87 1.92 0.93 1.94 2.84 1.00 1.00 0.96 1.11 0.99 4.16 2.98
f1 (ppm)
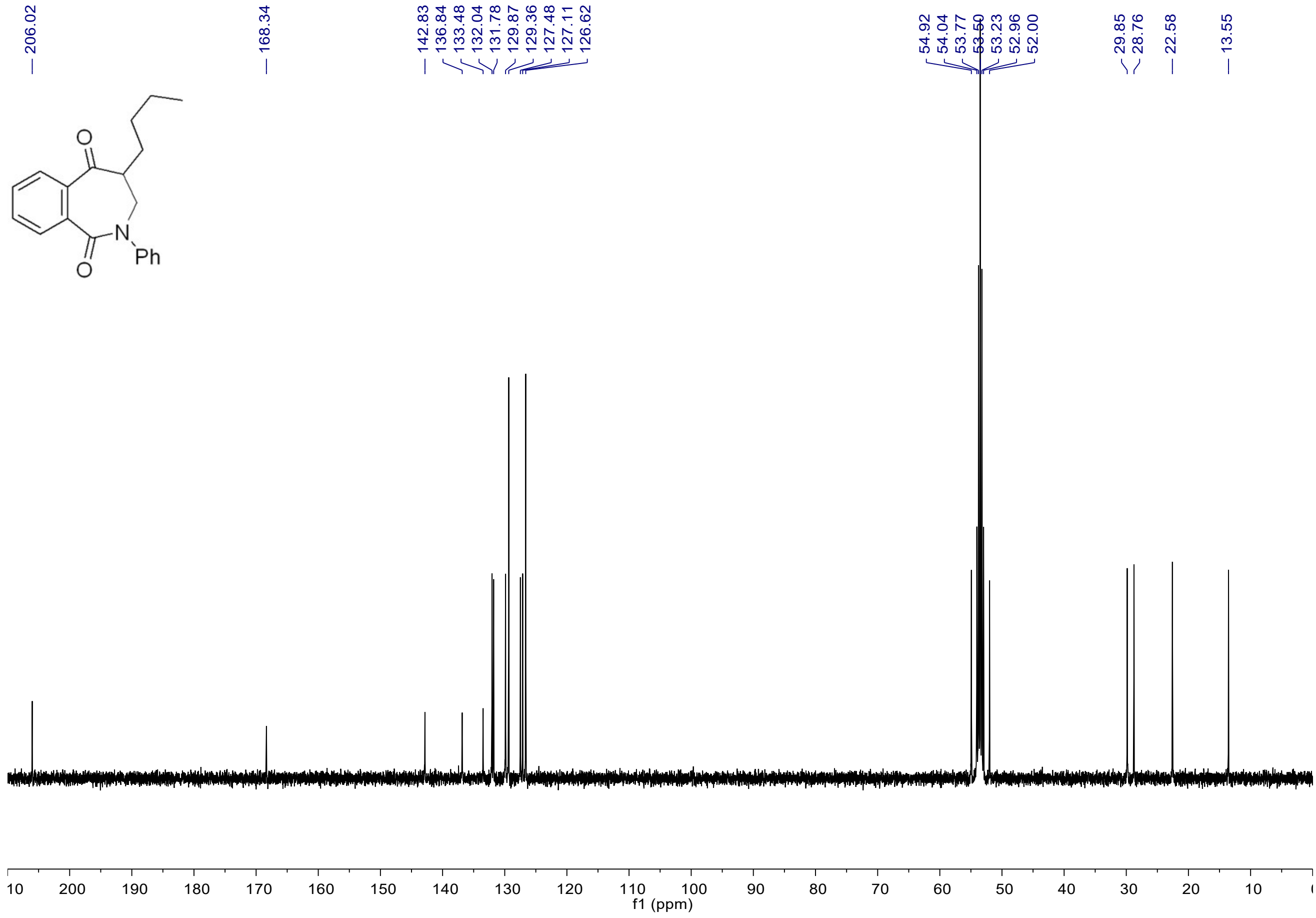
206.02 168.34 142.83 136.84 133.48 132.04 131.78 129.87 129.36 127.48 127.11 126.62 54.92 54.04 53.77 53.50 53.23 52.96 52.00 29.85 28.76 22.58 13.55
O
N
Ph
O
f1 (ppm)

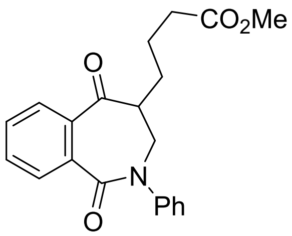

methyl 4-(1,5-dioxo-2-phenyl-2,3,4,5-tetrahydro-1H-benzo[c]azepin-4-yl)butanoate (S4). A colorless oil, 14 mg, 20% yield; ^{1}H NMR (CD_2Cl_2, 400 MHz, TMS) δ 7.86-7.77 (m, 1H), 7.64-7.49 (m, 2H), 7.49-7.41 (m, 1H), 7.41-7.31 (m, 2H), 7.29-7.20 (m, 3H), 4.00 (dd, *J* = 14.9, 10.4 Hz, 1H), 3.83-3.72 (m, 1H), 3.51 (s, 3H), 3.02-2.93 (m, 1H), 2.16 (t, *J* = 6.1 Hz, 2H), 1.60-1.41 (m, 4H); ^{13}C NMR (CD_2Cl_2, 100 MHz, TMS) δ 205.6, 173.2, 168.3, 142.7, 136.6, 133.5, 132.2, 131.8, 129.9, 129.4, 127.5, 127.2, 126.7, 54.6, 52.0, 51.4, 33.6, 29.5, 22.0; IR (neat) ν 2951, 2927, 2862, 1732, 1650, 1594, 1492, 1404, 1352, 1210, 1174, 1105, 1041, 883 cm^{-1}; HRMS (ESI) Calcd. for $C_{21}H_{21}NO_4Na^+$ Requires: 374.1363, Found: 374.1353.

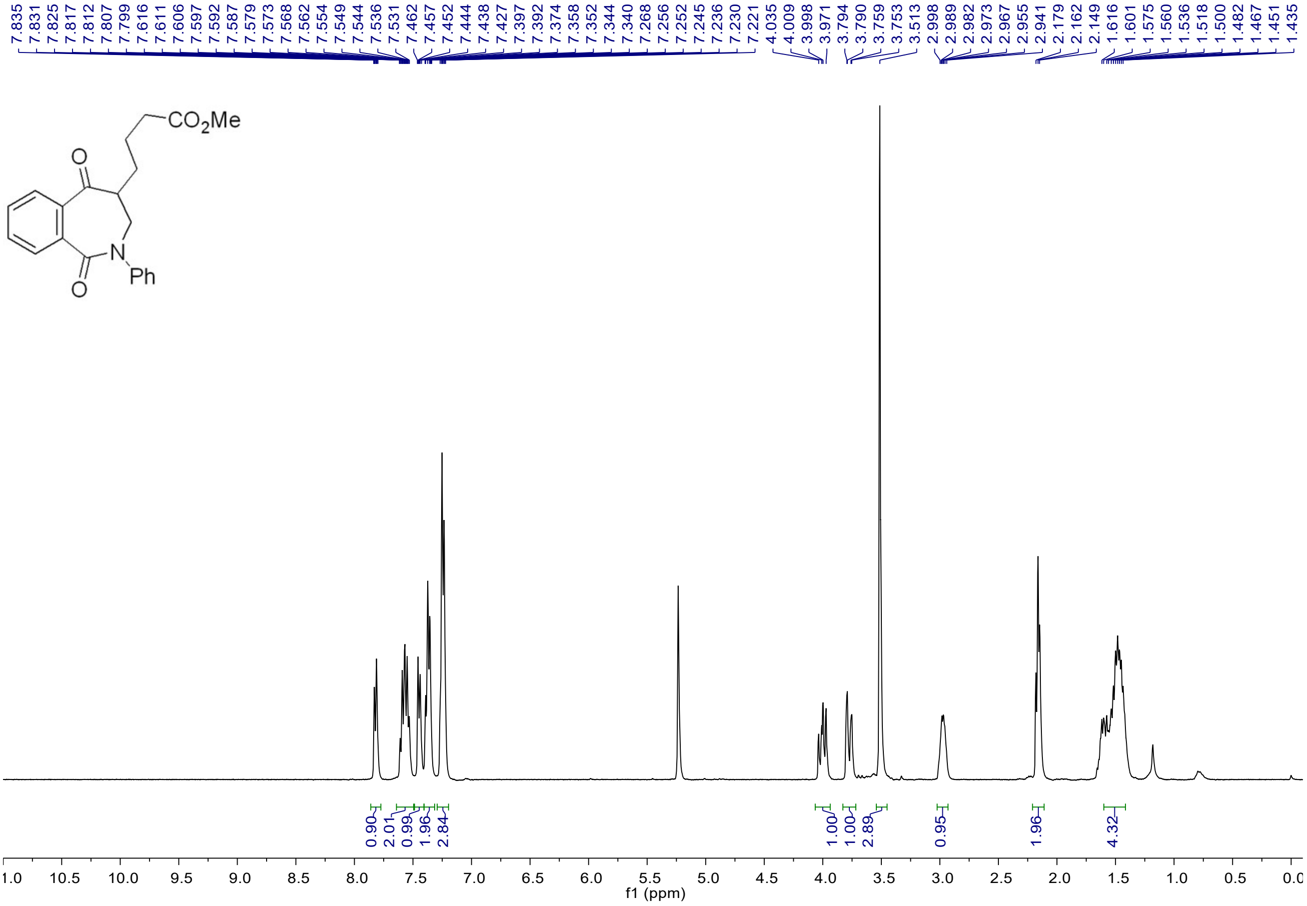

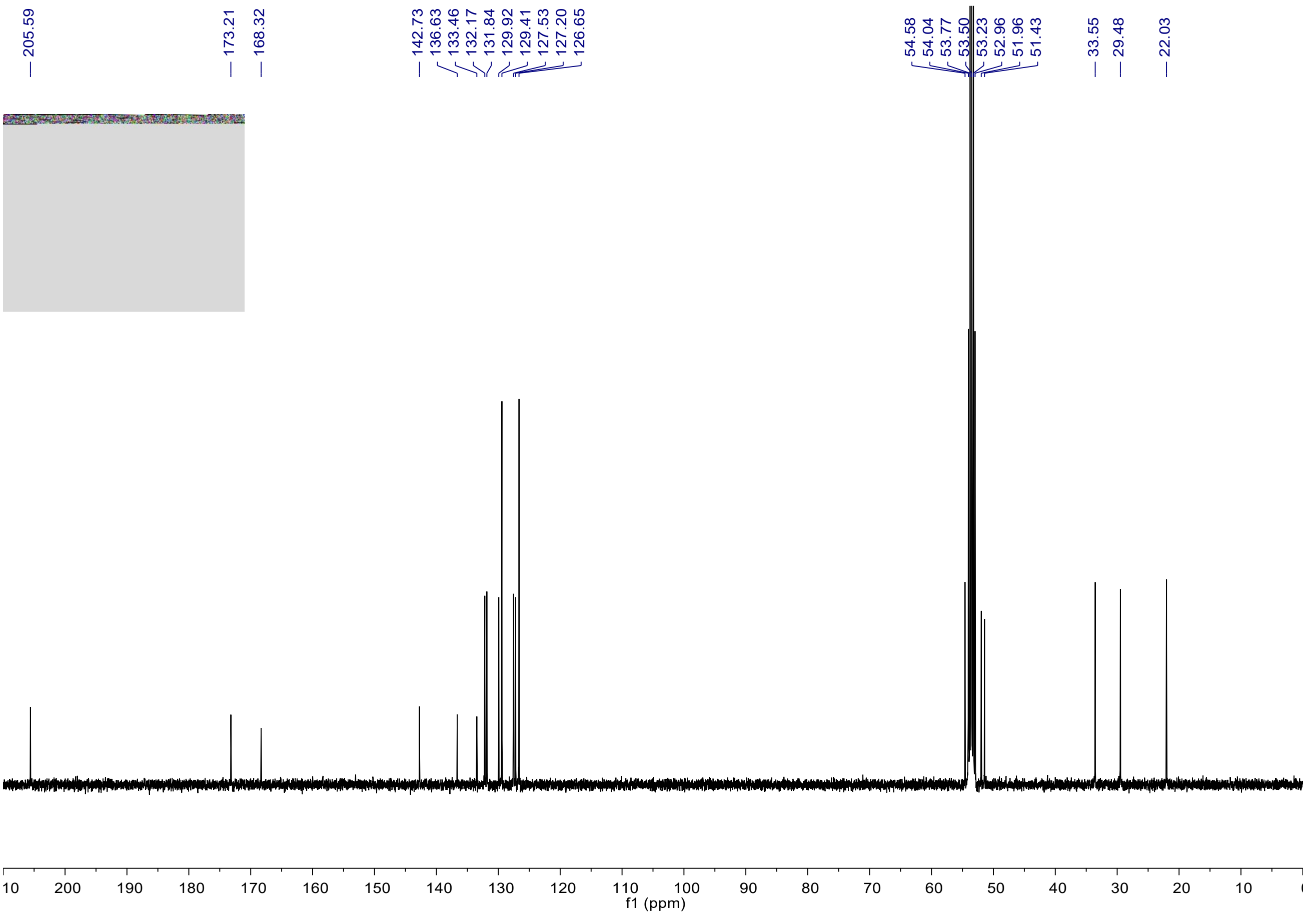

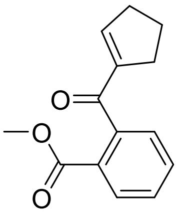

methyl 2-(cyclopent-1-ene-1-carbonyl)benzoate (7a). A colorless oil, 18 mg, 36% yield; ^{1}H NMR ($CDCl_3$, 400 MHz, TMS) δ 7.95 (dd, *J* = 7.7, 1.3 Hz, 1H), 7.62-7.53 (m, 1H), 7.53-7.45 (m, 1H), 7.35 (dd, *J* = 7.5, 1.3 Hz, 1H), 6.19-6.13 (m, 1H), 3.81 (s, 3H), 2.80-2.70 (m, 2H), 2.57-2.47 (m, 2H), 2.07-1.94 (m, 2H); ^{13}C NMR ($CDCl_3$, 100 MHz, TMS) δ 195.0, 166.7, 146.9, 146.6, 142.4, 132.0, 129.9, 129.3, 129.0, 127.3, 52.3, 34.0, 30.8, 23.0; IR (neat) ν 2948, 1722, 1654, 1433, 1359, 1275, 1125, 1079, 956, 772, 734, 711 cm^{-1}; HRMS (ESI) Calcd. for $C_{14}H_{14}O_3Na^+$ Requires: 253.0835, Found: 253.0829.

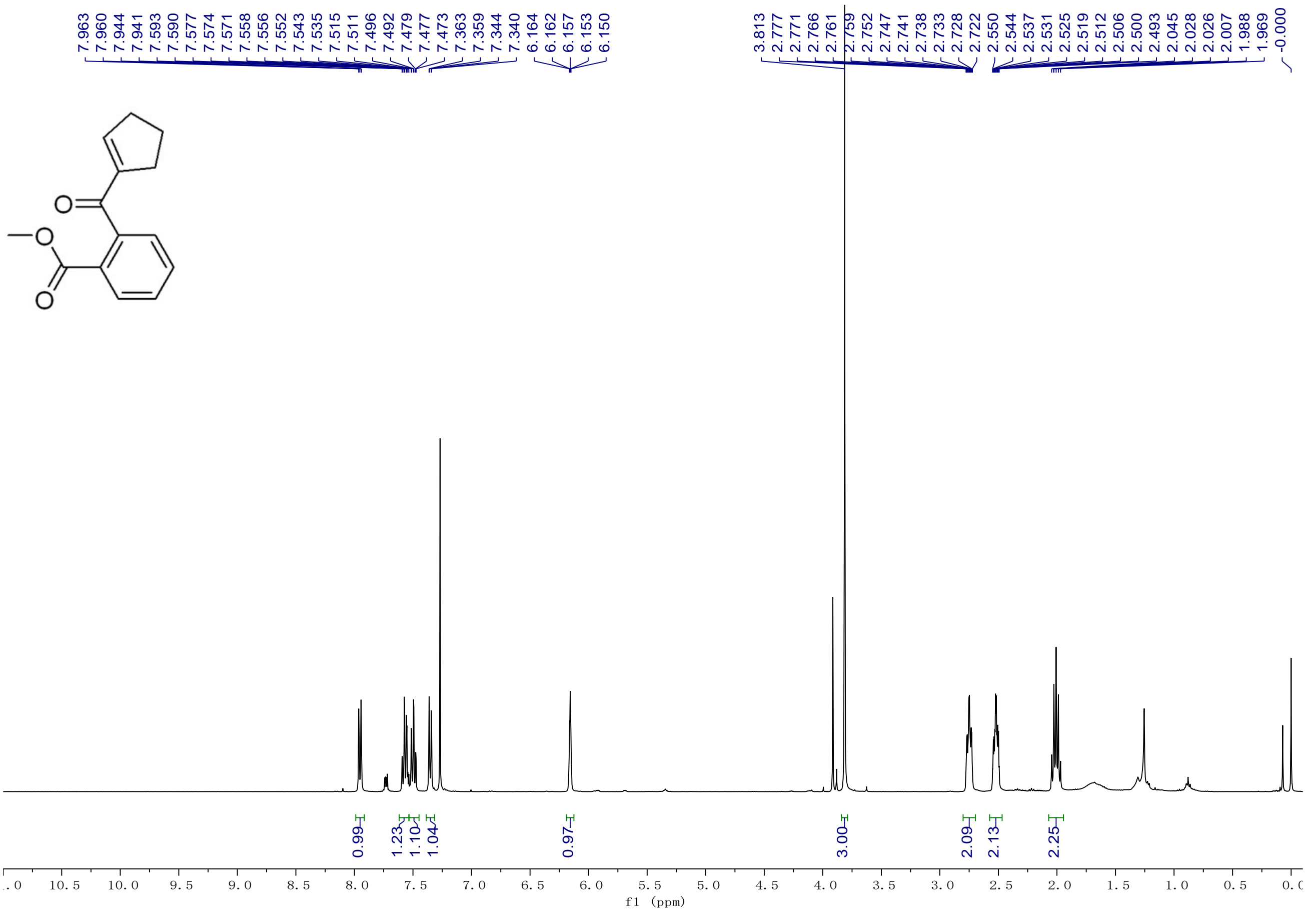
7.963 7.960 7.944 7.941 7.593 7.590 7.577 7.574 7.571 7.558 7.556 7.552 7.543 7.535 7.515 7.511 7.496 7.492 7.479 7.477 7.473 7.363 7.359 7.344 7.340 6.164 6.162 6.157 6.153 6.150
3.813 2.777 2.771 2.766 2.761 2.759 2.752 2.747 2.741 2.738 2.733 2.728 2.722 2.550 2.544 2.537 2.531 2.525 2.519 2.512 2.506 2.500 2.493 2.045 2.028 2.026 2.007 1.988 1.969 -0.000
0.99 1.23 1.10 1.04 0.97 3.00 2.09 2.13 2.25
f1 (ppm)

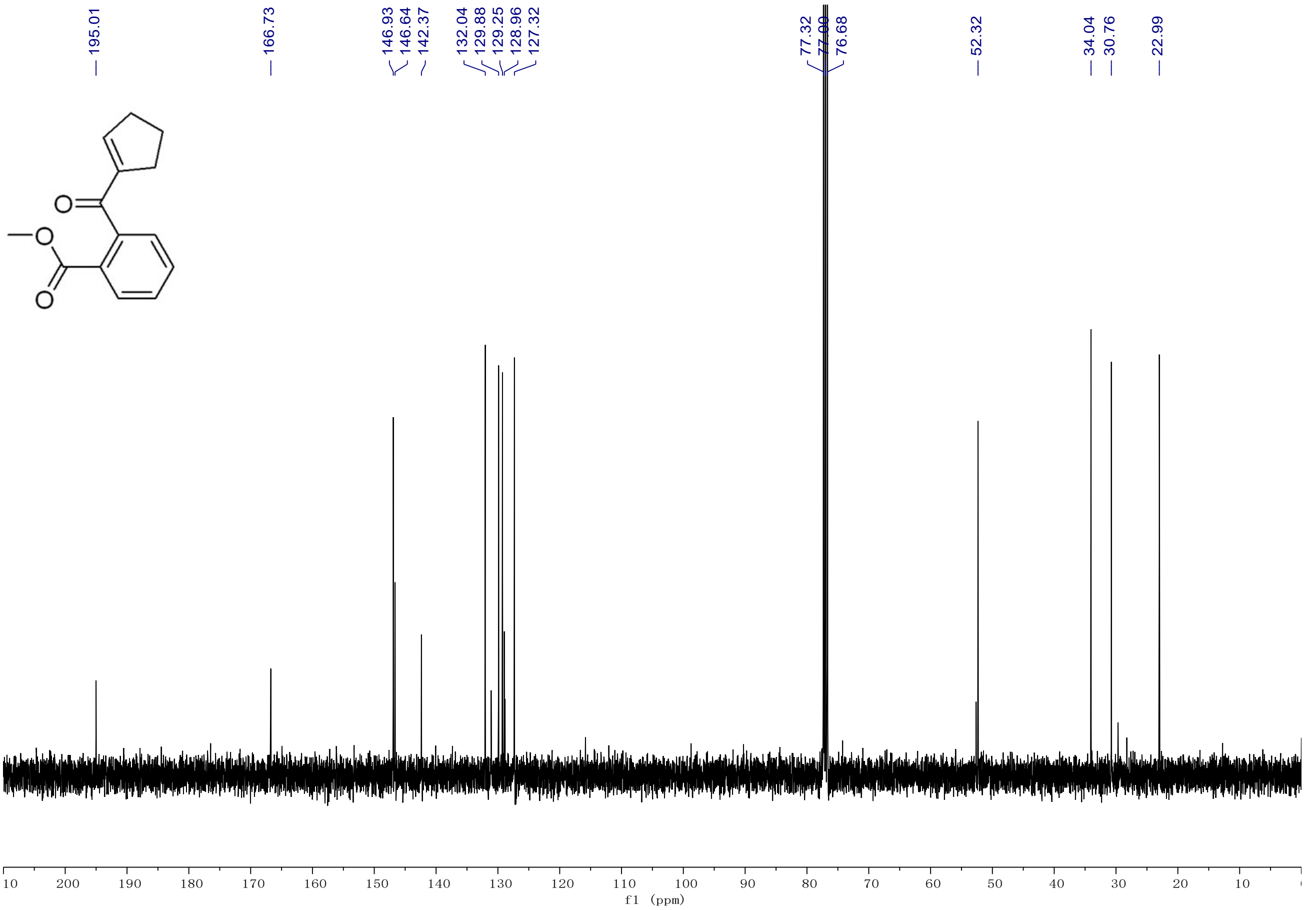
195.01 166.73 146.93 146.64 142.37 132.04 129.88 129.25 128.96 127.32 77.32 77.00 76.68 52.32 34.04 30.76 22.99
f1 (ppm)

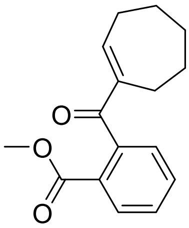

methyl 2-(cyclohept-1-ene-1-carbonyl)benzoate (7b). A colorless oil, 17 mg, 33% yield; ^{1}H NMR ($CDCl_3$, 400 MHz, TMS) δ 7.96 (dd, J = 7.8, 1.3 Hz, 1H), 7.61-7.51 (m, 1H), 7.51-7.42 (m, 1H), 7.32-7.28 (m, 1H), 6.41 (t, J = 6.6 Hz, 1H), 3.82 (s, 3H), 2.73-2.65 (m, 2H), 2.25 (q, J = 6.2 Hz, 2H), 1.87-1.76 (m, 2H), 1.64-1.55 (m, 2H), 1.55-1.48 (m, 2H); ^{13}C NMR (CD_2Cl_2, 100 MHz, TMS) δ 198.6, 166.5, 148.9, 147.0, 142.4, 132.0, 129.9, 128.9, 127.7, 52.3, 32.1, 29.3, 26.0, 25.9; IR (neat) ν 2921, 2848, 1722, 1655, 1635, 1272, 1125, 1073, 958, 772, 736, 716 cm^{-1}; HRMS (ESI) Calcd. for $C_{16}H_{18}O_3Na^+$ Requires: 281.1148, Found: 281.1146.

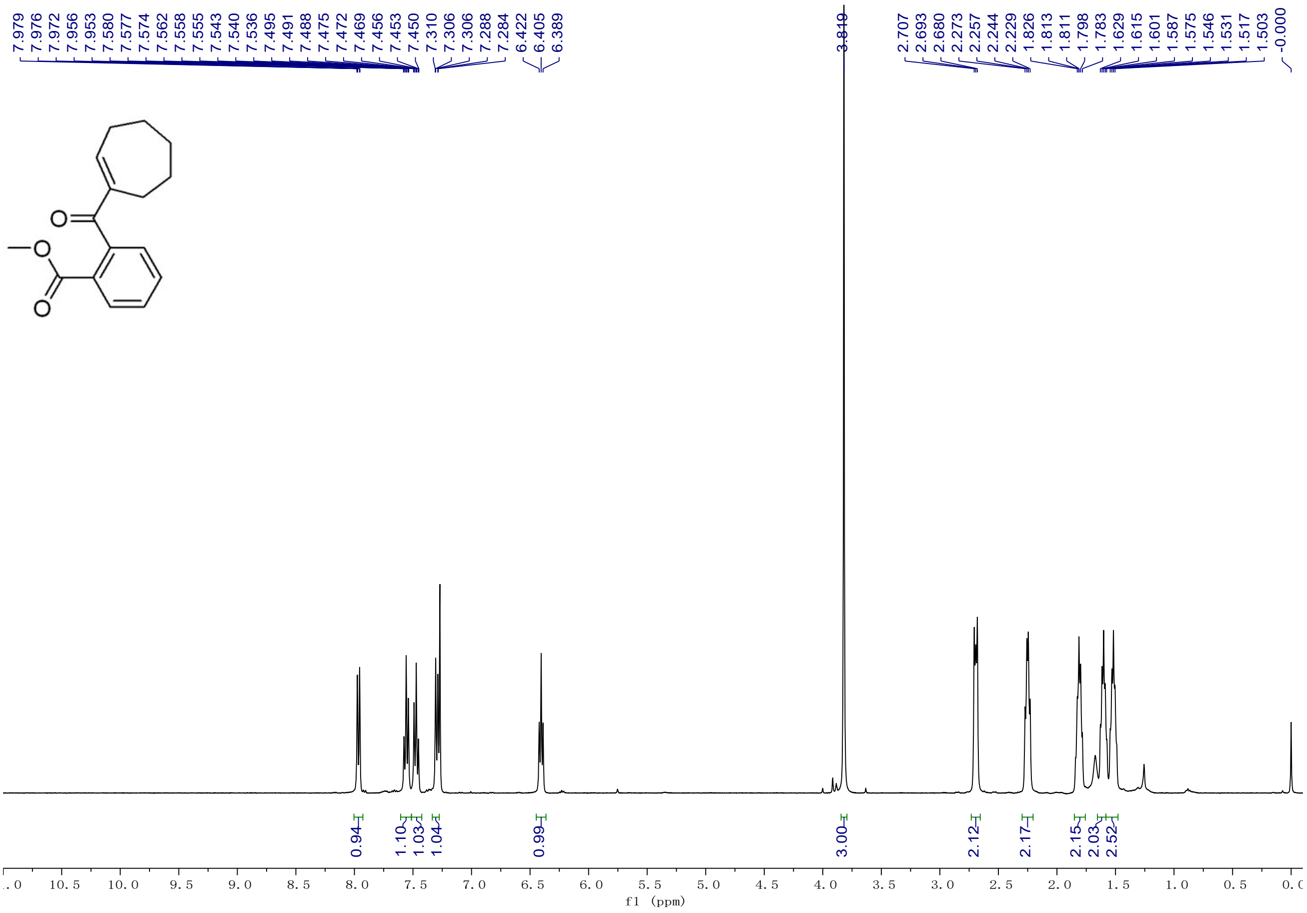

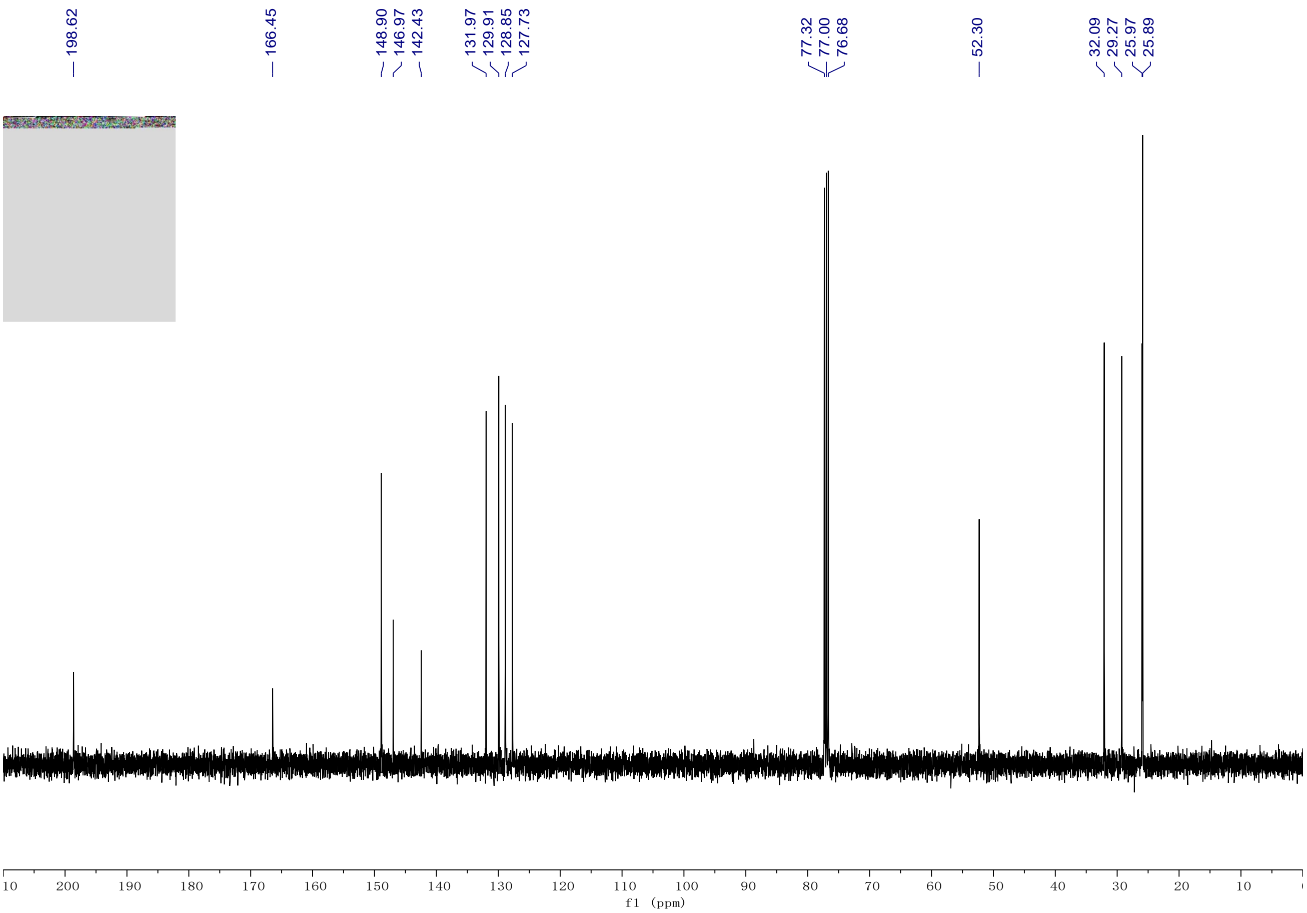

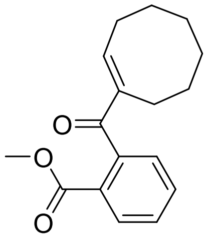

methyl (E)-2-(cyclooct-1-ene-1-carbonyl)benzoate (7c). A colorless oil, 22 mg, 40% yield; ^{1}H NMR ($CDCl_3$, 400 MHz, TMS) δ 7.99 (dd, J = 7.9, 1.5 Hz, 1H), 7.60-7.52 (m, 1H), 7.52-7.43 (m, 1H), 7.30-7.25 (m, 1H), 6.30-6.21 (m, 1H), 3.80 (s, 3H), 2.68-2.60 (m, 2H), 2.34-2.24 (m, 2H), 1.76-1.49 (m, 8H); ^{13}C NMR ($CDCl_3$, 100 MHz, TMS) δ 198.2, 166.4, 147.8, 143.6, 142.5, 131.8, 130.1, 128.8, 128.7, 127.6, 52.2, 28.89, 28.87, 27.41, 27.38, 26.20, 26.17, 23.7; IR (neat) ν 2919, 2848, 1723, 1655, 1633, 1274, 1126, 1089, 1059, 752, 714 cm^{-1}; HRMS (ESI) Calcd. for $C_{17}H_{20}O_3Na^{+}$ Requires:295.1305, Found:295.1307.

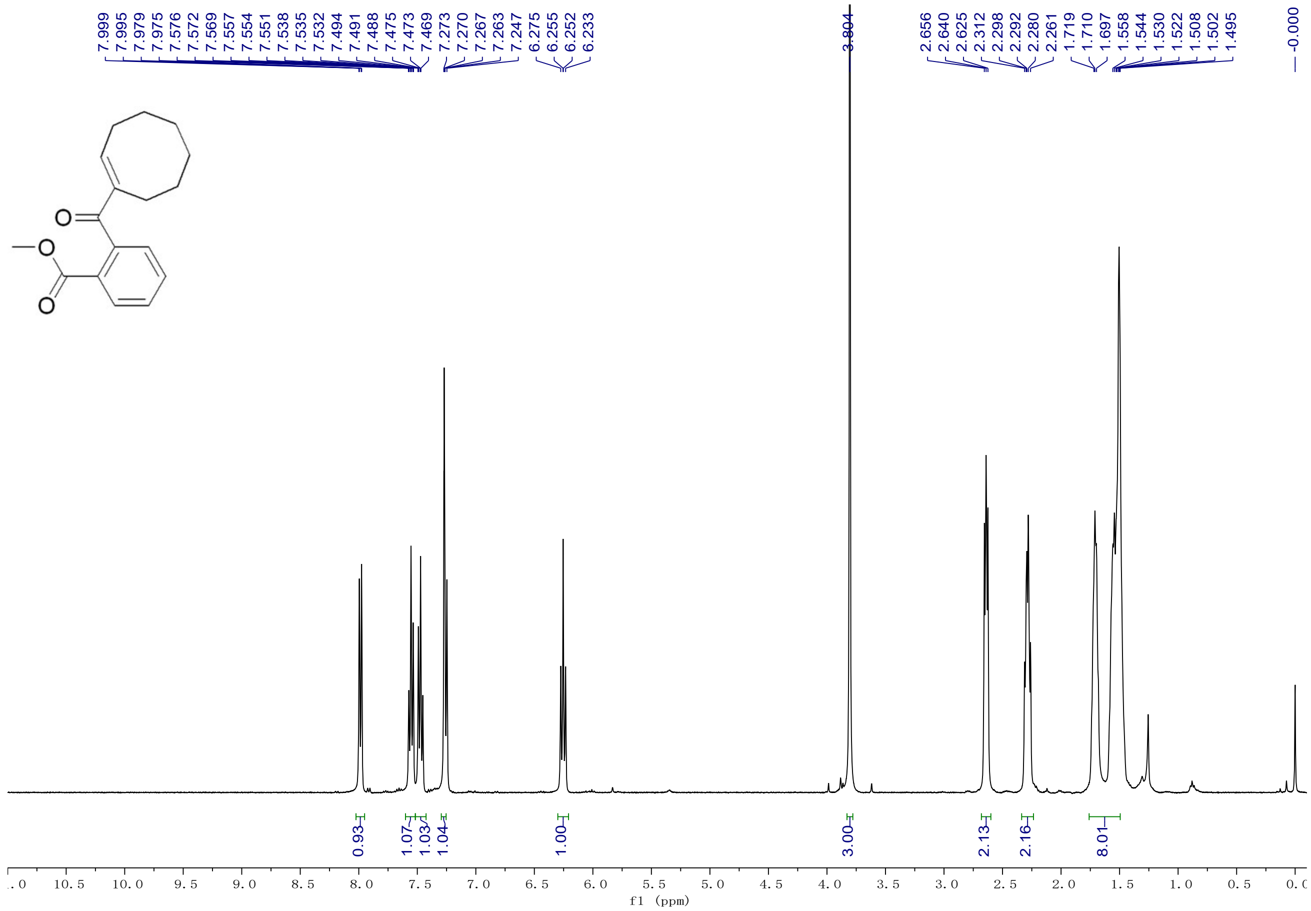
7.999
7.995
7.979
7.975
7.576
7.572
7.569
7.557
7.554
7.551
7.538
7.535
7.532
7.494
7.491
7.488
7.475
7.473
7.469
7.273
7.270
7.267
7.263
7.247
6.275
6.255
6.252
6.233
3.804
2.656
2.640
2.625
2.312
2.298
2.292
2.280
2.261
1.719
1.710
1.697
1.558
1.544
1.530
1.522
1.508
1.502
1.495
-0.000
O
O
O
0.93
1.07
1.03
1.04
1.00
3.00
2.13
2.16
8.01
f1 (ppm)

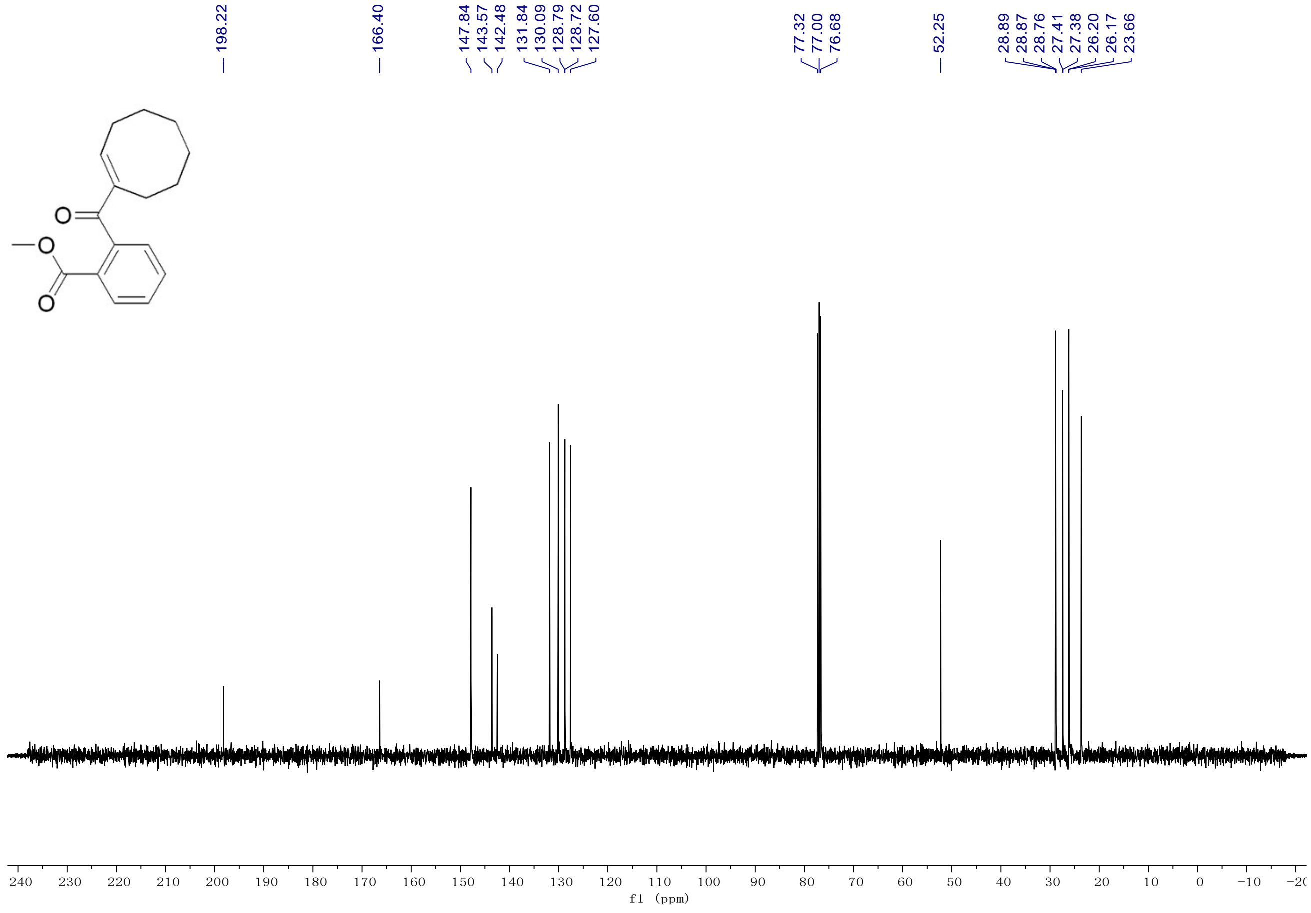
198.22
166.40
147.84
143.57
142.48
131.84
130.09
128.79
128.72
127.60
77.32
77.00
76.68
52.25
28.89
28.87
28.76
27.41
27.38
26.20
26.17
23.66
O
O
O
f1 (ppm)

(G) X-ray Crystal Data of Compounds 3ho, 3ca, 4 and 5

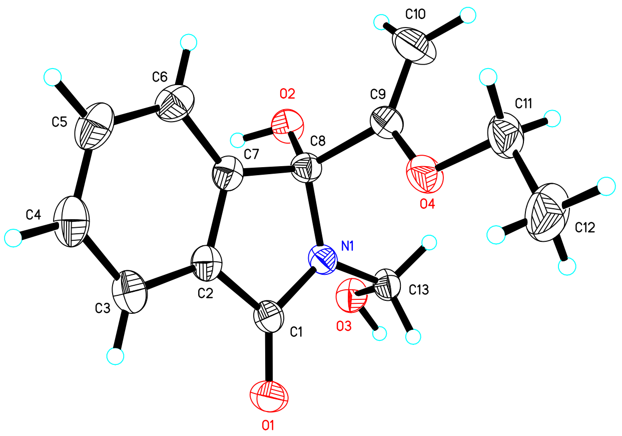

The crystal data of **3ho** have been deposited in CCDC with number 2054162. Empirical Formula: $C_{13}H_{15}NO_4$; Formula Weight: 249.26; Crystal Color, Habit: colorless, Crystal Dimensions: 0.200 x 0.150 x 0.120 mm^3; Crystal System: Monoclinic; Lattice Parameters: a = 6.8237(2)Å, b = 21.9249(6)Å, c = 8.5663(2)Å, α = 90°, β = 96.6540(10)°, γ = 90°, V = 1272.96(6)Å^3; Space group: P 21/c; Z = 4; D_{calc}= 1.301 g/cm^3; F_{000} = 528; Final R indices [I>2sigma(I)] R1 = 0.0376, wR2 = 0.0936.

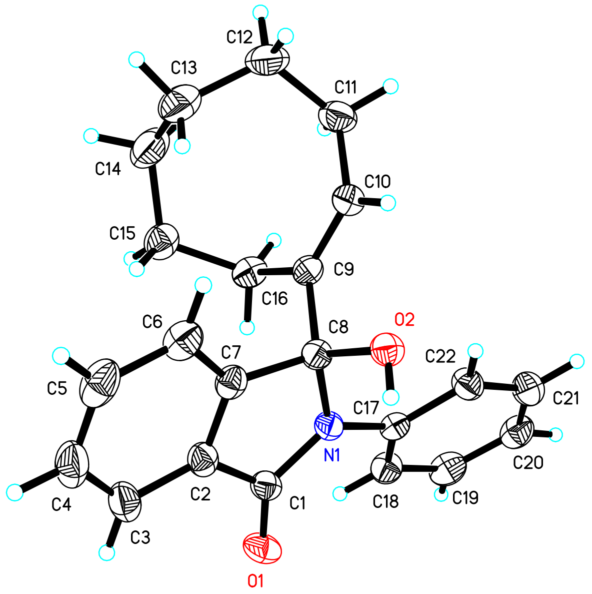

The crystal data of **3ca** have been deposited in CCDC with number 2055725. Empirical Formula: $C_{22}H_{23}NO_2$; Formula Weight: 333.41; Crystal Color, Habit: colorless, Crystal Dimensions: 0.200 x

0.150 x 0.130 mm^3; Crystal System: Monoclinic; Lattice Parameters: a = 11.9170(3)Å, b = 7.3388(2)Å, c = 20.1019(5)Å, α = 90°, β = 92.9320(10)°, γ = 90°, V = 1755.74(8)Å^3; Space group: P 21/c; Z = 4; D_{calc}= 1.261 g/cm^3; F_{000} = 712; Final R indices [I>2sigma(I)] R1 = 0.0462, wR2 = 0.1176.

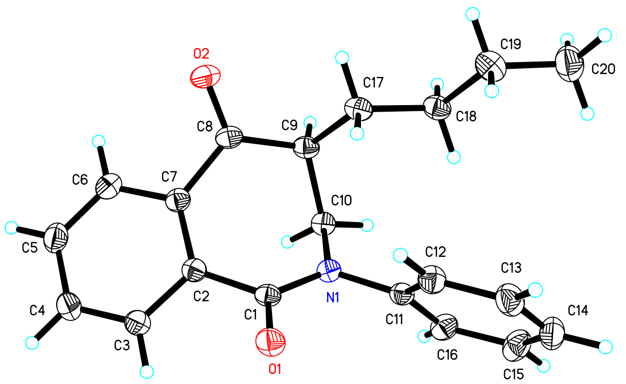

The crystal data of **4** have been deposited in CCDC with number 2058990. Empirical Formula: $C_{20}H_{21}NO_2$; Formula Weight: 307.38; Crystal Color, Habit: colorless, Crystal Dimensions: 0.200 x 0.160 x 0.110 mm^3; Crystal System: Triclinic; Lattice Parameters: a = 6.9435(2)Å, b = 11.9044(4)Å, c = 21.0418(7)Å, α = 74.1640(10)°, β = 85.9680(10)°, γ = 83.0160(10)°, V = 1659.59(9)Å^3; Space group: P -1; Z = 4; D_{calc}= 1.230 g/cm^3; F_{000} = 656; Final R indices [I>2sigma(I)] R1 = 0.0390, wR2 = 0.0875.

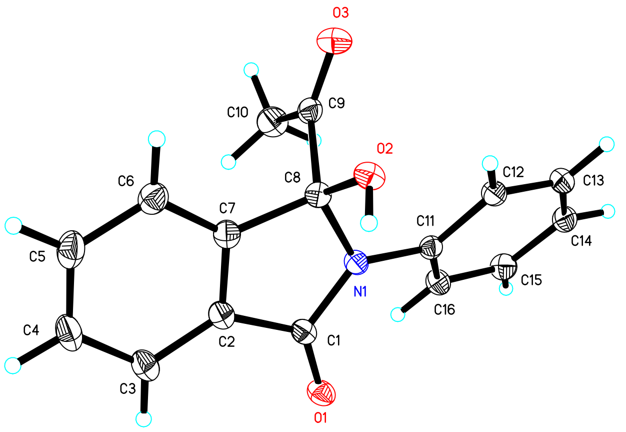

The crystal data of **5** have been deposited in CCDC with number 2048969. Empirical Formula: $C_{16}H_{13}NO_3$; Formula Weight: 267.27; Crystal Color, Habit: colorless, Crystal Dimensions: 0.200 x 0.150 x 0.130 mm^3; Crystal System: Monoclinic; Lattice Parameters: a = 8.8242(3)Å, b =

7.5306(2)Å, c = 19.3128(6)Å, α = 90°, β = 96.9800(10)°, γ = 90°, V = 1273.85(7)$Å^3$; Space group: P 21/c; Z = 4; D_{calc}= 1.394 g/cm^3; F_{000} = 560; Final R indices [I>2sigma(I)] R1 = 0.0328, wR2 = 0.0812.

(H) Radical Probe Experiment

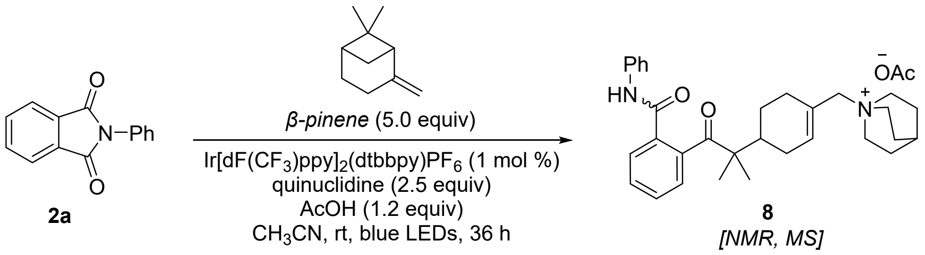

A 5 mL dry sealed tube equipped with a Teflon plug and a magnetic stirrer was charged with $Ir[dF(CF_3)ppy]_2(dtbbpy)PF_6$ (0.002 mmol, 0.01 equiv) and phthalimide **2a** (0.2 mmol, 1.0 equiv). After replacing the air in it with argon, 3.5 mL of dry acetonitrile, which has been degassed with argon, was injected under argon. Then, also under argon, 500 μL of quinuclidine acetonitrile solution (1.0 M, without oxygen), 14 μL of glacial acetic acid (0.24 mmol, 1.2 equiv) and β-pinene (1.0 mmol, 5 equiv) were injected with micro-injectors, respectively. After all the materials were added, the reaction tube was sealed with a Teflon plug under argon. Then, we placed the reaction tube under the blue light of an 8 W blue LED strip at room temperature (using a fan to maintain the temperature). After stirring for 36 hours under these conditions, the mixture was concentrated directly on a rotary evaporator. Based on LC-MS, **2a** was completely consumed and product **8** was the major product as an isomeric mixture. The corresponding residue was diluted with dichloromethane and purified by a column chromatography (SiO_2) directly using dichloromethane/methanol/AcOH (5/1/0.05) as the eluent to obtain the crude product **8**, which was further recrystallized from dichloromethane to give relative pure **8** (17 mg, 16% yield). The corresponding characterization data were shown below. Mass Spectra (HRMS) were recorded by ESI method. ^{1}H NMR spectra and ^{13}C NMR spectra were recorded at 400 and 100 MHz in CD_3OD. The Mass data was consistent with the calculated data of the corresponding quaternary ammonium salt **8** and the speculation was further supported by ^{1}H NMR, ^{13}C NMR, HMQC and HMBC. All the spectroscopic data obtained above fully support the structure of product **8**.

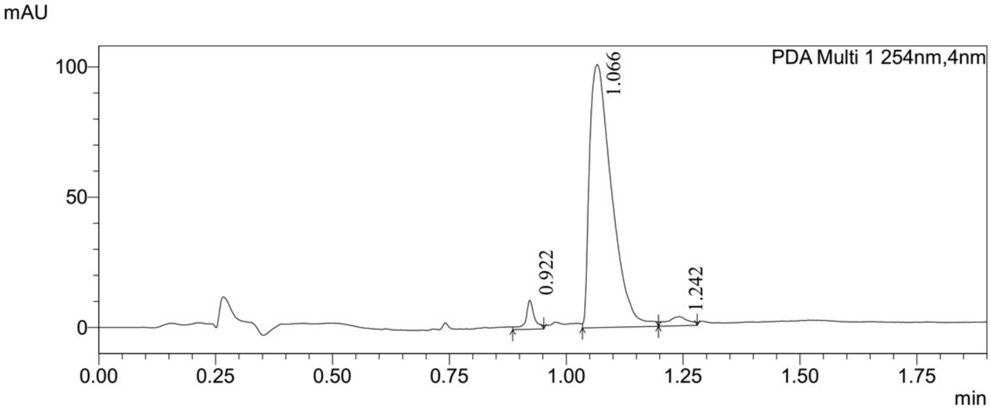

Peak Table

PDA Ch1 254nm

Peak#	Ret. Time	Height	Height%	Area	Area%
1	0.922	11096	9.607	13917	3.890
2	1.066	100900	87.356	332729	93.010
3	1.242	3508	3.037	11091	3.100
Total		115505	100.000	357736	100.000

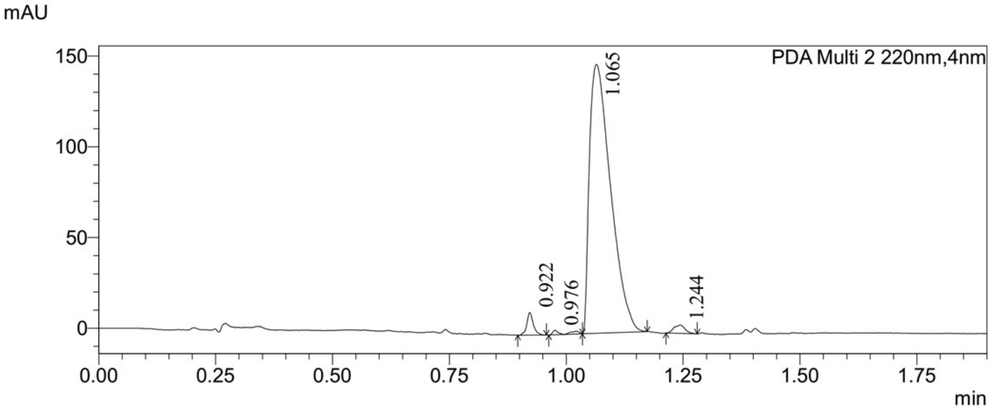

Peak Table

PDA Ch2 220nm

Peak#	Ret. Time	Height	Height%	Area	Area%
1	0.922	12405	7.412	12081	2.458
2	0.976	2434	1.454	4104	0.835
3	1.065	147914	88.379	467626	95.126
4	1.244	4610	2.755	7776	1.582
Total		167364	100.000	491587	100.000

MS Chromatogram

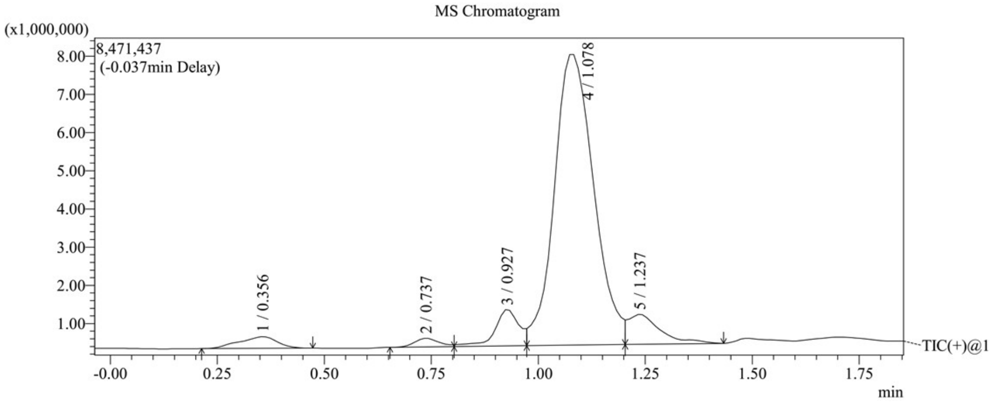

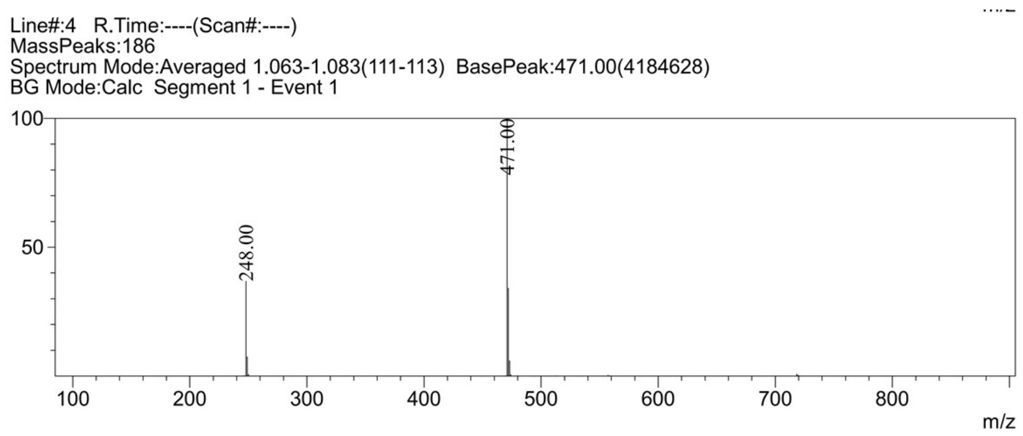

Figure S3. LC-MS spectra report

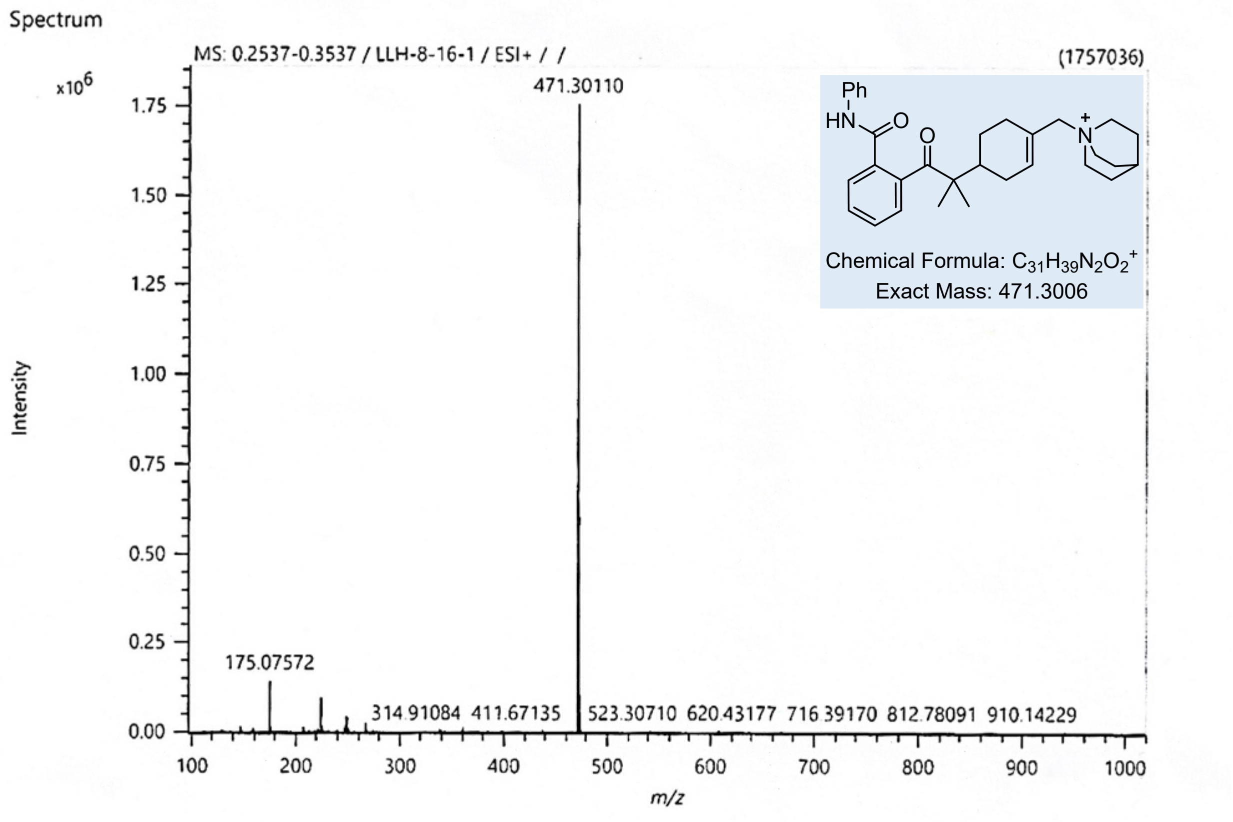

Figure S4. HR-MS spectra

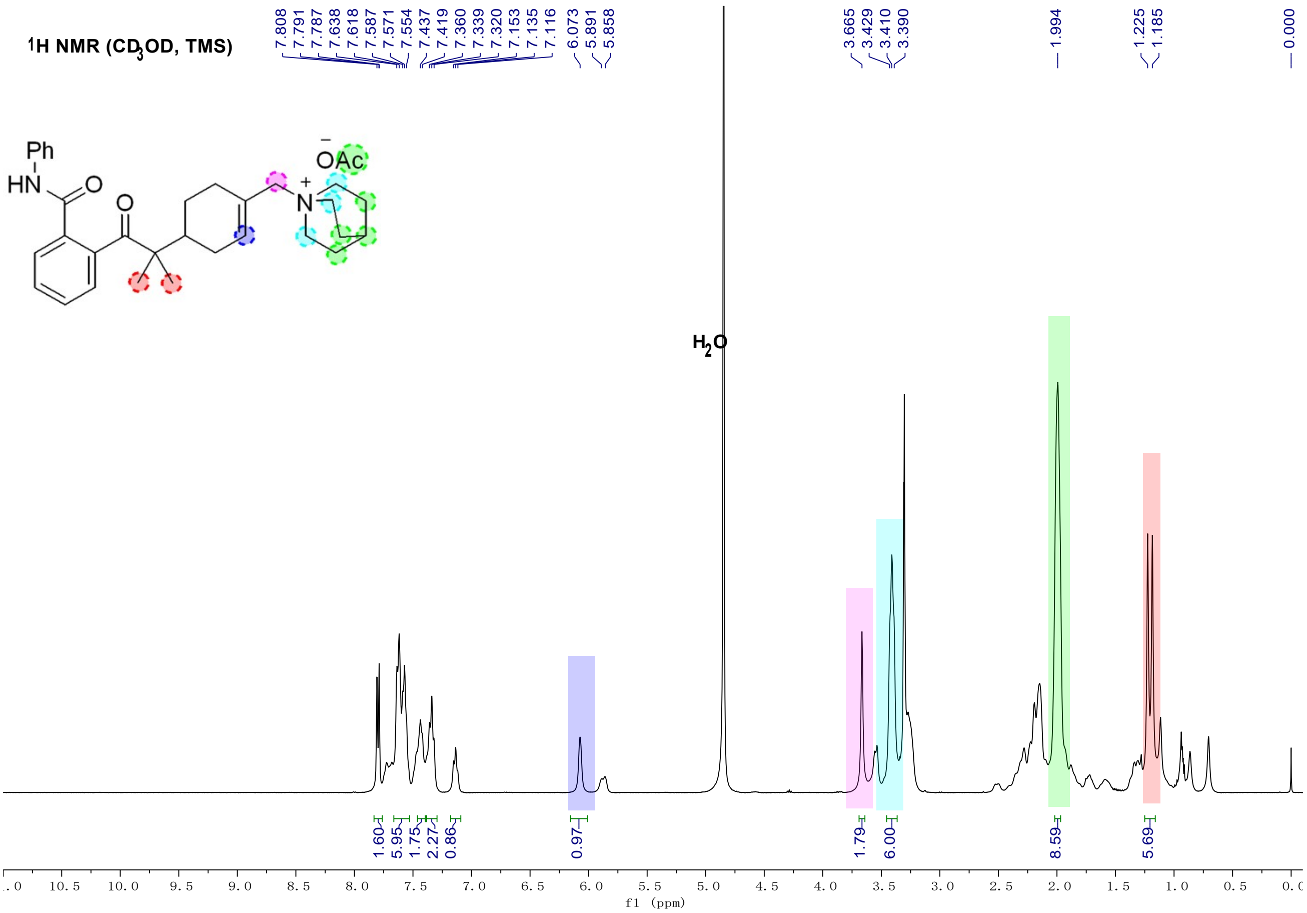
1H NMR (CD3OD, TMS)
7.808
7.791
7.787
7.638
7.618
7.587
7.571
7.554
7.437
7.419
7.360
7.339
7.320
7.153
7.135
7.116
6.073
5.891
5.858
3.665
3.429
3.410
3.390
1.994
1.225
1.185
0.000
Ph
HN
O
O
OAc
N
H2O
1.60
5.95
1.75
2.27
0.86
0.97
1.79
6.00
8.59
5.69
f1 (ppm)

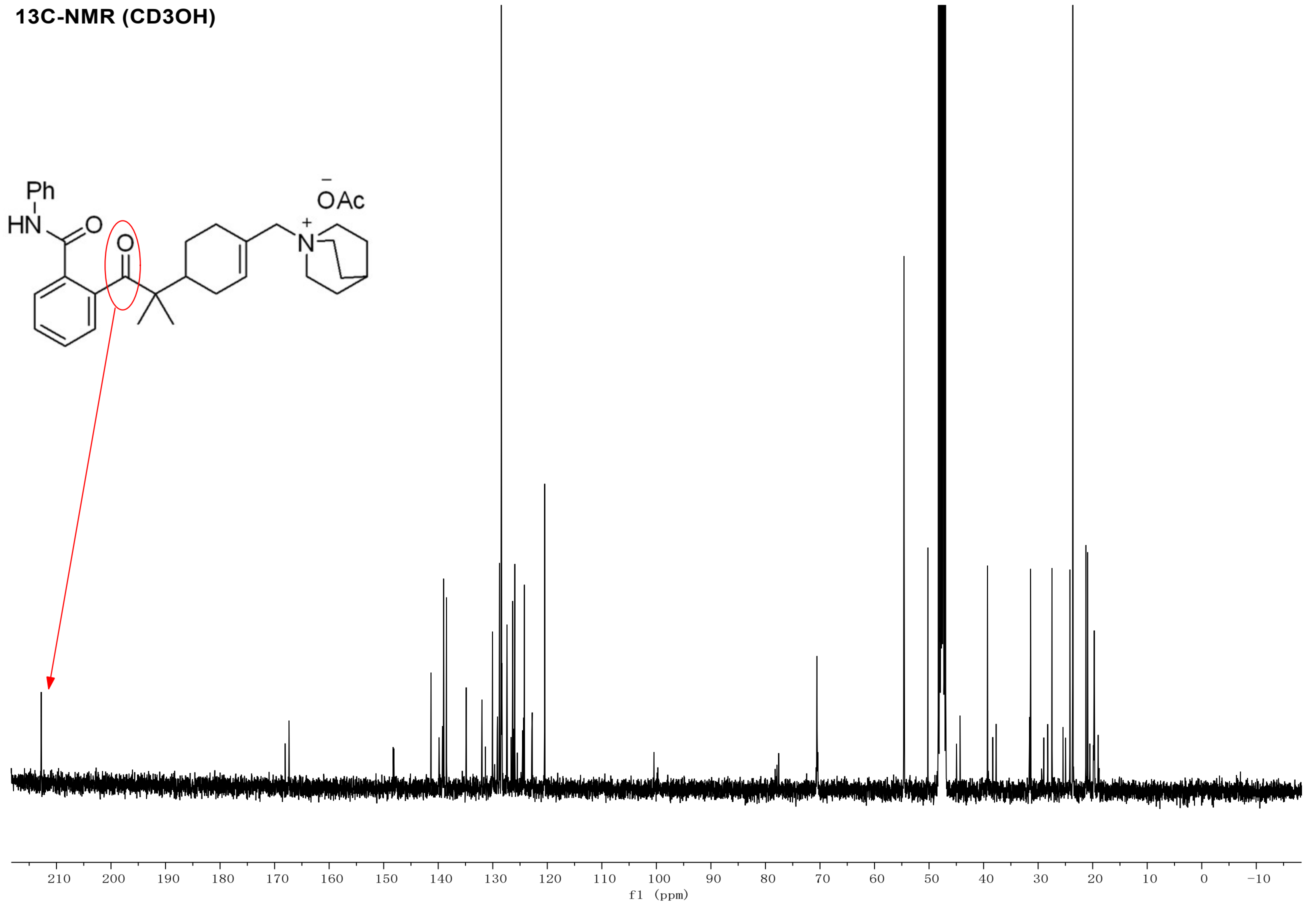
13C-NMR (CD3OH)
Ph
HN
O
O
OAc
N
f1 (ppm)

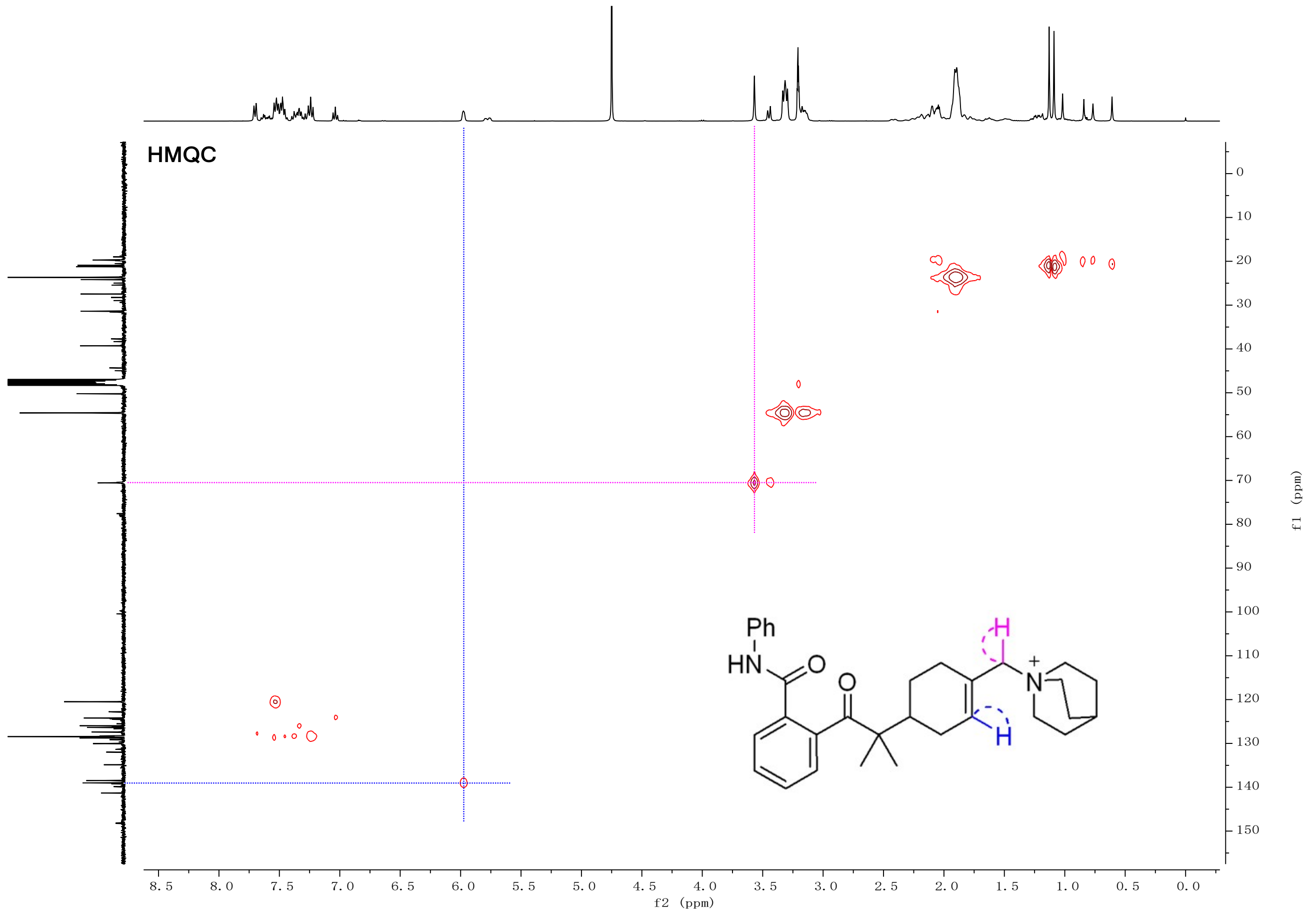
HMQC
f2 (ppm)
f1 (ppm)
Ph
HN
O
O
H
H
N

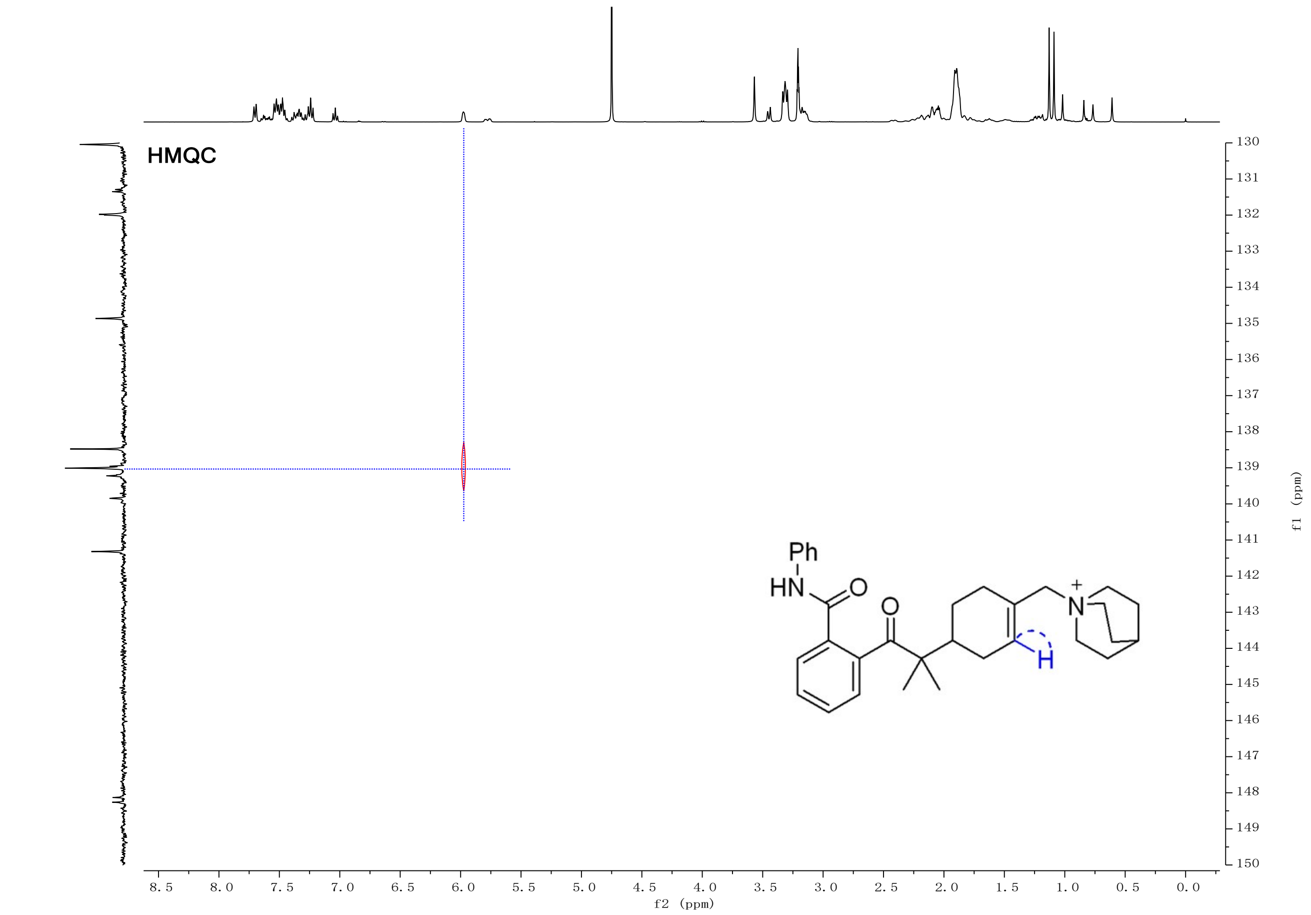
HMQC
f2 (ppm)
f1 (ppm)
Ph
HN
O
O
H
N

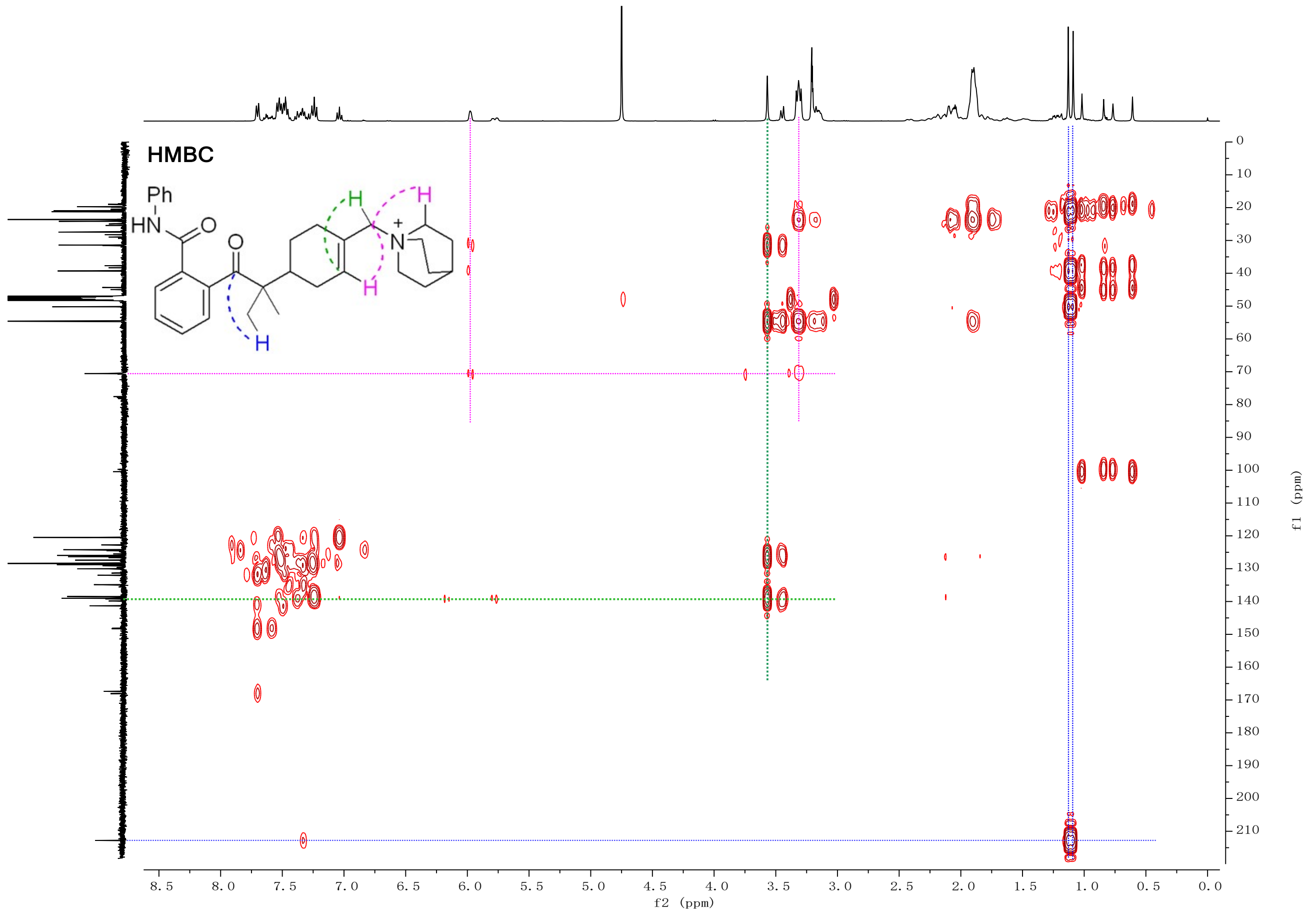

HMBC
Ph
HN
O
O
H
H
N
+
H
H
f2 (ppm)
f1 (ppm)

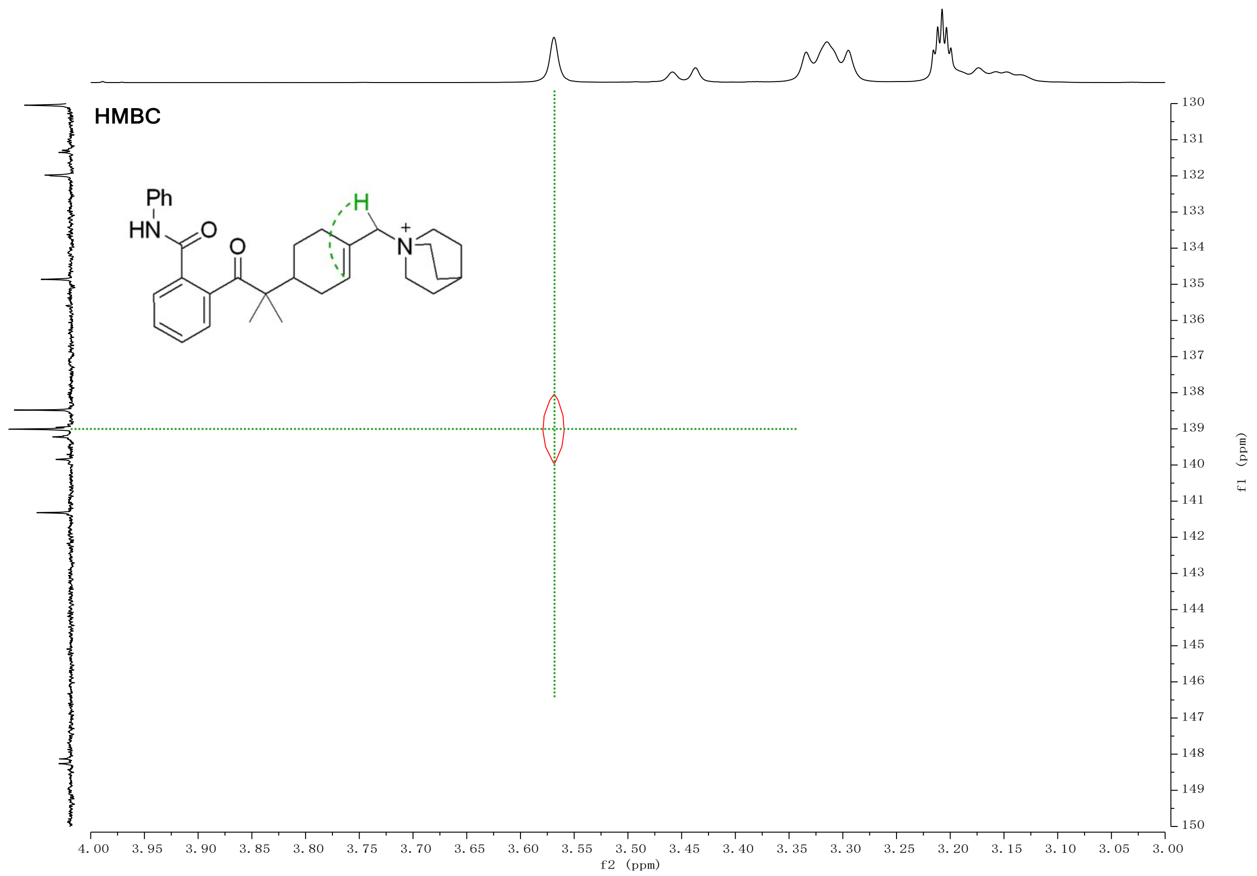

HMBC
Ph
HN
O
O
H
N
+
f2 (ppm)
f1 (ppm)

(I) Kinetic Isotope Effect (KIE) Study of the Reaction between 2a and 1k

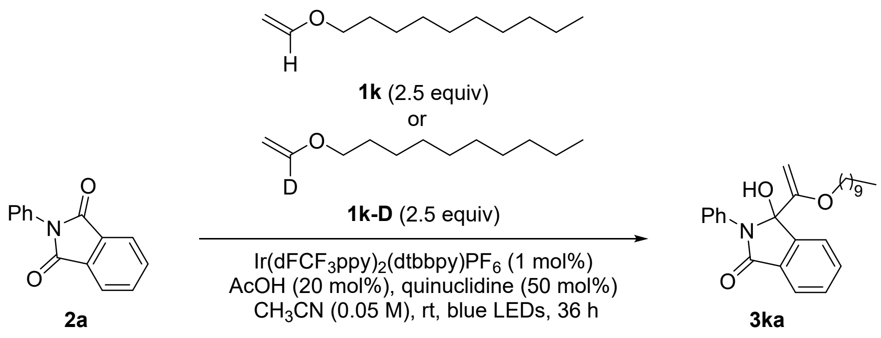

A 10 mL dry sealed tube equipped with a Teflon plug and a magnetic stirrer was charged with Ir[dF(CF_3)ppy]$_2$(dtbbpy)PF_6 (0.002 mmol, 0.01 equiv) and phthalimide **2a** (0.2 mmol, 1.0 equiv). After replacing the air in it with argon, 4.0 mL of dry acetonitrile, which has been degassed with argon, was injected under argon. Then, also under argon, 100 μL of quinuclidine acetonitrile solution (1.0 M, without oxygen), 2.3 μL of glacial acetic acid (0.04 mmol, 0.2 equiv) and olefin **1k** or **1k-D** (0.5 mmol, 2.5 equiv) were injected with micro-injectors, respectively. After all the materials were added, the reaction tube was sealed with a Teflon plug under argon and stirred in dark for 20 min to dissolve the phthalimide **2a**. Then, still under argon, the reaction solution was divided the homogeneous mixture into three 5 mL dry sealed tubes, each tube containing 1 mL of solution. After sealing with Teflon plugs, the reaction tubes were placed under the blue light of an 8 W blue LED strip and stirred at room temperature (using a fan to maintain the temperature). Then, one tube was removed from the light every 15 min for 45 min. After adding the internal standard 1,3,5-trimethoxybenzene, the mixture was separately concentrated on the rotary evaporator. The yields of **3ka** in 15 min, 30 min and 45 min were determined by ^{1}H-NMR analysis of the crude reaction mixtures relative to the internal standard, as shown in Figure S5.

$$k_H/k_D = 0.6667/0.64 = 1.04$$

The results revealed that the broken bond with the isotope atom was not in the rate-determining step of the reaction between **2a** and **1k**.

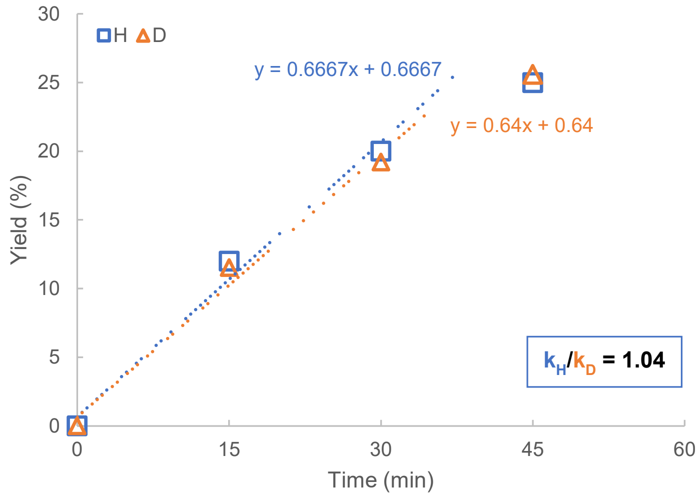

Figure S5. Kinetic Isotope Effect Study of the Reaction between 2a and 1k

(J) Computational Details

Computational methods

All DFT calculations were performed with Gaussian 16 program.[3] The geometries of all minima and transition states have been optimized at B3LYP/6-31G(d) level of theory. The subsequent frequency calculations on the stationary points were carried out at the same level of theory to ascertain the nature of the stationary points as minima or first-order saddle points on the respective potential energy surfaces. All transition states were characterized by one and only one imaginary frequency pertaining to the desired reaction coordinate. The intrinsic reaction coordinate (IRC) calculations were carried out at the same level of theory to further authenticate the transition states. The conformational space of flexible systems has first been searched manually. Thermochemical corrections to 298.15 K have been calculated for all minima from unscaled vibrational frequencies obtained at this same level. The solvent effect was estimated by the IEFPCM method with radii and nonelectrostatic terms for SMD salvation model in CH_3CN (ε = 35.688). Solution-phase single point energy calculations were performed at B3LYP/6-311+G(d,p) level based on the gas phase optimized structures.

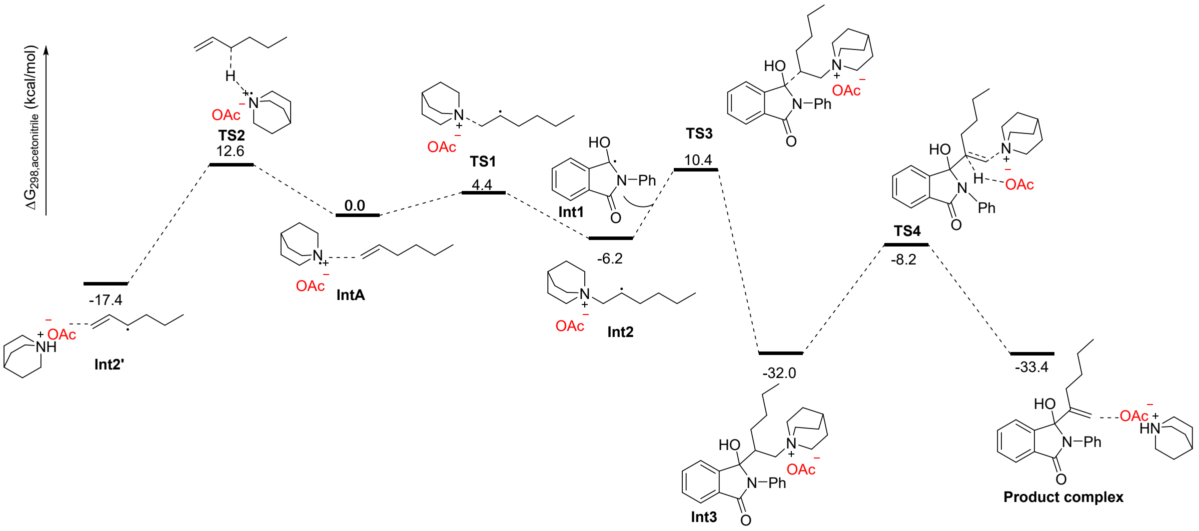

Scheme S5. The solvation Gibbs free energy profile in acetonitrile for the suggested reaction pathway.

We also carried on a DFT study to gain further insights into the whole reaction pathway. The solvation Gibbs free energy profile (ΔG_{298} (kcal/mol) in acetonitrile for the suggested reaction pathway is shown in Scheme S5. Starting from reactant complex **Int A**, we proposed two possible reaction patterns of this step. In the first reaction pattern, the addition of the quinuclidinium radical cation with olefin takes place to give the β-quinuclidinium radical intermediate **Int2**. The transition state of this process is **TS1** with an energy barrier of 4.4 kcal/mol. Another possible HAT process has to overcome an energy barrier of 12.6 kcal/mol via transition state **TS2**. Thus, we propose that the radical addition process is more preferred. Subsequently, the radical intermediate **Int2** underwent radical-radical coupling with **Int1**, giving an intermediate **Int3**. The transition state of this radical coupling process is **TS3** with an energy barrier of 16.6 kcal/mol. Passing through transition state **TS4**, the product complex **Int4** is generated with an energy barrier of 23.8 kcal/mol. In this step, the OAc^-

anion promotes the deprotonation and the elimination of quinuclidine. The DFT calculations support the proposed reaction mechanism.

Table S4. The total energies, enthalpies and free energies of all species in acetonitrile shown in Scheme S5.[a]

	E_{tot}	H_{298}	G_{298}
**Int A**	-793.8281886	-1815.459411	-793.4794348
**TS1**	-793.8321963	-793.3958184	-793.4723994
**TS2**	-793.8158657	-793.3833625	-793.4594265
**Int 2**	-793.8529882	-793.4135078	-793.4893028
**Int 2'**	-793.8608298	-793.4242686	-793.5071756
**Int1**	-744.9199448	-744.6990606	-744.7534076
**TS3**	-1538.774302	-1538.111504	-1538.216336
**Int 3**	-1538.847685	-1538.180168	-1538.283788
**TS4**	-1538.806202	-1538.144026	-1538.245972
**Product complex**	-1538.851465	-1538.183779	-1538.285723

a. Calculated at SMD/B3LYP/6-311+G(d,p)//B3LYP/6-31G(d) level

Archive Entries

Int A


```
1\1\GINC-B2173\SP\UB3LYP\6-311+G(d,p)\C15H28N1O2(2)\ROOT\07-Jun-2021\0
\\#p b3lyp/6-311+g(d,p) geom=check scrf=(iefpcm,smd,solvent=acetonitri
le)\\Title Card Required\\0,2\C,0,0.3247309298,1.2798100312,3.24369180
69\C,0,1.285201868,-0.5912317568,1.8609443941\C,0,1.5943473637,0.39863
42545,3.0030240733\H,0,0.5511518753,2.3449118467,3.1221708959\H,0,-0.0
679957435,1.143408199,4.2574118373\H,0,2.4562646346,1.0225779452,2.736
4904042\H,0,1.8657544121,-0.1490212388,3.9137970476\C,0,-0.2742950144,
1.1337492676,0.91461604\H,0,-1.1187544849,0.9375988828,0.2427659679\H,
0,-0.0013123401,2.1899666164,0.8096547161\C,0,0.938925143,0.2041520076
,0.5865277957\H,0,0.6944547501,-0.4839796565,-0.231329569\H,0,1.802852
1496,0.7963630527,0.2606726688\H,0,2.1516586589,-1.2373314967,1.678378
7288\C,0,0.0620421516,-1.4422981472,2.25461949\H,0,-0.1642638771,-2.16
72968172,1.4638700949\H,0,0.2772009434,-2.0158701531,3.1646647905\C,0,
-1.1442413764,-0.4740555908,2.4821964938\H,0,-1.9578404192,-0.68136091
92,1.7785950235\H,0,-1.5455139975,-0.5743764653,3.4969939313\N,0,-0.74
37136298,0.9302747757,2.2937840209\C,0,-7.0081136681,2.0932353324,-4.3
242627702\C,0,-6.7193568476,1.1539211816,-3.4218614184\H,0,-7.09880992
53,3.1420694948,-4.0523925412\H,0,-7.1710084186,1.8488378795,-5.372670
3471\H,0,-6.5579252502,1.4436836401,-2.3820242858\C,0,-5.2184489766,-0
.8996843453,-3.2807264222\C,0,-6.5828403886,-0.318164194,-3.7064518129
\H,0,-6.7564857927,-0.5082145801,-4.7748700508\H,0,-5.254453233,-1.993
9713799,-3.3816966188\H,0,-5.0569319042,-0.6912823367,-2.2148695702\C,
0,-4.0318159739,-0.3567039471,-4.0864685977\C,0,-2.6869866551,-0.91749
```

531,-3.6128331682\H,0,-4.0181314257,0.738733541,-4.0161551778\H,0,-4.1
774347833,-0.5905106983,-5.1515483697\H,0,-1.854369083,-0.5163248301,-
4.2032820536\H,0,-2.5103499704,-0.6636436855,-2.5610068798\H,0,-2.6590
795661,-2.0113228751,-3.7000171659\H,0,-7.3708734662,-0.8580025445,-3.
1595567935\O,0,-3.4333960028,0.3523544435,-0.0179389467\C,0,-4.2394875
198,1.1537020922,0.5356024141\O,0,-5.4212743736,1.0575664204,0.1007954
985\C,0,-3.8431760695,2.1386184201,1.590702264\H,0,-3.7921670472,3.138
109389,1.1423815365\H,0,-4.6046866448,2.1632398716,2.376805625\H,0,-2.
8605660303,1.8820272161,2.0131949258\\Version=ES64L-G16RevA.03\State=2
-A\HF=-793.8281886\S2=0.756398\S2-1=0.\S2A=0.750031\RMSD=9.164e-09\Dip
ole=3.7093077,0.1649514,2.3893116\Quadrupole=-5.3322728,-0.3585337,5.6
908065,1.9129598,6.1335251,2.2807013\PG=C01 [X(C15H28N1O2)]\\@

TS1

1\1\GINC-B2141\SP\UB3LYP\6-311+G(d,p)\C15H28N1O2(2)\ROOT\08-Jun-2021\0
\\#p b3lyp/6-311+g(d,p) geom=check scrf=(iefpcm,smd,solvent=acetonitri
le)\\Title Card Required\\0,2\C,0,0.4002420956,-2.0881399968,-0.531927
867\C,0,-1.6461633662,-2.7046848253,0.8092771756\C,0,-0.2441623239,-3.
1855966634,0.3770511801\H,0,0.4427627766,-2.4011635952,-1.5798020276\H
,0,1.414161035,-1.8352207955,-0.2099030086\H,0,-0.3104571847,-4.131760
4705,-0.1718364887\H,0,0.3838909998,-3.3647793226,1.25757552\C,0,-1.74
5259548,-1.0825034735,-1.1033238974\H,0,-2.3445275571,-0.1819179079,-0
.9234780471\H,0,-1.5715768429,-1.2287132358,-2.1743329303\C,0,-2.44004
69382,-2.317434808,-0.4519107552\H,0,-3.4683918382,-2.0464815072,-0.19
24720712\H,0,-2.4806509003,-3.1615061347,-1.1515487963\H,0,-2.16281273
57,-3.4997634804,1.3582214912\C,0,-1.5025496907,-1.4449291475,1.688229
2402\H,0,-2.4834104482,-0.9891331834,1.8545333167\H,0,-1.069983393,-1.
6938143847,2.6643807654\C,0,-0.5866667896,-0.4301416664,0.9449256303\H
,0,-1.009379914,0.5811814281,0.9204761591\H,0,0.418072781,-0.389166495
2,1.3788071584\N,0,-0.4306081361,-0.8744766944,-0.4545635193\C,0,0.428
5824125,0.7816691439,-1.5276766061\C,0,1.6461093341,1.1621362721,-1.00
71162956\H,0,-0.4596114021,1.3480257992,-1.2227998745\H,0,0.401914131,
0.2287785735,-2.4651390567\C,0,2.984847844,0.7727900492,-1.5560955101\
H,0,1.6304694438,1.8192868557,-0.1387080988\C,0,3.9735957846,0.1801565
016,-0.5233814418\H,0,2.8503986978,0.0660438846,-2.3850571159\H,0,3.45
8342178,1.6693300499,-1.990860632\H,0,4.8592631828,-0.1731189407,-1.06
95160502\H,0,3.5277909619,-0.7103639129,-0.0572643441\C,0,4.422192858,
1.1584094521,0.5704452474\C,0,5.4541593241,0.5470696573,1.5241176958\H
,0,3.5539576093,1.4993518238,1.1493494502\H,0,4.8457415224,2.055917329
1,0.0978625529\H,0,5.7607128194,1.2656871733,2.2921285063\H,0,5.048782
5219,-0.3342926534,2.0363249809\H,0,6.3550257604,0.2305473147,0.984283
3228\O,0,-3.4649982231,1.1197045864,0.1914244213\C,0,-2.8501897832,2.2
091448975,0.2197961042\O,0,-1.5977673739,2.3905449627,0.0474861229\C,0
,-3.6542972636,3.4994279762,0.4723111512\H,0,-3.1623162034,4.111293469

9,1.2352812357\H,0,-3.6850156364,4.0929332729,-0.4491995581\H,0,-4.675
0434216,3.260216896,0.7797916481\\Version=ES64L-G16RevA.03\State=2-A\H
F=-793.8321963\S2=0.767798\S2-1=0.\S2A=0.75008\RMSD=7.345e-09\Dipole=3
.7682354,-3.4643047,-0.8524702\Quadrupole=-9.533049,1.4589837,8.074065
3,11.0220508,-0.6327469,0.4451588\PG=C01 [X(C15H28N1O2)]\\@

TS2

1\1\GINC-B2177\SP\UB3LYP\6-311+G(d,p)\C15H28N1O2(2)\ROOT\09-Jun-2021\0
\\#p b3lyp/6-311+g(d,p) scrf=(iefpcm,smd,solvent=acetonitrile)\\Title
Card Required\\0,2\C,0,0.193194,-2.702602,0.011463\C,0,-2.32261,-2.618
829,0.165265\C,0,-1.081037,-3.508147,0.402475\H,0,0.620617,-3.061591,-
0.930589\H,0,0.972532,-2.761423,0.777725\H,0,-1.142269,-4.429297,-0.18
8741\H,0,-1.036999,-3.807315,1.456564\C,0,-1.028211,-1.130485,-1.38663
7\H,0,-1.292097,-0.070843,-1.449454\H,0,-0.411624,-1.398879,-2.251349\
C,0,-2.27779,-2.050172,-1.266852\H,0,-3.183645,-1.466142,-1.459865\H,0
,-2.245099,-2.858937,-2.007584\H,0,-3.23308,-3.209471,0.313754\C,0,-2.
280995,-1.41853,1.130994\H,0,-3.017189,-0.662478,0.841063\H,0,-2.49985
1,-1.721363,2.161682\C,0,-0.870918,-0.784265,1.040628\H,0,-0.925309,0.
301894,0.975668\H,0,-0.239435,-1.067024,1.890484\N,0,-0.166513,-1.2772
78,-0.179866\C,0,0.80418,2.717804,-0.569653\C,0,1.481657,1.734574,0.12
9336\H,0,0.559906,3.664975,-0.106478\H,0,0.763047,2.68878,-1.651704\H,
0,1.592559,1.858184,1.20839\C,0,3.125246,-0.222915,0.190492\H,0,1.9832
2,0.534871,-1.542712\H,0,3.119619,-1.284561,-0.096843\H,0,3.01838,-0.2
02974,1.285539\C,0,4.488626,0.383243,-0.188967\C,0,5.669139,-0.3193,0.
48863\H,0,4.487567,1.449954,0.072448\H,0,4.608749,0.33737,-1.280403\H,
0,6.624604,0.127265,0.190513\H,0,5.597068,-0.25236,1.581302\H,0,5.7033
32,-1.383839,0.224712\C,0,-2.188369,2.561491,0.089421\O,0,-1.215538,2.
078987,-0.629564\O,0,-3.12747,1.863112,0.488514\H,0,0.929408,-0.355282
,-0.315439\C,0,1.93508,0.493832,-0.445643\C,0,-2.153125,4.05615,0.4107
84\H,0,-1.985415,4.641276,-0.500011\H,0,-3.092475,4.36055,0.877201\H,0
,-1.330877,4.281721,1.101186\\Version=ES64L-G16RevA.03\State=2-A\HF=-7
93.8158657\S2=0.75619\S2-1=0.\S2A=0.750023\RMSD=8.552e-09\Dipole=2.644
7763,-2.3889112,-0.0262093\Quadrupole=-13.7109233,9.6662373,4.044686,8
.8468108,1.8833378,1.1409722\PG=C01 [X(C15H28N1O2)]\\@

Int 2

1\1\GINC-B2136\SP\UB3LYP\6-311+G(d,p)\C15H28N1O2(2)\ROOT\05-Jun-2021\0
\\#p b3lyp/6-311+g(d,p) geom=check scrf=(iefpcm,smd,solvent=acetonitri
le)\\Title Card Required\\0,2\C,0,-0.147563921,-2.3170242115,-0.448946
3321\C,0,-2.4063460067,-2.42503224,0.6503814104\C,0,-1.1049642116,-3.2
06513894,0.3799974351\H,0,-0.1256660657,-2.6014371233,-1.5052588339\H,
0,0.8763201882,-2.3316598354,-0.0681963841\H,0,-1.3120860238,-4.129869
1671,-0.171621717\H,0,-0.6329754474,-3.4953957481,1.3260657175\C,0,-1.
9342846497,-0.762948672,-1.1589138376\H,0,-2.3286803618,0.2362637032,-

0.9148828118\H,0,-1.6889413067,-0.8448070768,-2.2213379838\C,0,-2.9129
588681,-1.8570481896,-0.6866734702\H,0,-3.899949709,-1.4013377926,-0.5
600753194\H,0,-3.0089908364,-2.6600913778,-1.4281895756\H,0,-3.1564042
202,-3.0848495804,1.0982477931\C,0,-2.0938551642,-1.2401205728,1.58427
8347\H,0,-2.9375201239,-0.5437735103,1.6073223856\H,0,-1.9035325447,-1
.587875423,2.6058549102\C,0,-0.8542880188,-0.4946061153,1.0540741781\H
,0,-0.9710924951,0.5940574624,1.0494460224\H,0,0.0603190751,-0.7730536
423,1.5845386135\N,0,-0.6162398689,-0.8852234959,-0.4015944312\C,0,0.3
982874455,0.082359307,-1.040302466\C,0,1.7377039452,0.0891966653,-0.39
80895387\H,0,-0.1008058159,1.0643576563,-0.9337679223\H,0,0.4584940757
,-0.2174994157,-2.0919277826\C,0,2.9773421234,-0.2725972085,-1.1559542
731\H,0,1.8312192736,0.5899400496,0.5617320351\C,0,4.2596770548,-0.313
1482833,-0.3064700783\H,0,2.83298401,-1.2468605158,-1.6524471101\H,0,3
.1279345026,0.4404423093,-1.9887813543\H,0,5.0607469025,-0.7713076749,
-0.9024048415\H,0,4.1016910193,-0.9775823818,0.5555883682\C,0,4.732690
4866,1.062233133,0.1843779698\C,0,6.014979368,0.9874081676,1.019318876
7\H,0,3.9418404017,1.5426706871,0.7754119605\H,0,4.8971334405,1.714005
3933,-0.685265507\H,0,6.3342168021,1.9824117665,1.3484381016\H,0,5.870
2032136,0.3711244625,1.9152782807\H,0,6.8383616435,0.5460451872,0.4441
835482\O,0,-3.1747154956,1.6566605137,0.0959325588\C,0,-2.3221820316,2
.5825117788,0.187675777\O,0,-1.0601164732,2.4580701699,0.108194864\C,0
,-2.8452950441,4.0134624451,0.3993879562\H,0,-2.2654518715,4.518236218
3,1.1796360606\H,0,-2.7077883827,4.5899132927,-0.524324096\H,0,-3.9069
880137,4.0138437793,0.6599014964\\Version=ES64L-G16RevA.03\State=2-A\H
F=-793.8529882\S2=0.75402\S2-1=0.\S2A=0.750012\RMSD=1.815e-09\Dipole=2
.214465,-4.3153285,-0.7082978\Quadrupole=-7.9903382,-0.2900266,8.28036
48,11.1784792,-0.4651303,0.6049321\PG=C01 [X(C15H28N1O2)]\\@

Int 2’

1\1\GINC-B2111\SP\UB3LYP\6-311+G(d,p)\C15H28N1O2(2)\ROOT\07-Jun-2021\0
\\#p b3lyp/6-311+g(d,p) geom=check scrf=(iefpcm,smd,solvent=acetonitri
le)\\Title Card Required\\0,2\C,0,-0.7322832626,0.186833527,-0.2130981
145\C,0,-0.190428873,-0.0983082024,1.1542836144\H,0,-0.3384752373,-0.4
017103389,-1.0423728049\C,0,1.3332943773,0.1187203274,1.2756152754\H,0
,-0.7065773237,0.5237377352,1.8987208165\H,0,-0.4049359022,-1.14536332
4,1.4270566463\H,0,1.8438125616,-0.4871039464,0.5143258002\H,0,1.56381
94527,1.1666655714,1.0409564338\C,0,-1.7022554215,1.1420152584,-0.4952
170523\C,0,-2.2280285093,1.3971779261,-1.7528894832\H,0,-2.0828615577,
1.7285979654,0.3438527733\H,0,-2.991904597,2.1540002978,-1.9005430141\
C,0,1.8772781121,-0.2341683181,2.6634442129\H,0,1.6856030344,-1.286189
7585,2.908060437\H,0,2.9593342123,-0.0703208402,2.7214039558\H,0,1.405
2428073,0.3775910483,3.4421220454\H,0,-1.8994943736,0.8330574283,-2.62
13092753\C,0,2.3208556208,1.7830247289,-5.1094163308\C,0,2.6361374407,
1.341710505,-2.7496880409\C,0,0.9885699336,2.9671189144,-3.4681196864\

C,0,3.4071333293,2.898899145,-5.0919451398\H,0,2.7384769886,0.79726667
05,-5.3303922825\H,0,1.5502022647,1.9878151958,-5.8608466353\H,0,2.091
8640428,1.2400624733,-1.804614031\H,0,3.0427984394,0.3587314349,-3.003
1820954\C,0,3.7442274516,2.4311988352,-2.6545854507\C,0,2.0365738101,4
.1176902992,-3.3794406194\H,0,0.4459818456,2.8351559747,-2.5266538705\
H,0,0.2421224276,3.1650901233,-4.2449429304\C,0,3.4300888104,3.5324578
358,-3.6864894914\H,0,4.3935557462,2.4843326421,-5.3325465702\H,0,3.19
14821436,3.6658897252,-5.8459416302\H,0,3.7830918826,2.860863892,-1.64
60912403\H,0,4.7322880555,1.9997410319,-2.8557401821\H,0,2.0323671809,
4.5711600174,-2.3807240864\H,0,1.798898341,4.9165366541,-4.0924931232\
H,0,4.1886338519,4.3220864533,-3.6400345543\N,0,1.6395971275,1.6784227
303,-3.7961232895\H,0,0.6140289041,0.3637661645,-3.8597966926\O,0,-0.0
135479678,-0.4527997686,-3.8755410044\C,0,0.6735007084,-1.5385057522,-
4.242780275\O,0,1.8664062304,-1.5361962455,-4.5010131763\C,0,-0.198634
0443,-2.774709025,-4.3020115582\H,0,-0.6500915443,-2.9628905622,-3.321
9922902\H,0,-1.0192535319,-2.6192663962,-5.0104809635\H,0,0.3979277355
,-3.6352338009,-4.6073186287\\Version=ES64L-G16RevA.03\State=2-A\HF=-7
93.8608298\S2=0.775662\S2-1=0.\S2A=0.750144\RMSD=3.610e-09\Dipole=0.15
20898,1.1217165,0.5098728\Quadrupole=-3.7324243,2.2927227,1.4397016,4.
4270832,2.1429466,-4.474279\PG=C01 [X(C15H28N1O2)]\\@

Int 1

1\1\GINC-A743\SP\UB3LYP\6-311+G(d,p)\C14H10N1O2(2)\SIOC001\13-Apr-2021
\0\\#p b3lyp/6-311+g(d,p) geom=check scrf=(iefpcm,smd,solvent=acetonit
rile)\\Title Card Required\\0,2\C,0,-0.0459822412,0.1910590152,0.22416
07448\C,0,0.1683128762,-0.2316082931,1.5707259487\C,0,1.4596184003,-0.
4171029202,2.0665004867\C,0,2.5356552786,-0.1805127767,1.2171941221\C,
0,2.3255304501,0.2334523721,-0.1213320067\C,0,1.0521005929,0.420781195
,-0.6324527789\C,0,-1.4370542842,0.2761549405,0.0384666846\H,0,1.60593
21712,-0.7399071126,3.0931531998\H,0,3.5508238326,-0.3153943469,1.5789
617418\H,0,3.1868092056,0.4076329153,-0.7607650908\H,0,0.8993931097,0.
7373738446,-1.6595986827\O,0,-2.0692764827,0.585190265,-1.1082996198\H
,0,-3.0291792021,0.4628141784,-0.9923718655\N,0,-2.0893686335,-0.07067
57313,1.2096994336\C,0,-3.4933785813,-0.0434917284,1.4082338126\C,0,-4
.2478444122,1.0506142678,0.9544362086\C,0,-4.1290727994,-1.1097188268,
2.0589793062\C,0,-5.6342741374,1.0566266188,1.1214038674\H,0,-3.747819
5664,1.910359192,0.5167162981\C,0,-5.5097679509,-1.0791640027,2.239109
3851\H,0,-3.5360266849,-1.9372637454,2.4278996456\C,0,-6.2689312196,-0
.0065299119,1.7624564314\H,0,-6.2112018635,1.9058977388,0.7661695971\H
,0,-5.9973267983,-1.9049399081,2.7496091697\H,0,-7.3463013732,0.003433
7477,1.899848652\C,0,-1.1186101914,-0.420849242,2.2263128788\O,0,-1.41
60803164,-0.781612562,3.3582253685\\Version=ES64L-G09RevD.01\State=2-A
\HF=-744.9199448\S2=0.762127\S2-1=0.\S2A=0.750106\RMSD=2.362e-09\Dipol
e=-1.0698697,0.490298,-1.6048873\Quadrupole=14.2388593,-3.6013441,-10.

6375152,-0.7960955,-0.4650396,-1.6814187\PG=C01 [X(C14H10N1O2)]\\@

TS3

1\1\GINC-B2151\SP\RB3LYP\6-311+G(d,p)\C29H38N2O4\ROOT\24-Jun-2021\0\\#
p b3lyp/6-311+g(d,p) scrf=(iefpcm,smd,solvent=acetonitrile)\\Title Car
d Required\\0,1\C\C,1,1.4059428\C,2,1.39374346,1,121.50278596\C,3,1.39
381634,2,118.07642243,1,1.79242248,0\C,4,1.40704032,3,120.40723985,2,1
.57308674,0\C,5,1.39419239,4,121.62635662,3,-1.83887732,0\C,1,1.453612
48,6,132.10920967,5,178.06306648,0\H,3,1.08593558,2,120.12557718,1,179
.97977377,0\H,4,1.08598401,3,120.02662061,2,179.60150964,0\H,5,1.08687
201,4,119.16397445,3,177.1192737,0\H,6,1.08579926,5,121.09988929,4,179
.37187204,0\O,7,1.35510542,1,121.2119604,6,-17.30849649,0\H,12,0.97740
06,7,109.01865087,1,-164.8596185,0\N,7,1.39091791,1,107.72742928,6,-16
4.63587721,0\C,14,1.42569287,7,124.50603153,1,163.67946167,0\C,15,1.40
334401,14,118.75184309,7,-131.86827596,0\C,15,1.40052389,14,120.941819
28,7,45.99696753,0\C,16,1.39124262,15,119.6163495,14,177.93386228,0\H,
16,1.08276207,15,119.76171424,14,-2.80573922,0\C,17,1.39798815,15,119.
2746632,14,-176.58135087,0\H,17,1.08703217,15,122.79981544,14,6.299286
81,0\C,20,1.39239678,17,120.71837581,15,-1.49735027,0\H,18,1.08660648,
16,119.27131691,15,179.52907067,0\H,20,1.08279607,17,118.20958173,15,1
79.68263393,0\H,22,1.08638892,20,120.22369536,17,-179.19417967,0\C,14,
1.43512035,7,110.491893,1,-8.7494149,0\O,26,1.21648238,14,125.22326124
,7,-178.96342129,0\C,7,2.37627776,1,88.2899833,6,87.16814782,0\H,28,1.
08547411,7,90.06603158,1,-17.13544564,0\C,28,1.50359429,7,102.37463684
,1,100.41659827,0\C,30,1.54739791,28,116.30954323,7,-135.84522638,0\H,
30,1.09790728,28,110.13812692,7,96.99956005,0\H,30,1.10305772,28,108.7
0719056,7,-18.25555369,0\C,31,1.53485687,30,112.76316106,28,163.282361
87,0\H,31,1.09771478,30,110.86077774,28,-74.64779837,0\H,31,1.10040772
,30,108.82906698,28,41.89185887,0\C,34,1.53179486,31,113.10383589,30,-
179.88618121,0\H,34,1.09871781,31,109.10164078,30,-57.58531889,0\H,34,
1.09866524,31,109.25471928,30,57.92859866,0\H,37,1.09582763,34,111.300
76272,31,179.9784688,0\H,37,1.09718386,34,111.28315508,31,-60.00662028
,0\H,37,1.09735505,34,111.24256365,31,59.92366522,0\C,28,1.48174882,7,
95.19069063,1,-134.82943817,0\H,43,1.09713851,28,113.00717704,7,-76.37
336585,0\H,43,1.09450097,28,111.74960421,7,46.71118565,0\C,43,2.483538
86,28,153.29392851,7,167.17202322,0\C,46,2.48603472,43,61.73514454,28,
29.05311198,0\C,47,2.45848711,46,59.75329915,43,-72.0958385,0\C,46,1.5
4515024,43,147.06769232,28,5.71364642,0\H,46,1.09662333,43,81.02975447
,28,127.40505921,0\H,46,1.09441083,43,88.2090861,28,-123.8404189,0\H,4
7,1.08869398,46,140.40952755,43,-29.88582577,0\C,47,1.54754473,46,92.1
810234,43,-159.03669525,0\C,48,1.55038848,47,92.73014161,46,-85.927744
38,0\H,48,1.0919122,47,86.0810193,46,161.45417241,0\H,48,1.0936418,47,
142.34646788,46,46.83541384,0\C,53,1.53557519,47,109.43353941,46,23.60
491638,0\H,49,1.0949196,46,110.04945083,43,103.36657218,0\H,49,1.09574

035,46,109.96025996,43,-138.70607883,0\H,53,1.09665457,47,110.92480295
,46,145.26356142,0\H,53,1.09498487,47,108.95394931,46,-96.94136001,0\H
,54,1.09522055,48,109.88627235,47,-97.77276172,0\H,54,1.09590578,48,11
0.18433015,47,144.62068898,0\H,57,1.09517716,53,110.50363569,47,-176.1
8110005,0\N,48,1.50567348,47,35.69961182,46,35.44692278,0\H,47,1.10056
376,46,87.26140372,43,88.69533084,0\O,47,3.01294141,46,83.67356972,43,
90.97978565,0\C,67,1.25661538,47,121.53221023,46,-28.16387215,0\O,68,1
.27362245,67,126.80598069,47,-15.41394925,0\C,68,1.54175734,67,115.540
02606,47,163.49887964,0\H,70,1.09793051,68,109.66523544,67,61.25935355
,0\H,70,1.09214373,68,112.04049199,67,-176.78213428,0\H,70,1.09605163,
68,109.48805316,67,-55.5224942,0\\Version=ES64L-G16RevA.03\State=1-A\H
F=-1538.774302\RMSD=3.393e-09\Dipole=4.7597071,-0.4137948,-2.8105203\
Quadrupole=-1.2781093,0.9780055,0.3001038,-16.5048222,-12.8754276,6.59
98689\PG=C01 [X(C29H38N2O4)]\\@

Int 3

1\1\GINC-B2126\SP\RB3LYP\6-311+G(d,p)\C29H38N2O4\ROOT\10-Jun-2021\0\\#
p b3lyp/6-311+g(d,p) geom=check scrf=(iefpcm,smd,solvent=acetonitrile)
\\Title Card Required\\0,1\C,0,2.2252665708,-0.6105734283,-1.217010240
1\C,0,3.3731639318,0.014260708,-0.7413904166\C,0,4.6364675221,-0.54499
01238,-0.9015174394\C,0,4.7274347518,-1.7668286369,-1.5718403617\C,0,3
.576919972,-2.391482999,-2.0728126889\C,0,2.3114597395,-1.8180918766,-
1.9031610307\C,0,0.9984784557,0.2470683354,-0.9282173289\H,0,5.5136084
812,-0.0324136562,-0.5181794944\H,0,5.6968273414,-2.2358230741,-1.7152
549644\H,0,3.6692077834,-3.3356621778,-2.602715381\H,0,1.4268456145,-2
.3062093752,-2.3029870804\O,0,0.331423801,0.608862581,-2.1382584639\H,
0,0.9980079226,0.9918241597,-2.7324464507\N,0,1.6143549198,1.418507746
7,-0.2678521497\C,0,0.9024025201,2.6091613008,0.0986328344\C,0,0.78611
16774,3.655407633,-0.824179011\C,0,0.3208860534,2.7293732491,1.3629970
311\C,0,0.0785159096,4.8080142163,-0.4849885132\H,0,1.2562472958,3.562
8838943,-1.7988758917\C,0,-0.4101337559,3.8716088471,1.6906196116\H,0,
0.4017403808,1.9218053015,2.0812502299\C,0,-0.5272254711,4.9135553623,
0.7700336045\H,0,-0.0038971424,5.6203516856,-1.2022527734\H,0,-0.90631
71853,3.9154082649,2.6545156255\H,0,-1.0922676182,5.8058083824,1.02651
92069\C,0,3.001154241,1.3253819983,-0.1405832479\O,0,3.7390269305,2.16
41404223,0.3416112572\C,0,-0.0808147063,-0.4986326976,-0.0781376314\H,
0,-0.3176595646,-1.3742129997,-0.6959716339\C,0,0.4532300805,-0.994604
7132,1.2957012907\C,0,0.8100225886,-2.4872816886,1.3767275024\H,0,-0.3
023622607,-0.7983953842,2.0634097989\H,0,1.3324965393,-0.4010357817,1.
5757919185\C,0,1.1577512086,-2.9082465941,2.8114230703\H,0,-0.04691329
25,-3.0881990758,1.0338742587\H,0,1.6432474085,-2.7302877007,0.7038548
585\C,0,1.4703743938,-4.4019482349,2.9425241891\H,0,2.0216101594,-2.32
30457131,3.1578905095\H,0,0.3197579084,-2.6386503237,3.4672279327\H,0,
1.7156260876,-4.6684039717,3.9769469991\H,0,0.61254914,-5.0151245258,2

.6381599202\H,0,2.3226719363,-4.6898023464,2.3136238923\C,0,-1.3547772
161,0.3735956123,0.0588790058\H,0,-1.4584574284,0.783543396,1.06904881
88\H,0,-1.3526285871,1.1880452294,-0.6651428743\C,0,-3.776174926,0.638
8198441,0.2466600181\C,0,-2.8114590501,-1.6016859344,0.6676820736\C,0,
-2.8900588772,-0.6816838844,-1.6368900372\C,0,-5.1697300605,0.06888488
59,-0.0950668516\H,0,-3.5981996682,0.8212439831,1.3133529527\H,0,-3.56
79639621,1.5684985576,-0.2891083228\H,0,-2.0925829083,-2.3127468582,0.
2562809126\C,0,-4.2555831174,-2.1439540014,0.5912635505\C,0,-4.2066189
308,-1.474452435,-1.815951954\H,0,-2.0144805036,-1.2374222829,-1.97487
86229\H,0,-2.899680379,0.2704103206,-2.173310726\C,0,-5.0260568181,-1.
4041016993,-0.5136557032\H,0,-5.8206428111,0.151401698,0.7814287608\H,
0,-5.6380507176,0.6468811393,-0.9005916829\H,0,-4.2361181718,-3.221018
0681,0.3915363413\H,0,-4.7515961223,-2.0039123333,1.5579566164\H,0,-3.
9917705249,-2.5196746859,-2.0653711073\H,0,-4.773329839,-1.0546852319,
-2.6540763472\H,0,-6.0122429185,-1.8547822513,-0.6656719812\N,0,-2.684
2338722,-0.3413537834,-0.1797137974\H,0,-2.5188392788,-1.333541014,1.6
98028011\O,0,-2.0644106361,-0.8757432235,3.5153367631\C,0,-2.143391003
1,0.3598355814,3.7672381769\O,0,-2.2950014186,1.2921817929,2.919191749
1\C,0,-2.0162810227,0.7905509714,5.2382303197\H,0,-1.0442113914,1.2757
231938,5.3940365665\H,0,-2.7854869756,1.5307284015,5.4837970048\H,0,-2
.0919514733,-0.0670845601,5.9116466174\\Version=ES64L-G16RevA.03\State
=1-A\HF=-1538.8476849\RMSD=5.246e-09\Dipole=-1.8970224,-2.1041384,-5.6
650311\Quadrupole=6.9596362,1.108649,-8.0682853,-11.0877102,9.5514388,
4.2604702\PG=C01 [X(C29H38N2O4)]\\@

TS4

1\1\GINC-B2177\SP\RB3LYP\6-311+G(d,p)\C29H38N2O4\ROOT\18-Jun-2021\0\\#
p b3lyp/6-311+g(d,p) geom=check scrf=(iefpcm,smd,solvent=acetonitrile)
\\Title Card Required\\0,1\C,0,-1.3127507042,-1.4189978195,1.232914198
7\C,0,-2.6963050965,-1.5633730939,1.2238589646\C,0,-3.3216525745,-2.74
96192499,1.5935540861\C,0,-2.5123864361,-3.8107924951,2.0041371948\C,0
,-1.1195968125,-3.6616322039,2.0485613712\C,0,-0.5025348396,-2.4631615
697,1.669408082\C,0,-0.9032115395,0.0021601663,0.8427663014\H,0,-4.404
6073254,-2.826765136,1.5669220688\H,0,-2.9626141088,-4.754043692,2.301
8558444\H,0,-0.504386596,-4.4909852827,2.3885311689\H,0,0.5757990922,-
2.3553451167,1.7249463247\O,0,-0.265946145,0.6129912931,1.9748199326\H
,0,-0.8077662028,0.3915945984,2.7504182394\N,0,-2.2806116111,0.6188665
321,0.6379476362\C,0,-2.5120626752,1.9657769325,0.2478294774\C,0,-1.92
90229561,3.0289303357,0.9531447123\C,0,-3.3639335094,2.2488063941,-0.8
308528165\C,0,-2.1691693726,4.3474328408,0.564319522\H,0,-1.2951463687
,2.8120765567,1.8047639536\C,0,-3.613837573,3.5691275043,-1.2002927339
\H,0,-3.8377907596,1.4316350561,-1.3611022518\C,0,-3.0129067595,4.6249
772035,-0.5116353089\H,0,-1.7072824292,5.1603786148,1.119338826\H,0,-4
.2801774397,3.7717676123,-2.0346978269\H,0,-3.2078149138,5.6529190912,

-0.8054874039\C,0,-3.3254396419,-0.2847963504,0.8059283213\O,0,-4.5181
679368,-0.063009187,0.6561217246\C,0,0.0372137304,0.164174294,-0.36089
60836\H,0,1.1328618926,-1.2716641171,-0.5071466669\C,0,-0.5814508072,-
0.1221847583,-1.7560988714\C,0,-1.2644136865,-1.4737871177,-2.03190612
89\H,0,0.2332554972,-0.0188553882,-2.4882614373\H,0,-1.3094581565,0.66
39728466,-2.022081646\C,0,-1.630646215,-1.6348270887,-3.5147549101\H,0
,-0.6133253253,-2.3012354458,-1.7309593985\H,0,-2.1807440905,-1.567257
2942,-1.4378747797\C,0,-2.3631312018,-2.9466125055,-3.8154308891\H,0,-
2.2560690738,-0.7874448934,-3.8317973858\H,0,-0.7150792224,-1.58026236
37,-4.1219207469\H,0,-2.6051314738,-3.0379021397,-4.8810780931\H,0,-1.
751443525,-3.8137221261,-3.5366704129\H,0,-3.3027477833,-3.012781515,-
3.2533149834\C,0,0.7476743442,1.4233048468,-0.3581451606\H,0,0.7970859
224,1.9375756795,-1.3222111506\H,0,0.5317755102,2.1218787715,0.4450474
205\C,0,3.2422707549,0.7863489684,-1.2111343886\C,0,2.8469815476,0.764
9009041,1.2203206838\C,0,2.9534801858,2.8670640649,0.0015216522\C,0,4.
7615940034,0.6966580438,-0.9117349619\H,0,2.7896543599,-0.1915142276,-
1.3698758012\H,0,3.0401142292,1.396719078,-2.0973008702\H,0,2.20649007
58,1.2207104126,1.977132209\C,0,4.3576632229,0.9116697152,1.5412267671
\C,0,4.499305212,2.9640539195,0.1143007842\H,0,2.4533023009,3.30223538
7,0.8713051602\H,0,2.5711718087,3.3757203388,-0.8890836222\C,0,5.06847
99453,1.5486834135,0.3334894272\H,0,5.0532686166,-0.343654157,-0.72983
43143\H,0,5.336840788,1.0492171845,-1.7754439844\H,0,4.506765566,1.531
6001643,2.4335082026\H,0,4.7823177399,-0.074466195,1.7571231707\H,0,4.
774103123,3.6208691547,0.947017519\H,0,4.9240243033,3.4051098835,-0.79
50702841\H,0,6.1490575978,1.597935772,0.5054775194\N,0,2.5244765748,1.
4397196927,-0.0761193286\H,0,2.5541301291,-0.2804447937,1.1298877787\O
,0,2.7938529057,-2.4429225032,0.9446470518\C,0,2.4648762126,-2.7717401
783,-0.1882474499\O,0,1.61340977,-2.1042181784,-0.95751896\C,0,2.99316
4068,-4.0171556402,-0.8718157043\H,0,3.3780109714,-3.7733890058,-1.867
0935327\H,0,3.7763572773,-4.4729033092,-0.264166817\H,0,2.1735526091,-
4.7312441292,-1.009393191\\Version=ES64L-G16RevA.03\State=1-A\HF=-1538
.806202\RMSD=2.252e-09\Dipole=4.1880658,1.720826,-0.3924331\Quadrupol
e=-11.8857067,14.1824325,-2.2967258,12.9471028,-2.6365925,-1.2268616\P
G=C01 [X(C29H38N2O4)]\\@

Product complex

1\1\GINC-B2171\SP\RB3LYP\6-311+G(d,p)\C29H38N2O4\ROOT\18-Jun-2021\0\\#
p b3lyp/6-311+g(d,p) geom=check scrf=(iefpcm,smd,solvent=acetonitrile)
\\Title Card Required\\0,1\C,0,1.6855473227,-1.4341164738,-1.208843702
7\C,0,3.0692930958,-1.4011881303,-1.061014061\C,0,3.8516964918,-2.5423
073162,-1.2066428105\C,0,3.1999772442,-3.7368507729,-1.519992276\C,0,1
.8070352934,-3.7689696323,-1.6774370422\C,0,1.0219967727,-2.6183884903
,-1.5252844523\C,0,1.1042944503,-0.0353708286,-1.0412827943\H,0,4.9291
088228,-2.4881050612,-1.0826282261\H,0,3.7752950271,-4.6505569816,-1.6

429986184\H,0,1.3207941394,-4.7106038985,-1.9186980395\H,0,-0.06457621
24,-2.6539429911,-1.6107456796\O,0,0.4279098129,0.3813935535,-2.226207
3491\H,0,0.916975231,0.0181752051,-2.9827126553\N,0,2.336539997,0.7632
300236,-0.8028399477\C,0,2.3702400509,2.1715215937,-0.5915028458\C,0,1
.8563934908,3.0551041729,-1.5519311743\C,0,2.9546166819,2.6861976763,0
.5740906843\C,0,1.8955322808,4.4316934966,-1.3279837333\H,0,1.42778271
5,2.6548586766,-2.4631736055\C,0,3.0073731881,4.0642289246,0.781508615
\H,0,3.3786938532,2.0034959598,1.3017582021\C,0,2.4709197616,4.9418858
601,-0.162728427\H,0,1.492261788,5.1072371399,-2.0780792103\H,0,3.4694
291775,4.4514677928,1.6856234639\H,0,2.5118249356,6.015034921,0.002865
1332\C,0,3.5020210961,-0.0117135091,-0.7719745868\O,0,4.629795713,0.39
85243353,-0.5585877142\C,0,0.0403360679,0.0551827051,0.1036604058\H,0,
-0.6905997963,-0.7166634036,-0.1738866844\C,0,0.6263706444,-0.28535194
69,1.5030310075\C,0,0.3731025449,-1.7220432363,1.9888005724\H,0,0.2064
696956,0.4049853142,2.2462885396\H,0,1.7051435645,-0.0834131639,1.5078
504963\C,0,0.9256439995,-1.968744356,3.3987401108\H,0,-0.7039645809,-1
.9343530797,1.9716253503\H,0,0.8332257539,-2.4332948069,1.2925193384\C
,0,0.6838283467,-3.3992061832,3.8916360235\H,0,2.0040271091,-1.7528819
498,3.4142559446\H,0,0.4639270185,-1.258714944,4.1012630679\H,0,1.0800
35966,-3.5471512031,4.9030530775\H,0,-0.3871550855,-3.6333580176,3.911
8336977\H,0,1.1687444213,-4.1301077516,3.2331068629\C,0,-0.6644187411,
1.4313854607,0.0602858969\H,0,-0.2712406048,2.1034826752,0.8280239473\
H,0,-0.5262613599,1.9068429626,-0.9088323038\C,0,-2.5898665315,0.71209
32262,1.543219624\C,0,-2.8806526071,0.7528193567,-0.9216301673\C,0,-2.
6377886624,2.8647784852,0.335368284\C,0,-4.0827422004,0.9747735173,1.8
349881132\H,0,-2.4455698859,-0.3624092752,1.3730707368\H,0,-1.93377583
99,1.0867319725,2.3320898088\H,0,-2.6714495941,1.3944193191,-1.7826064
275\C,0,-4.3876763774,0.626369832,-0.6148309575\C,0,-4.1798475225,2.94
23949544,0.302660673\H,0,-2.1734313756,3.3935223662,-0.5005418411\H,0,
-2.2228608632,3.2680837774,1.2631110061\C,0,-4.750491439,1.5383155181,
0.5693876569\H,0,-4.5372560143,0.0202461604,2.1150108677\H,0,-4.209297
6976,1.6713761161,2.6729884684\H,0,-4.9647061991,0.8970895203,-1.50606
57716\H,0,-4.6091776822,-0.4146971931,-0.3631788578\H,0,-4.5224942768,
3.3131219986,-0.6698749562\H,0,-4.5219123032,3.6561280808,1.059861985\
H,0,-5.836141804,1.5898644017,0.6995702608\N,0,-2.1759600081,1.4207672
072,0.2594745008\H,0,-2.4336367226,-0.2375081988,-1.0730123395\O,0,-2.
0177994,-2.2052776227,-0.9917354347\C,0,-2.6867429003,-2.7529990011,-0
.0600313986\O,0,-3.1522954862,-2.1748186291,0.9596249899\C,0,-2.917100
9415,-4.2674981608,-0.1854449157\H,0,-3.7020565806,-4.6057152146,0.495
9255442\H,0,-3.1715527532,-4.5332988295,-1.2167896514\H,0,-1.988149316
3,-4.7958799163,0.0657350817\\Version=ES64L-G16RevA.03\State=1-A\HF=-1
538.8518646\RMSD=5.870e-09\Dipole=-1.4263678,4.1215329,0.1619788\Quadr
upole=-18.5005445,14.2835709,4.2169737,-30.3530023,2.7113208,6.5431107
\PG=C01 [X(C29H38N2O4)]\\@

(K) References

(1) Terada, M.; Tanaka, H.; Sorimachi, K. Enantioselective Direct Aldol-Type Reaction of Azlactone Via Protonation of Vinyl Ethers by a Chiral Brønsted Acid Catalyst. *J. Am. Chem. Soc.* **2009**, *131*, 3430-3431.

(2) Werner, G.; Rodygin, K. S.; Kostin, A. A.; Gordeev, E. G.; Kashin, A. S.; Ananikov, V. P. A Solid Acetylene Reagent with Enhanced Reactivity: Fluoride-Mediated Functionalization of Alcohols and Phenols.. *Green Chem.* **2017**, *19*, 3032-3041.

(3) Gaussian 16, Revision A.03, Frisch, M. J.; Trucks, G. W.; Schlegel, H. B.; Scuseria, G. E.; Robb, M. A.; Cheeseman, J. R.; Scalmani, G.; Barone, V.; Petersson, G. A.; Nakatsuji, H.; Li, X.; Caricato, M.; Marenich, A. V.; Bloino, J.; Janesko, B. G.; Gomperts, R.; Mennucci, B.; Hratchian, H. P.; Ortiz, J. V.; Izmaylov, A. F.; Sonnenberg, J. L.; Williams-Young, D.; Ding, F.; Lipparini, F.; Egidi, F.; Goings, J.; Peng, B.; Petrone, A.; Henderson, T.; Ranasinghe, D.; Zakrzewski, V. G.; Gao, J.; Rega, N.; Zheng, G.; Liang, W.; Hada, M.; Ehara, M.; Toyota, K.; Fukuda, R.; Hasegawa, J.; Ishida, M.; Nakajima, T.; Honda, Y.; Kitao, O.; Nakai, H.; Vreven, T.; Throssell, K.; Montgomery, J. A., Jr.; Peralta, J. E.; Ogliaro, F.; Bearpark, M. J.; Heyd, J. J.; Brothers, E. N.; Kudin, K. N.; Staroverov, V. N.; Keith, T. A.; Kobayashi, R.; Normand, J.; Raghavachari, K.; Rendell, A. P.; Burant, J. C.; Iyengar, S. S.; Tomasi, J.; Cossi, M.; Millam, J. M.; Klene, M.; Adamo, C.; Cammi, R.; Ochterski, J. W.; Martin, R. L.; Morokuma, K.; Farkas, O.; Foresman, J. B.; Fox, D. J. Gaussian, Inc., Wallingford CT, 2016.
